# Facile synthesis of new *N*-(aminocycloalkylene)amino acid compounds using chiral triflate esters with *N*-Boc-aminopyrrolidines and *N*-Boc-aminopiperidines†

**DOI:** 10.1039/d3ra03060a

**Published:** 2023-07-18

**Authors:** Gita Matulevičiūtė, Neringa Kleizienė, Greta Račkauskienė, Vytas Martynaitis, Aurimas Bieliauskas, Urtė Šachlevičiūtė, Rokas Jankauskas, Martynas R. Bartkus, Frank A. Sløk, Algirdas Šačkus

**Affiliations:** a Institute of Synthetic Chemistry, Kaunas University of Technology K. Baršausko g. 59 Kaunas LT-51423 Lithuania algirdas.sackus@ktu.lt; b Department of Organic Chemistry, Kaunas University of Technology Radvilėnų pl. 19 Kaunas LT-50254 Lithuania; c Vipergen ApS Gammel Kongevej 23A Copenhagen V DK-1610 Denmark

## Abstract

In this study, we prepared a series of new *N*-(aminocycloalkylene)amino acid derivatives for use in chiral building blocks. The method was based on the conversion of enantiopure α-hydroxy acid esters into the corresponding chiral triflate esters, which were displaced by a nucleophilic substitution S_N_2 reaction with aminopyrrolidine and aminopiperidine derivatives, and the inversion of the configuration to give methyl 2-[(Boc-amino)cycloamin-1-yl]alkanoates with good yield and high enantiomeric and diastereomeric purity. Synthesized 2-[(Boc-amino)piperidin-1-yl]propanoates combined with ethyl l-phenylalaninate gave new chiral *N*-Boc- and *N*-nosyl-dipeptides containing a piperidine moiety. The structures were elucidated by ^1^H-, ^13^C-, and ^15^N-NMR spectroscopy, high-resolution mass spectrometry, and X-ray crystallography analyses.

## Abstract 2

In this study, we prepared a series of new *N*-(aminocycloalkylene)amino acid derivatives for use in chiral building blocks.

## Graphical abstract

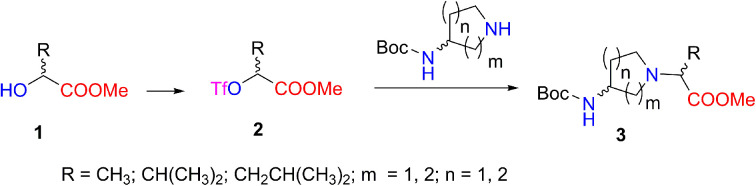

## Introduction

**Fig. 1:**
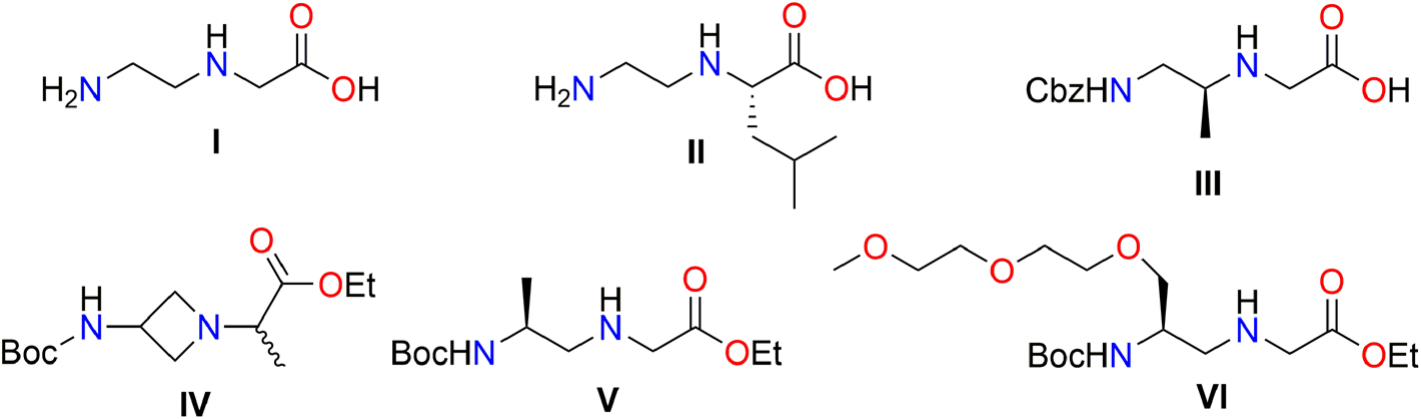
Examples of *N*-(ω-aminoalkylene)amino acid compounds I–VI.

*N*-(ω-Aminoalkylene)amino acids have many interesting biological activities and play important roles in medicinal chemistry and drug discovery for the pharmaceutical industry.^1–4^ In biochemistry, *N*-(ω-aminoalkylene)amino acids, such as *N*-(2-aminoethyl)glycine I, are used for the synthesis of peptide nucleic acids (PNAs) (Fig. 1).^5–8^

*N*-(ω-Aminoalkylene)amino acids can be synthesized in various ways: alkylation, reductive amination, and the Mitsunobu reaction.^9^ In the alkylation reaction, a molecule of ethylenediamine or its mono N-protected derivative reacts with a molecule of an α-halocarboxylic acid to form an *N*-(ω-aminoalkylene)amino acid molecule. Byt and Gilon reported a method for the alkylation synthesis of *N*-(ω-aminoalkylene)amino acids *via* the reaction of alkylenediamine (NH_2_(CH_2_)_*n*_NH_2_, *n* = 2, 3, 6) with α-haloacetic acid at 25 °C for 48 h.^10^ This experiment had a yield of 53–72%, but used a large excess of alkylenediamine. When optically active α-halogenocarboxylic acids were used as alkylating agents with ethylenediamine, a S_N_2 nucleophilic substitution occurred with the consequent inversion of configuration. For example, (*S*)-2-[(2-aminoethyl)amino]-4-methylpentanoic acid (II) was synthesized using ethylenediamine with (*R*)-2-chloro-4-methylpentanoic acid. However, the target compound gave a poor yield of 36% due to the undesired formation of the corresponding 2,3-dehydro carboxylic acid in the mixture.

It is widely known that the bromo derivative of α-haloacetic acid provides a higher yield during the nucleophilic substitution reaction because of its leaving group ability compared to that of the chloroacetic acid derivative.^9^ Feagin *et al.* reported the preparation of benzyl 2-{[2-(Boc-amino)ethyl]amino}acetate by reacting *N*-Boc-ethylenediamine and ethyl bromoacetate in the presence of triethylamine in acetonitrile at 70–80 °C for 100 min with a good yield of 72%.^11^ Sugiyama *et al.* reported that chiral diamine, (*S*)-Cbz-HNCH_2_CH(CH_3_)NH_2_, reacted with ethyl bromoacetate and yielded the target chiral compound III in the presence of potassium carbonate in DCM at room temperature, but undesired *N*,*N*-dialkylated compounds were observed in the mixture as well.^12^ Sherer and Brugger reported the details of the reaction of *tert*-butyl *N*-(azetidin-3-yl)carbamate with ethyl 2-bromopropanoate in the presence of triethylamine in DCM at room temperature for 13 h, a sequence which provided ethyl 2-[3-(*N*-Boc-amino)azetidin-l-y])propanoate IV at a yield of 44%.^13^ The compound IV block was used to prepare polycyclic Toll-like receptor (TLR) antagonists useful in the treatment of immune disorders.^14^

The two most commonly used direct reductive amination methods are described below. The first method utilizes hydride reducing agents, particularly sodium cyanoborohydride – NaBH_3_CN.^15^ Manna *et al.* reported that the treatment of (2*S*)-2-(*N*-Boc-amino)propanal with ethyl glycinate hydrochloride in methanol gave the corresponding imine which, after the addition of NaBH_3_CN and acetic acid, gave the final chiral glycine ester derivative V with a good yield 68%.^16^ The second method is catalytic hydrogenation with platinum, palladium, or nickel catalysts.^17^ For example, *N*-(2-aminoethyl)glycine (I) was prepared by the reaction of diaminoethane with glyoxylic acid monohydrate and over Pd/C in ethanol under hydrogen gas at atm. pressure and room temperature.^18^ Dueholm and co-workers reported that the treatment of *N*-Boc-aminoaldehyde with methylglycinate hydrochloride in a solvent containing KOAc and a catalyst Pd/C under a hydrogen atmosphere afforded methyl-*N*-(2-Boc-aminoethyl)glycinate (71%).^19^ The difficulty of obtaining amino aldehydes as compounds has been noted, as has their instability.^20^

Falkevich *et al.* reported on the development and synthesis of *N*-(ω-aminoalkylene)amino acid derivatives from *N*-Boc-β-amino alcohols with *N-o*-nitrobenzenesulfonyl-protected (*o*-NBS-protected) amino acid esters using the Mitsunobu reaction.^21^ For example, the treatment of (2*S*)-2-(methylamino)propan-1-ol with *o*-NBS-Gly-OEt under Mitsunobu conditions followed, by deprotection with thiophenol, yielded the chiral glycine ester derivative V.^16^ Sabu *et al.* used the Mitsunobu reaction for the prepared *N*-(2-aminoethyl)glycine derivative VI containing the diglyme moiety.^22^

Herein, we report the design and preparation of methyl 2-[(*N*-Boc-amino)cycloaminyl]alkanoates from chiral triflate esters with chiral 3-Boc-aminopyrrolidine, 3-Boc-aminopiperidine and achiral 4-Boc-aminopiperidine. Such amino acid derivatives offer valuable properties as isosteres, new conformationally restricted chiral amino acids, and building blocks that can be used as potentially biologically active substances and peptides.^23–27^

## Results and discussion

**Scheme 1:**
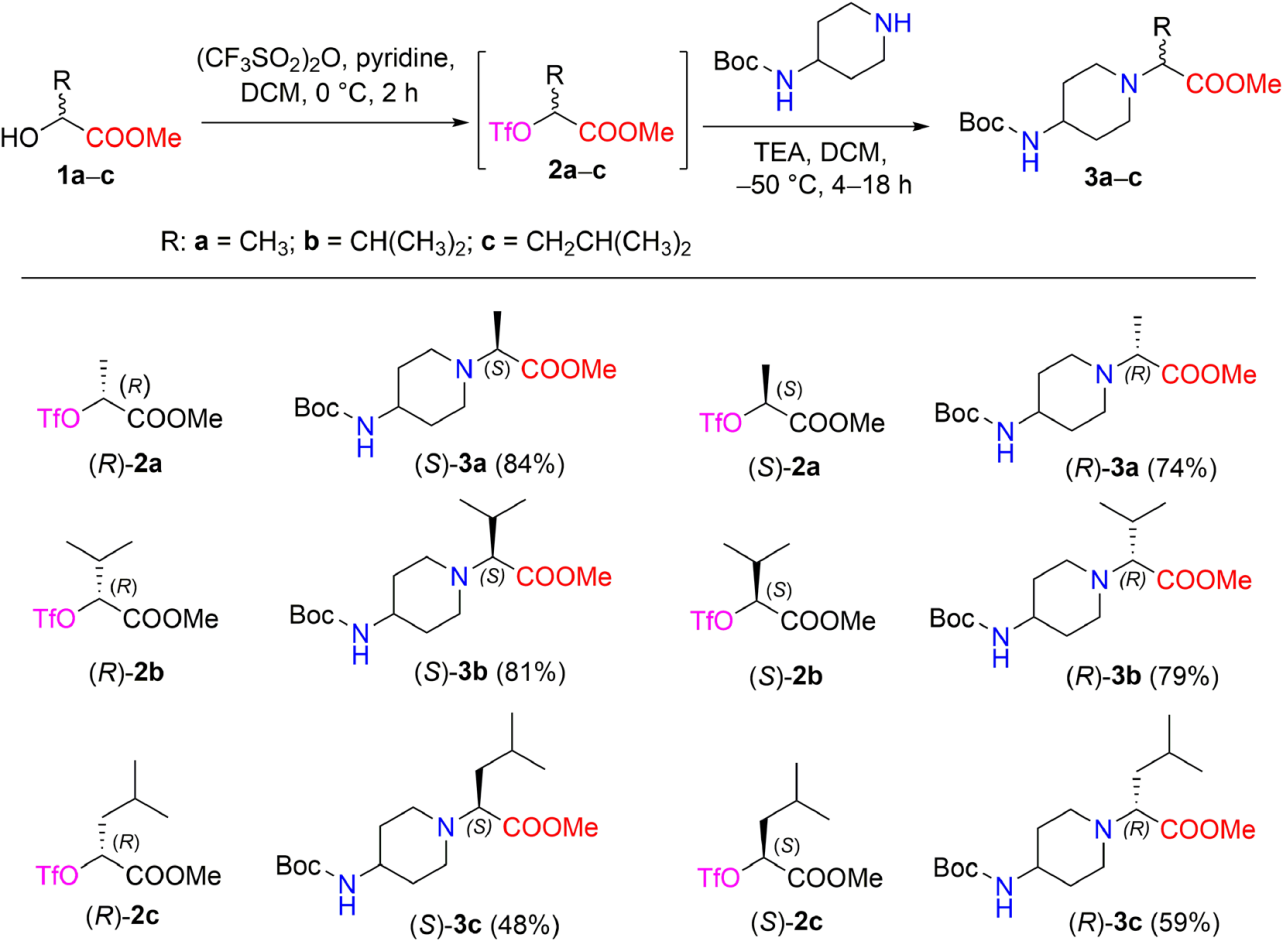
Enantiomers (*S*)- and (*R*)-3a–c synthesized from chiral triflates (*S*)- and (*R*)-2a–c with 4-Boc-aminopiperidine. *Yields are the overall yields of two steps.

The synthetic strategy of methyl 2-[(Boc-amino)cycloaminyl]alkanoates is outlined in Scheme 1. The starting (*R*)- and (*S*)-2-hydroxy acid esters 1a–c used in this study are commercially available or were prepared from their acid form (see Experimental section). The synthetic sequence was started by transforming α-hydroxy carboxylates (*R*)-1a and (*S*)-1a into the triflate esters, methyl(2*R*)- and (2*S*)-2-[(trifluoromethanesulfonyl)oxy]propanoates (*R*)-2a and (*S*)-2a, using trifluoromethanesulfonic anhydride and pyridine in DCM.^28^ A triflate group is an excellent leaving group used in nucleophilic substitution reactions and has been shown to be significantly superior to other leaving groups in the Walden inversion, where the inversion of a stereogenic centre in a chiral molecule takes place.^29,30^ It is known that the reaction of enantiopure α-halocarboxylic acid esters with amines is accompanied by extensive racemization of α-amino esters; with α-methanesulfonyloxy and α-toluenesulfonyloxy carboxylic acid derivatives, both racemization and elimination products are formed due to the drastic conditions.^31^ Therefore, triflate esters with primary and secondary amines are known to give *N*-substituted α-aminocarboxylates in the S_N_2 reaction, resulting in good chemical as well as optical yields. Another advantage of triflate esters is that they can be generated *in situ* and used subsequently without isolation.^32^ According to Effenberger *et al.* ethyl (*S*)-2-hydroxypropionate was converted to a triflate ester and then treatment of the triflate ester with *N*-benzyl-*N*-methylamine in dichloromethane at 0–20 °C supplied ethyl-*N*-benzyl-*N*-methyl-d-alaninate (yield, 96%);^33^ Nilsson *et al.* reported that methyl(*S*)-3-(benzyloxy)-2-[(trifluoromethanesulfonyl)oxy]propanoate with 1-methylpiperazine maintained at −40 °C in toluene containing DIPEA produced methyl(*R*)-3-(benzyloxy)-2-(4-methylpiperazin-1-yl)propanoates (yield, 66%; ee 96%).^34^

In our work, the reaction of chiral triflate esters (*R*)-2a or (*S*)-2a with 4-Boc-aminopiperidine in the presence of TEA in DCM at −50 °C led to the formation of the enantiomeric pure 2-[(Boc-amino)piperidinyl]propanoates, (*S*)-3a in a 84% yield, and (*R*)-3a in a 74% yield. The structural assignment of compounds (*S*)-3a and (*R*)-3a was readily deduced *via* detailed spectral data analysis. The IR spectrum of (*S*)-3a contained characteristic absorption bands, such as 1728 (C=O, ester) and 1681 (C=O, Boc) cm^−1^. The ^1^H NMR spectrum of compound (*S*)-3a revealed a characteristic resonance for the protons of the COOCH_3_ group, which appeared as a singlet at *δ* 3.68 ppm, and the methyl protons of the Boc-group, which appeared as a singlet at *δ* 1.42 ppm, whereas the methyl protons of the –CHC*H̲*_3_ moiety yielded a doublet at *δ* 1.27 (*J* = 7.1 Hz) ppm. In the ^13^С NMR spectrum of compound (*S*)-3a, the signals of the piperidine carbons С-3,5 (*δ* 33.0 ppm), C-4,6 (*δ* 47.9 ppm), and C-2 (*δ* 49.5 ppm), as well as the signals of carbonyl carbons of esters –COOCH_3_ (*δ* 173.6 ppm) and –COO(CH_3_)_3_ (*δ* 155.2 ppm), were detected and distinguished based on long-range correlation data obtained through an ^1^H–^13^C HMBC experiment. The IR spectrum and ^1^H-, ^13^C-, and ^15^N NMR spectra of compound (*R*)-3a showed that the corresponding spectral data are identical to those of compound (*S*)-3a. Furthermore, the reaction conditions for the synthesis of compounds (*S*)-3a and (*R*)-3a were applied to the synthesis of other 2-[(Boc-amino)piperidinyl]alkanoates such as (*S*)-3b,c and (*R*)-3b,c (Scheme 1). In nucleophilic substitution reactions with the chiral triflate esters (*R*)-2b,c and (*S*)-2b,c, 4-Boc-aminopiperidine afforded compounds 3b,c in yields of 48–81%. The structures of the newly synthesized chiral Boc-amino ester derivatives (*S*)-3a–c and (*R*)-3a–c were described and confirmed by IR, NMR spectroscopy, and high-resolution mass spectrometry (ESI in Fig. S4–S25†).

The synthesized compounds 3a–c exhibited optical activity. The corresponding (*S*)- and (*R*)-enantiomers rotated the plane-polarized light in equal amounts but in opposite directions; specific rotations [*α*]^20^_D_ for the solution of compounds (*S*)-3a and (*R*)-3a in methanol were −19.4° and 19.5°, respectively. Furthermore, enantiomers (*S*)-3b and (*R*)-3b, which contained an isopropyl group, afforded a [*α*]^20^_D_ = 34.4° (MeOH) and [*α*]^20^_D_ = −34.6° (MeOH), respectively. Similarly, [*α*]^20^_D_ for the solution of compounds (*S*)-3c and (*R*)-3c, which contained a 1-isobutyl group, were −19.2° (MeOH) and 19.4° (MeOH), respectively.

**Fig. 2:**
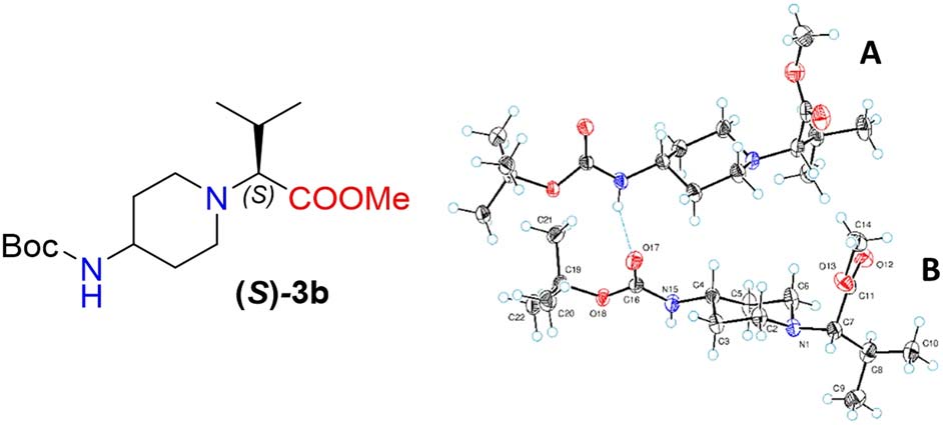
The ORTEP plot of the X-ray structure of (*S*)-3b.

**Fig. 3:**
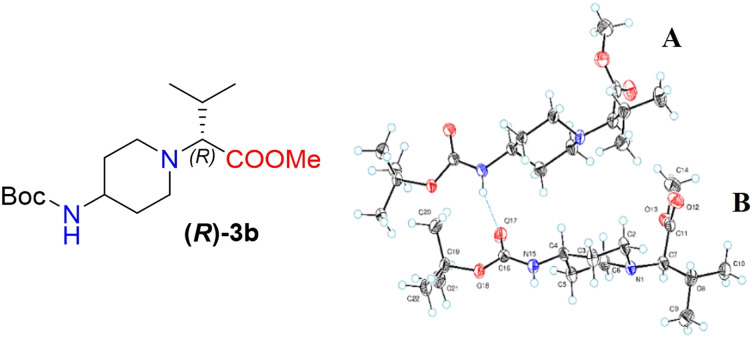
The ORTEP plot of the X-ray structure of (*R*)-3b.

Moreover, X-ray crystallography data confirmed the absolute structure of enantiomers (*S*)-3b (ref. 35) (Fig. 2) and (*R*)-3b (ref. 36) (Fig. 3). A single crystal of compounds (*S*)-3b and (*R*)-3b was prepared from acetonitrile for X-ray diffraction analysis. The asymmetric unit of (*R*)-3b consists of two rotameric forms (A and B) (Fig. 3). Both rotamers represent 1,4-*trans*-disubstituted piperidine in chair conformation, and the substituents are in equatorial positions. The (*R*)-valinate fragment is orientated so that methoxycarbonylic fragment is directed parallel to the axial C–H bonds of the piperidine ring. One of the methyls from valine is orientated anti-parallel to the methoxycarbonylic fragment.

Owing to possible rotation about the C–C(O) bond, methoxycarbonylic fragments in the A and B rotamers are orientated differently: the dihedral angle O=C–C–H in rotamer A is 14.80°, and the same angle in rotamer B is 169.02°. N–H and C=O bonds from the *tert*-butoxycarbonylamino fragment of molecule (*R*)-3b are in *E* conformation, and they are orientated parallel to the axial C–H bonds of the piperidine ring. Rotamers A and B in the asymmetric unit are both held by three hydrogen bonds (C(55)–H(55a)⋯O(17) 2.825 Å, 131.75°; N(65)–H(65)⋯O(17) 2.042 Å, 159.55°; and C(20)–H(20b)⋯O(68) 2.84 Å, 136.5°).

The crystals of (*S*)-3b are fully enantiomorphous to the crystals of (*R*)-3b (Fig. 2). This means that the crystals of (*S*)-3b and (*R*)-3b are related, like the left hand is to the right hand. For such crystals, the crystal structures are identical (the same lattice symmetry, equal cell parameters, *etc.*), except that the molecules of (*S*)-3b and (*R*)-3b are enantiomers.

We also tried to analyze the enantiomers (*S*)-3a–c and (*R*)-3a–c and their unprotected forms by chiral HPLC analysis. Various attempts were made using different enantioselective HPLC columns, but this method proved unsuccessful. Then, we proceeded to determine the enantiomeric purity of our compounds using NMR methods. Many NMR spectroscopic techniques rely on chiral auxiliaries such as chiral derivatization agents, chiral lanthanide shift reagents, metal complexes, and chiral solvating agents.^37,38^ Fuertes *et al.* developed a simple chiral derivatization protocol for the enantiopure determination of chiral primary amines using ^1^H NMR spectroscopic analysis. The method involves the condensation of amines with 2-formylphenylboronic acid (2-FPBA) and (*S*)-1,1′-bi-2-naphthol ((*S*)-BINOL). This method allows a mixture of diastereomeric derivatives to be obtained, the ratio of which can be determined by integrating the resonances in their ^1^H NMR spectra, which makes it easy to determine the enantiopurity of the starting amine.^39^

**Scheme 2:**
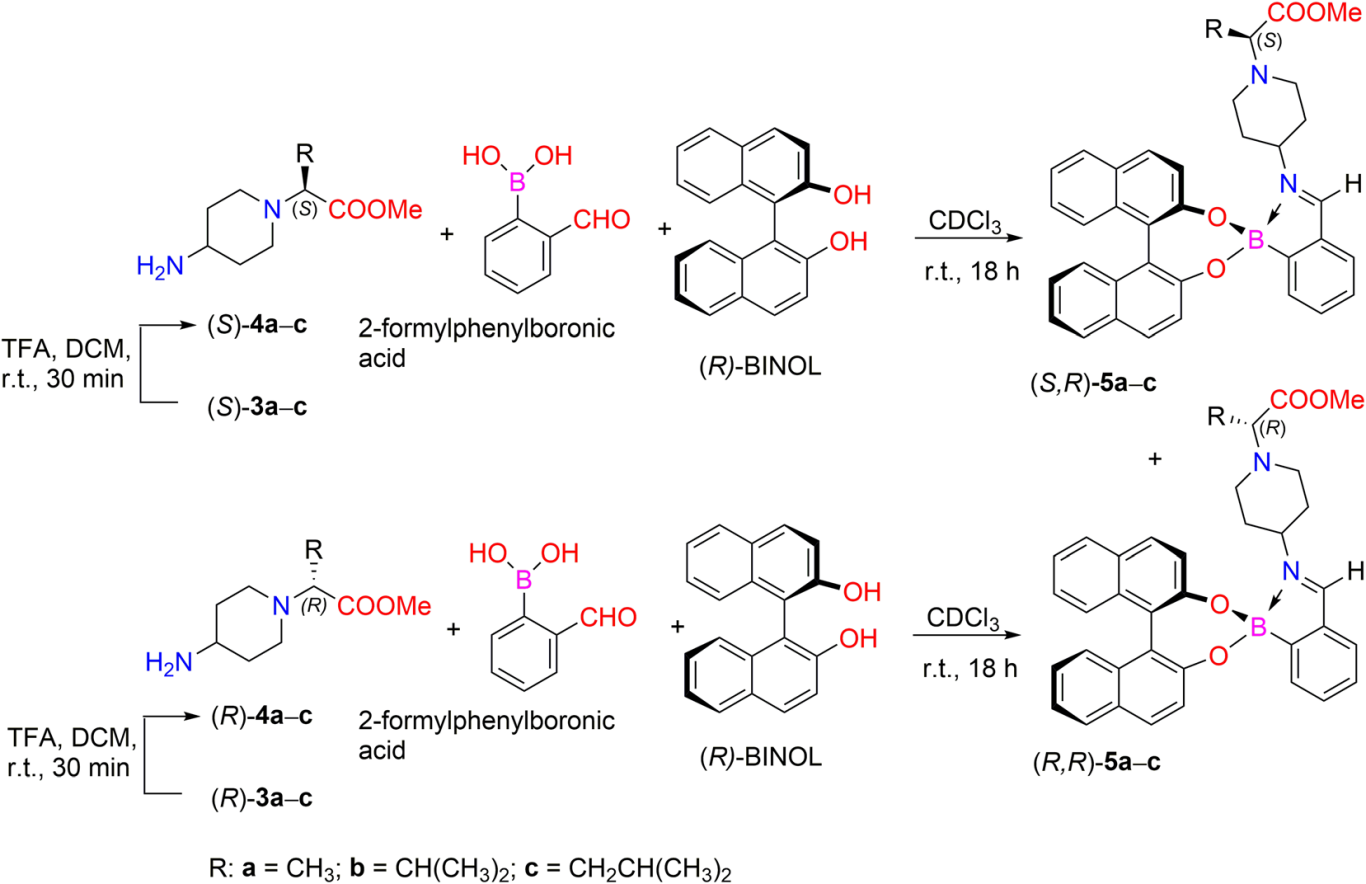
Three-component assembly for the determination of ee for enantiomers (*S*)- and (*R*)-4a–c.

In our case, the synthetic strategy to determine enantiomeric excess (ee) for amines containing remote stereogenic centers is based on the formation of iminoboronate ester complexes (Scheme 2). Deprotection of the *N*-Boc group from compounds 3a–c was carried out in the presence of TFA, followed by base workup using Cs_2_CO_3_ in order to generate free primary amines 4a–c. Furthermore, the reaction of chiral primary amines (*S*)-4a–c and (*R*)-4a–c with 2-FPBA and stereodefined (*R*)-BINOL in CDCl_3_ with 4 Å molecular sieves for 18 h at room temperature afforded a mixture of diastereomeric iminoboronate ester complexes (*S*,*R*)-5a–c and (*R*,*R*)-6a–c. The diastereomeric ratio (dr) was determined by comparing the integration ratios of distinct protons in their ^1^H NMR spectra, thus allowing indirect determination of the enantiopurity of their parent amines 4a–c (Table 1).

**Table 1:** Determination of enantiopurity for compounds (*S*,*R*)-5a–c, (*R*,*R*)-5a–c and 4a–c in ^1^H NMR spectra from the reaction of amines 4a–c with 2-FPBA and (*R*)-BINOL

Entry	Compound	dr^a^	ǀΔ*δ*ǀ^b^ (ppm)	Compound	ee^e^ (%)
1	(*S*,*R*)-5a	100 : 0	0.03^c^	(*S*)-4a	100
2	(*R*,*R*)-5a	100 : 0	0.03^c^	(*R*)-4a	100
3	(*S*,*R*)-5b	96 : 4	0.02^d^	(*S*)-4b	92
4	(*R*,*R*)-5b	94 : 6	0.02^d^	(*R*)-4b	88
5	(*S*,*R*)-5c	93 : 7	0.01^d^	(*S*)-4c	86
6	(*R*,*R*)-5c	100 : 0	0.01^d^	(*R*)-4c	100

a dr of pairs of iminoboronate esters 5a–c was determined by ^1^H NMR (700 MHz, CDCl_3_) spectroscopic analysis from crude sample.

b Chemical shift differences (Δ*δ*) of the corresponding protons a, b resonances of pairs of iminoboronate esters (*S*,*R*)-5a–c and (*R*,*R*)-5a–c in the ^1^H NMR spectra from reaction of amines 4a–c with 2-FPBA and (*R*)-BINOL.

c –CH̲CH_3_.

d –COOCH_3_.

e Determined by ^1^H NMR spectroscopic analysis of the iminoboronate esters (*S*,*R*)-5a–c and (*R*,*R*)-5a–c.

**Fig. 4:**
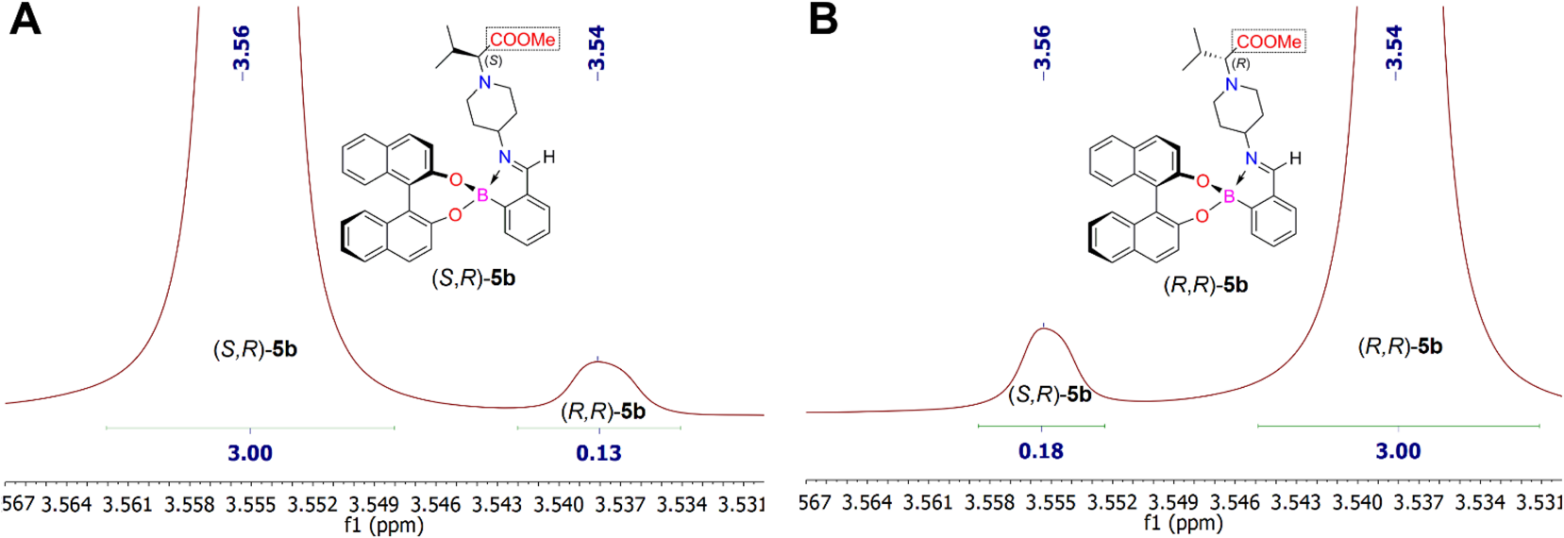
Fragments of ^1^H NMR spectra of synthesized diastereomeric iminoboronate ester complexes (*S*,*R*)-5b (part A) and (*R*,*R*)-5b (part B).

Analysis of the ^1^H NMR spectra of the iminoboronate ester complexes (*S*,*R*)-5b and (*R*,*R*)-5b revealed a characteristic resonance of the methyl protons of the esteric group (COOCH_3_), which appeared in their ^1^H NMR spectra as a singlet at *δ* 3.56 ppm and *δ* 3.54 ppm (chemical shift difference, Δ*δ*, between methyl protons of COOCH_3_ is 0.02 ppm), respectively (Fig. 4). NMR analysis showed that the dr of the iminoboronate ester complex (*S*,*R*)-5b was 96 : 4, whereas the dr of (*R*,*R*)-5b was 94 : 6. Furthermore, this diastereomeric ratio is expected to be in quantitative agreement with the enantiomeric ratio of chiral amines (*S*)-4b and (*R*)-4b. Thus allowing us to conclude that their ee are 92% and 88%, respectively. Analysis of the ^1^H NMR spectra of each derivatization reaction revealed the presence of at least one pair of resolved diastereomeric resonances in each case, whose integrals could be used to determine indirectly the enantiopurity of their parent amine 4. The ^1^H NMR spectrum of the pair of diastereomers (*S*,*R*)-5c and (*R*,*R*)-5c showed characteristic methyl proton resonances of the ester group (COOCH_3_) at *δ* 3.57 ppm and *δ* 3.56 ppm, respectively. In this case, the products (*S*,*R*)-5c and (*R*,*R*)-5c were obtained with 93 : 7 dr (86% ee for (*S*)-4c) and 100 : 0 dr (100% ee for (*R*)-4c), respectively. However, the ^1^H NMR spectra of the corresponding products, (*S*,*R*)-5a and (*R*,*R*)-5a, showed that the methyl ester group protons overlapped and resonated at *δ* 3.62 ppm. Therefore, the determination of diastereomers (*S*,*R*)-5a and (*R*,*R*)-5a according to the diastereomeric ratio by integration of their ^1^H NMR spectra showed the distinct resonances of the proton from the –C*H̲*CH_3_ moiety. The –C*H̲*CH_3_ fragment gave quadruplets at *δ* 3.21 (*J* = 7.0 Hz) and *δ* 3.18 (*J* = 7.0 Hz) ppm, respectively. The investigation of synthesized iminoboronate ester complexes (*S*,*R*)-5a and (*R*,*R*)-5a produced ^1^H NMR spectra with a diastereomeric ratio of 100 : 0 (100% ee for (*S*)-4a and (*R*)-4a) for both complexes.

In addition, deprotection of the *N*-Boc group from compounds 3a–c potentially had no influence on changing the enantiomeric ratio for obtained amines 4a–c. Therefore, the synthesis of enantiomers 3a–c (Scheme 1), as described above, is highly enantioselective with no or limited epimerization.

The prepared enantiomers, (*S*)-3a and (*R*)-3a, are potential synthons in the synthesis of small peptides. Synthetic small peptides, including heterocyclic dipeptides, are thus attractive agents and targets for therapies and diagnostics.^40–42^ For example, Pavadai *et al.* reported synthesis of a piperidin-4-one derivative containing dipeptide as an acetyl cholinesterase and β-secretase inhibitor;^43^ Blaszczyk *et al.* synthesized new piperidine dipeptides exhibiting arginase inhibition, with high intracellular activity that could be of use in the treatment of cancer.^44,45^

**Scheme 3:**
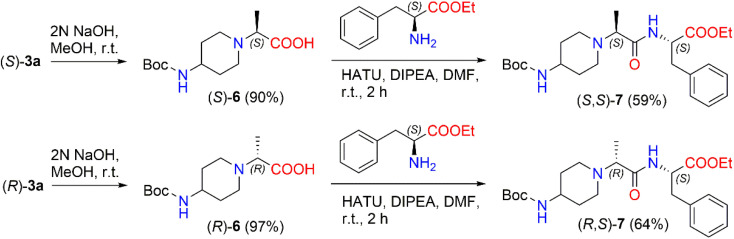
Synthesis of diastereomeric *N*-Boc-dipeptides (*S*,*S*)-7 and (*R*,*S*)-7.

In the present work, we prepared 2-(Boc-amino)piperidine dipeptides (*S*,*S*)-7 and (*R*,*S*)-7 by coupling enantiomers (*S*)-3a and (*R*)-3a with l-phenylalanine (Scheme 3). First, the ester (*S*)-3a was hydrolyzed with 2 N NaOH in methanol to afford acid (*S*)-6 in a yield of 90%. After that, the reaction of 2-[(Boc-amino)piperidinyl]propionic acid (*S*)-6 with 1-[bis(dimethylamino)methylene]-1*H*-1,2,3-triazolo[4,5-*b*]pyridinium-3-oxid hexafluorophosphate (HATU) was carried out in the presence of DIPEA in a polar aprotic solvent, such as DMF, at room temperature to form the corresponding active ester. HATU has proven to be a highly reactive peptide coupling reagent, free from by-product formation and product racemization compared to other commonly used coupling reagents.^46^ In our case, the corresponding active ester was coupled with l-phenylalanine ethyl ester hydrochloride to produce *N*-Boc-dipeptide (*S*,*S*)-7 in a 59% yield. The same method was used to synthesize *N*-Boc-dipeptide (*R*,*S*)-7 from acid (*R*)-6 with l-phenylalanine. The formation of *N*-Boc-dipeptides (*S*,*S*)-7 and (*R*,*S*)-7 was established by NMR analysis. The reaction produced (*S*,*S*)-7 in a diastereomeric ratio of 94 : 6 and (*R*,*S*)-7 in a diastereomeric ratio of 90 : 10 (ESI in Fig. S38–S43†). The ^1^H NMR spectrum of (*S*,*S*)-7 revealed a quadruplet of –C*H̲*CH_3_ proton at *δ* 3.01 (*J* = 7.0 Hz) ppm and the signals of the –CHC*H̲*_2_Ph protons split to multiplets and detected at *δ* 3.05–3.10 ppm and *δ* 3.17–3.22 ppm. In the case of the ^1^H NMR spectrum of (*R*,*S*)-7, a quadruplet of –C*H̲*CH_3_ proton was registered at *δ* 3.06 (*J* = 7.1 Hz) ppm, whereas the signals of –CHC*H̲*_2_Ph protons were observed as a doublet at *δ* 3.13 (*J* = 6.1 Hz) ppm.

**Scheme 4:**
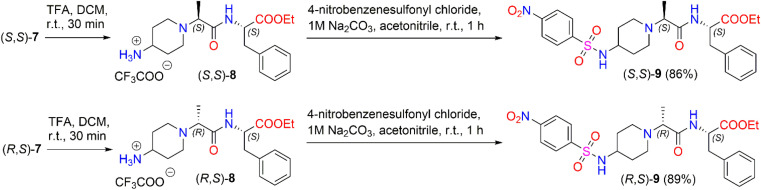
Synthesis of diastereomeric *N*-nosyl-dipeptides (*S*,*S*)-9 and (*R*,*S*)-9.

We also investigated the transformation of *N*-Boc-dipeptides (*S*,*S*)-7 and (*R*,*S*)-7 to *N*-nosyl-dipeptides (*S*,*S*)-9 and (*R*,*S*)-9 (Scheme 4). The use of a *p*-nitrobenzenesulfonyl (nosyl) group to protect the amino functional group is of great importance for obtaining *N*-methylated amino acids and peptides.^47^ Moreover, sulfonamides play a significant role in medicine as antibiotics, antithyroid agents, and antitumor drugs.^48,49^ For instance, Murthy *et al.* reported a series of novel benzhydryl piperazine-coupled nitrobenzenesulfonamide hybrids as agents which showed excellent anti-tuberculosis activity,^50^ and Ugwuja *et al.* developed and synthesized new peptide-derived antimalaria and antimicrobial agents bearing a sulfonamide moiety.^51^

To remove the protecting Boc-group,^52^ dipeptide (*S*,*S*)-7 was dissolved in DCM and then TFA was added under stirring at room temperature for 30 min. After the removal of the solvent *in vacuo*, the corresponding trifluoroacetate (*S*,*S*)-8 was used directly in the next step without further purification. The deblocked product, (*S*,*S*)-8, was coupled with 4-nitrobenzenesulfonyl chloride in the presence of 1 M Na_2_CO_3_ in acetonitrile to obtain the corresponding diastereomeric N-nosyl-dipeptide (*S*,*S*)-9 in a yield of 86%. The same reaction conditions as above were applied to synthesize N-nosyl-dipeptide (*R*,*S*)-9 in a yield of 89%. ^1^H NMR spectroscopy of the products confirmed the structure of the corresponding *N*-nosyl-dipeptides, (*S*,*S*)-9 and (*R*,*S*)-9; in particular, the ^1^H NMR spectrum showed a doublet signal of –CHC*H̲*_3_ protons at *δ* 1.08 (*J* = 7.0 Hz) ppm and *δ* 1.12 (*J* = 7.0 Hz) ppm for (*S*,*S*)-9 and (*R*,*S*)-9, respectively. The reaction gave the corresponding product, (*S*,*S*)-9, in a dr of 94 : 6, whereas product (*R*,*S*)-9 was obtained in a dr of 90 : 10 (ESI in Fig. S44–S53†).

**Scheme 5:**
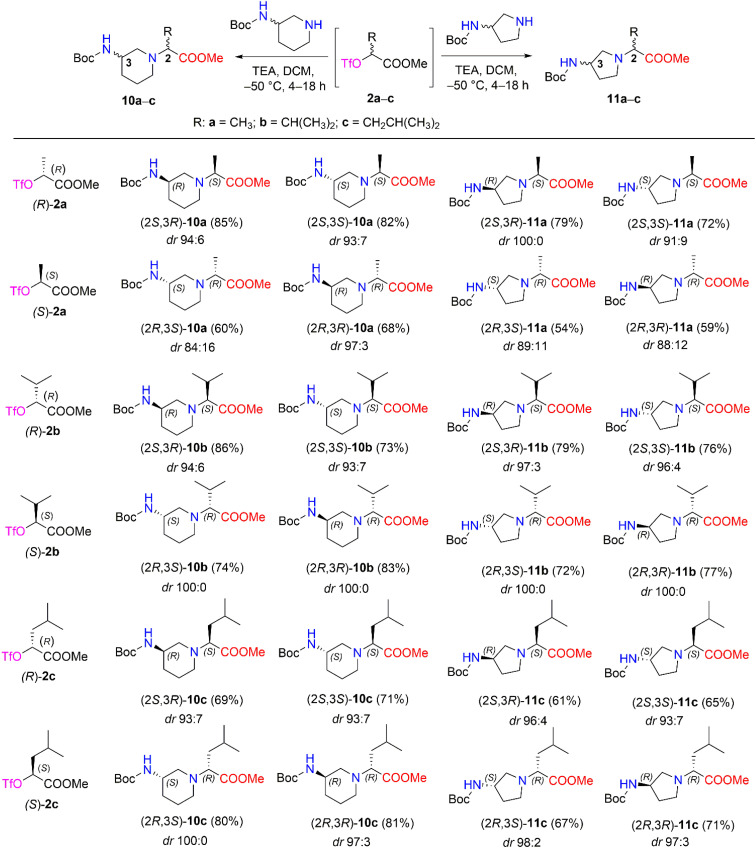
Diastereomers 10a–c and 11a–c synthesized from chiral triflate esters (*S*)- and (*R*)-2a–c. *Yields are the overall yields of two steps.

We then performed a nucleophilic substitution reaction with triflate esters (*R*)-2a–c and (*S*)-2a–c, chiral 3-Boc-aminopiperidine and 3-Boc-aminopyrrolidine, to obtain diastereomers 10a–c and 11a–c, respectively (Scheme 5). Optimization of the nucleophilic substitution conditions was undertaken for determination of diastereomeric selectivity, choosing triflate esters (*R*)-2b and (*S*)-2b as enantiomeric pair. Then, nucleophilic substitution with (*R*)-3-Boc-aminopiperidine was carried out at different temperatures, such as room temperature, −30 °C, and −50 °C, and the ^1^H NMR spectral data of the crude samples of products (2*S*,3*R*)-10b and (2*R*,3*R*)-10b were analyzed (Table 2). The ^1^H NMR spectra of (2*S*,3*R*)-10b and (2*R*,3*R*)-10b revealed characteristic resonance for the doublet signal of the proton of –C*H̲*CH(CH_3_)_2_ at *δ* 2.67 (*J* = 10.8 Hz) ppm and *δ* 2.71 (*J* = 10.8 Hz) ppm, respectively. In our study, the poorest stereoselectivity was observed when the reaction mixture was stirred at room temperature − 75 : 25 dr for (2*S*,3*R*)-10b and 78 : 22 dr for (2*R*,3*R*)-10b. Furthermore, when the reaction was carried out at −30 °C, the resulting diastereomeric ratios were 87 : 13 dr and 93 : 7 dr, respectively, for (2*S*,3*R*)-10b and (2*R*,3*R*)-10b. Moreover, carrying out the reaction at −50 °C yielded a high stereoselectivity (for (2*S*,3*R*)-10b, it was 94 : 6 dr, and for (2*R*,3*R*)-10b, it was 100 : 0 dr) and a good yield (for (2*S*,3*R*)-10b, yield was 86%, and for (2*R*,3*R*)-10b yield was 83%).

**Table 2:** Effect of temperature on the selectivity of (2*S*,3*R*)-10b and (2*R*,3*R*)-10b

Entry	Temperature	*t* (h)	dr of (2*S*,3*R*)-10b^a^	dr of (2*R*,3*R*)-10b^a^
1	r.t.	18	75 : 25	78 : 22
2	−30 °C	18	87 : 13	93 : 7
3	−50 °C	18	94 : 6	100 : 0

a Ratio was determined by ^1^H NMR spectral data from crude sample.

The obtained optimal conditions for the stereoselective synthesis of (2*S*,3*R*)-10b and (2*R*,3*R*)-10b were applied to the synthesis of other diastereomers, 10a–c and 11a–c (Scheme 5). 3-Boc-aminopiperidine in nucleophilic substitution reactions with chiral triflate esters (*R*)-2a–c and (*S*)-2a–c afforded diastereomers 10a–c in yields of 60–86%. Moreover, the synthetic conditions were used to carry out the corresponding reactions with another cyclic amine, such as 3-Boc-aminopyrrolidine. As a result, diastereomers 11a–c were obtained in 54–79% yields. The dr of aminopiperidine and aminopyrrolidine derivatives 10a–c (84 : 16 ≤ dr ≤ 100 : 0) and 11a–c (88 : 12 ≤ dr ≤ 100 : 0) were determined by their ^1^H NMR spectra after purification with column chromatography (Scheme 5) (ESI in Fig. S54–S134†).

## Materials and methods

### General information

All starting materials were purchased from commercial suppliers and were used as received. (*R*)-1,1′-Bi-2-naphthol ((*R*)-BINOL), 99% (ee: 99%); (+)-methyl-d-lactate, 98% (ee: 96%); and (−)-methyl *L*-lactate, 98% (ee: 97%) were purchased from Sigma-Aldrich. (*R*)-2-Hydroxy-3-methylbutyric acid, 97%; (*S*)-2-hydroxy-3-methylbutyric acid, 98%; (*R*)-2-hydroxy-4-methylpentanoic acid, 97%; and (*S*)-2-hydroxy-4-methylpentanoic acid, 97% were purchased from Combi-Blocks. Methylation of (*R*)- and (*S*)-hydroxy acids was carried out with dimethyl sulfate in the presence of potassium carbonate in acetone according to the described method.^53^ Flash column chromatography was performed on silica gel 60 Å (230–400 μm, Merck KGaA, Darmstadt, Germany). Thin-layer chromatography was carried out on silica gel plates (Merck Kieselgel 60 F_254_) and visualized by UV light (254 nm). Melting points were determined on a Büchi M-565 melting point apparatus and were uncorrected. The IR spectra were recorded on a Bruker Vertex 70v FT-IR spectrometer (Bruker Optik GmbH, Ettlingen, Germany) using neat samples and are reported in the frequency of absorption (cm^−1^). Mass spectra were obtained on a Shimadzu LCMS-2020 (ESI^+^) spectrometer (Shimadzu Corporation, Kyoto, Japan). High-resolution mass spectra were measured on a Bruker MicrOTOF-Q III (ESI^+^), or on a Bruker maXis 4G (ESI^+^) spectrometer (Bruker Daltonik GmbH, Bremen, Germany). Optical rotation data were recorded on a UniPol L SCHMIDT + HAENSCH polarimeter (concentration of compound (g/100 mL) was included in calculations automatically (Windaus-Labortechnik GmbH & Co. KG, Clausthal-Zellerfeld, Germany)). HPLC analysis was carried out on a Shimadzu LC-2030C apparatus with CHIRAL ART Amylose-SA (100 × 4.6 mm I.D.; S-3 μm; chiral selector amylose tris(3,5-dimethylphenylcarbamate)) and CHIRAL ART Cellulose-SB (100 × 4.6 mm I.D.; S-3 μm; chiral selector cellulose tris(3,5-dimethylphenylcarbamate)) (YMC, Shimadzu USA Manufacturing, Inc., Canby, OR, USA). Single crystals were investigated on a Rigaku, XtaLAB Synergy, Dualflex, HyPix diffractometer. The crystals were kept at 150.0(1) K during data collection. Using Olex2, the structure was solved with the ShelXT structure solution program using intrinsic phasing and refined with the olex2.refine refinement package using Gauss–Newton minimisation. The ^1^H-, ^13^C-, and ^15^N-NMR spectra were recorded in CDCl_3_ solutions at 25 °C on a Bruker Avance III 700 (700 MHz for ^1^H, 176 MHz for ^13^C, 71 MHz for ^15^N, Bruker BioSpin AG, Fallanden, Switzerland) spectrometer equipped with a 5 mm TCI ^1^H–^13^C/^15^N/D z-gradient cryoprobe. The chemical shifts (*δ*), expressed in ppm, were relative to tetramethylsilane (TMS). The ^15^N-NMR spectra were referenced to neat, external nitromethane (coaxial capillary). Full and unambiguous assignment of the ^1^H-, ^13^C-, and ^15^N-NMR resonances was achieved using a combination of standard NMR spectroscopic techniques such as DEPT, COSY, gs-HSQC, and gs-HMBC experiments. NMR spectra and HRMS data for all new compounds are provided in the ESI.†

### Methylation of (*R*)- and (*S*)-hydroxy acids (1b, 1c)

Me_2_SO_4_ (1.5 equiv.) was added to a solution of the corresponding chiral hydroxy acid (2 g) and K_2_CO_3_ (3 equiv.) in acetone (0.3 M). The reaction mixture was stirred under reflux for 5 hours. After removal of the solvent *in vacuo*, the residue was dissolved in water (15 mL) and washed with EtOAc (2 × 15 mL) and brine (15 mL). The extracts were combined and dried over sodium sulfate, filtered, and concentrated. The crude product was purified by flash chromatography (SiO_2_, eluent : acetone/*n*-hexane, 1 : 5, v/v) to produce the desired compounds (1b–c).

#### Methyl(2*R*)-2-hydroxy-3-methylbutanoate ((*R*)-1b)

Transparent oil, yield 1.746 mg (78%), [*α*]^20^_D_ = −5.1° (*c* 0.91, MeOH). ^1^H-NMR (700 MHz, CDCl_3_): *δ* 0.85 (d, *J* = 6.9 Hz, 3H, CHCH(C*H̲*_3_)_2_), 1.01 (d, *J* = 6.9 Hz, 3H, CHCH(C*H̲*_3_)_2_), 2.02–2.09 (m, 1H, CHC*H̲*(CH_3_)_2_), 2.57 (s, 1H, OH), 3.78 (s, 3H, OCH_3_), 4.04 (d, *J* = 3.6 Hz, 1H, C*H̲*CH(CH_3_)_2_). ^13^C-NMR (176 MHz, CDCl_3_): *δ* 16.1 (CHCH(*C̲*H_3_)_2_), 18.9 (CHCH(*C̲*H_3_)_2_), 32.3 (CH*C̲*H(CH_3_)_2_), 52.5 (OCH_3_), 75.2 (*C̲*HCH(CH_3_)_2_), 175.5 (*C̲*OOCH_3_). IR (FT-IR, *ν*_max_, cm^−1^): 3464 (O–H), 2967, 1729 (C=O), 1027. HRMS (ESI^+^) for C_6_H_12_NaO_3_ ([M + Na]^+^) calcd 155.0679, found 155.0680.

#### Methyl(2*S*)-2-hydroxy-3-methylbutanoate ((*S*)-1b)

Transparent oil, yield 1.590 g (71%), [*α*]^20^_D_ = 5.3° (*c* 0.82, MeOH). Spectra for this compound matched those previously reported.^54^

#### Methyl(2*R*)-2-hydroxy-4-methylpentanoate ((*R*)-1c)

Transparent oil, yield 1.526 g (69%), [*α*]^20^_D_ = −11.1° (*c* 1.32, MeOH). Spectra for this compound matched those previously reported.^55^

#### Methyl(2*S*)-2-hydroxy-4-methylpentanoate ((*S*)-1c)

Transparent oil, yield 1.393 g (63%), [*α*]^20^_D_ = 11.2° (*c* 1.26, MeOH). Spectra for this compound matched those previously reported.^56^

### Synthesis of triflates (2a–c)

A solution of the corresponding ester (1a–c) (1 equiv.) and pyridine (1.2 equiv.) in DCM (0.1 M) was cooled to 0 °C and stirred for 5 min under an argon atmosphere. Then, trifluoromethanesulfonic anhydride (1.2 equiv.) was added dropwise, and the reaction mixture was stirred for 2 hours. The resulting solution was quenched with water (10 mL), and the aqueous layer was separated and extracted with DCM (2 × 15 mL) and brine (15 mL). The organic layer was dried with anhydrous sodium sulfate, filtered, and then concentrated under reduced pressure. Crude product (2a–c) was directly used in the next step without further purification.

### Synthesis of alkanoates (3a–c, 10a–c and 11a–c)

#### Method A

Triflate (2a) (500 mg, 1 equiv.) was added to a mixture of *N*-Boc-cycloamine (1 equiv.) and TEA (1 equiv.) in DCM (15 mL) under an argon atmosphere at −50 °C, and the solution was stirred at this temperature for 4 hours. The reaction mixture was diluted with DCM (10 mL) and washed with H_2_O (2 × 15 mL) and brine (15 mL). The organic layer was dried with anhydrous sodium sulfate, filtered, and then concentrated under reduced pressure. The crude product was purified by flash chromatography.

#### Method B

Triflate (2b–c) (500 mg, 1 equiv.) was added to a mixture of *N*-Boc-cycloamine (1.5 equiv.) and TEA (1.5 equiv.) in DCM (15 mL) under an argon atmosphere at −50 °C, and the solution was stirred at r.t. overnight. The reaction mixture was diluted with DCM (10 mL) and washed with H_2_O (2 × 15 mL) and brine (15 mL). The organic layer was dried with anhydrous sodium sulfate, filtered, and then concentrated under reduced pressure. The crude product was purified by flash chromatography.

##### Methyl(*2S*)-2-{4-[(*tert*-butoxycarbonyl)amino]piperidin-1-yl}propanoate ((*S*)-3a). Method A

Compound (*R*)-2a was coupled with 4-Boc-aminopiperidine. The obtained residue was purified by column chromatography (SiO_2_, eluent : acetone/*n*-hexane, 1 : 5, v/v) to provide compound (*S*)-3a as white crystals. Yield 509 mg (84%), mp 76–78 °C, [*α*]^20^_D_ = −19.4° (*c* 0.89, MeOH). ^1^H-NMR (700 MHz, CDCl_3_): *δ* 1.27 (d, *J* = 7.1 Hz, 3H, CHC*H̲*_3_), 1.35–1.47 (m, 2H, Pip 3,5-H), 1.42 (s, 9H, C(CH_3_)_3_), 1.87–1.94 (m, 2H, Pip 3,5-H), 2.28 (td, *J* = 11.5 Hz, 2.6 Hz, 1H, Pip 6-H), 2.35 (td, *J* = 11.4 Hz, 2.6 Hz, 1H, Pip 2-H), 2.79–2.86 (m, 2H, Pip 2,6-H), 3.27 (q, *J* = 7.0 Hz, 1H, C*H̲*CH_3_), 3.39–3.50 (m, 1H, Pip 4-H), 3.68 (s, 3H, OCH_3_), 4.42 (s, 1H, NH). ^13^C-NMR (176 MHz, CDCl_3_): *δ* 15.0 (CH*C̲*H_3_), 28.5 (C(*C̲*H_3_)_3_), 33.0 (Pip 3,5-C), 47.9 (Pip 4,6-C), 49.5 (Pip 2-C), 51.5 (OCH_3_), 62.8 (*C̲*HCH_3_), 79.3 (*C̲*(CH_3_)_3_), 155.2 (*C̲*OOC(CH_3_)_3_), 173.6 (*C̲*OOCH_3_). ^15^N-NMR (71 MHz, CDCl_3_): *δ* −330.3 (Pip), −285.3 (NH). IR (FT-IR, *ν*_max_, cm^−1^): 2947, 2811, 1728 (C=O), 1681 (C=O), 1172, 1047, 885. MS *m*/*z* (%): 287 ([M + H]^+^). HRMS (ESI^+^) for C_14_H_27_N_2_O_4_ ([M + H]^+^) calcd 287. 1965, found 287.1968.

##### Methyl(2*R*)-2-{4-[(*tert*-butoxycarbonyl)amino]piperidin-1-yl}propanoate ((*R*)-3a). Method A

Compound (*S*)-2a was coupled with 4-Boc-aminopiperidine. The obtained residue was purified by column chromatography (SiO_2_, eluent : acetone/*n*-hexane, 1 : 7, v/v) to provide compound (*R*)-3a as yellowish crystals. Yield 449 mg (74%), mp 76–78 °C, [*α*]^20^_D_ = 19.5° (*c* 0.86, MeOH). ^1^H-NMR (700 MHz, CDCl_3_): *δ* 1.27 (d, *J* = 7.1 Hz, 3H, CHC*H̲*_3_), 1.36–1.46 (m, 2H, Pip 3,5-H), 1.42 (s, 9H, C(CH_3_)_3_), 1.88–1.94 (m, 2H, Pip 3,5-H), 2.28 (td, *J* = 11.5 Hz, 2.6 Hz, 1H, Pip 6-H), 2.35 (td, *J* = 11.4 Hz, 2.6 Hz, 1H, Pip 2-H), 2.79–2.86 (m, 2H, Pip 2,6-H), 3.28 (q, *J* = 7.0 Hz, 1H, C*H̲*CH_3_), 3.40–3.50 (m, 1H, Pip 4-H), 3.68 (s, 3H, OCH_3_), 4.43 (s, 1H, NH). ^13^C-NMR (176 MHz, CDCl_3_): *δ* 15.0 (CH*C̲*H_3_), 28.5 (C(*C̲*H_3_)_3_), 33.0 (Pip 3,5-C), 47.9 (Pip 4,6-C), 49.5 (Pip 2-C), 51.5 (OCH_3_), 62.8 (*C̲*HCH_3_), 79.3 (*C̲*(CH_3_)_3_), 155.2 (*C̲*OOC(CH_3_)_3_), 173.6 (*C̲*OOCH_3_). ^15^N-NMR (71 MHz, CDCl_3_): *δ* −330.3 (Pip), −285.3 (NH). IR (FT-IR, *ν*_max_, cm^−1^): 2946, 2811, 1728 (C=O), 1680 (C=O), 1170, 1046, 884. MS *m*/*z* (%): 287 ([M + H]^+^). HRMS (ESI^+^) for C_14_H_27_N_2_O_4_ ([M + H]^+^) calcd 287.1965, found 287.1967.

##### Methyl(2*S*)-2-{4-[(*tert*-butoxycarbonyl)amino]piperidin-1-yl}-3-methylbutanoate ((*S*)-3b). Method B

Compound (*R*)-2b was coupled with 4-Boc-aminopiperidine. The obtained residue was purified by column chromatography (SiO_2_, eluent: EtOAc/*n*-hexane, 1 : 5, v/v) to provide compound (*S*)-3b as white crystals. Yield 482 mg (81%), mp 97–98 °C, [*α*]^20^_D_ = 34.4° (*c* 0.91, MeOH). ^1^H-NMR (700 MHz, CDCl_3_): *δ* 0.84 (d, *J* = 6.6 Hz, 3H, CHCH(C*H̲*_3_)_2_), 0.93 (d, *J* = 6.6 Hz, 3H, CHCH(C*H̲*_3_)_2_), 1.27–1.33 (m, 1H, Pip 3-H), 1.36–1.47 (m, 1H, Pip 5-H), 1.42 (s, 9H, C(CH_3_)_3_), 1.85–1.92 (m, 2H, Pip 3,5-H), 1.98–2.04 (m, 1H, CHC*H̲*(CH_3_)_2_), 2.15–2.21 (m, 1H, Pip 6-H), 2.32–2.39 (m, 1H, Pip 2-H), 2.66–2.73 (m, 1H, Pip 6-H), 2.71 (d, *J* = 10.5 Hz, 1H, C*H̲*CH(CH_3_)_2_), 2.76–2.81 (m, 1H, Pip 2-H), 3.35–3.48 (m, 1H, Pip 4-H), 3.67 (s, 3H, OCH_3_), 4.42 (s, 1H, NH). ^13^C-NMR (176 MHz, CDCl_3_): *δ* 19.4 (CHCH(*C̲*H_3_)_2_), 19.8 (CHCH(*C̲*H_3_)_2_), 27.0 (CH*C̲*H(CH_3_)_2_), 28.5 (C(*C̲*H_3_)_3_), 33.2 (Pip 5-C), 33.4 (Pip 3-C), 45.8 (Pip 6-C), 48.1 (Pip 4-C), 50.7 (OCH_3_), 51.1 (Pip 2-C), 74.5 (*C̲*HCH(CH_3_)_3_), 79.3 (*C̲*(CH_3_)_3_), 155.2 (*C̲*OOC(CH_3_)_3_), 172.1 (*C̲*OOCH_3_). IR (FT-IR, *ν*_max_, cm^−1^): 2967, 2824, 1726 (C=O), 1680 (C=O), 1163, 1006, 773. MS *m*/*z* (%): 315 ([M + H]^+^). HRMS (ESI^+^) for C_16_H_31_N_2_O_4_ ([M + H]^+^) calcd 315.2278, found 315.2281.

##### Methyl(2*R*)-2-{4-[(*tert*-butoxycarbonyl)amino]piperidin-1-yl}-3-methylbutanoate ((*R*)-3b). Method B

Compound (*S*)-2b was coupled with 4-Boc-aminopiperidine. The obtained residue was purified by column chromatography (SiO_2_, eluent: EtOAc/*n*-hexane, 1 : 5, v/v) to provide compound (*R*)-3b as white crystals. Yield 470 mg (79%), mp 98–100 °C, [*α*]^20^_D_ = −34.6° (*c* 1.29, MeOH). ^1^H-NMR (700 MHz, CDCl_3_): *δ* 0.84 (d, *J* = 6.5 Hz, 3H, CHCH(C*H̲*_3_)_2_), 0.93 (d, *J* = 6.7 Hz, 3H, CHCH(C*H̲*_3_)_2_), 1.26–1.34 (m, 1H, Pip 3-H), 1.38–1.46 (m, 1H, Pip 5-H), 1.42 (s, 9H, C(CH_3_)_3_), 1.84–1.91 (m, 2H, Pip 3,5-H), 1.97–2.04 (m, 1H, CHC*H̲*(CH_3_)_2_), 2.15–2.22 (m, 1H, Pip 6-H), 2.32–2.40 (m, 1H, Pip 2-H), 2.66–2.74 (m, 1H, Pip 6-H), 2.71 (d, *J* = 10.5 Hz, 1H, C*H̲*CH(CH_3_)_2_), 2.76–2.81 (m, 1H, Pip 2-H), 3.34–3.50 (m, 1H, Pip 4-H), 3.67 (s, 3H, OCH_3_), 4.42 (s, 1H, NH). ^13^C-NMR (176 MHz, CDCl_3_): *δ* 19.4 (CHCH(*C̲*H_3_)_2_), 19.8 (CHCH(*C̲*H_3_)_2_), 27.0 (CH*C̲*H(CH_3_)_2_), 28.5 (C(*C̲*H_3_)_3_), 33.2 (Pip 5-C), 33.4 (Pip 3-C), 45.9 (Pip 6-C), 48.1 (Pip 4-C), 50.7 (OCH_3_), 51.1 (Pip 2-C), 74.5 (*C̲*HCH(CH_3_)_3_), 79.3 (*C̲*(CH_3_)_3_), 155.2 (*C̲*OOC(CH_3_)_3_), 172.1 (*C̲*OOCH_3_). IR (FT-IR, *ν*_max_, cm^−1^): 2967, 2824, 1727 (C=O), 1680 (C=O), 1163, 1006, 773. MS *m*/*z* (%): 315 ([M + H]^+^). HRMS (ESI^+^) for C_16_H_31_N_2_O_4_ ([M + H]^+^) calcd 315.2278, found 315.2279.

##### Methyl(2*S*)-2-{4-[(*tert*-butoxycarbonyl)amino]piperidin-1-yl}-4-methylpentanoate ((*S*)-3c). Method B

Compound (*R*)-2c was coupled with 4-Boc-aminopiperidine. The obtained residue was purified by column chromatography (SiO_2_, eluent: EtOAc/*n*-hexane, 1 : 9, v/v) to provide compound (*S*)-3c as white crystals. Yield 283 mg (48%), mp 77–79 °C, [*α*]^20^_D_ = −19.2° (*c* 0.98, MeOH). ^1^H-NMR (700 MHz, CDCl_3_): *δ* 0.87 (d, *J* = 6.1 Hz, 3H, CHCH_2_CH(C*H̲*_3_)_2_), 0.90 (d, *J* = 6.2 Hz, 3H, CHCH_2_CH(C*H̲*_3_)_2_), 1.27–1.35 (m, 1H, CHC*H̲*_2_CH(CH_3_)_2_), 1.35–1.53 (m, 2H, Pip 3,5-H), 1.42 (s, 9H, C(CH_3_)_3_), 1.54–1.61 (m, 2H, CHCH_2_C*H̲*(CH_3_)_2_ and CHC*H̲*_2_CH(CH_3_)_2_), 1.84–1.94 (m, 2H, Pip 3,5-H), 2.25–2.30 (m, 1H, Pip 6-H), 2.39–2.46 (m, 1H, Pip 2-H), 2.72–2.86 (m, 2H, Pip 2,6-H), 3.21–3.28 (m, 1H, C*H̲*CH_2_CH(CH_3_)_2_), 3.38–3.50 (m, 1H, Pip 4-H), 3.67 (s, 3H, OCH_3_), 4.42 (s, 1H, NH). ^13^C-NMR (176 MHz, CDCl_3_): *δ* 22.7 (CHCH_2_CH(*C̲*H_3_)_2_), 25.2 (CHCH_2_*C̲*H(CH_3_)_2_), 28.5 (C(*C̲*H_3_)_3_), 33.1 (Pip 5-C), 33.3 (Pip 3-C), 38.5 (CH*C̲*H_2_CH(CH_3_)_2_), 46.4 (Pip 6-C), 48.0 (Pip 4-C), 50.6 (Pip 2-C), 51.1 (OCH_3_), 65.7 (*C̲*HCH_2_CH(CH_3_)_2_), 79.3 (*C̲*(CH_3_)_3_), 155.2 (*C̲*OOC(CH_3_)_3_), 173.0 (*C̲*OOCH_3_). IR (FT-IR, *ν*_max_, cm^−1^): 2953, 2869, 1734 (C=O), 1677 (C=O), 1162, 1004, 750. MS *m*/*z* (%): 329 ([M + H]^+^). HRMS (ESI^+^) for C_17_H_33_N_2_O_4_ ([M + H]^+^) calcd 329.2435, found 329.2433.

##### Methyl(2*R*)-2-{4-[(*tert*-butoxycarbonyl)amino]piperidin-1-yl}-4-methylpentanoate ((*R*)-3c). Method B

Compound (*S*)-2c was coupled with 4-Boc-aminopiperidine. The obtained residue was purified by column chromatography (SiO_2_, eluent: EtOAc/*n*-hexane, 1 : 9, v/v) to provide compound (*R*)-3c as white crystals. Yield 348 mg (59%), mp 79–81 °C, [*α*]^20^_D_ = 19.4° (*c* 1.06, MeOH). ^1^H-NMR (700 MHz, CDCl_3_): *δ* 0.87 (d, *J* = 6.2 Hz, 3H, CHCH_2_CH(C*H̲*_3_)_2_), 0.90 (d, *J* = 6.3 Hz, 3H, CHCH_2_CH(C*H̲*_3_)_2_), 1.26–1.34 (m, 1H, CHC*H̲*_2_CH(CH_3_)_2_), 1.35–1.52 (m, 2H, Pip 3,5-H), 1.42 (s, 9H, C(CH_3_)_3_), 1.53–1.61 (m, 2H, CHCH_2_C*H̲*(CH_3_)_2_ and CHC*H̲*_2_CH(CH_3_)_2_), 1.85–1.92 (m, 2H, Pip 3,5-H), 2.22–2.31 (m, 1H, Pip 6-H), 2.38–2.47 (m, 1H, Pip 2-H), 2.73–2.85 (m, 2H, Pip 2,6-H), 3.20–3.28 (m, 1H, C*H̲*CH_2_CH(CH_3_)_2_), 3.38–3.47 (m, 1H, Pip 4-H), 3.66 (s, 3H, OCH_3_), 4.42 (s, 1H, NH). ^13^C-NMR (176 MHz, CDCl_3_): *δ* 22.7 (CHCH_2_CH(*C̲*H_3_)_2_), 25.2 (CHCH_2_*C̲*H(CH_3_)_2_), 28.5 (C(*C̲*H_3_)_3_), 33.1 (Pip 5-C), 33.3 (Pip 3-C), 38.5 (CH*C̲*H_2_CH(CH_3_)_2_), 46.4 (Pip 6-C), 48.0 (Pip 4-C), 50.6 (Pip 2-C), 51.1 (OCH_3_), 65.7 (*C̲*HCH_2_CH(CH_3_)_2_), 79.3 (*C̲*(CH_3_)_3_), 155.2 (*C̲*OOC(CH_3_)_3_), 173.0 (*C̲*OOCH_3_). IR (FT-IR, *ν*_max_, cm^−1^): 2953, 2869, 1734 (C=O), 1678 (C=O), 1162, 1004, 750. MS *m*/*z* (%): 329 ([M + H]^+^). HRMS (ESI^+^) for C_17_H_33_N_2_O_4_ ([M + H]^+^) calcd 329.2435, found 329.2434.

##### Methyl(2*S*)-2-{(3*R*)-3-[(*tert*-butoxycarbonyl)amino]piperidin-1-yl}propanoate ((2*S*,3*R*)-10a). Method A

Compound (*R*)-2a was coupled with (*R*)-3-Boc-aminopiperidine. The obtained residue was purified by column chromatography (SiO_2_, eluent : acetone/*n*-hexane, 1 : 5, v/v) to provide compound (2*S*,3*R*)-10a as transparent oil in an 85% yield (515 mg) with a dr of 94 : 6, [*α*]^20^_D_ = 14.2° (*c* 0.77, MeOH). ^1^H-NMR (700 MHz, CDCl_3_): *δ* 1.25 (d, *J* = 7.1 Hz, 3H, CHC*H̲*_3_), 1.43 (s, 9H, C(CH_3_)_3_), 1.47–1.61 (m, 3H, Pip 4,5-H), 1.62–1.71 (m, 1H, Pip 5-H), 2.32–2.47 (m, 2H, Pip 2,6-H), 2.54–2.62 (m, 1H, Pip 6-H), 2.70–2.80 (m, 1H, Pip 2-H), 3.27–3.34 (m, 1H, C*H̲*CH_3_), 3.64–3.72 (m, 1H, Pip 3-H), 3.67 (s, 3H, OCH_3_), 5.05 (s, 1H, NH). ^13^C-NMR (176 MHz, CDCl_3_): *δ* 14.3 (CH*C̲*H_3_), 22.7 (Pip 5-C), 28.6 (C(*C̲*H_3_)_3_), 29.9 (Pip 4-C), 46.6 (Pip 3-C), 50.0 (Pip 6-C), 51.4 (OCH_3_), 54.9 (Pip 2-C), 62.5 (*C̲*HCH_3_), 79.1 (*C̲*(CH_3_)_3_), 155.3 (*C̲*OOC(CH_3_)_3_), 173.5 (*C̲*OOCH_3_). ^15^N-NMR (71 MHz, CDCl_3_): *δ* −337.5 (Pip), −290.0 (NH). IR (FT-IR, *ν*_max_, cm^−1^): 2939, 2858, 1732 (C=O), 1698 (C=O), 1160, 1050, 780. MS *m*/*z* (%): 287 ([M + H]^+^). HRMS (ESI^+^) for C_14_H_27_N_2_O_4_ ([M + H]^+^) calcd 287.1965, found 287.1968.

##### Methyl(2*R*)-2-{(3*S*)-3-[(*tert*-butoxycarbonyl)amino]piperidin-1-yl}propanoate ((2*R*,3*S*)-10a). Method A

Compound (*S*)-2a was coupled with (*S*)-3-Boc-aminopiperidine. The obtained residue was purified by column chromatography (SiO_2_, eluent : acetone/*n*-hexane, 1 : 5, v/v) to provide compound (2*R*,3*S*)-10a as yellowish oil in an 60% yield (364 mg) with a dr of 84 : 16, [*α*]^20^_D_ = −14.4° (*c* 1.00, MeOH). ^1^H-NMR (700 MHz, CDCl_3_): *δ* 1.25 (d, *J* = 7.1 Hz, 3H, CHC*H̲*_3_), 1.43 (s, 9H, C(CH_3_)_3_), 1.47–1.61 (m, 3H, Pip 4,5-H), 1.63–1.70 (m, 1H, Pip 5-H), 2.35–2.53 (m, 2H, Pip 2,6-H), 2.54–2.63 (m, 1H, Pip 6-H), 2.72–2.82 (m, 1H, Pip 2-H), 3.27–3.34 (m, 1H, C*H̲*CH_3_), 3.62–3.75 (m, 1H, Pip 3-H), 3.68 (s, 3H, OCH_3_), 5.05 (s, 1H, NH). ^13^C-NMR (176 MHz, CDCl_3_): *δ* 14.3 (CH*C̲*H_3_), 22.7 (Pip 5-C), 28.6 (C(*C̲*H_3_)_3_), 29.9 (Pip 4-C), 46.6 (Pip 3-C), 50.0 (Pip 6-C), 51.4 (OCH_3_), 54.9 (Pip 2-C), 62.5 (*C̲*HCH_3_), 79.1 (*C̲*(CH_3_)_3_), 155.3 (*C̲*OOC(CH_3_)_3_), 173.5 (*C̲*OOCH_3_). IR (FT-IR, *ν*_max_, cm^−1^): 2940, 2858, 1732 (C=O), 1699 (C=O), 1161, 1049, 780. MS *m*/*z* (%): 287 ([M + H]^+^). HRMS (ESI^+^) for C_14_H_27_N_2_O_4_ ([M + H]^+^) calcd 287.1965, found 287.1967.

##### Methyl(2*S*)-2-{(3*S*)-3-[(*tert*-butoxycarbonyl)amino]piperidin-1-yl}propanoate ((2*S*,3*S*)-10a). Method A

Compound (*R*)-2a was coupled with (*S*)-3-Boc-aminopiperidine. The obtained residue was purified by column chromatography (SiO_2_, eluent : acetone/*n*-hexane, 1 : 5, v/v) to provide compound (2*S*,3*S*)-10a as white crystals in an 82% yield (497 mg) with a dr of 93 : 7, mp 77–78 °C, [*α*]^20^_D_ = −52.8° (*c* 0.98, MeOH). ^1^H-NMR (700 MHz, CDCl_3_): *δ* 1.25 (d, *J* = 7.2 Hz, 3H, CHC*H̲*_3_), 1.43 (s, 9H, C(CH_3_)_3_), 1.46–1.53 (m, 2H, Pip 4,5-H), 1.55–1.62 (m, 1H, Pip 4-H), 1.64–1.72 (m, 1H, Pip 5-H), 2.43–2.57 (m, 3H, Pip 2,6-H), 2.62–2.70 (m, 1H, Pip 2-H), 3.29 (q, *J* = 7.1 Hz, 1H, C*H̲*CH_3_), 3.67 (s, 3H, OCH_3_), 3.69–3.75 (m, 1H, Pip 3-H), 4.96 (s, 1H, NH). ^13^C-NMR (176 MHz, CDCl_3_): *δ* 15.2 (CH*C̲*H_3_), 22.7 (Pip 5-C), 28.6 (C(*C̲*H_3_)_3_), 29.9 (Pip 4-C), 46.4 (Pip 3-C), 50.3 (Pip 6-C), 51.3 (OCH_3_), 54.6 (Pip 2-C), 62.5 (*C̲*HCH_3_), 79.1 (*C̲*(CH_3_)_3_), 155.3 (*C̲*OOC(CH_3_)_3_), 173.5 (*C̲*OOCH_3_). ^15^N-NMR (71 MHz, CDCl_3_): *δ* −336.5 (Pip), −290.2 (NH). IR (FT-IR, *ν*_max_, cm^−1^): 2942, 2844, 1736 (C=O), 1699 (C=O), 1164, 1089, 775. MS *m*/*z* (%): 287 ([M + H]^+^). HRMS (ESI^+^) for C_14_H_27_N_2_O_4_ ([M + H]^+^) calcd 287.1965, found 287.1967.

##### Methyl(2*R*)-2-{(3*R*)-3-[(*tert*-butoxycarbonyl)amino]piperidin-1-yl}propanoate ((2*R*,3*R*)-10a). Method A

Compound (*S*)-2a was coupled with (*R*)-3-Boc-aminopiperidine. The obtained residue was purified by column chromatography (SiO_2_, eluent : acetone/*n*-hexane, 1 : 5, v/v) to provide compound (2*R*,3*R*)-10a as white crystals in an 68% yield (412 mg) with a dr of 97 : 3, mp 77–78 °C, [*α*]^20^_D_ = 53.1° (*c* 0.91, MeOH). ^1^H-NMR (700 MHz, CDCl_3_): *δ* 1.25 (d, *J* = 7.1 Hz, 3H, CHC*H̲*_3_), 1.43 (s, 9H, C(CH_3_)_3_), 1.47–1.53 (m, 2H, Pip 4,5-H), 1.55–1.62 (m, 1H, Pip 4-H), 1.64–1.72 (m, 1H, Pip 5-H), 2.36–2.57 (m, 3H, Pip 2,6-H), 2.63–2.71 (m, 1H, Pip 2-H), 3.29 (q, *J* = 7.1 Hz, 1H, C*H̲*CH_3_), 3.67 (s, 3H, OCH_3_), 3.69–3.74 (m, 1H, Pip 3-H), 4.97 (s, 1H, NH). ^13^C-NMR (176 MHz, CDCl_3_): *δ* 15.1 (CH*C̲*H_3_), 22.7 (Pip 5-C), 28.6 (C(*C̲*H_3_)_3_), 29.9 (Pip 4-C), 46.4 (Pip 3-C), 50.3 (Pip 6-C), 51.3 (OCH_3_), 54.6 (Pip 2-C), 62.5 (*C̲*HCH_3_), 79.1 (*C̲*(CH_3_)_3_), 155.3 (*C̲*OOC(CH_3_)_3_), 173.5 (*C̲*OOCH_3_). ^15^N-NMR (71 MHz, CDCl_3_): *δ* −336.4 (Pip), −290.2 (NH). IR (FT-IR, *ν*_max_, cm^−1^): 2941, 2846, 1736 (C=O), 1699 (C=O), 1163, 1089, 775. MS *m*/*z* (%): 287 ([M + H]^+^). HRMS (ESI^+^) for C_14_H_27_N_2_O_4_ ([M + H]^+^) calcd 287.1965, found 287.1968.

##### Methyl(2*S*)-2-{(3*R*)-3-[(*tert*-butoxycarbonyl)amino]piperidin-1-yl}-3-methylbutanoate ((2*S*,3*R*)-10b). Method B

Compound (*R*)-2b was coupled with (*R*)-3-Boc-aminopiperidine. The obtained residue was purified by column chromatography (SiO_2_, eluent: EtOAc/*n*-hexane, 1 : 5, v/v) to provide compound (2*S*,3*R*)-10b as yellowish oil in an 86% yield (512 mg) with a dr of 94 : 6, [*α*]^20^_D_ = 19.0° (*c* 0.92, MeOH). ^1^H-NMR (700 MHz, CDCl_3_): *δ* 0.84 (d, *J* = 6.5 Hz, 3H, CHCH(C*H̲*_3_)_2_), 0.98 (d, *J* = 6.7 Hz, 3H, CHCH(C*H̲*_3_)_2_), 1.38–1.50 (m, 1H, Pip 5-H), 1.44 (m, 9H, C(CH_3_)_3_), 1.51–1.57 (m, 1H, Pip 4-H), 1.59–1.65 (m, 2H, Pip 4,5-H), 2.03–2.09 (m, 1H, CHC*H̲*(CH_3_)_2_), 2.21–2.38 (m, 2H, Pip 2,6-H), 2.49–2.58 (m, 1H, Pip 6-H), 2.67 (d, *J* = 10.8 Hz, 1H, C*H̲*CH(CH_3_)_2_), 2.73–2.80 (m, 1H, Pip 2-H), 3.61–3.72 (m, 1H, Pip 3-H), 3.67 (s, 3H, OCH_3_), 4.87 (s, 1H, NH). ^13^C-NMR (176 MHz, CDCl_3_): *δ* 19.4 (CHCH(*C̲*H_3_)_2_), 20.0 (CHCH(*C̲*H_3_)_2_), 23.0 (Pip 5-C), 27.0 (CH*C̲*H(CH_3_)_2_), 28.6 (C(*C̲*H_3_)_3_), 30.4 (Pip 4-C), 47.1 (Pip 3-C), 49.8 (Pip 6-C), 50.7 (OCH_3_), 55.8 (Pip 2-C), 74.7 (*C̲*HCH(CH_3_)_3_), 79.2 (*C̲*(CH_3_)_3_), 155.3 (*C̲*OOC(CH_3_)_3_), 172.0 (*C̲*OOCH_3_). IR (FT-IR, *ν*_max_, cm^−1^): 2937, 2811, 1729 (C=O), 1711 (C=O), 1161, 1017, 778. MS *m*/*z* (%): 315 ([M + H]^+^). HRMS (ESI^+^) for C_16_H_31_N_2_O_4_ ([M + H]^+^) calcd 315.2278, found 315.2281.

##### Methyl(2*R*)-2-{(3*S*)-3-[(*tert*-butoxycarbonyl)amino]piperidin-1-yl}-3-methylbutanoate ((2*R*,3*S*)-10b). Method B

Compound (*S*)-2b was coupled with (*S*)-3-Boc-aminopiperidine. The obtained residue was purified by column chromatography (SiO_2_, eluent: EtOAc/*n*-hexane, 1 : 5, v/v) to provide compound (2*R*,3*S*)-10b as orange oil in an 74% yield (440 mg) with a dr of 100 : 0, [*α*]^20^_D_ = −18.9° (*c* 0.96, MeOH). ^1^H-NMR (700 MHz, CDCl_3_): *δ* 0.84 (d, *J* = 6.6 Hz, 3H, CHCH(C*H̲*_3_)_2_), 0.98 (d, *J* = 6.7 Hz, 3H, CHCH(C*H̲*_3_)_2_), 1.37–1.48 (m, 1H, Pip 5-H), 1.43 (s, 9H, C(CH_3_)_3_), 1.51–1.56 (m, 1H, Pip 4-H), 1.58–1.68 (m, 2H, Pip 4,5-H), 2.02–2.08 (m, 1H, CHC*H̲*(CH_3_)_2_), 2.20–2.39 (m, 2H, Pip 2,6-H), 2.47–2.58 (m, 1H, Pip 6-H), 2.67 (d, *J* = 10.8 Hz, 1H, C*H̲*CH(CH_3_)_2_), 2.72–2.81 (m, 1H, Pip 2-H), 3.60–3.71 (m, 1H, Pip 3-H), 3.67 (s, 3H, OCH_3_), 4.86 (s, 1H, NH). ^13^C-NMR (176 MHz, CDCl_3_): *δ* 19.4 (CHCH(*C̲*H_3_)_2_), 20.0 (CHCH(*C̲*H_3_)_2_), 23.0 (Pip 5-C), 27.0 (CH*C̲*H(CH_3_)_2_), 28.6 (C(*C̲*H_3_)_3_), 30.4 (Pip 4-C), 47.1 (Pip 3-C), 49.9 (Pip 6-C), 50.7 (OCH_3_), 55.8 (Pip 2-C), 74.7 (*C̲*HCH(CH_3_)_3_), 79.2 (*C̲*(CH_3_)_3_), 155.3 (*C̲*OOC(CH_3_)_3_), 172.0 (*C̲*OOCH_3_). IR (FT-IR, *ν*_max_, cm^−1^): 2937, 2811, 1727 (C=O), 1711 (C=O), 1161, 1017, 778. MS *m*/*z* (%): 315 ([M + H]^+^). HRMS (ESI^+^) for C_16_H_31_N_2_O_4_ ([M + H]^+^) calcd 315.2278, found 315.2280.

##### Methyl(2*S*)-2-{(3*S*)-3-[(*tert*-butoxycarbonyl)amino]piperidin-1-yl}-3-methylbutanoate ((2*S*,3*S*)-10b). Method B

Compound (*R*)-2b was coupled with (*S*)-3-Boc-aminopiperidine. The obtained residue was purified by column chromatography (SiO_2_, eluent: EtOAc/*n*-hexane, 1 : 5, v/v) to provide compound (2*S*,3*S*)-10b as yellowish oil in an 73% yield (434 mg) with a dr of 93 : 7, [*α*]^20^_D_ = −90.2° (*c* 0.92, MeOH). ^1^H-NMR (700 MHz, CDCl_3_): *δ* 0.83 (d, *J* = 6.5 Hz, 3H, CHCH(C*H̲*_3_)_2_), 0.94 (d, *J* = 6.7 Hz, 3H, CHCH(C*H̲*_3_)_2_), 1.37–1.50 (m, 2H, Pip 4,5-H), 1.43 (m, 9H, C(CH_3_)_3_), 1.58–1.71 (m, 2H, Pip 4,5-H), 1.96–2.03 (m, 1H, CHC*H̲*(CH_3_)_2_), 2.28–2.51 (m, 3H, Pip 2,6-H), 2.58–2.65 (m, 1H, Pip 2-H), 2.71 (d, *J* = 10.8 Hz, 1H, C*H̲*CH(CH_3_)_2_), 3.67 (m, 3H, OCH_3_), 3.69–3.75 (m, 1H, Pip 3-H), 4.84 (s, 1H, NH). ^13^C-NMR (176 MHz, CDCl_3_): *δ* 19.3 (CHCH(*C̲*H_3_)_2_), 20.0 (CHCH(*C̲*H_3_)_2_), 23.1 (Pip 5-C), 26.8 (CH*C̲*H(CH_3_)_2_), 28.6 (C(*C̲*H_3_)_3_), 30.3 (Pip 4-C), 46.7 (Pip 3-C), 50.2 (Pip 6-C), 50.7 (OCH_3_), 55.2 (Pip 2-C), 74.8 (*C̲*HCH(CH_3_)_3_), 79.2 (*C̲*(CH_3_)_3_), 155.3 (*C̲*OOC(CH_3_)_3_), 172.1 (*C̲*OOCH_3_). IR (FT-IR, *ν*_max_, cm^−1^): 2963, 2811, 1729 (C=O), 1712 (C=O), 1160, 1001, 780. MS *m*/*z* (%): 315 ([M + H]^+^). HRMS (ESI^+^) for C_16_H_31_N_2_O_4_ ([M + H]^+^) calcd 315.2278, found 315.2280.

##### Methyl(2*R*)-2-{(3*R*)-3-[(*tert*-butoxycarbonyl)amino]piperidin-1-yl}-3-methylbutanoate ((2*R*,3*R*)-10b). Method B

Compound (*S*)-2b was coupled with (*R*)-3-Boc-aminopiperidine. The obtained residue was purified by column chromatography (SiO_2_, eluent: EtOAc/*n*-hexane, 1 : 5, v/v) to provide compound (2*R*,3*R*)-10b as yellowish oil in an 83% yield (494 mg) with a dr of 100 : 0, [*α*]^20^_D_ = 90.4° (*c* 1.03, MeOH). ^1^H-NMR (700 MHz, CDCl_3_): *δ* 0.83 (d, *J* = 6.5 Hz, 3H, CHCH(C*H̲*_3_)_2_), 0.95 (d, *J* = 6.6 Hz, 3H, CHCH(C*H̲*_3_)_2_), 1.38–1.49 (m, 2H, Pip 4,5-H), 1.43 (s, 9H, C(CH_3_)_3_), 1.61–1.72 (m, 2H, Pip 4,5-H), 1.97–2.03 (m, 1H, CHC*H̲*(CH_3_)_2_), 2.32–2.52 (m, 3H, Pip 2,6-H), 2.58–2.67 (m, 1H, Pip 2-H), 2.71 (d, *J* = 10.8 Hz, 1H, C*H̲*CH(CH_3_)_2_), 3.67 (s, 3H, OCH_3_), 3.69–3.76 (m, 1H, Pip 3-H), 4.84 (s, 1H, NH). ^13^C-NMR (176 MHz, CDCl_3_): *δ* 19.3 (CHCH(*C̲*H_3_)_2_), 20.0 (CHCH(*C̲*H_3_)_2_), 23.1 (Pip 5-C), 26.8 (CH*C̲*H(CH_3_)_2_), 28.6 (C(*C̲*H_3_)_3_), 30.3 (Pip 4-C), 46.7 (Pip 3-C), 50.3 (Pip 6-C), 50.7 (OCH_3_), 55.1 (Pip 2-C), 74.8 (*C̲*HCH(CH_3_)_3_), 79.2 (*C̲*(CH_3_)_3_), 155.3 (*C̲*OOC(CH_3_)_3_), 172.1 (*C̲*OOCH_3_). IR (FT-IR, *ν*_max_, cm^−1^): 2962, 2811, 1729 (C=O), 1712 (C=O), 1160, 1002, 780. MS *m*/*z* (%): 315 ([M + H]^+^). HRMS (ESI^+^) for C_16_H_31_N_2_O_4_ ([M + H]^+^) calcd 315.2278, found 315.2280.

##### Methyl(2*S*)-2-{(3*R*)-3-[(*tert*-butoxycarbonyl)amino]piperidin-1-yl}-4-methylpentanoate ((2*S*,3*R*)-10c). Method B

Compound (*R*)-2c was coupled with (*R*)-3-Boc-aminopiperidine. The obtained residue was purified by column chromatography (SiO_2_, eluent: EtOAc/*n*-hexane, 1 : 7, v/v) to provide compound (2*S*,3*R*)-10c as yellow oil in an 69% yield (409 mg) with a dr of 93 : 7, [*α*]^20^_D_ = 19.2° (*c* 1.51, MeOH). ^1^H-NMR (700 MHz, CDCl_3_): *δ* 0.88 (d, *J* = 6.6 Hz, 3H, CHCH_2_CH(C*H̲*_3_)_2_), 0.92 (d, *J* = 6.7 Hz, 3H, CHCH_2_CH(C*H̲*_3_)_2_), 1.41–1.46 (m, 1H, Pip 5-H), 1.43 (s, 9H, C(CH_3_)_3_), 1.50–1.58 (m, 3H, Pip 4-H, CHCH_2_C*H̲*(CH_3_)_2_, CHC*H̲*_2_CH(CH_3_)_2_), 1.59–1.67 (m, 3H, Pip 4,5-H and CHC*H̲*_2_CH(CH_3_)_2_), 2.31–2.44 (m, 2H, Pip 2,6-H), 2.54–2.62 (m, 1H, Pip 6-H), 2.78–2.86 (m, 1H, Pip 2-H), 3.22 (t, *J* = 7.7 Hz, 1H, C*H̲*CH_2_CH(CH_3_)_2_), 3.63–3.69 (m, 1H, Pip 3-H), 3.66 (s, 3H, OCH_3_), 4.91 (s, 1H, NH). ^13^C-NMR (176 MHz, CDCl_3_): *δ* 22.3 (CHCH_2_CH(*C̲*H_3_)_2_), 22.9 (Pip 5-C and CHCH_2_CH(*C̲*H_3_)_2_), 25.1 (CHCH_2_*C̲*H(CH_3_)_2_), 28.6 (C(*C̲*H_3_)_3_), 30.2 (Pip 4-C), 38.1 (CH*C̲*H_2_CH(CH_3_)_2_), 46.9 (Pip 3-C), 49.6 (Pip 6-C), 51.0 (OCH_3_), 55.7 (Pip 2-C), 65.6 (*C̲*HCH_2_CH(CH_3_)_2_), 79.1 (*C̲*(CH_3_)_3_), 155.3 (*C̲*OOC(CH_3_)_3_), 173.0 (*C̲*OOCH_3_). IR (FT-IR, *ν*_max_, cm^−1^): 2953, 2867, 1721 (C=O), 1711 (C=O), 1159, 1008, 778. MS *m*/*z* (%): 329 ([M + H]^+^). HRMS (ESI^+^) for C_17_H_33_N_2_O_4_ ([M + H]^+^) calcd 329.2435, found 329.2439.

##### Methyl(2*R*)-2-{(3*S*)-3-[(*tert*-butoxycarbonyl)amino]piperidin-1-yl}-4-methylpentanoate ((2*R*,3*S*)-10c). Method B

Compound (*S*)-2c was coupled with (*S*)-3-Boc-aminopiperidine. The obtained residue was purified by column chromatography (SiO_2_, eluent: EtOAc/*n*-hexane, 1 : 7, v/v) to provide compound (2*R*,3*S*)-10c as transparent oil in an 80% yield (472 mg) with a dr of 100 : 0, [*α*]^20^_D_ = −19.1° (*c* 1.13, MeOH). ^1^H-NMR (700 MHz, CDCl_3_): *δ* 0.88 (d, *J* = 6.6 Hz, 3H, CHCH_2_CH(C*H̲*_3_)_2_), 0.92 (d, *J* = 6.6 Hz, 3H, CHCH_2_CH(C*H̲*_3_)_2_), 1.41–1.48 (m, 1H, Pip 5-H), 1.43 (s, 9H, C(CH_3_)_3_), 1.49–1.58 (m, 3H, Pip 4-H, CHCH_2_C*H̲*(CH_3_)_2_, CHC*H̲*_2_CH(CH_3_)_2_), 1.59–1.69 (m, 3H, Pip 4,5-H and CHC*H̲*_2_CH(CH_3_)_2_), 2.29–2.45 (m, 2H, Pip 2,6-H), 2.55–2.62 (m, 1H, Pip 6-H), 2.78–2.87 (m, 1H, Pip 2-H), 3.22 (t, *J* = 7.6 Hz, 1H, C*H̲*CH_2_CH(CH_3_)_2_), 3.63–3.70 (m, 1H, Pip 3-H), 3.67 (s, 3H, OCH_3_), 4.91 (s, 1H, NH). ^13^C-NMR (176 MHz, CDCl_3_): *δ* 22.3 (CHCH_2_CH(*C̲*H_3_)_2_), 22.9 (Pip 5-C and CHCH_2_CH(*C̲*H_3_)_2_), 25.1 (CHCH_2_*C̲*H(CH_3_)_2_), 28.6 (C(*C̲*H_3_)_3_), 30.2 (Pip 4-C), 38.1 (CH*C̲*H_2_CH(CH_3_)_2_), 46.9 (Pip 3-C), 49.6 (Pip 6-C), 51.0 (OCH_3_), 55.7 (Pip 2-C), 65.6 (*C̲*HCH_2_CH(CH_3_)_2_), 79.1 (*C̲*(CH_3_)_3_), 155.3 (*C̲*OOC(CH_3_)_3_), 173.0 (*C̲*OOCH_3_). IR (FT-IR, *ν*_max_, cm^−1^): 2953, 2869, 1725 (C=O), 1711 (C=O), 1159, 1008, 778. MS *m*/*z* (%): 329 ([M + H]^+^). HRMS (ESI^+^) for C_17_H_33_N_2_O_4_ ([M + H]^+^) calcd 329.2435, found 329.2438.

##### Methyl(2*S*)-2-{(3*S*)-3-[(*tert*-butoxycarbonyl)amino]piperidin-1-yl}-4-methylpentanoate ((2*S*,3*S*)-10c). Method B

Compound (*R*)-2c was coupled with (*S*)-3-Boc-aminopiperidine. The obtained residue was purified by column chromatography (SiO_2_, eluent: EtOAc/*n*-hexane, 1 : 7, v/v) to provide compound (2*S*,3*S*)-10c as white crystals in an 71% yield (419 mg) with a dr of 93 : 7, mp 54–55 °C, [*α*]^20^_D_ = −57.6° (*c* 1.35, MeOH). ^1^H-NMR (700 MHz, CDCl_3_): *δ* 0.89 (d, *J* = 6.6 Hz, 3H, CHCH_2_CH(C*H̲*_3_)_2_), 0.91 (d, *J* = 6.7 Hz, 3H, CHCH_2_CH(C*H̲*_3_)_2_), 1.42–1.56 (m, 4H, Pip 4,5-H, CHCH_2_C*H̲*(CH_3_)_2_ and CHC*H̲*_2_CH(CH_3_)_2_), 1.43 (s, 9H, C(CH_3_)_3_), 1.57–1.70 (m, 3H, Pip 4,5-H and CHC*H̲*_2_CH(CH_3_)_2_), 2.42–2.58 (m, 3H, Pip 2,6-H), 2.62–2.69 (m, 1H, Pip 2-H), 3.24 (t, *J* = 7.7 Hz, 1H, C*H̲*CH_2_CH(CH_3_)_2_), 3.66 (s, 3H, OCH_3_), 3.68–3.73 (m, 1H, Pip 3-H), 4.87 (s, 1H, NH). ^13^C-NMR (176 MHz, CDCl_3_): *δ* 22.4 (CHCH_2_CH(*C̲*H_3_)_2_), 22.9 (CHCH_2_CH(*C̲*H_3_)_2_), 23.0 (Pip 5-C), 25.0 (CHCH_2_*C̲*H(CH_3_)_2_), 28.6 (C(*C̲*H_3_)_3_), 30.2 (Pip 4-C), 38.4 (CH*C̲*H_2_CH(CH_3_)_2_), 46.6 (Pip 3-C), 50.4 (Pip 6-C), 51.0 (OCH_3_), 54.9 (Pip 2-C), 65.6 (*C̲*HCH_2_CH(CH_3_)_2_), 79.1 (*C̲*(CH_3_)_3_), 155.3 (*C̲*OOC(CH_3_)_3_), 173.0 (*C̲*OOCH_3_). IR (FT-IR, *ν*_max_, cm^−1^): 2953, 2867, 1728 (C=O), 1688 (C=O), 1163, 1011, 781. MS *m*/*z* (%): 329 ([M + H]^+^). HRMS (ESI^+^) for C_17_H_33_N_2_O_4_ ([M + H]^+^) calcd 329.2435, found 329.2441

##### Methyl(2*R*)-2-{(3*R*)-3-[(*tert*-butoxycarbonyl)amino]piperidin-1-yl}-4-methylpentanoate ((2*R*,3*R*)-10c). Method B

Compound (*S*)-2c was coupled with (*R*)-3-Boc-aminopiperidine. The obtained residue was purified by column chromatography (SiO_2_, eluent: EtOAc/*n*-hexane, 1 : 7, v/v) to provide compound (2*R*,3*R*)-10c as white crystals in an 81% yield (478 mg) with a dr of 97 : 3, mp 55–57 °C, [*α*]^20^_D_ = 57.8° (*c* 1.16, MeOH). ^1^H-NMR (700 MHz, CDCl_3_): *δ* 0.88 (d, *J* = 6.6 Hz, 3H, CHCH_2_CH(C*H̲*_3_)_2_), 0.91 (d, *J* = 6.7 Hz, 3H, CHCH_2_CH(C*H̲*_3_)_2_), 1.42–1.56 (m, 4H, Pip 4,5-H, CHCH_2_C*H̲*(CH_3_)_2_ and CHC*H̲*_2_CH(CH_3_)_2_), 1.43 (s, 9H, C(CH_3_)_3_), 1.57–1.71 (m, 3H, Pip 4,5-H and CHC*H̲*_2_CH(CH_3_)_2_), 2.42–2.58 (m, 3H, Pip 2,6-H), 2.60–2.69 (m, 1H, Pip 2-H), 3.24 (t, *J* = 7.6 Hz, 1H, C*H̲*CH_2_CH(CH_3_)_2_), 3.66 (s, 3H, OCH_3_), 3.68–3.76 (m, 1H, Pip 3-H), 4.87 (s, 1H, NH). ^13^C-NMR (176 MHz, CDCl_3_): *δ* 22.4 (CHCH_2_CH(*C̲*H_3_)_2_), 22.9 (CHCH_2_CH(*C̲*H_3_)_2_), 23.0 (Pip 5-C), 25.0 (CHCH_2_*C̲*H(CH_3_)_2_), 28.6 (C(*C̲*H_3_)_3_), 30.2 (Pip 4-C), 38.4 (CH*C̲*H_2_CH(CH_3_)_2_), 46.6 (Pip 3-C), 50.4 (Pip 6-C), 51.0 (OCH_3_), 54.8 (Pip 2-C), 65.6 (*C̲*HCH_2_CH(CH_3_)_2_), 79.1 (*C̲*(CH_3_)_3_), 155.3 (*C̲*OOC(CH_3_)_3_), 173.0 (*C̲*OOCH_3_). IR (FT-IR, *ν*_max_, cm^−1^): 2953, 2867, 1728 (C=O), 1688 (C=O), 1163, 1011, 781. MS *m*/*z* (%): 329 ([M + H]^+^). HRMS (ESI^+^) for C_17_H_33_N_2_O_4_ ([M + H]^+^) calcd 329.2435, found 329.2437.

##### Methyl(2*S*)-2-{(3*R*)-3-[(*tert*-butoxycarbonyl)amino]pyrrolidin-1-yl}propanoate ((2*S*,3*R*)-11a). Method A

Compound (*R*)-2a was coupled with (*R*)-3-Boc-aminopyrrolidine. The obtained residue was purified by column chromatography (SiO_2_, eluent : acetone/*n*-hexane, 1 : 5, v/v) to provide compound (2*S*,3*R*)-11a as yellowish oil in an 79% yield (455 mg) with a dr of 100 : 0, [*α*]^20^_D_ = 5.7° (*c* 0.86, MeOH). ^1^H-NMR (700 MHz, CDCl_3_): *δ* 1.32 (d, *J* = 7.1 Hz, 3H, CHC*H̲*_3_), 1.41 (s, 9H, C(CH_3_)_3_), 1.56–1.67 (m, 1H, Pyr 4-H), 2.13–2.22 (m, 1H, Pyr 4-H), 2.48–2.57 (m, 1H, Pyr 5-H), 2.60–2.68 (m, 1H, Pyr 2-H), 2.78–2.87 (m, 2H, Pyr 2,5-H), 3.24 (q, *J* = 7.0 Hz, 1H, C*H̲*CH_3_), 3.69 (s, 3H, OCH_3_), 4.09–4.19 (m, 1H, Pyr 3-H), 4.97 (s, 1H, NH). ^13^C-NMR (176 MHz, CDCl_3_): *δ* 17.1 (CH*C̲*H_3_), 28.5 (C(*C̲*H_3_)_3_), 32.5 (Pyr 4-C), 49.3 (Pyr 5-C), 49.8 (Pyr 3-C), 51.7 (OCH_3_), 57.3 (Pyr 2-C), 60.7 (*C̲*HCH_3_), 79.3 (*C̲*(CH_3_)_3_), 155.5 (*C̲*OOC(CH_3_)_3_), 174.1 (*C̲*OOCH_3_). ^15^N-NMR (71 MHz, CDCl_3_): *δ* −329.8 (Pyr), −283.5 (NH). IR (FT-IR, *ν*_max_, cm^−1^): 2976, 2816, 1734 (C=O), 1709 (C=O), 1158, 1057, 853. MS *m*/*z* (%): 273 ([M + H]^+^). HRMS (ESI^+^) for C_13_H_25_N_2_O_4_ ([M + H]^+^) calcd 273.1809, found 273.1809.

##### Methyl(2*R*)-2-{(3*S*)-3-[(*tert*-butoxycarbonyl)amino]pyrrolidin-1-yl}propanoate ((2*R*,3*S*)-11a). Method A

Compound (*S*)-2a was coupled with (*S*)-3-Boc-aminopyrrolidine. The obtained residue was purified by column chromatography (SiO_2_, eluent : acetone/*n*-hexane, 1 : 6, v/v) to provide compound (2*R*,3*S*)-11a as yellowish oil in an 54% yield (311 mg) with a dr of 89 : 11, [*α*]^20^_D_ = −5.6° (*c* 0.91, MeOH). ^1^H-NMR (700 MHz, CDCl_3_): *δ* 1.31 (d, *J* = 7.1 Hz, 3H, CHC*H̲*_3_), 1.40 (s, 9H, C(CH_3_)_3_), 1.57–1.67 (m, 1H, Pyr 4-H), 2.14–2.21 (m, 1H, Pyr 4-H), 2.49–2.56 (m, 1H, Pyr 5-H), 2.61–2.67 (m, 1H, Pyr 2-H), 2.75–2.87 (m, 2H, Pyr 2,5-H), 3.23 (q, *J* = 7.0 Hz, 1H, C*H̲*CH_3_), 3.68 (s, 3H, OCH_3_), 4.10–4.19 (m, 1H, Pyr 3-H), 5.01 (s, 1H, NH). ^13^C-NMR (176 MHz, CDCl_3_): *δ* 17.1 (CH*C̲*H_3_), 28.5 (C(*C̲*H_3_)_3_), 32.4 (Pyr 4-C), 49.3 (Pyr 5-C), 49.8 (Pyr 3-C), 51.7 (OCH_3_), 57.2 (Pyr 2-C), 60.8 (*C̲*HCH_3_), 79.3 (*C̲*(CH_3_)_3_), 155.5 (*C̲*OOC(CH_3_)_3_), 174.1 (*C̲*OOCH_3_). IR (FT-IR, *ν*_max_, cm^−1^): 2977, 2817, 1735 (C=O), 1708 (C=O), 1158, 1057, 853. MS *m*/*z* (%): 273 ([M + H]^+^). HRMS (ESI^+^) for C_13_H_25_N_2_O_4_ ([M + H]^+^) calcd 273.1809, found 273.1811.

##### Methyl(2*S*)-2-{(3*S*)-3-[(*tert*-butoxycarbonyl)amino]pyrrolidin-1-yl}propanoate ((2*S*,3*S*)-11a). Method A

Compound (*R*)-2a was coupled with (*S*)-3-Boc-aminopyrrolidine. The obtained residue was purified by column chromatography (SiO_2_, eluent : acetone/*n*-hexane, 1 : 5, v/v) to provide compound (2*S*,3*S*)-11a as yellowish oil in an 72% yield (415 mg) with a dr of 91 : 9, [*α*]^20^_D_ = −19.2° (*c* 0.84, MeOH). ^1^H-NMR (700 MHz, CDCl_3_): *δ* 1.33 (d, *J* = 7.0 Hz, 3H, CHC*H̲*_3_), 1.41 (s, 9H, C(CH_3_)_3_), 1.59–1.65 (m, 1H, Pyr 4-H), 2.16–2.23 (m, 1H, Pyr 4-H), 2.46–2.53 (m, 1H, Pyr 5-H), 2.54–2.61 (m, 1H, Pyr 2-H), 2.80–2.92 (m, 2H, Pyr 2,5-H), 3.23 (q, *J* = 7.0 Hz, 1H, C*H̲*CH_3_), 3.70 (s, 3H, OCH_3_), 4.13–4.19 (m, 1H, Pyr 3-H), 4.91 (s, 1H, NH). ^13^C-NMR (176 MHz, CDCl_3_): *δ* 17.1 (CH*C̲*H_3_), 28.5 (C(*C̲*H_3_)_3_), 32.5 (Pyr 4-C), 49.2 (Pyr 5-C), 49.8 (Pyr 3-C), 51.8 (OCH_3_), 57.7 (Pyr 2-C), 60.9 (*C̲*HCH_3_), 79.3 (*C̲*(CH_3_)_3_), 155.5 (*C̲*OOC(CH_3_)_3_), 174.1 (*C̲*OOCH_3_). ^15^N-NMR (71 MHz, CDCl_3_): *δ* −329.1, −283.5 (NH). IR (FT-IR, *ν*_max_, cm^−1^): 2977, 2815, 1709 (C=O), 1693 (C=O), 1160, 1058, 781. MS *m*/*z* (%): 273 ([M + H]^+^). HRMS (ESI^+^) for C_13_H_25_N_2_O_4_ ([M + H]^+^) calcd 273.1809, found 273.1811.

##### Methyl(2*R*)-2-{(3*R*)-3-[(*tert*-butoxycarbonyl)amino]pyrrolidin-1-yl}propanoate ((2*R*,3*R*)-11a). Method A

Compound (*S*)-2a was coupled with (*R*)-3-Boc-aminopyrrolidine. The obtained residue was purified by column chromatography (SiO_2_, eluent : acetone/*n*-hexane, 1 : 5, v/v) to provide compound (2*R*,3*R*)-11a as yellowish oil in an 59% yield (340 mg) with a dr of 88 : 12, [*α*]^20^_D_ = 19.3° (*c* 1.21, MeOH). ^1^H-NMR (700 MHz, CDCl_3_): *δ* 1.34 (d, *J* = 7.0 Hz, 3H, CHC*H̲*_3_), 1.42 (s, 9H, C(CH_3_)_3_), 1.61–1.66 (m, 1H, Pyr 4-H), 2.17–2.23 (m, 1H, Pyr 4-H), 2.48–2.55 (m, 1H, Pyr 5-H), 2.56–2.63 (m, 1H, Pyr 2-H), 2.82–2.93 (m, 2H, Pyr 2,5-H), 3.24 (q, *J* = 7.0 Hz, 1H, C*H̲*CH_3_), 3.70 (s, 3H, OCH_3_), 4.13–4.22 (m, 1H, Pyr 3-H), 4.92 (s, 1H, NH). ^13^C-NMR (176 MHz, CDCl_3_): *δ* 17.1 (CH*C̲*H_3_), 28.5 (C(*C̲*H_3_)_3_), 32.5 (Pyr 4-C), 49.2 (Pyr 5-C), 49.8 (Pyr 3-C), 51.8 (OCH_3_), 57.7 (Pyr 2-C), 61.0 (*C̲*HCH_3_), 79.3 (*C̲*(CH_3_)_3_), 155.5 (*C̲*OOC(CH_3_)_3_), 174.1 (*C̲*OOCH_3_). IR (FT-IR, *ν*_max_, cm^−1^): 2977, 2815, 1709 (C=O), 1693 (C=O), 1160, 1056, 781. MS *m*/*z* (%): 273 ([M + H]^+^). ]^+^). HRMS (ESI^+^) for C_13_H_25_N_2_O_4_ ([M + H]^+^) calcd 273.1809, found 273.1808.

##### Methyl(2*S*)-2-{(3*R*)-3-[(*tert*-butoxycarbonyl)amino]pyrrolidin-1-yl}-3-methylbutanoate ((2*S*,3*R*)-11b). Method B

Compound (*R*)-2b was coupled with (*R*)-3-Boc-aminopyrrolidine. The obtained residue was purified by column chromatography (SiO_2_, eluent: EtOAc/*n*-hexane, 1 : 5, v/v) to provide compound (2*S*,3*R*)-11b as yellowish oil in an 79% yield (449 mg) with a dr of 97 : 3, [*α*]^20^_D_ = 12.1° (*c* 0.94, MeOH). ^1^H-NMR (700 MHz, CDCl_3_): *δ* 0.89 (d, *J* = 6.7 Hz, 3H, CHCH(C*H̲*_3_)_2_), 0.98 (d, *J* = 6.8 Hz, 3H, CHCH(C*H̲*_3_)_2_), 1.43 (s, 9H, C(CH_3_)_3_), 1.54–1.62 (m, 1H, Pyr 4-H), 1.97–2.04 (m, 1H, CHC*H̲*(CH_3_)_2_), 2.11–2.18 (m, 1H, Pyr 4-H), 2.60–2.70 (m, 2H, Pyr 2,5-H), 2.71–2.83 (m, 2H, Pyr 2,5-H), 2.94 (d, *J* = 9.1 Hz, 1H, C*H̲*CH(CH_3_)_2_), 3.68 (s, 3H, OCH_3_), 4.06–4.17 (m, 1H, Pyr 3-H), 4.87 (s, 1H, NH). ^13^C-NMR (176 MHz, CDCl_3_): *δ* 19.2 (CHCH(*C̲*H_3_)_2_), 20.0 (CHCH(*C̲*H_3_)_2_), 28.6 (C(*C̲*H_3_)_3_), 29.1 (CH*C̲*H(CH_3_)_2_), 32.2 (Pyr 4-C), 47.7 (Pyr 5-C), 49.8 (Pyr 3-C), 51.0 (OCH_3_), 56.7 (Pyr 2-C), 71.0 (*C̲*HCH(CH_3_)_3_), 79.3 (*C̲*(CH_3_)_3_), 155.5 (*C̲*OOC(CH_3_)_3_), 172.8 (*C̲*OOCH_3_). IR (FT-IR, *ν*_max_, cm^−1^): 2965, 2815, 1711 (C=O), 1695 (C=O), 1159, 1003, 782. MS *m*/*z* (%): 301 ([M + H]^+^). HRMS (ESI^+^) for C_15_H_29_N_2_O_4_ ([M + H]^+^) calcd 301.2122, found 301.2125.

##### Methyl(2*R*)-2-{(3*S*)-3-[(*tert*-butoxycarbonyl)amino]pyrrolidin-1-yl}-3-methylbutanoate ((2*R*,3*S*)-11b). Method B

Compound (*S*)-2b was coupled with (*S*)-3-Boc-aminopyrrolidine. The obtained residue was purified by column chromatography (SiO_2_, eluent: EtOAc/*n*-hexane, 1 : 5, v/v) to provide compound (2*R*,3*S*)-11b as transparent oil in an 72% yield (409 mg) with a dr of 100 : 0, [*α*]^20^_D_ = −11.8° (*c* 1.12, MeOH). ^1^H-NMR (700 MHz, CDCl_3_): *δ* 0.89 (d, *J* = 6.7 Hz, 3H, CHCH(C*H̲*_3_)_2_), 0.97 (d, *J* = 6.7 Hz, 3H, CHCH(C*H̲*_3_)_2_), 1.42 (s, 9H, C(CH_3_)_3_), 1.53–1.61 (m, 1H, Pyr 4-H), 1.96–2.02 (m, 1H, CHC*H̲*(CH_3_)_2_), 2.10–2.18 (m, 1H, Pyr 4-H), 2.58–2.67 (m, 2H, Pyr 2,5-H), 2.71–2.81 (m, 2H, Pyr 2,5-H), 2.93 (d, *J* = 9.1 Hz, 1H, C*H̲*CH(CH_3_)_2_), 3.67 (s, 3H, OCH_3_), 4.08–4.14 (m, 1H, Pyr 3-H), 4.86 (s, 1H, NH). ^13^C-NMR (176 MHz, CDCl_3_): *δ* 19.2 (CHCH(*C̲*H_3_)_2_), 19.9 (CHCH(*C̲*H_3_)_2_), 28.5 (C(*C̲*H_3_)_3_), 29.1 (CH*C̲*H(CH_3_)_2_), 32.2 (Pyr 4-C), 47.6 (Pyr 5-C), 49.8 (Pyr 3-C), 50.9 (OCH_3_), 56.7 (Pyr 2-C), 71.0 (*C̲*HCH(CH_3_)_3_), 79.3 (*C̲*(CH_3_)_3_), 155.5 (*C̲*OOC(CH_3_)_3_), 172.9 (*C̲*OOCH_3_). IR (FT-IR, *ν*_max_, cm^−1^): 2965, 2815, 1711 (C=O), 1694 (C=O), 1159, 1003, 782. MS *m*/*z* (%): 301 ([M + H]^+^). HRMS (ESI^+^) for C_15_H_29_N_2_O_4_ ([M + H]^+^) calcd 301.2122, found 301.2122.

##### Methyl(2*S*)-2-{(3*S*)-3-[(*tert*-butoxycarbonyl)amino]pyrrolidin-1-yl}-3-methylbutanoate ((2*S*,3*S*)-11b). Method B

Compound (*R*)-2b was coupled with (*S*)-3-Boc-aminopyrrolidine. The obtained residue was purified by column chromatography (SiO_2_, eluent: EtOAc/*n*-hexane, 1 : 5, v/v) to provide compound (2*S*,3*S*)-11b as white crystals in an 76% yield (431 mg) with a dr of 96 : 4, mp 47–49 °C, [*α*]^20^_D_ = −24.1° (*c* 1.00, MeOH). ^1^H-NMR (700 MHz, CDCl_3_): *δ* 0.88 (d, *J* = 6.7 Hz, 3H, CHCH(C*H̲*_3_)_2_), 0.97 (d, *J* = 6.8 Hz, 3H, CHCH(C*H̲*_3_)_2_), 1.42 (s, 9H, C(CH_3_)_3_), 1.53–1.61 (m, 1H, Pyr 4-H), 1.94–2.03 (m, 1H, CHC*H̲*(CH_3_)_2_), 2.09–2.18 (m, 1H, Pyr 4-H), 2.43–2.56 (m, 2H, Pyr 2,5-H), 2.82–2.91 (m, 2H, Pyr 2,5-H), 2.94 (d, *J* = 9.2 Hz, 1H, C*H̲*CH(CH_3_)_2_), 3.67 (s, 3H, OCH_3_), 4.07–4.15 (m, 1H, Pyr 3-H), 4.77 (s, 1H, NH). ^13^C-NMR (176 MHz, CDCl_3_): *δ* 19.2 (CHCH(*C̲*H_3_)_2_), 19.9 (CHCH(*C̲*H_3_)_2_), 28.5 (C(*C̲*H_3_)_3_), 28.8 (CH*C̲*H(CH_3_)_2_), 32.1 (Pyr 4-C), 48.7 (Pyr 5-C), 49.7 (Pyr 3-C), 50.9 (OCH_3_), 55.7 (Pyr 2-C), 71.0 (*C̲*HCH(CH_3_)_3_), 79.3 (*C̲*(CH_3_)_3_), 155.5 (*C̲*OOC(CH_3_)_3_), 172.7 (*C̲*OOCH_3_). IR (FT-IR, *ν*_max_, cm^−1^): 2966, 2875, 1723 (C=O), 1685 (C=O), 1149, 1004, 782. MS *m*/*z* (%): 301 ([M + H]^+^). HRMS (ESI^+^) for C_15_H_28_N_2_NaO_4_ ([M + Na]^+^) calcd 323.1941, found 323.1940.

##### Methyl(2*R*)-2-{(3*R*)-3-[(*tert*-butoxycarbonyl)amino]pyrrolidin-1-yl}-3-methylbutanoate ((2*R*,3*R*)-11b). Method B

Compound (*S*)-2b was coupled with (*R*)-3-Boc-aminopyrrolidine. The obtained residue was purified by column chromatography (SiO_2_, eluent: EtOAc/*n*-hexane, 1 : 5, v/v) to provide compound (2*R*,3*R*)-11b as white crystals in an 77% yield (438 mg) with a dr of 100 : 0, mp 49–51 °C, [*α*]^20^_D_ = 24.4° (*c* 0.96, MeOH). ^1^H-NMR (700 MHz, CDCl_3_): *δ* 0.88 (d, *J* = 6.7 Hz, 3H, CHCH(C*H̲*_3_)_2_), 0.97 (d, *J* = 6.7 Hz, 3H, CHCH(C*H̲*_3_)_2_), 1.42 (s, 9H, C(CH_3_)_3_), 1.53–1.62 (m, 1H, Pyr 4-H), 1.95–2.03 (m, 1H, CHC*H̲*(CH_3_)_2_), 2.09–2.17 (m, 1H, Pyr 4-H), 2.43–2.56 (m, 2H, Pyr 2,5-H), 2.81–2.91 (m, 2H, Pyr 2,5-H), 2.94 (d, *J* = 9.2 Hz, 1H, C*H̲*CH(CH_3_)_2_), 3.67 (s, 3H, OCH_3_), 4.06–4.17 (m, 1H, Pyr 3-H), 4.78 (s, 1H, NH). ^13^C-NMR (176 MHz, CDCl_3_): *δ* 19.2 (CHCH(*C̲*H_3_)_2_), 19.9 (CHCH(*C̲*H_3_)_2_), 28.5 (C(*C̲*H_3_)_3_), 28.8 (CH*C̲*H(CH_3_)_2_), 32.1 (Pyr 4-C), 48.7 (Pyr 5-C), 49.7 (Pyr 3-C), 50.9 (OCH_3_), 55.7 (Pyr 2-C), 71.0 (*C̲*HCH(CH_3_)_3_), 79.3 (*C̲*(CH_3_)_3_), 155.5 (*C̲*OOC(CH_3_)_3_), 172.6 (*C̲*OOCH_3_). IR (FT-IR, *ν*_max_, cm^−1^): 2966, 2875, 1723 (C=O), 1685 (C=O), 1149, 1004, 782. MS *m*/*z* (%): 301 ([M + H]^+^). HRMS (ESI^+^) for C_15_H_29_N_2_O_4_ ([M + H]^+^) calcd 301.2122, found 301.2124.

##### Methyl(2*S*)-2-{(3*R*)-3-[(*tert*-butoxycarbonyl)amino]pyrrolidin-1-yl}-4-methylpentanoate ((2*S*,3*R*)-11c). Method B

Compound (*R*)-2c was coupled with (*R*)-3-Boc-aminopyrrolidine. The obtained residue was purified by column chromatography (SiO_2_, eluent: EtOAc/*n*-hexane, 1 : 7, v/v) to provide compound (2*S*,3*R*)-11c as yellow oil in an 61% yield (345 mg) with a dr of 96 : 4, [*α*]^20^_D_ = 20.1° (*c* 1.01, MeOH). ^1^H-NMR (700 MHz, CDCl_3_): *δ* 0.88 (d, *J* = 6.3 Hz, 3H, CHCH_2_CH(C*H̲*_3_)_2_), 0.91 (d, *J* = 6.4 Hz, 3H, CHCH_2_CH(C*H̲*_3_)_2_), 1.42 (s, 9H, C(CH_3_)_3_), 1.48–1.55 (m, 1H, CHC*H̲*_2_CH(CH_3_)_2_), 1.56–1.64 (m, 3H, CHCH_2_C*H̲*(CH_3_)_2_, Pyr 4-H and CHC*H̲*_2_CH(CH_3_)_2_), 2.09–2.17 (m, 1H, Pyr 4-H), 2.61–2.71 (m, 2H, Pyr 2,5-H), 2.73–2.89 (m, 2H, Pyr 2,5-H), 3.28–3.37 (m, 1H, C*H̲*CH_2_CH(CH_3_)_2_), 3.68 (s, 3H, OCH_3_), 4.07–4.17 (m, 1H, Pyr 3-H), 4.95 (s, 1H, NH). ^13^C-NMR (176 MHz, CDCl_3_): *δ* 22.6 (CHCH_2_CH(*C̲*H_3_)_2_), 22.8 (CHCH_2_CH(*C̲*H_3_)_2_), 25.3 (CHCH_2_*C̲*H(CH_3_)_2_), 28.5 (C(*C̲*H_3_)_3_), 32.4 (Pyr 4-C), 40.2 (CH*C̲*H_2_CH(CH_3_)_2_), 48.2 (Pyr 5-C), 49.9 (Pyr 3-C), 51.3 (OCH_3_), 56.5 (Pyr 2-C), 62.9 (*C̲*HCH_2_CH(CH_3_)_2_), 79.3 (*C̲*(CH_3_)_3_), 155.5 (*C̲*OOC(CH_3_)_3_), 173.9 (*C̲*OOCH_3_). IR (FT-IR, *ν*_max_, cm^−1^): 2956, 2870, 1722 (C=O), 1711 (C=O), 1157, 1077, 781. MS *m*/*z* (%): 315 ([M + H]^+^). HRMS (ESI^+^) for C_16_H_31_N_2_O_4_ ([M + H]^+^) calcd 315.2278, found 315.2285.

##### Methyl(2*R*)-2-{(3*S*)-3-[(*tert*-butoxycarbonyl)amino]pyrrolidin-1-yl}-4-methylpentanoate ((2*R*,3*S*)-11c). Method B

Compound (*S*)-2c was coupled with (*S*)-3-Boc-aminopyrrolidine. The obtained residue was purified by column chromatography (SiO_2_, eluent: EtOAc/*n*-hexane, 1 : 7, v/v) to provide compound (2*R*,3*S*)-11c as transparent oil in an 67% yield (379 mg) with a dr of 98 : 2, [*α*]^20^_D_ = −19.9° (*c* 1.08, MeOH). ^1^H-NMR (700 MHz, CDCl_3_): *δ* 0.88 (d, *J* = 6.3 Hz, 3H, CHCH_2_CH(C*H̲*_3_)_2_), 0.91 (d, *J* = 6.4 Hz, 3H, CHCH_2_CH(C*H̲*_3_)_2_), 1.42 (s, 9H, C(CH_3_)_3_), 1.47–1.55 (m, 1H, CHC*H̲*_2_CH(CH_3_)_2_), 1.56–1.64 (m, 3H, CHCH_2_C*H̲*(CH_3_)_2_, Pyr 4-H and CHC*H̲*_2_CH(CH_3_)_2_), 2.09–2.19 (m, 1H, Pyr 4-H), 2.60–2.71 (m, 2H, Pyr 2,5-H), 2.73–2.89 (m, 2H, Pyr 2,5-H), 3.29–3.35 (m, 1H, C*H̲*CH_2_CH(CH_3_)_2_), 3.68 (s, 3H, OCH_3_), 4.06–4.17 (m, 1H, Pyr 3-H), 4.95 (s, 1H, NH). ^13^C-NMR (176 MHz, CDCl_3_): *δ* 22.6 (CHCH_2_CH(*C̲*H_3_)_2_), 22.8 (CHCH_2_CH(*C̲*H_3_)_2_), 25.3 (CHCH_2_*C̲*H(CH_3_)_2_), 28.5 (C(*C̲*H_3_)_3_), 32.4 (Pyr 4-C), 40.2 (CH*C̲*H_2_CH(CH_3_)_2_), 48.2 (Pyr 5-C), 49.9 (Pyr 3-C), 51.3 (OCH_3_), 56.5 (Pyr 2-C), 62.9 (*C̲*HCH_2_CH(CH_3_)_2_), 79.3 (*C̲*(CH_3_)_3_), 155.5 (*C̲*OOC(CH_3_)_3_), 173.9 (*C̲*OOCH_3_). IR (FT-IR, *ν*_max_, cm^−1^): 2956, 2870, 1720 (C=O), 1711 (C=O), 1156, 1077, 781. MS *m*/*z* (%): 315 ([M + H]^+^). HRMS (ESI^+^) for C_16_H_31_N_2_O_4_ ([M + H]^+^) calcd 315.2278, found 315.2284.

##### Methyl(2*S*)-2-{(3*S*)-3-[(*tert*-butoxycarbonyl)amino]pyrrolidin-1-yl}-4-methylpentanoate ((2*S*,3*S*)-11c). Method B

Compound (*R*)-2c was coupled with (*S*)-3-Boc-aminopyrrolidine. The obtained residue was purified by column chromatography (SiO_2_, eluent: EtOAc/*n*-hexane, 1 : 7, v/v) to provide compound (2*S*,3*S*)-11c as yellowish oil in an 65% yield (367 mg) with a dr of 93 : 7, [*α*]^20^_D_ = −11.6° (*c* 0.94, MeOH). ^1^H-NMR (700 MHz, CDCl_3_): *δ* 0.89 (d, *J* = 6.4 Hz, 3H, CHCH_2_CH(C*H̲*_3_)_2_), 0.91 (d, *J* = 6.4 Hz, 3H, CHCH_2_CH(C*H̲*_3_)_2_), 1.42 (s, 9H, C(CH_3_)_3_), 1.49–1.54 (m, 1H, CHC*H̲*_2_CH(CH_3_)_2_), 1.55–1.66 (m, 3H, CHCH_2_C*H̲*(CH_3_)_2_, Pyr 4-H and CHC*H̲*_2_CH(CH_3_)_2_), 2.09–2.19 (m, 1H, Pyr 4-H), 2.48–2.56 (m, 2H, Pyr 2,5-H), 2.86–2.98 (m, 2H, Pyr 2,5-H), 3.29–3.35 (m, 1H, C*H̲*CH_2_CH(CH_3_)_2_), 3.68 (s, 3H, OCH_3_), 4.08–4.19 (m, 1H, Pyr 3-H), 4.83 (s, 1H, NH). ^13^C-NMR (176 MHz, CDCl_3_): *δ* 22.6 (CHCH_2_CH(*C̲*H_3_)_2_), 22.9 (CHCH_2_CH(*C̲*H_3_)_2_), 25.2 (CHCH_2_*C̲*H(CH_3_)_2_), 28.5 (C(*C̲*H_3_)_3_), 32.4 (Pyr 4-C), 40.1 (CH*C̲*H_2_CH(CH_3_)_2_), 48.5 (Pyr 5-C), 49.8 (Pyr 3-C), 51.4 (OCH_3_), 56.5 (Pyr 2-C), 63.0 (*C̲*HCH_2_CH(CH_3_)_2_), 79.3 (*C̲*(CH_3_)_3_), 155.5 (*C̲*OOC(CH_3_)_3_), 173.8 (*C̲*OOCH_3_). IR (FT-IR, *ν*_max_, cm^−1^): 2956, 2870, 1711 (C=O), 1696 (C=O), 1158, 1008, 781. MS *m*/*z* (%): 315 ([M + H]^+^). HRMS (ESI^+^) for C_16_H_31_N_2_O_4_ ([M + H]^+^) calcd 315.2278, found 315.2279.

##### Methyl(2*R*)-2-{(3*R*)-3-[(*tert*-butoxycarbonyl)amino]pyrrolidin-1-yl}-4-methylpentanoate ((2*R*,3*R*)-11c). Method B

Compound (*S*)-2c was coupled with (*R*)-3-Boc-aminopyrrolidine. The obtained residue was purified by column chromatography (SiO_2_, eluent: EtOAc/*n*-hexane, 1 : 7, v/v) to provide compound (2*R*,3*R*)-11c as transparent oil in an 71% yield (401 mg) with a dr of 97 : 3, [*α*]^20^_D_ = 11.6° (*c* 1.01, MeOH). ^1^H-NMR (700 MHz, CDCl_3_): *δ* 0.89 (d, *J* = 6.3 Hz, 3H, CHCH_2_CH(C*H̲*_3_)_2_), 0.91 (d, *J* = 6.4 Hz, 3H, CHCH_2_CH(C*H̲*_3_)_2_), 1.42 (s, 9H, C(CH_3_)_3_), 1.49–1.54 (m, 1H, CHC*H̲*_2_CH(CH_3_)_2_), 1.55–1.66 (m, 3H, CHCH_2_C*H̲*(CH_3_)_2_, Pyr 4-H and CHC*H̲*_2_CH(CH_3_)_2_), 2.10–2.19 (m, 1H, Pyr 4-H), 2.46–2.51 (m, 2H, Pyr 2,5-H), 2.86–2.98 (m, 2H, Pyr 2,5-H), 3.29–3.36 (m, 1H, C*H̲*CH_2_CH(CH_3_)_2_), 3.68 (s, 3H, OCH_3_), 4.09–4.17 (m, 1H, Pyr 3-H), 4.85 (s, 1H, NH). ^13^C-NMR (176 MHz, CDCl_3_): *δ* 22.6 (CHCH_2_CH(*C̲*H_3_)_2_), 22.9 (CHCH_2_CH(*C̲*H_3_)_2_), 25.2 (CHCH_2_*C̲*H(CH_3_)_2_), 28.5 (C(*C̲*H_3_)_3_), 32.4 (Pyr 4-C), 40.1 (CH*C̲*H_2_CH(CH_3_)_2_), 48.6 (Pyr 5-C), 49.8 (Pyr 3-C), 51.4 (OCH_3_), 56.5 (Pyr 2-C), 63.0 (*C̲*HCH_2_CH(CH_3_)_2_), 79.3 (*C̲*(CH_3_)_3_), 155.5 (*C̲*OOC(CH_3_)_3_), 173.8 (*C̲*OOCH_3_). IR (FT-IR, *ν*_max_, cm^−1^): 2956, 2870, 1711 (C=O), 1696 (C=O), 1158, 1008, 781. MS *m*/*z* (%): 315 ([M + H]^+^). HRMS (ESI^+^) for C_16_H_31_N_2_O_4_ ([M + H]^+^) calcd 315.2278, found 315.2284.

### Protocol used for investigation of ee values (diastereomers 5a–c)

Compounds 3a–c (100 mg) were dissolved in DCM (1 mL) and treated with TFA (1 mL). The solutions were stirred at r.t. for 30 min. After removal of the solvent *in vacuo*, the obtained ammonium salts were neutralized with Cs_2_CO_3_ (1 equiv.) in order to generate amines 4a–c, which were directly used in the next step without further purification.

2-Formylphenylboronic acid (1 equiv.) was dissolved in CDCl_3_ (3 mL) and treated with (*R*)-1,1′-bi-2-naphthol ((*R*)-BINOL) (1.1 equiv.). The solution was stirred at 40 °C for 30 min. Then, the corresponding amine (4a–c) (1 equiv.) was added to reaction mixture with 4 Å molecular sieves and stirred at r.t. for 18 h. The reaction mixture was transferred to an NMR tube for ^1^H NMR analysis.

### Synthesis of (piperidin-1-yl)propanoic acid (6)

The corresponding ester (3a) (200 mg) was dissolved in MeOH (2 mL) and treated with 2 N NaOH (1 mL). The solution was stirred at r.t. for 2 h. After removal of the solvent *in vacuo*, the residue was purified by flash chromatography (EtOAc→MeOH).

#### (2*S*)-2-{4-[(*tert*-butoxycarbonyl)amino]piperidin-1-yl}propanoic acid ((*S*)-6)

Yellowish oil, yield 171 mg (90%), [*α*]^20^_D_ = 3.5° (*c* 0.86, MeOH). ^1^H-NMR (700 MHz, CDCl_3_): *δ* 1.38–1.47 (m, 3H, CHC*H̲*_3_), 1.41 (s, 9H, C(CH_3_)_3_), 1.91–2.10 (m, 4H, Pip 3,5-H), 2.57–2.73 (m, 1H, Pip 6-H), 2.75–2.84 (m, 1H, Pip 2-H), 3.37–3.54 (m, 3H, Pip 2,6-H and C*H̲*CH_3_), 3.56–3.67 (m, 1H, Pip 4-H), 4.84–5.01 (m, 2H, NH and COOH). ^13^C-NMR (176 MHz, CDCl_3_): *δ* 13.3 (CH*C̲*H_3_), 28.6 (C(*C̲*H_3_)_3_), 29.7 (Pip 5-C), 29.9 (Pip 3-C), 46.0 (Pip 4-C), 48.3 (Pip 6-C), 51.2 (Pip 2-C), 65.8 (*C̲*HCH_3_), 79.5 (*C̲*(CH_3_)_3_), 155.6 (*C̲*OOC(CH_3_)_3_), 173.1 (*C̲*OOH). IR (FT-IR, *ν*_max_, cm^−1^): 3427, 2977, 1687 (C=O), 1617 (C=O), 1167, 1016, 863. MS *m*/*z* (%): 273 ([M + H]^+^). HRMS (ESI^+^) for C_13_H_25_N_2_O_4_ ([M + H]^+^) calcd 273,1809, found 273.1805.

#### (2*R*)-2-{4-[(*tert*-butoxycarbonyl)amino]piperidin-1-yl}propanoic acid ((*R*)-6)

Yellowish oil, yield 185 mg (97%), [*α*]^20^_D_ = −3.7° (*c* 0.93, MeOH). ^1^H-NMR (700 MHz, CDCl_3_): *δ* 1.35–1.47 (m, 3H, CHC*H̲*_3_), 1.41 (s, 9H, C(CH_3_)_3_), 1.88–2.10 (m, 4H, Pip 3,5-H), 2.58–2.70 (m, 1H, Pip 6-H), 2.73–2.83 (m, 1H, Pip 2-H), 3.33–3.53 (m, 3H, Pip 2,6-H and C*H̲*CH_3_), 3.55–3.67 (m, 1H, Pip 4-H), 4.94–5.30 (m, 2H, NH and COOH). ^13^C-NMR (176 MHz, CDCl_3_): *δ* 13.3 (CH*C̲*H_3_), 28.6 (C(*C̲*H_3_)_3_), 29.8 (Pip 5-C), 30.0 (Pip 3-C), 46.0 (Pip 4-C), 48.3 (Pip 6-C), 51.1 (Pip 2-C), 65.8 (*C̲*HCH_3_), 79.5 (*C̲*(CH_3_)_3_), 155.6 (*C̲*OOC(CH_3_)_3_), 173.3 (*C̲*OOH). IR (FT-IR, *ν*_max_, cm^−1^): 3427, 2978, 1688 (C=O), 1615 (C=O), 1162, 1018, 863. MS *m*/*z* (%): 273 ([M + H]^+^). HRMS (ESI^+^) for C_13_H_25_N_2_O_4_ ([M + H]^+^) calcd 273.1809, found 273.1809.

### Synthesis of peptides (7)

DIPEA (0.1 mL, 1 equiv.) was added to a mixture of acid 6 (150 mg, 1 equiv.) and HATU (210 mg, 1 equiv.) in DMF (10 mL), and the solution was stirred at r.t. for 5 min. Then l-phenylalanine ethyl ester hydrochloride (127 mg, 1 equiv.) was added to reaction mixture. After 30 min DIPEA (0.2 mL, 2 equiv.) was added to solution and stirred at r.t. for 1 h. Reaction mixture was diluted with EtOAc (10 mL), washed with 1 M KHSO_4_ (10 mL), 1 M NaHCO_3_ (10 mL) and brine (10 mL). The organic layer was dried with anhydrous sodium sulfate, filtered, and concentrated under reduced pressure. The crude product was purified by flash chromatography (SiO_2_, eluent : acetone/*n*-hexane, 1 : 3, v/v).

#### Ethyl-*N*-[(2*S*)-2-{4-[(*tert*-butoxycarbonyl)amino]piperidin-1-yl}propanoyl]-l-phenylalaninate ((*S*,*S*)-7)

Transparent oil in an 59% yield (146 mg) with a dr of 94 : 6, [*α*]^20^_D_ = −2.1° (*c* 0.96, MeOH). ^1^H-NMR (700 MHz, CDCl_3_): *δ* 1.09–1.17 (m, 1H, Pip 3-H), 1.12 (d, *J* = 7.1 Hz, 3H, CHC*H̲*_3_), 1.24–1.33 (m, 1H, Pip 5-H), 1.26 (t, *J* = 7.2 Hz, 3H, COOCH_2_C*H̲*_3_), 1.44 (s, 9H, C(CH_3_)_3_), 1.77–1.85 (m, 2H, Pip 3,5-H), 2.05–2.12 (m, 1H, Pip 6-H), 2.24–2.29 (m, 1H, Pip 2-H), 2.43–2.48 (m, 1H, Pip 6-H), 2.61–2.69 (m, 1H, Pip 2-H), 3.01 (q, *J* = 7.0 Hz, 1H, C*H̲*CH_3_), 3.05–3.10 (m, 1H, PhC*H̲*_2_CH), 3.17–3.22 (m, 1H, PhC*H̲*_2_CH), 3.35–3.43 (m, 1H, Pip 4-H), 4.19 (q, *J* = 7.2 Hz, 2H, COOC*H̲*_2_CH_3_), 4.39 (s, 1H, NHBoc), 4.83 (q, *J* = 6.9 Hz, 6.1 Hz, 1H, PhCH_2_C*H̲*), 7.10–7.14 (m, 2H, Ph 2,6-H), 7.21–7.24 (m, 1H, Ph 4-H), 7.25–7.29 (m, 2H, Ph 3,5-H), 7.62 (d, *J* = 8.1 Hz, 1H, NHCO). ^13^C-NMR (176 MHz, CDCl_3_): *δ* 11.9 (CH*C̲*H_3_), 14.3 (COOCH_2_*C̲*H_3_), 28.5 (C(*C̲*H_3_)_3_), 32.9 (Pip 3-C), 33.0 (Pip 5-C), 38.0 (Ph*C̲*H_2_CH), 46.8 (Pip 6-C), 47.5 (Pip 2-C), 51.1 (Pip 4-C), 52.5 (PhCH_2_*C̲*H), 61.6 (COO*C̲*H_2_CH_3_), 63.9 (*C̲*HCH_3_), 79.4 (*C̲*(CH_3_)_3_), 127.1 (Ph 4-C), 128.6 (Ph 3,5-C), 129.3 (Ph 2,6-C), 136.2 (Ph 1-C), 155.2 (*C̲*OOC(CH_3_)_3_), 171.9 (*C̲*OOCH_2_CH_3_), 173.9 (*C̲*ONH). IR (FT-IR, *ν*_max_, cm^−1^): 3330, 2977, 2936, 1669 (C=O), 1499, 1169, 1021, 700. MS *m*/*z* (%): 448 ([M + H]^+^). HRMS (ESI^+^) for C_24_H_38_N_3_O_5_ ([M + H]^+^) calcd 448.2806, found 448.2809.

#### Ethyl-*N*-[(2*R*)-2-{4-[(*tert*-butoxycarbonyl)amino]piperidin-1-yl}propanoyl]-l-phenylalaninate ((*R*,*S*)-7)

Transparent oil in an 64% yield (158 mg) with a dr of 90 : 10, [*α*]^20^_D_ = −13.6° (*c* 1.27, MeOH). ^1^H-NMR (700 MHz, CDCl_3_): *δ* 1.11–1.20 (m, 1H, Pip 3-H), 1.17 (d, *J* = 7.1 Hz, 3H, CHC*H̲*_3_), 1.22–1.28 (m, 1H, Pip 5-H), 1.24 (t, *J* = 7.2 Hz, 3H, COOCH_2_C*H̲*_3_), 1.45 (s, 9H, C(CH_3_)_3_), 1.80–1.86 (m, 2H, Pip 3,5-H), 2.09–2.15 (m, 1H, Pip 2-H), 2.33–2.40 (m, 1H, Pip 6-H), 2.51–2.56 (m, 1H, Pip 6-H), 2.62–2.67 (m, 1H, Pip 2-H), 3.06 (q, *J* = 7.1 Hz, 1H, C*H̲*CH_3_), 3.13 (d, *J* = 6.1 Hz, 2H, PhC*H̲*_2_CH), 3.35–3.44 (m, 1H, Pip 4-H), 4.17 (q, *J* = 7.2 Hz, 2H, COOC*H̲*_2_CH_3_), 4.40 (s, 1H, NHBoc), 4.83 (dt, *J* = 8.9 Hz, 6.1 Hz, 1H, PhCH_2_C*H̲*), 7.12–7.15 (m, 2H, Ph 2,6-H), 7.23–7.27 (m, 1H, Ph 4-H), 7.27–7.31 (m, 2H, Ph 3,5-H), 7.71 (d, *J* = 8.7 Hz, 1H, NHCO). ^13^C-NMR (176 MHz, CDCl_3_): *δ* 10.7 (CH*C̲*H_3_), 14.2 (COOCH_2_*C̲*H_3_), 28.5 (C(*C̲*H_3_)_3_), 32.7 (Pip 3-C), 33.2 (Pip 5-C), 37.9 (Ph*C̲*H_2_CH), 46.6 (Pip 2-C), 47.4 (Pip 4-C), 50.9 (Pip 6-C), 52.4 (PhCH_2_*C̲*H), 61.5 (COO*C̲*H_2_CH_3_), 63.7 (*C̲*HCH_3_), 79.4 (*C̲*(CH_3_)_3_), 127.1 (Ph 4-C), 128.7 (Ph 3,5-C), 129.4 (Ph 2,6-C), 136.3 (Ph 1-C), 155.2 (*C̲*OOC(CH_3_)_3_), 171.8 (*C̲*OOCH_2_CH_3_), 173.7 (*C̲*ONH). IR (FT-IR, *ν*_max_, cm^−1^): 3332, 2977, 2936, 1671 (C=O), 1498, 1169, 1022, 700. MS *m*/*z* (%): 448 ([M + H]^+^). HRMS (ESI^+^) for C_24_H_38_N_3_O_5_ ([M + H]^+^) calcd 448.2806, found 448.2808.

### Synthesis of nosyl-peptides (9)

The corresponding *N*-Boc-dipeptide (100 mg) was dissolved in DCM (1 mL) and treated with TFA (1 mL). The solution was stirred at r.t. for 30 min. After removal of the solvent *in vacuo*, the obtained amine was used directly in the next step without further purification.

4-Nitrobenzenesulfonyl chloride (50 mg, 1 equiv.) was added to a mixture of the corresponding amine (78 mg, 1 equiv.) and 1 M Na_2_CO_3_ (1 mL, 4 equiv.) in acetonitrile (15 mL), and the solution was stirred at r.t. for 1 hour. The reaction mixture was diluted with EtOAc (20 mL) and washed with H_2_O (2 × 15 mL) and brine (15 mL). The organic layer was dried with anhydrous sodium sulfate, filtered, and then concentrated under reduced pressure. The crude product was purified by flash chromatography (SiO_2_, eluent : acetone/*n*-hexane, 1 : 1, v/v).

#### Ethyl-*N*-[(2*S*)-2-{4-[(4-nitrobenzene-1-sulfonyl)amino]piperidin-1-yl}propanoyl]-l-phenylalaninate ((*S*,*S*)-9)

Yellowish oil in an 86% yield (102 mg) with a dr of 94 : 6, [*α*]^20^_D_ = −7.6° (*c* 0.81, MeOH). ^1^H-NMR (700 MHz, CDCl_3_): *δ* 1.08 (d, *J* = 7.0 Hz, 3H, CHC*H̲*_3_), 1.20–1.26 (m, 1H, Pip 3-H), 1.24 (t, *J* = 7.2 Hz, 3H, COOCH_2_C*H̲*_3_), 1.34–1.39 (m, 1H, Pip 5-H), 1.63–1.70 (m, 2H, Pip 3,5-H), 1.98–2.05 (m, 1H, Pip 6-H), 2.15–2.20 (m, 1H, Pip 2-H), 2.39–2.44 (m, 1H, Pip 6-H), 2.57–2.65 (m, 1H, Pip 2-H), 2.97 (q, *J* = 7.1 Hz, 1H, C*H̲*CH_3_), 3.02–3.07 (m, 1H, PhC*H̲*_2_CH), 3.09–3.15 (m, 1H, Pip 4-H), 3.15–3.21 (m, 1H, PhC*H̲*_2_CH), 4.16 (q, *J* = 7.1 Hz, 2H, COOC*H̲*_2_CH_3_), 4.77–4.83 (m, 1H, PhCH_2_C*H̲*), 5.16 (d, *J* = 7.7 Hz, 1H, NHSO_2_), 7.07–7.10 (m, 2H, Ph 2,6-H), 7.17–7.20 (m, 1H, Ph 4-H), 7.21–7.25 (m, 2H, Ph 3,5-H), 7.47 (d, *J* = 8.2 Hz, 1H, NHCO), 8.06 (d, *J* = 8.8 Hz, 2H, PhNO_2_ 2,6-H), 8.35 (d, *J* = 8.8 Hz, 2H, PhNO_2_ 3,5-H). ^13^C-NMR (176 MHz, CDCl_3_): *δ* 12.0 (CH*C̲*H_3_), 14.2 (COOCH_2_*C̲*H_3_), 33.4 (Pip 3,5-C), 37.8 (Ph*C̲*H_2_CH), 46.6 (Pip 6-C), 50.5 (Pip 2-C), 51.1 (Pip 4-C), 52.5 (PhCH_2_*C̲*H), 61.6 (COO*C̲*H_2_CH_3_), 63.8 (*C̲*HCH_3_), 124.5 (PhNO_2_ 3,5-C), 127.1 (Ph 4-C), 128.2 (PhNO_2_ 2,6-C), 128.6 (Ph 3,5-C), 129.3 (Ph 2,6-C), 136.1 (Ph 1-C), 147.3 (PhNO_2_ 4-C), 150.1 (PhNO_2_ 1-C), 171.9 (*C̲*OOCH_2_CH_3_), 173.6 (*C̲*ONH). ^15^N-NMR (71 MHz, CDCl_3_): *δ* −332.1 (Pip), −274.5 (NH–SO_2_), −271.8 (NH–CO), −16.0 (NO_2_). IR (FT-IR, *ν*_max_, cm^−1^): 3269, 3104, 2927, 1659 (C=O), 1528 (N–O), 1348 (S=O), 1163 (C=O), 1092, 736. MS *m*/*z* (%): 533 ([M + H]^+^). HRMS (ESI^+^) for C_25_H_33_N_4_O_7_S ([M + H]^+^) calcd 533.2064, found 533.2068.

#### Ethyl-*N*-[(2*R*)-2-{4-[(4-nitrobenzene-1-sulfonyl)amino]piperidin-1-yl}propanoyl]-l-phenylalaninate ((*R*,*S*)-9)

Yellowish oil in an 89% yield (106 mg) with a dr of 90 : 10, [*α*]^20^_D_ = −14.2° (*c* 0.99, MeOH). ^1^H-NMR (700 MHz, CDCl_3_): *δ* 1.12 (d, *J* = 7.0 Hz, 3H, CHC*H̲*_3_), 1.18–1.26 (m, 1H, Pip 3-H), 1.22 (t, *J* = 7.2 Hz, 3H, COOCH_2_C*H̲*_3_), 1.30–1.40 (m, 1H, Pip 5-H), 1.63–1.73 (m, 2H, Pip 3,5-H), 2.00–2.07 (m, 1H, Pip 2-H), 2.25–2.32 (m, 1H, Pip 6-H), 2.47–2.53 (m, 1H, Pip 6-H), 2.56–2.62 (m, 1H, Pip 2-H), 3.04 (q, *J* = 7.1 Hz, 1H, C*H̲*CH_3_), 3.08–3.17 (m, 3H, Pip 4-H and PhC*H̲*_2_CH), 4.11–4.18 (m, 2H, COOC*H̲*_2_CH_3_), 4.80 (dt, *J* = 8.7 Hz, 6.0 Hz, 1H, PhCH_2_C*H̲*), 5.05 (d, *J* = 7.7 Hz, 1H, NHSO_2_), 7.07–7.11 (m, 2H, Ph 2,6-H), 7.19–7.23 (m, 1H, Ph 4-H), 7.23–7.27 (m, 2H, Ph 3,5-H), 7.56 (d, *J* = 8.6 Hz, 1H, NHCO), 8.05 (d, *J* = 8.8 Hz, 2H, PhNO_2_ 2,6-H), 8.35 (d, *J* = 8.8 Hz, 2H, PhNO_2_ 3,5-H). ^13^C-NMR (176 MHz, CDCl_3_): *δ* 10.5 (CH*C̲*H_3_), 14.2 (COOCH_2_*C̲*H_3_), 33.2 (Pip 3-C), 33.7 (Pip 5-C), 37.9 (Ph*C̲*H_2_CH), 46.3 (Pip 2-C), 50.3 (Pip 6-C), 51.0 (Pip 4-C), 52.4 (PhCH_2_*C̲*H), 61.6 (COO*C̲*H_2_CH_3_), 63.6 (*C̲*HCH_3_), 124.5 (PhNO_2_ 3,5-C), 127.1 (Ph 4-C), 128.2 (PhNO_2_ 2,6-C), 128.7 (Ph 3,5-C), 129.4 (Ph 2,6-C), 136.2 (Ph 1-C), 147.3 (PhNO_2_ 4-C), 150.1 (PhNO_2_ 1-C), 171.8 (*C̲*OOCH_2_CH_3_), 173.3 (*C̲*ONH). ^15^N-NMR (71 MHz, CDCl_3_): *δ* −332.9 (Pip), −274.5 (NH–SO_2_), −271.3 (NH–CO), −16.0 (NO_2_). IR (FT-IR, *ν*_max_, cm^−1^): 3328, 3176, 2938, 1657 (C=O), 1528 (N–O), 1348 (S=O), 1165 (C=O), 1091, 734. MS *m*/*z* (%): 533 ([M + H]^+^). HRMS (ESI^+^) for C_25_H_33_N_4_O_7_S ([M + H]^+^) calcd 533.2064, found 533.2066.

## Conclusions

In this study, we prepared a series of new heterocyclic analogues of *N*-(ω-aminoalkylene)amino acid derivatives as chiral building blocks. The method was based on the conversion of enantiopure α-hydroxy acid esters into the corresponding chiral triflate esters, which were displaced in a nucleophilic substitution S_N_2 reaction with aminopyrrolidine and aminopiperidine derivatives by the inversion of the configuration to produce methyl 2-[(Boc-amino)cycloamin-1-yl]alkanoates with a good yield and with high enantiomeric and diastereomeric purity. The synthesized 2-[(Boc-amino)piperidin-1-yl]proponoates combined with methyl-l-phenylalaninate, produced new chiral *N*-Boc- and *N*-nosyl-dipeptides containing a piperidine moiety. The structures were elucidated by ^1^H-, ^13^C-, and ^15^N-NMR spectroscopy, high-resolution mass spectrometry, and X-ray crystallography analysis.

## Author contributions

Conceptualization, F. A. S. and A. Š.; methodology, F. A. S., A. Š. and N. K.; formal analysis, G. M., A. B. and G. R.; investigation, G. M., U. Š., R. J.; M. R. B. and A. B; resources, F. A. S. and A. Š.; data curation, F. A. S., A. Š. and N. K.; writing—original draft preparation, G. M., V. M. and A. Š.; writing—review and editing, G. M., A. Š., V. M. and F. A. S.; visualization, G. M., A. B., V. M., F. A. S. and A. Š.; supervision, A. Š. and F. A. S.; funding acquisition, F. A. S. and A. Š. All authors have read and agreed to the published version of the manuscript.

## Conflicts of interest

There are no conflicts to declare.

## Supplementary Material

RA-013-D3RA03060A-s001

RA-013-D3RA03060A-s002
