# Supplementary material for: Facile synthesis of new N-(aminocycloalkylene)amino acid compounds using chiral triflate esters with N-Boc-aminopyrrolidines and N-Boc-aminopiperidines
Source: RSC Adv. 2023 Jul 18;13(31):21378–94. doi: 10.1039/d3ra03060a (PMC10353522; doi:10.1039/d3ra03060a)
Supplement: RA-013-D3RA03060A-s001 [file RA-013-D3RA03060A-s001.pdf]

Electronic Supplementary Material (ESI) for RSC Advances.
This journal is © The Royal Society of Chemistry 2023

Supplemental Material

# Facile synthesis of new *N*-(aminocycloalkylene)amino acid compounds using chiral triflate esters with *N*-Boc-aminopyrrolidines and *N*-Boc-aminopiperidines

Gita Matulevičiūtė, [a,b] Neringa Kleizienė, [a] Greta Račkauskienė, [a] Vytas Martynaitis, [b] Aurimas Bieliauskas, [a] Urtė Šachlevičiūtė, [a] Rokas Jankauskas, [a] Martynas R. Bartkus, [a] Frank A. Sløk [c] and Algirdas Šačkus [*,a,b]

[a] Institute of Synthetic Chemistry, Kaunas University of Technology,
K. Baršausko g. 59, Kaunas LT-51423, Lithuania;

[b] Department of Organic Chemistry, Kaunas University of Technology,
Radvilėnų pl. 19, Kaunas LT-50254, Lithuania;

[c] Vipergen ApS,
Gammel Kongevej 23A, Copenhagen V DK-1610, Denmark

* Corresponding authors

E-mail address: algirdas.sackus@ktu.lt (A. Šačkus), neringa.kleiziene@ktu.lt (N. Kleizienė)

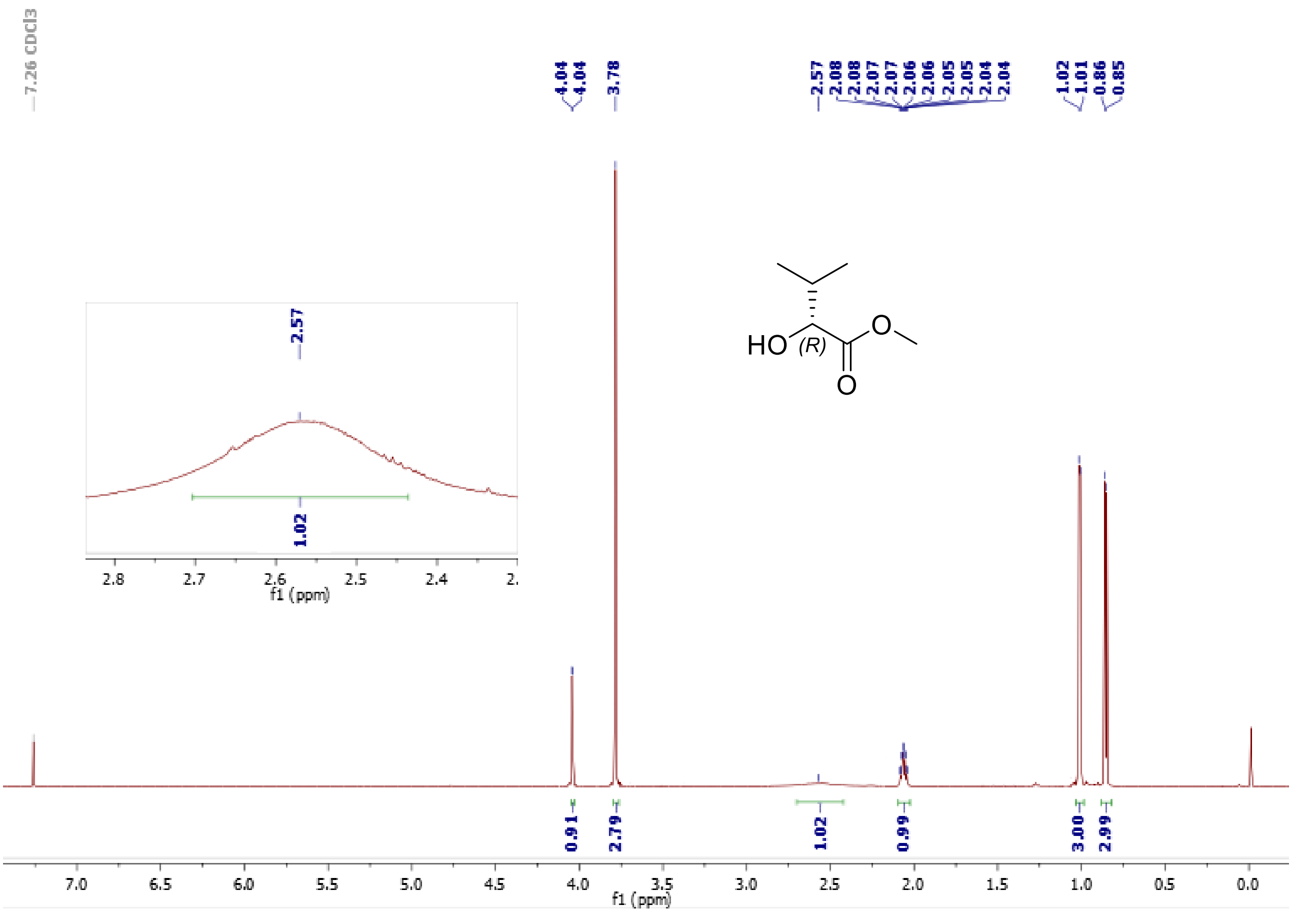


**Figure S1. Methyl (2*R*)-2-hydroxy-3-methylbutanoate ((*R*)-1b). $^{1}$H NMR spectrum (700 MHz, $CDCl_3$).**

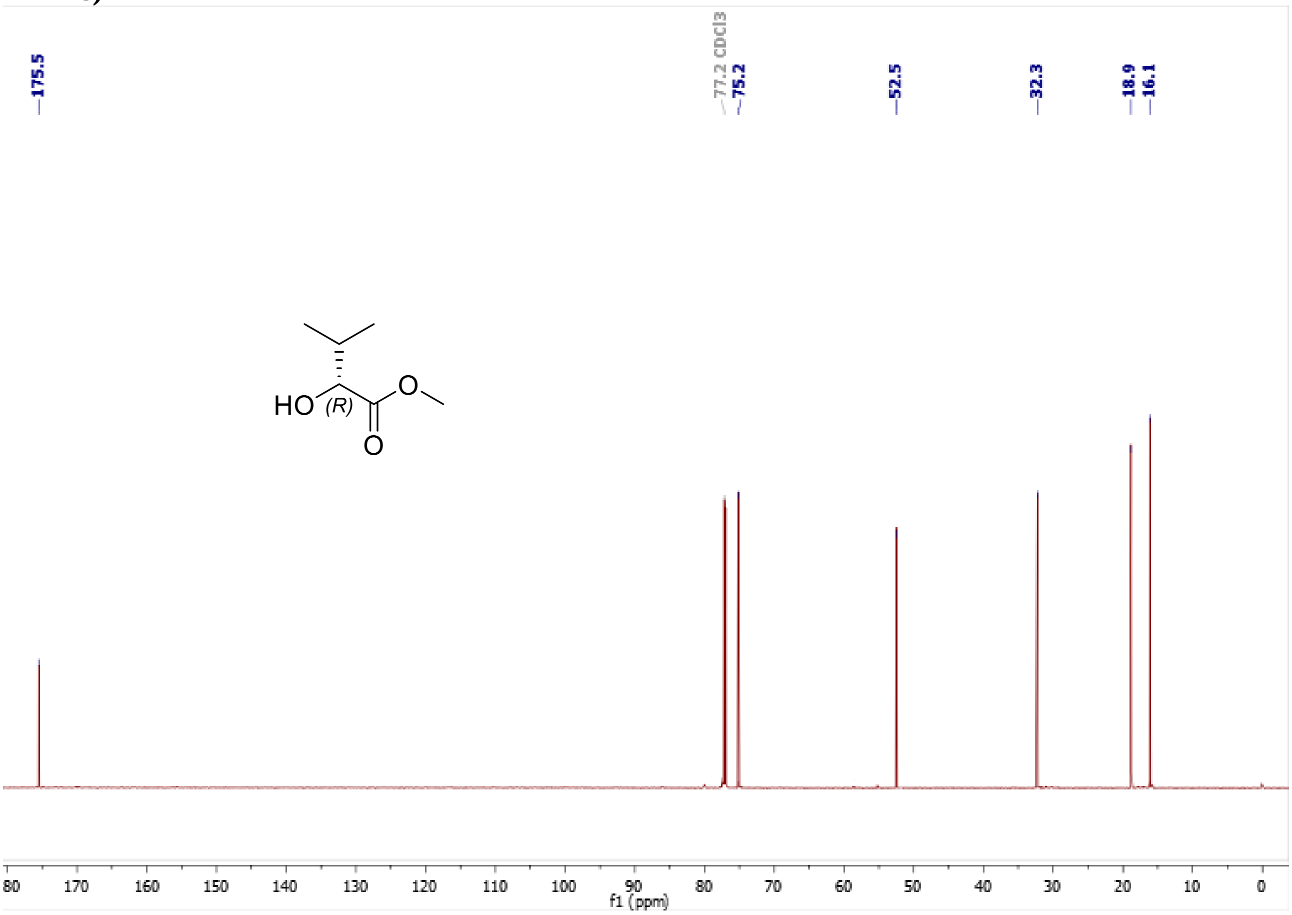


**Figure S2. Methyl (2*R*)-2-hydroxy-3-methylbutanoate ((*R*)-1b). $^{13}$C NMR spectrum (176 MHz, $CDCl_3$).**

# Compound Spectrum SmartFormula Report

**Analysis Info**

| | | | |
|---|---|---|---|
| Analysis Name | D:\Data\GMP-959.d | Acquisition Date | 5/2/2023 7:43:47 PM |
| Method | DirectInfusion_TuneLow_pos.m | Operator | hplc |
| Sample Name | GMP-959 | Instrument | micrOTOF-Q III 8228888.20448 |
| Comment | AB | | |

**Acquisition Parameter**

| | | | | | |
|---|---|---|---|---|---|
| Source Type | ESI | Ion Polarity | Positive | Set Nebulizer | 0.4 Bar |
| Focus | Not active | Set Capillary | 4500 V | Set Dry Heater | 180 °C |
| Scan Begin | 50 m/z | Set End Plate Offset | -500 V | Set Dry Gas | 4.0 l/min |
| Scan End | 1000 m/z | Set Collision Cell RF | 140.0 Vpp | Set Divert Valve | Waste |

| # | RT [min] | Area | Int. Type | I | S/N | Chromatogram | Max. m/z | FWHM [min] |
|---|---|---|---|---|---|---|---|---|
| n.a. | 6.1 | n.a. | Single spectrum | n.a. | n.a. | n.a. | 155.0680 | n.a. |

**+MS, 6.1min #367**

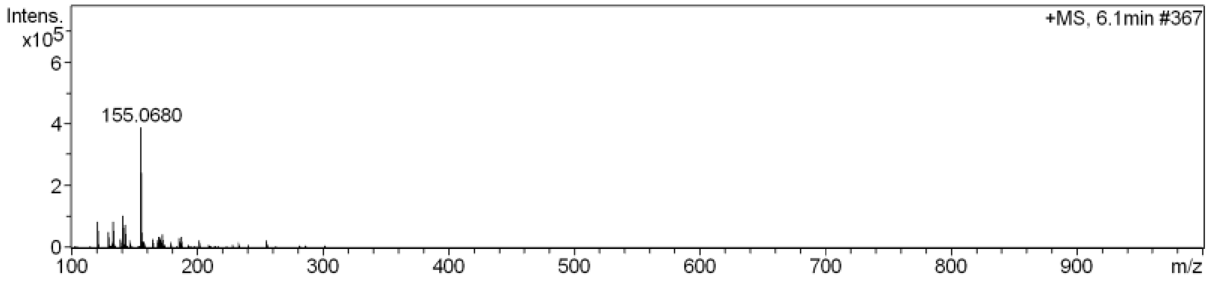


| Meas. m/z | # | Ion Formula | m/z | err [ppm] | mSigma | # Sigma | Score | rdb | e⁻ Conf | N-Rule |
|---|---|---|---|---|---|---|---|---|---|---|
| 155.0680 | 1 | C6H12NaO3 | 155.0679 | -0.8 | 25.6 | 1 | 100.00 | 0.5 | even | ok |

**Figure S3. Methyl (2*R*)-2-hydroxy-3-methylbutanoate ((*R*)-1b). HRMS (ESI-TOF).**

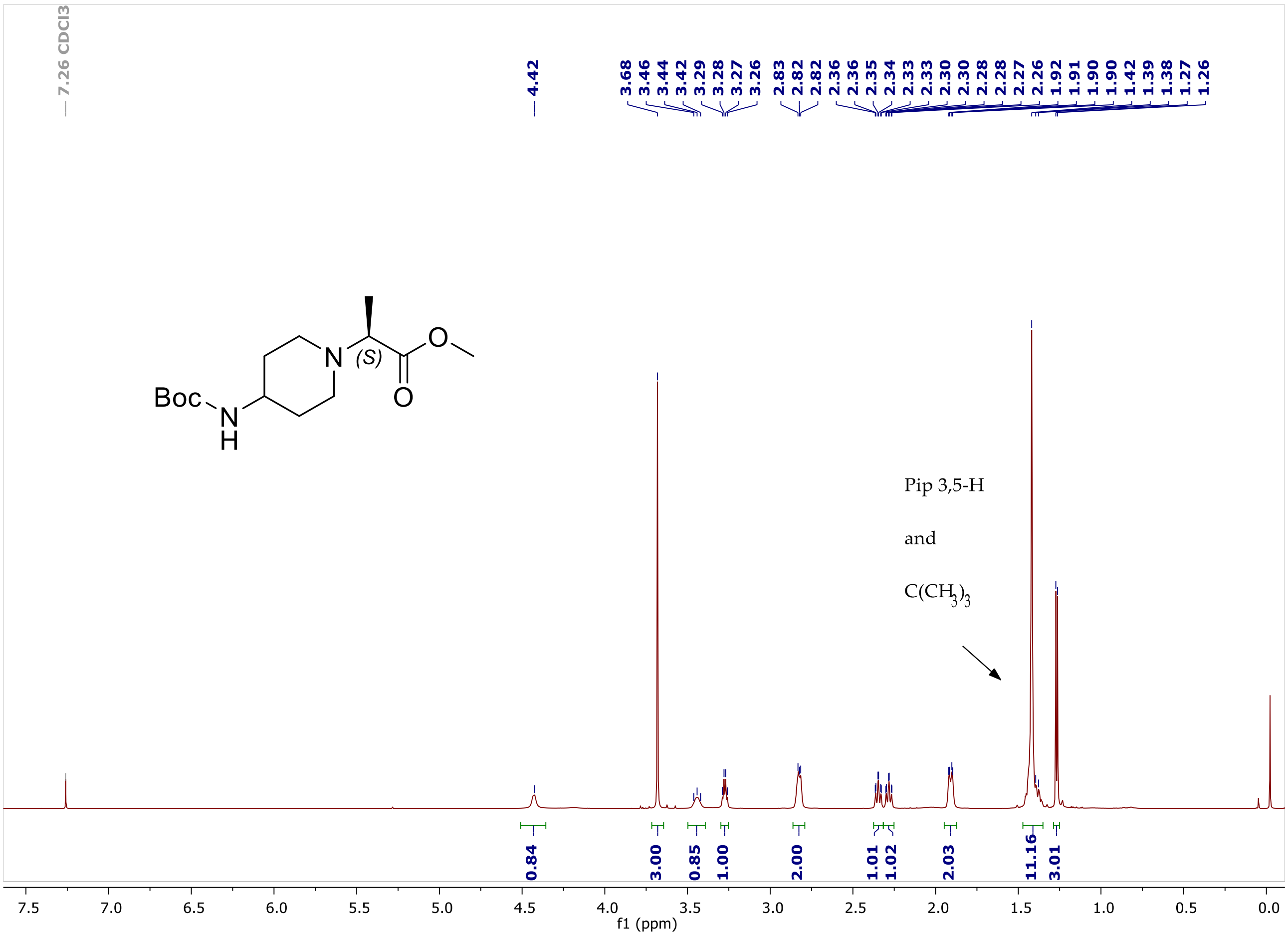


**Figure S4. Methyl (2*S*)-2-{4-[(*tert*-butoxycarbonyl)amino]piperidin-1-yl}propanoate ((*S*)-3a). $^{1}$H NMR spectrum (700 MHz, $CDCl_3$).**

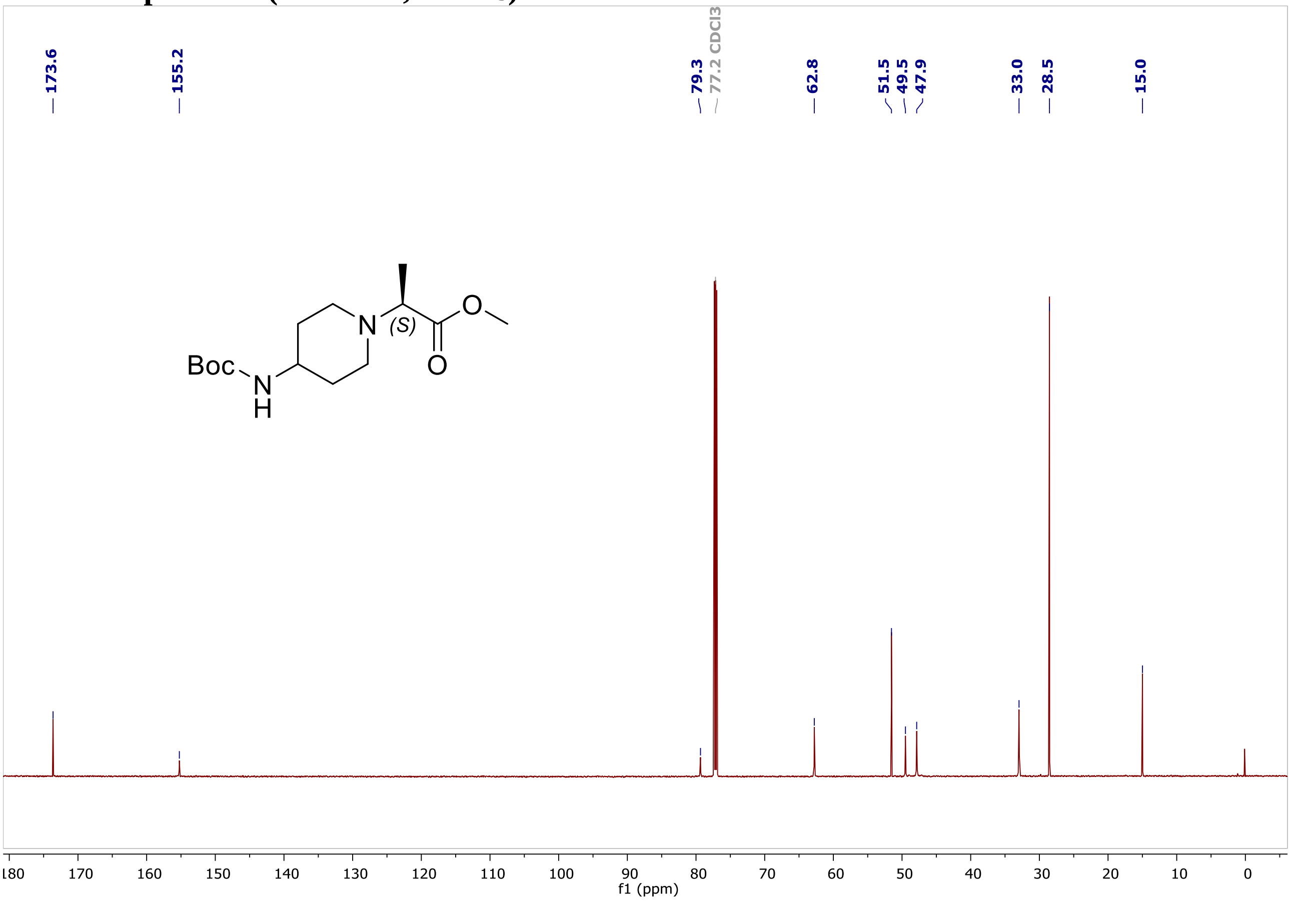


**Figure S5. Methyl (2*S*)-2-{4-[(*tert*-butoxycarbonyl)amino]piperidin-1-yl}propanoate ((*S*)-3a). $^{13}$C NMR spectrum (176 MHz, $CDCl_3$).**

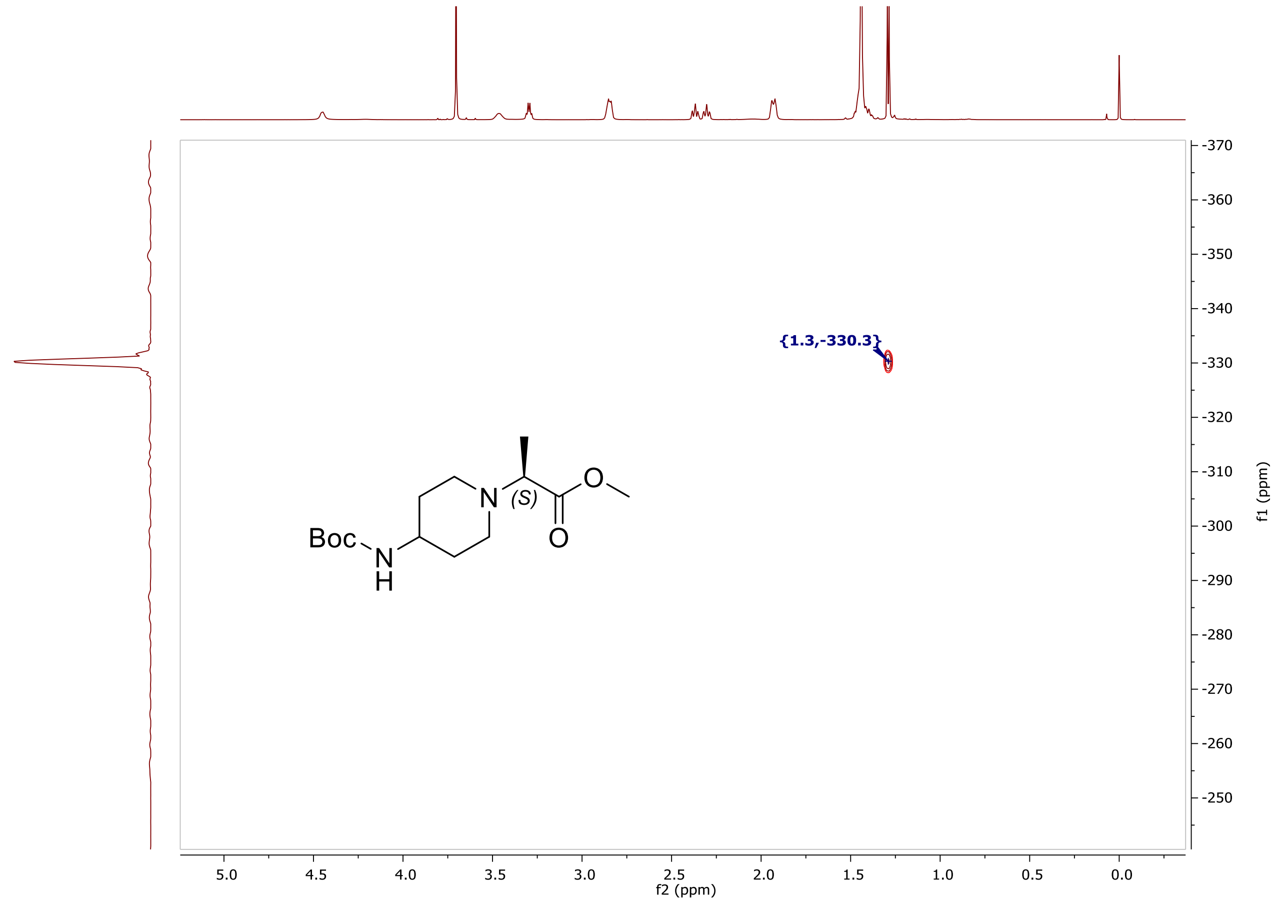


**Figure S6. Methyl (2*S*)-2-{4-[(*tert*-butoxycarbonyl)amino]piperidin-1-yl}propanoate ((*S*)-3a). $^{1}$H-$^{15}$N HMBC spectrum (71 MHz, CDCl$_3$).**

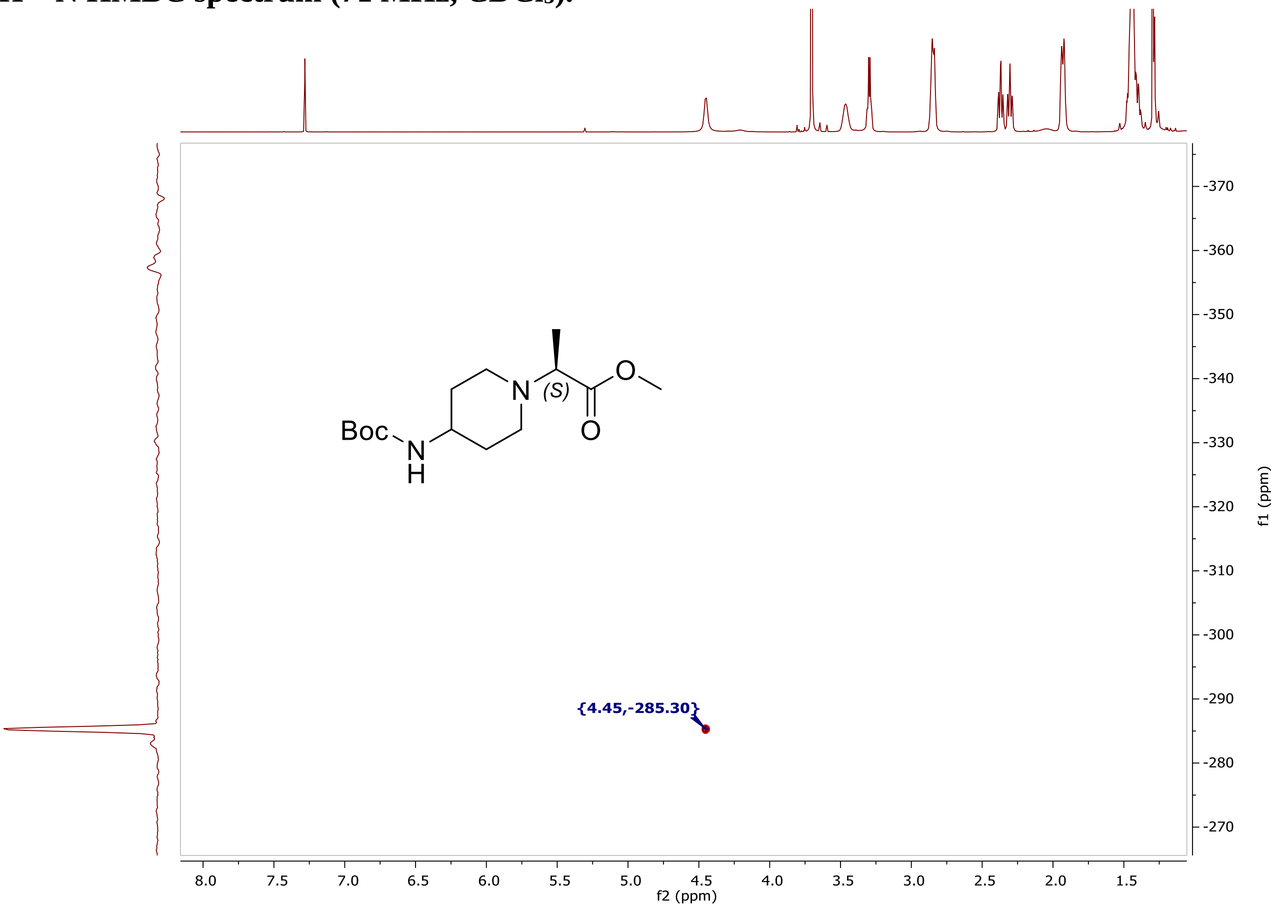


**Figure S7. Methyl (2*S*)-2-{4-[(*tert*-butoxycarbonyl)amino]piperidin-1-yl}propanoate ((*S*)-3a). $^{1}$H-$^{15}$N HSQC spectrum (71 MHz, CDCl$_3$).**

# Mass Spectrum SmartFormula Report

**Analysis Info**

| | | | |
|---|---|---|---|
| Analysis Name | D:\Data\Organikai\2023_04_11\GMP_358_1-B,3_01_10216.d | Acquisition Date | 4/14/2023 4:10:20 PM |
| Method | organikai_esi_pos_2013_recover.m | Operator | Milda Pukalskiene |
| Sample Name | GMP_358 | Instrument / Ser# | maXis 4G 20218 |
| Comment | | | |

**Acquisition Parameter**

| | | | | | |
|---|---|---|---|---|---|
| Source Type | ESI | Ion Polarity | Positive | Set Nebulizer | 1.5 Bar |
| Focus | Not active | Set Capillary | 4500 V | Set Dry Heater | 180 °C |
| Scan Begin | 40 m/z | Set End Plate Offset | -500 V | Set Dry Gas | 8.0 l/min |
| Scan End | 1800 m/z | Set Collision Cell RF | 350.0 Vpp | Set Divert Valve | Waste |

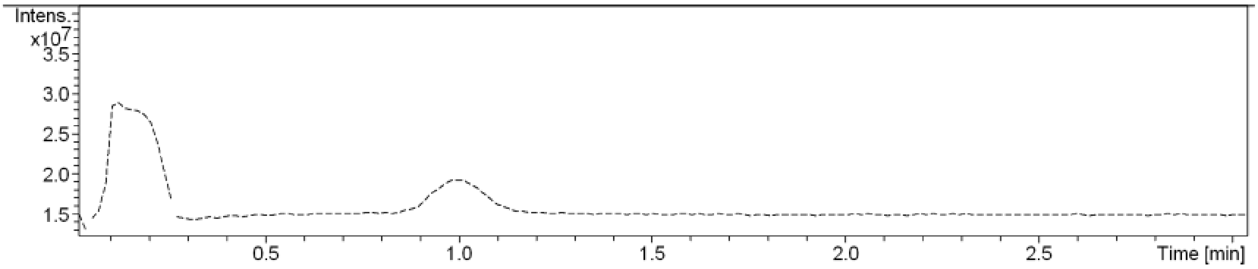


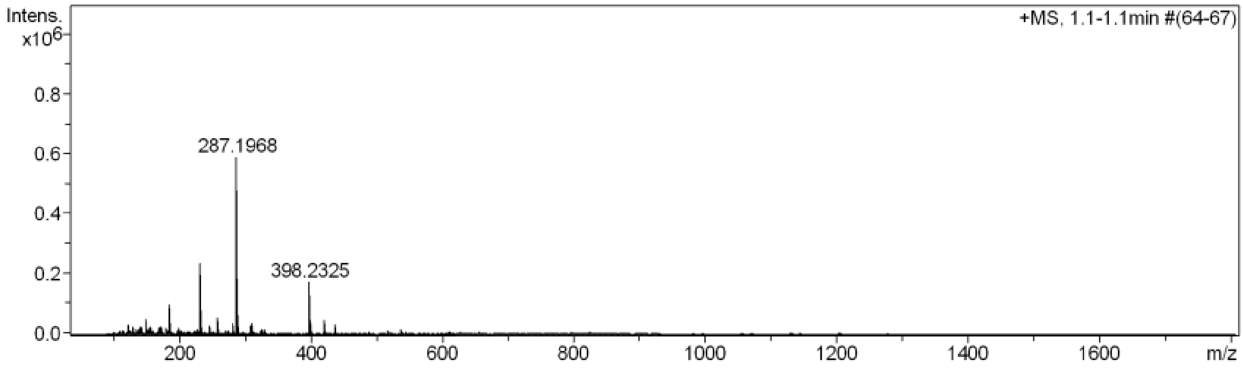


| Meas. m/z | # | Formula | Score | m/z | err [ppm] | Mean err [ppm] | mSigma | rdb | e⁻ Conf | N-Rule |
|---|---|---|---|---|---|---|---|---|---|---|
| 287.1968 | 1 | C 14 H 27 N 2 O 4 | 100.00 | 287.1965 | -0.8 | -0.5 | 15.5 | 2.5 | even | ok |

**Figure S8. Methyl (2*S*)-2-{4-[(*tert*-butoxycarbonyl)amino]piperidin-1-yl}propanoate ((*S*)-3a). HRMS (ESI-TOF).**

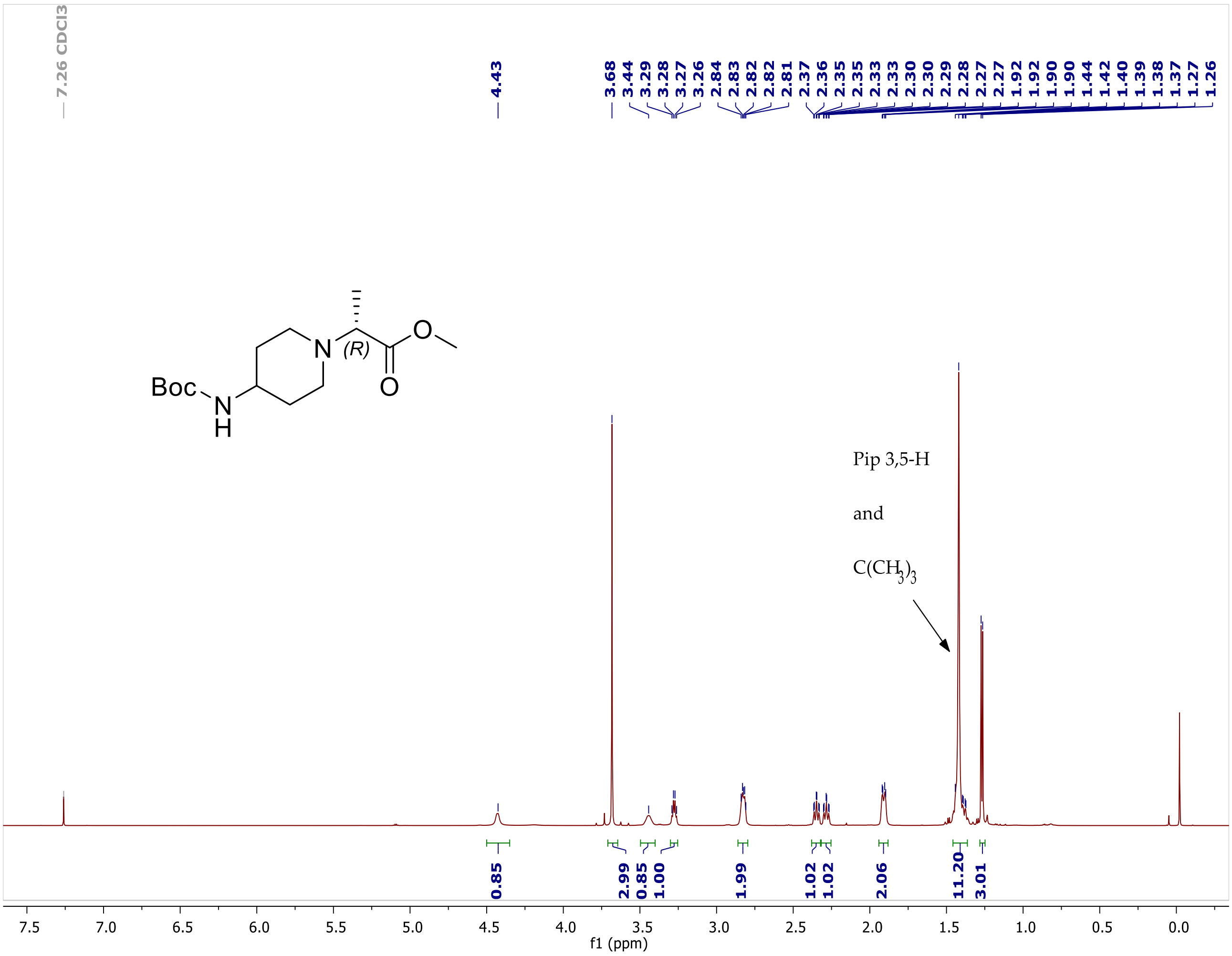


**Figure S9. Methyl (2*R*)-2-{4-[(*tert*-butoxycarbonyl)amino]piperidin-1-yl}propanoate ((*R*)-3a).** **$^{1}$H NMR spectrum (700 MHz, $CDCl_3$).**

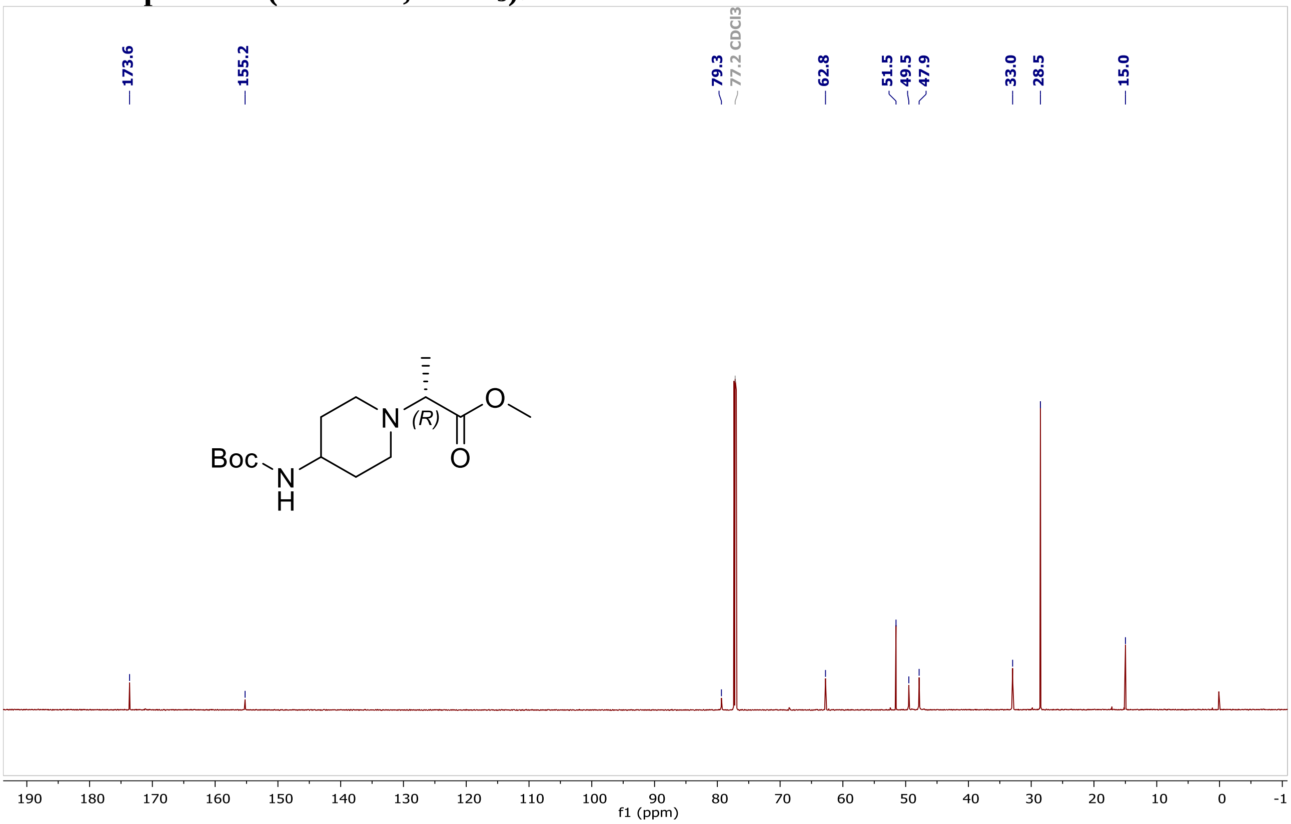


**Figure S10. Methyl (2*R*)-2-{4-[(*tert*-butoxycarbonyl)amino]piperidin-1-yl}propanoate ((*R*)-3a).** **$^{13}$C NMR spectrum (176 MHz, $CDCl_3$).**

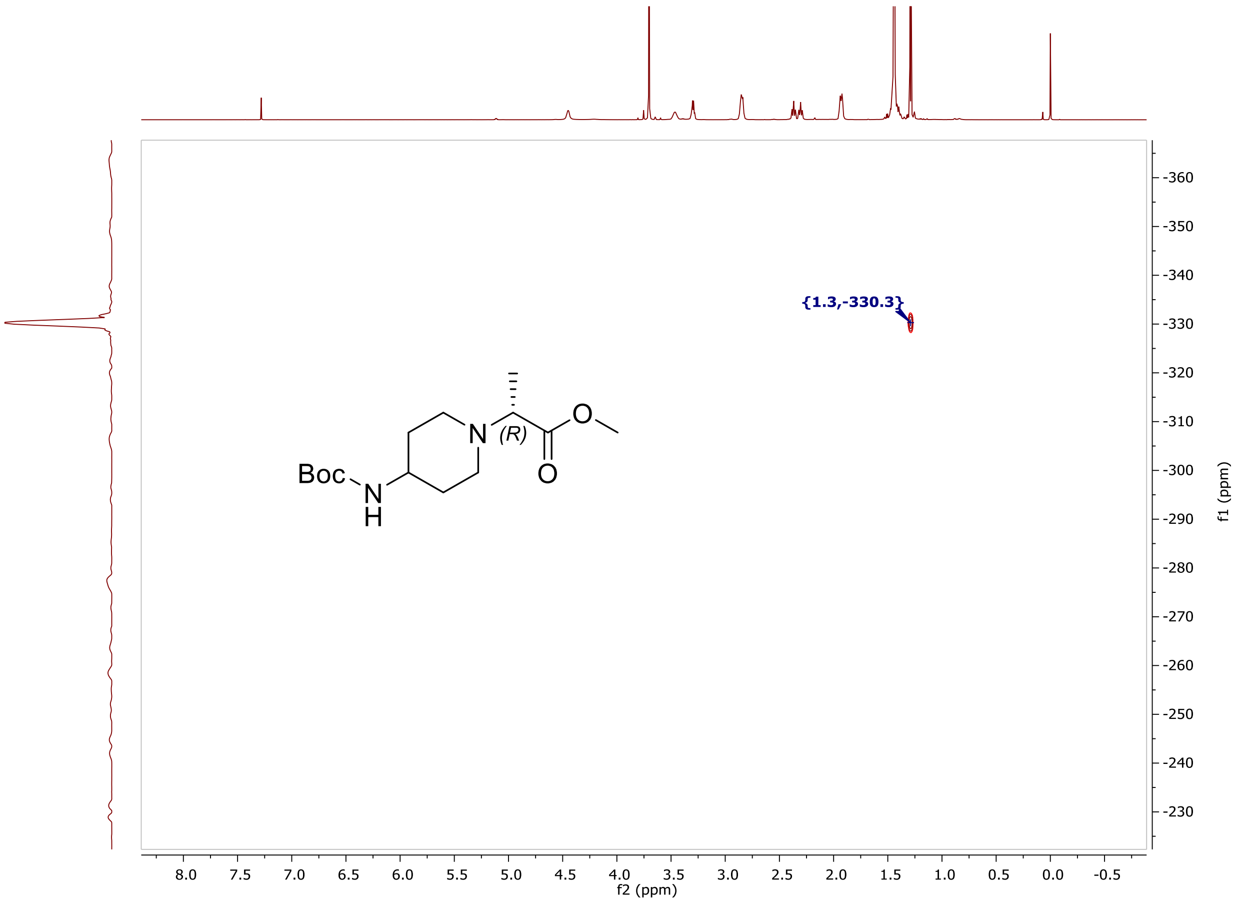


**Figure S11. Methyl (2*R*)-2-{4-[(*tert*-butoxycarbonyl)amino]piperidin-1-yl}propanoate ((*R*)-3a). $^{1}H$-$^{15}N$ HMBC spectrum (71 MHz, $CDCl_3$).**

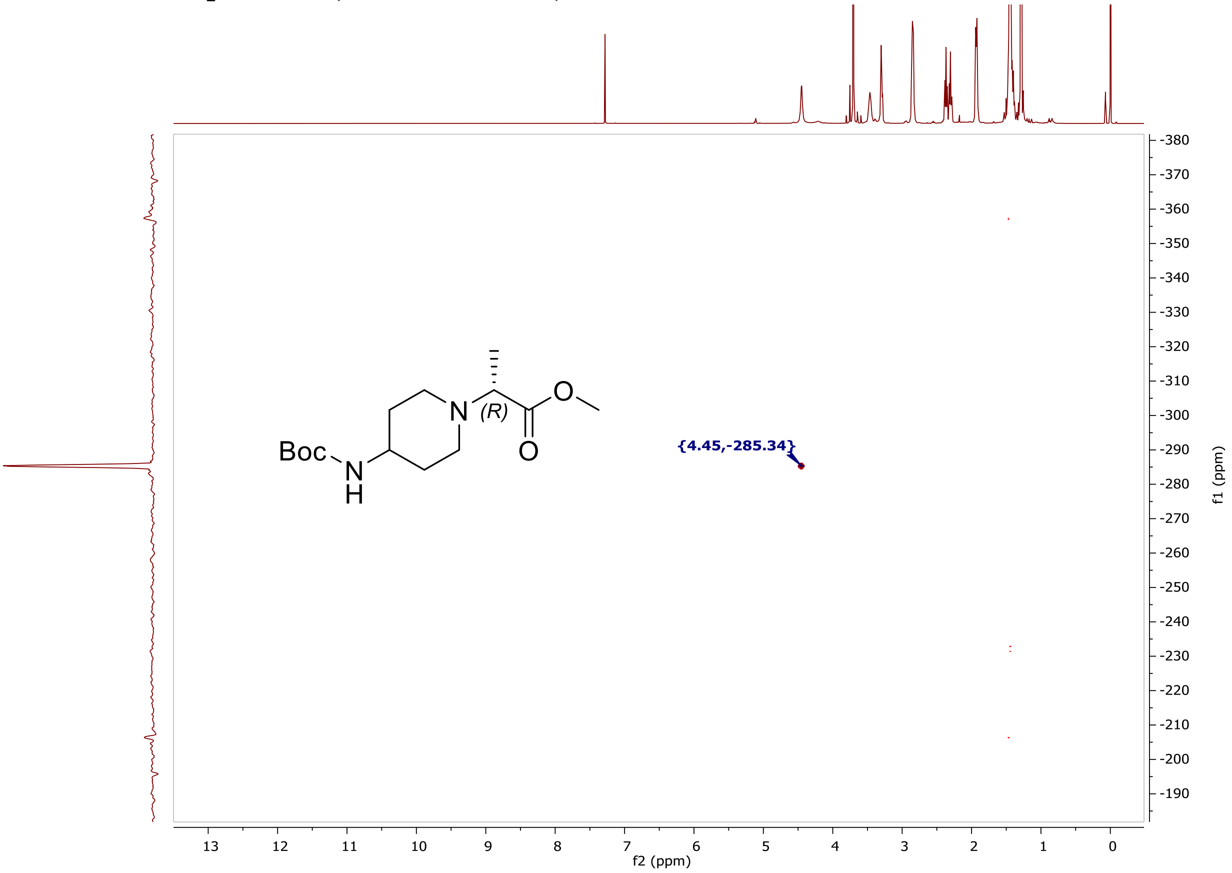


**Figure S12. Methyl (2*R*)-2-{4-[(*tert*-butoxycarbonyl)amino]piperidin-1-yl}propanoate ((*R*)-3a). $^{1}H$-$^{15}N$ HSQC spectrum (71 MHz, $CDCl_3$).**

# Mass Spectrum SmartFormula Report

**Analysis Info**

| | | | |
|---|---|---|---|
| Analysis Name | D:\Data\Organikai\2023_04_11\GMP_371_1-B,2_01_10215.d | Acquisition Date | 4/14/2023 4:05:49 PM |
| Method | organikai_esi_pos_2013_recover.m | Operator | Milda Pukalskiene |
| Sample Name | GMP_371 | Instrument / Ser# | maXis 4G 20218 |
| Comment | | | |

**Acquisition Parameter**

| | | | | | |
|---|---|---|---|---|---|
| Source Type | ESI | Ion Polarity | Positive | Set Nebulizer | 1.5 Bar |
| Focus | Not active | Set Capillary | 4500 V | Set Dry Heater | 180 °C |
| Scan Begin | 40 m/z | Set End Plate Offset | -500 V | Set Dry Gas | 8.0 l/min |
| Scan End | 1800 m/z | Set Collision Cell RF | 350.0 Vpp | Set Divert Valve | Waste |

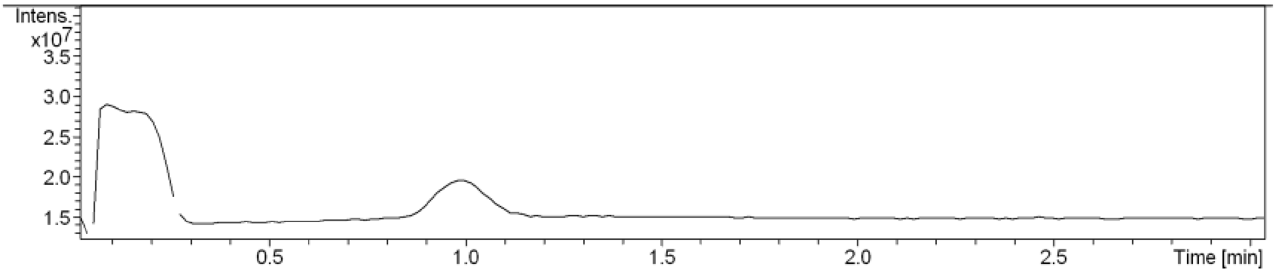


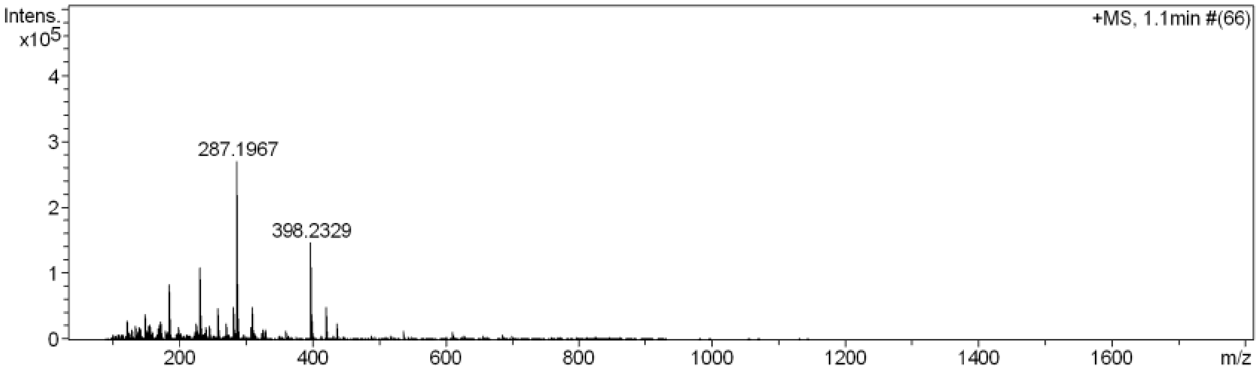


| Meas. m/z | # | Formula | Score | m/z | err [ppm] | Mean err [ppm] | mSigma | rdb | e⁻ Conf | N-Rule |
|---|---|---|---|---|---|---|---|---|---|---|
| 287.1967 | 1 | C 14 H 27 N 2 O 4 | 100.00 | 287.1965 | -0.6 | -0.4 | 12.2 | 2.5 | even | ok |

**Figure S13. Methyl (2*R*)-2-{4-[(*tert*-butoxycarbonyl)amino]piperidin-1-yl}propanoate ((*R*)-3a). HRMS (ESI-TOF).**

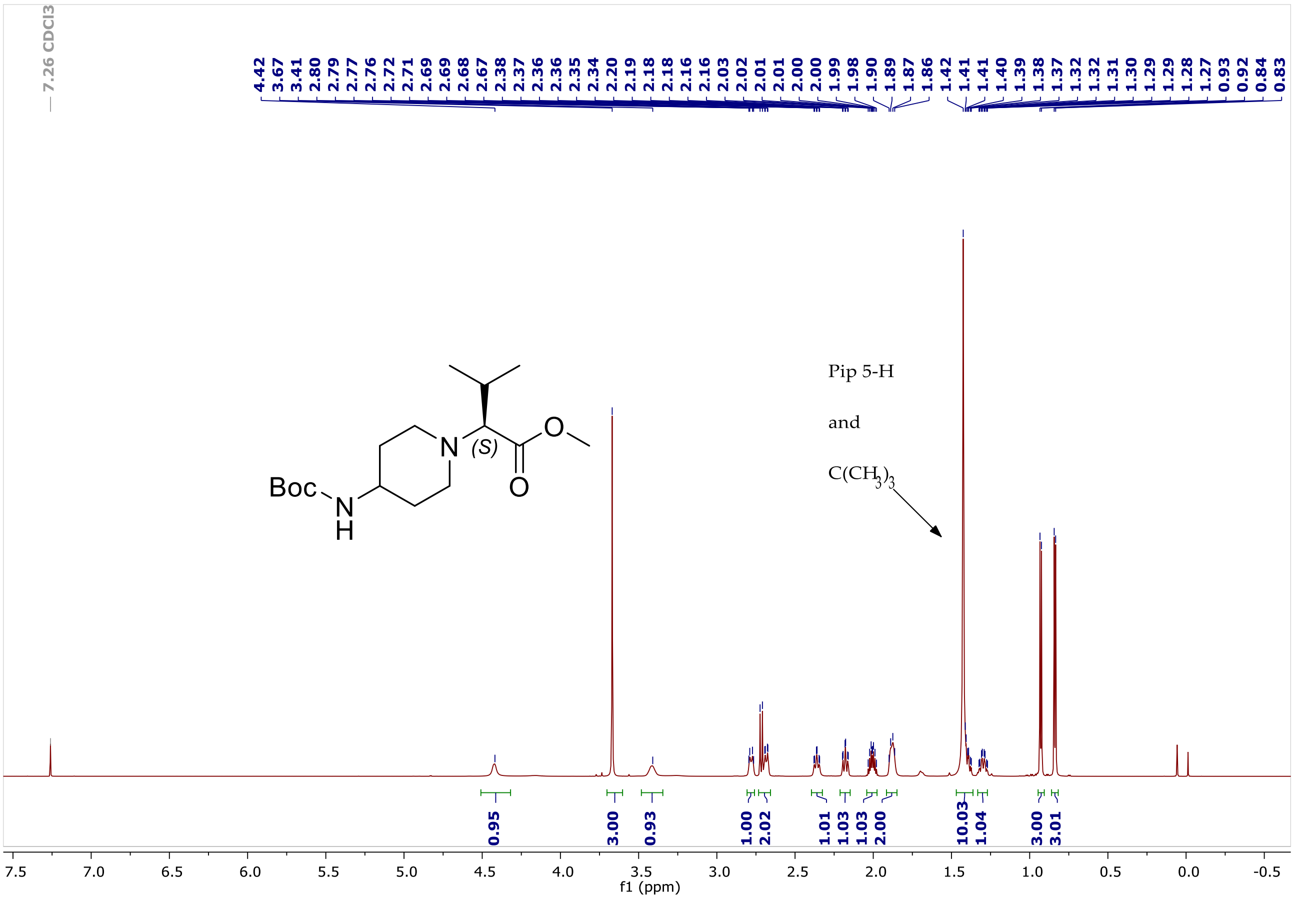


**Figure S14. Methyl (2*S*)-2-{4-[(*tert*-butoxycarbonyl)amino]piperidin-1-yl}-3-methylbutanoate ((*S*)-3b). $^{1}$H NMR spectrum (700 MHz, $CDCl_3$).**

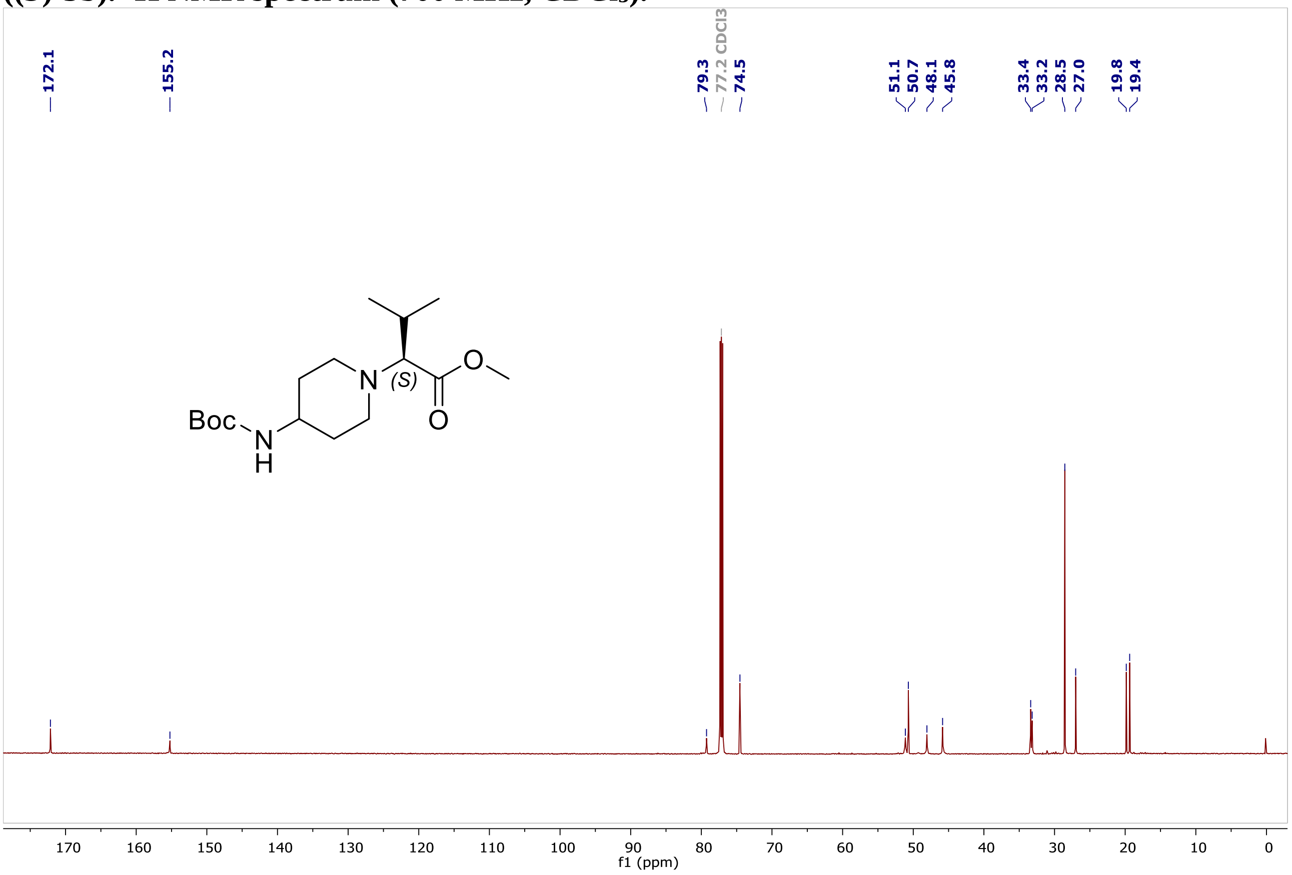


**Figure S15. Methyl (2*S*)-2-{4-[(*tert*-butoxycarbonyl)amino]piperidin-1-yl}-3-methylbutanoate ((*S*)-3b). $^{13}$C NMR spectrum (176 MHz, $CDCl_3$).**

# Mass Spectrum SmartFormula Report

**Analysis Info**

| | | | |
|---|---|---|---|
| Analysis Name | D:\Data\Organikai\2023_04_11\GMP_575_1-B,5_01_10218.d | Acquisition Date | 4/14/2023 4:19:15 PM |
| Method | organikai_esi_pos_2013_recover.m | Operator | Milda Pukalskiene |
| Sample Name | GMP_575 | Instrument / Ser# | maXis 4G 20218 |
| Comment | | | |

**Acquisition Parameter**

| | | | | | |
|---|---|---|---|---|---|
| Source Type | ESI | Ion Polarity | Positive | Set Nebulizer | 1.5 Bar |
| Focus | Not active | Set Capillary | 4500 V | Set Dry Heater | 180 °C |
| Scan Begin | 40 m/z | Set End Plate Offset | -500 V | Set Dry Gas | 8.0 l/min |
| Scan End | 1800 m/z | Set Collision Cell RF | 350.0 Vpp | Set Divert Valve | Waste |

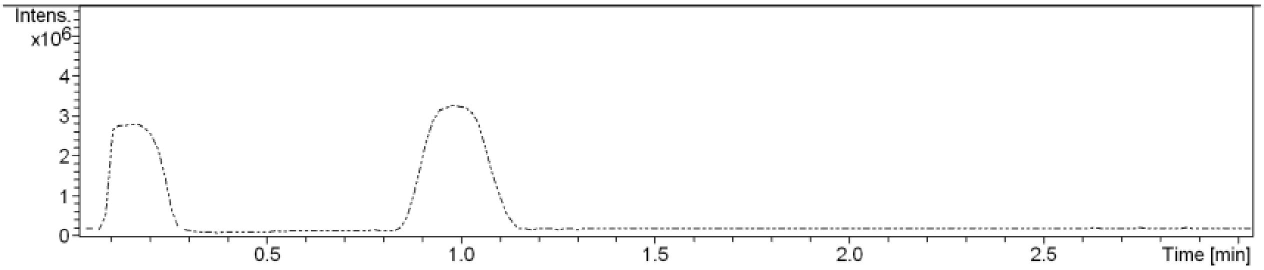


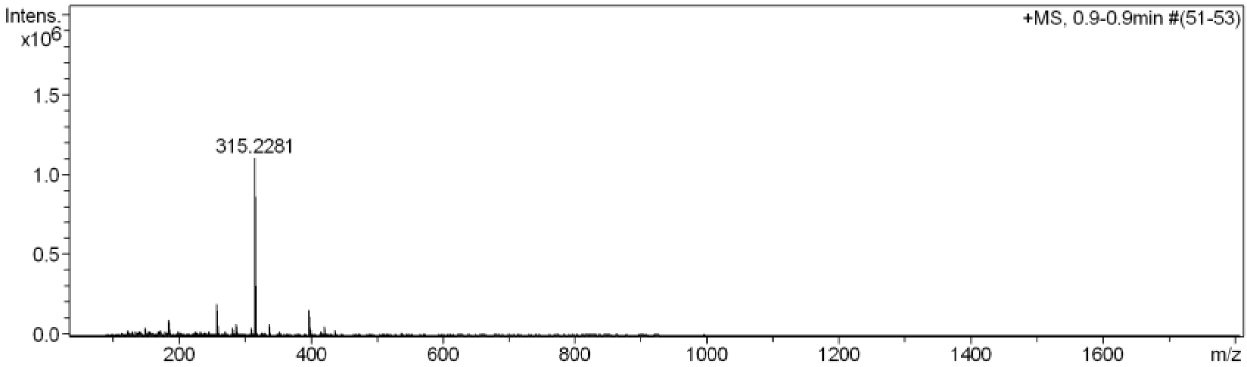


| Meas. m/z | # | Formula | Score | m/z | err [ppm] | Mean err [ppm] | mSigma | rdb | e⁻ Conf | N-Rule |
|---|---|---|---|---|---|---|---|---|---|---|
| 315.2281 | 1 | C 16 H 31 N 2 O 4 | 100.00 | 315.2278 | -0.9 | -0.7 | 16.9 | 2.5 | even | ok |
| | 2 | C 17 H 27 N 6 | 51.82 | 315.2292 | 3.3 | 3.5 | 29.2 | 7.5 | even | ok |

**Figure S16. Methyl (2*S*)-2-{4-[(*tert*-butoxycarbonyl)amino]piperidin-1-yl}-3-methylbutanoate ((*S*)-3b). HRMS (ESI-TOF).**

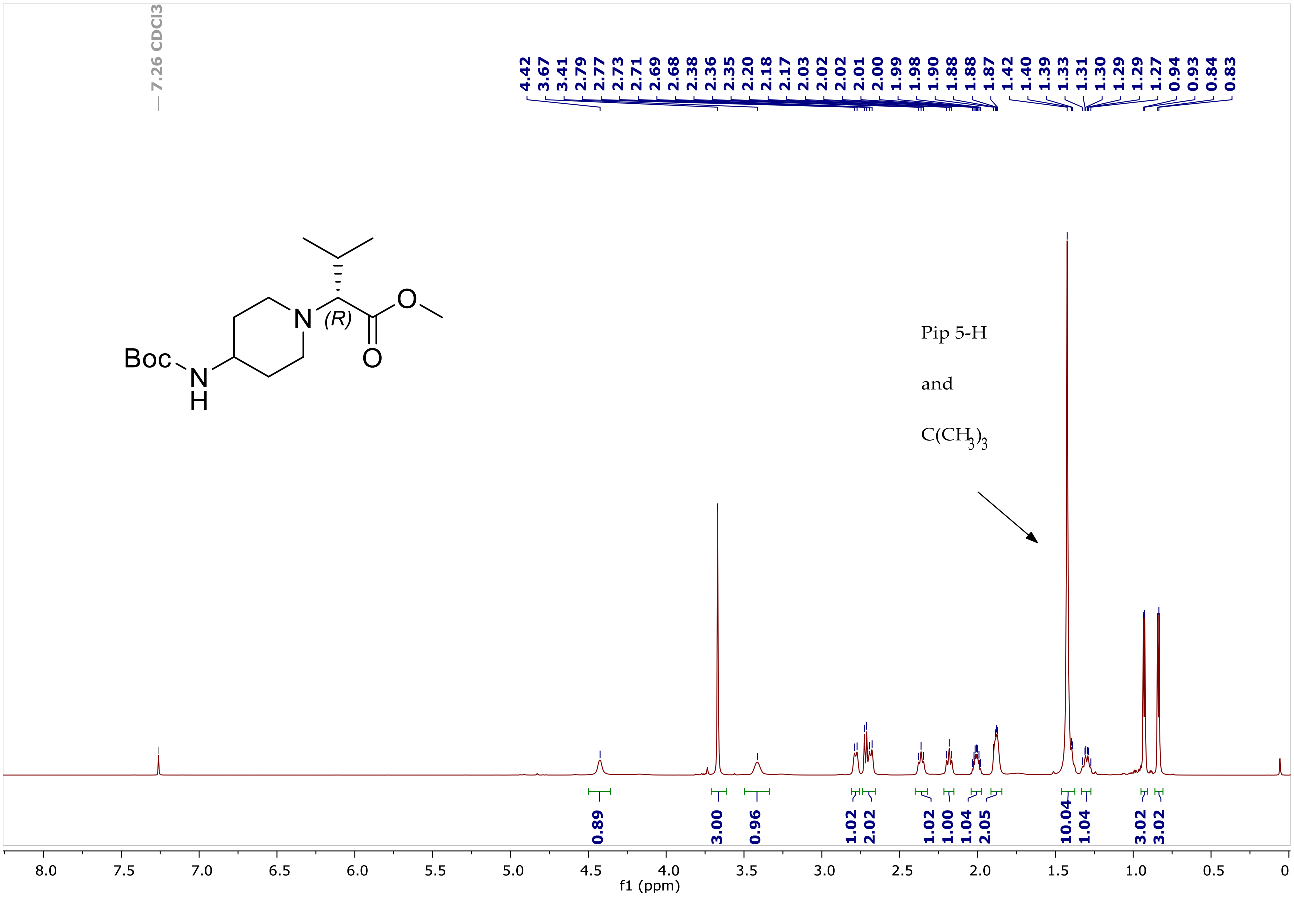


**Figure S17. Methyl (2*R*)-2-{4-[(*tert*-butoxycarbonyl)amino]piperidin-1-yl}-3-methylbutanoate ((*R*)-3b). $^{1}$H NMR spectrum (700 MHz, CDCl₃).**

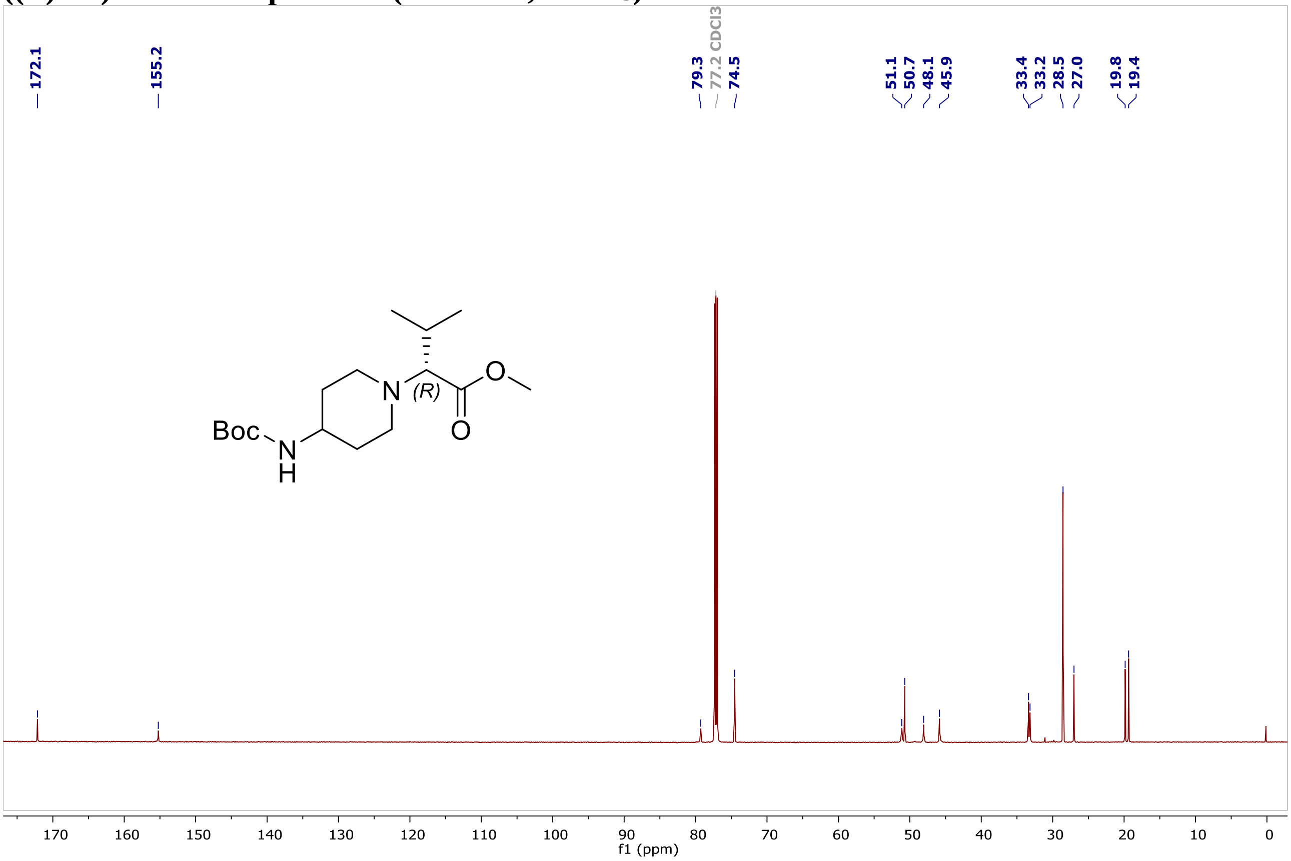


**Figure S18. Methyl (2*R*)-2-{4-[(*tert*-butoxycarbonyl)amino]piperidin-1-yl}-3-methylbutanoate ((*R*)-3b). $^{13}$C NMR spectrum (176 MHz, CDCl₃).**

# Mass Spectrum SmartFormula Report

**Analysis Info**

| | | | |
|---|---|---|---|
| Analysis Name | D:\Data\Organikai\2023_04_11\GMP_571_1-B,4_01_10217.d | Acquisition Date | 4/14/2023 4:14:48 PM |
| Method | organikai_esi_pos_2013_recover.m | Operator | Milda Pukalskiene |
| Sample Name | GMP_571 | Instrument / Ser# | maXis 4G 20218 |
| Comment | | | |

**Acquisition Parameter**

| | | | | | |
|---|---|---|---|---|---|
| Source Type | ESI | Ion Polarity | Positive | Set Nebulizer | 1.5 Bar |
| Focus | Not active | Set Capillary | 4500 V | Set Dry Heater | 180 °C |
| Scan Begin | 40 m/z | Set End Plate Offset | -500 V | Set Dry Gas | 8.0 l/min |
| Scan End | 1800 m/z | Set Collision Cell RF | 350.0 Vpp | Set Divert Valve | Waste |

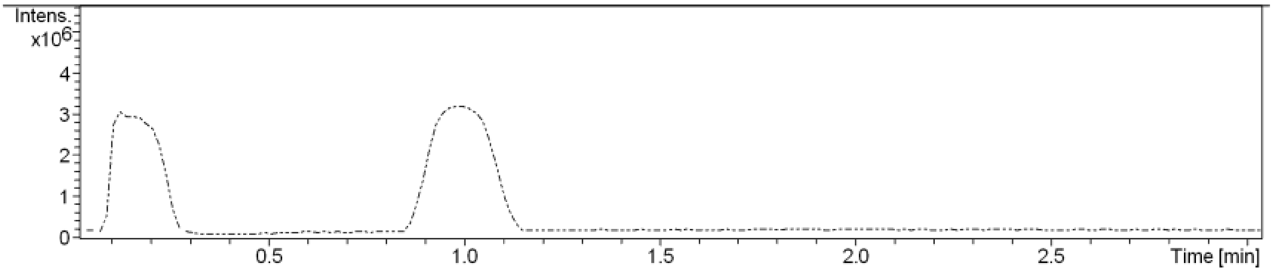


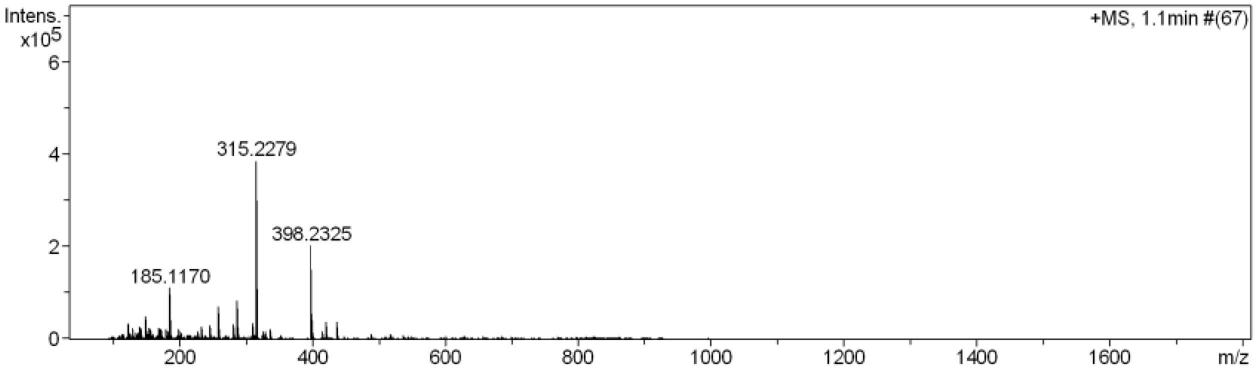


| Meas. m/z | # | Formula | Score | m/z | err [ppm] | Mean err [ppm] | mSigma | rdb | e⁻ Conf | N-Rule |
|---|---|---|---|---|---|---|---|---|---|---|
| 315.2279 | 1 | C 16 H 31 N 2 O 4 | 100.00 | 315.2278 | -0.3 | -0.2 | 18.9 | 2.5 | even | ok |
| | 2 | C 17 H 27 N 6 | 45.53 | 315.2292 | 3.9 | 4.0 | 27.9 | 7.5 | even | ok |

**Figure S19. Methyl (2*R*)-2-{4-[(*tert*-butoxycarbonyl)amino]piperidin-1-yl}-3-methylbutanoate ((*R*)-3b). HRMS (ESI-TOF).**

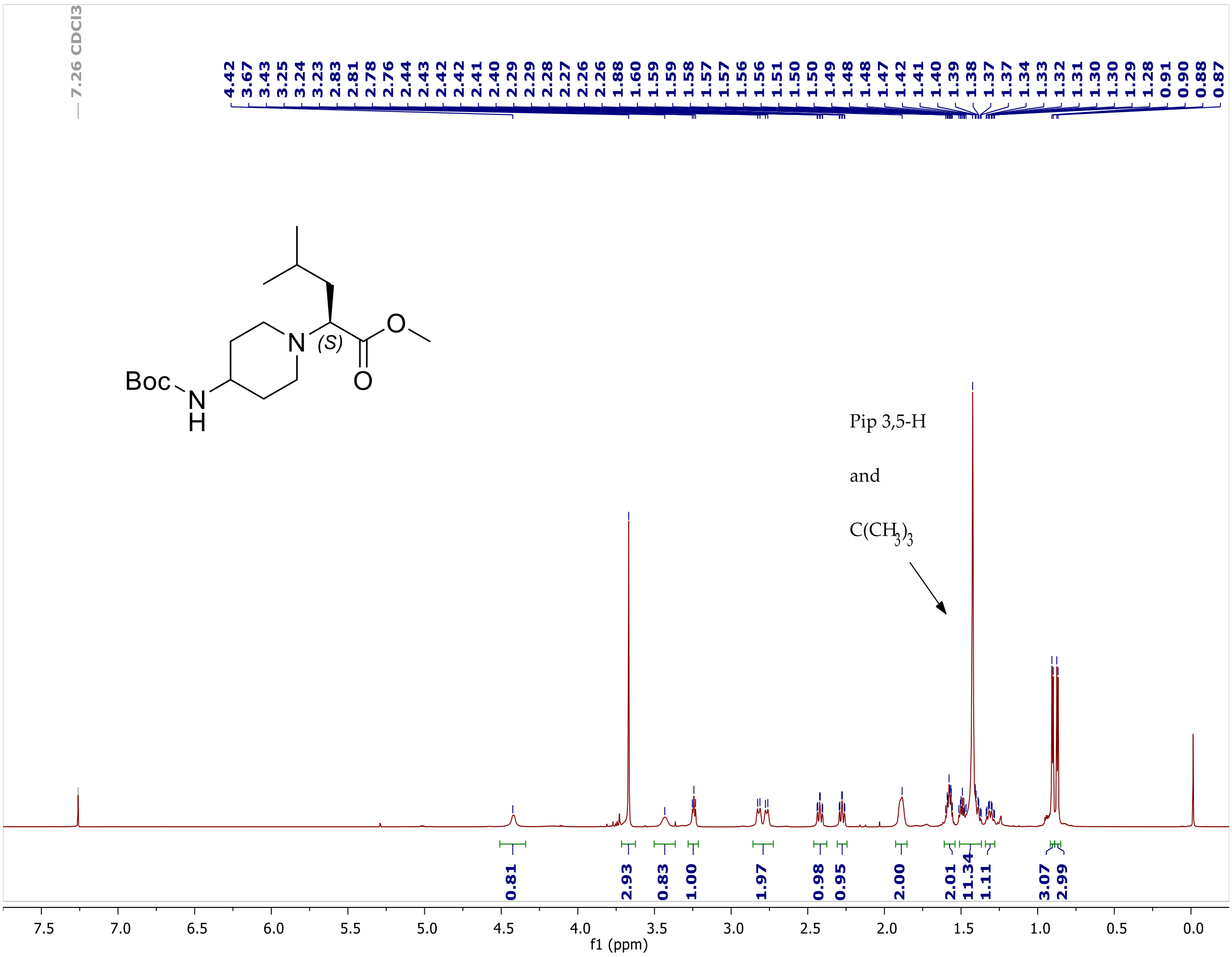


**Figure S20. Methyl (2*S*)-2-{4-[(*tert*-butoxycarbonyl)amino]piperidin-1-yl}-4-methylpentanoate ((*S*)-3c). $^{1}H$ NMR spectrum (700 MHz, $CDCl_3$).**

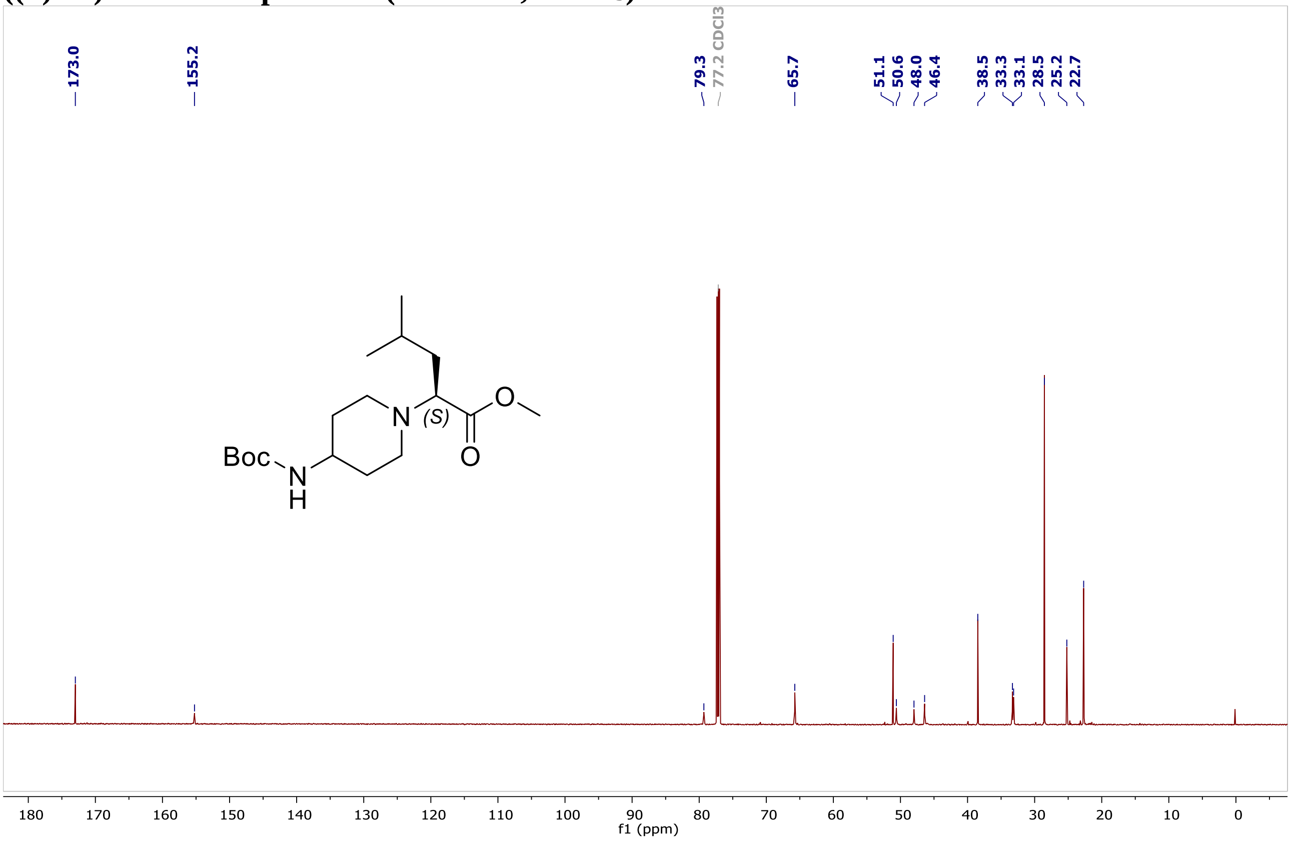


**Figure S21. Methyl (2*S*)-2-{4-[(*tert*-butoxycarbonyl)amino]piperidin-1-yl}-4-methylpentanoate ((*S*)-3c). $^{13}C$ NMR spectrum (176 MHz, $CDCl_3$).**

# Mass Spectrum SmartFormula Report

**Analysis Info**

| | | | |
|---|---|---|---|
| Analysis Name | D:\Data\Organikai\2023_04_11\GMP_602_1-B,7_01_10220.d | Acquisition Date | 4/14/2023 4:28:12 PM |
| Method | organikai_esi_pos_2013_recover.m | Operator | Milda Pukalskiene |
| Sample Name | GMP_602 | Instrument / Ser# | maXis 4G 20218 |
| Comment | | | |

**Acquisition Parameter**

| | | | | | |
|---|---|---|---|---|---|
| Source Type | ESI | Ion Polarity | Positive | Set Nebulizer | 1.5 Bar |
| Focus | Not active | Set Capillary | 4500 V | Set Dry Heater | 180 °C |
| Scan Begin | 40 m/z | Set End Plate Offset | -500 V | Set Dry Gas | 8.0 l/min |
| Scan End | 1800 m/z | Set Collision Cell RF | 350.0 Vpp | Set Divert Valve | Waste |

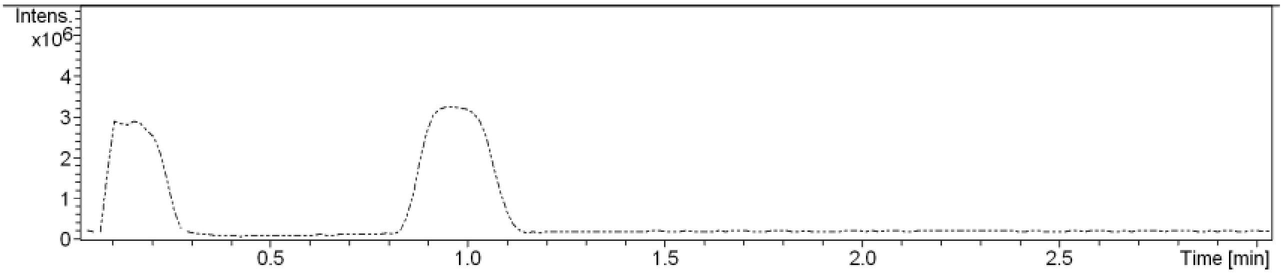


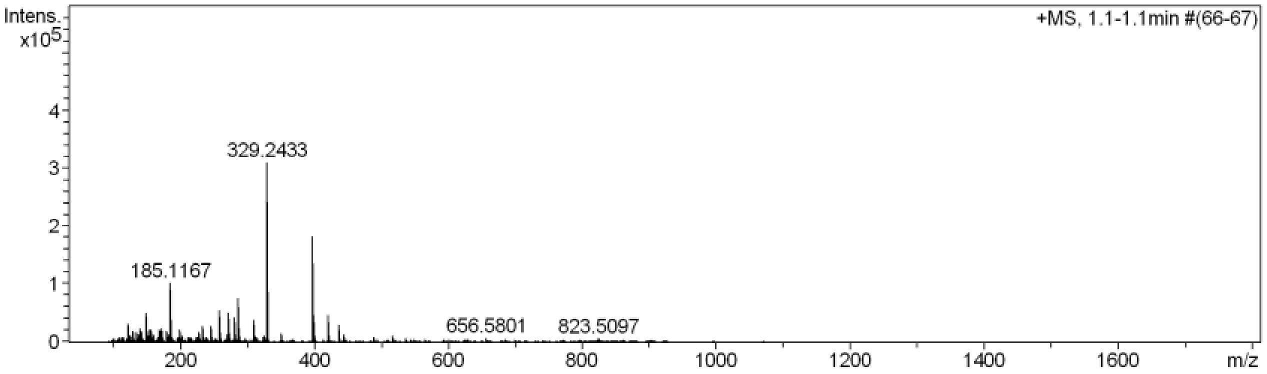


| Meas. m/z | # | Formula | Score | m/z | err [ppm] | Mean err [ppm] | mSigma | rdb | e⁻ Conf | N-Rule |
|---|---|---|---|---|---|---|---|---|---|---|
| 329.2433 | 1 | C 17 H 33 N 2 O 4 | 100.00 | 329.2435 | 0.6 | 0.9 | 16.7 | 2.5 | even | ok |
| | 2 | C 18 H 29 N 6 | 36.31 | 329.2448 | 4.7 | 4.9 | 29.3 | 7.5 | even | ok |

**Figure S22. Methyl (2*S*)-2-{4-[(*tert*-butoxycarbonyl)amino]piperidin-1-yl}-4-methylpentanoate ((*S*)-3c). HRMS (ESI-TOF).**

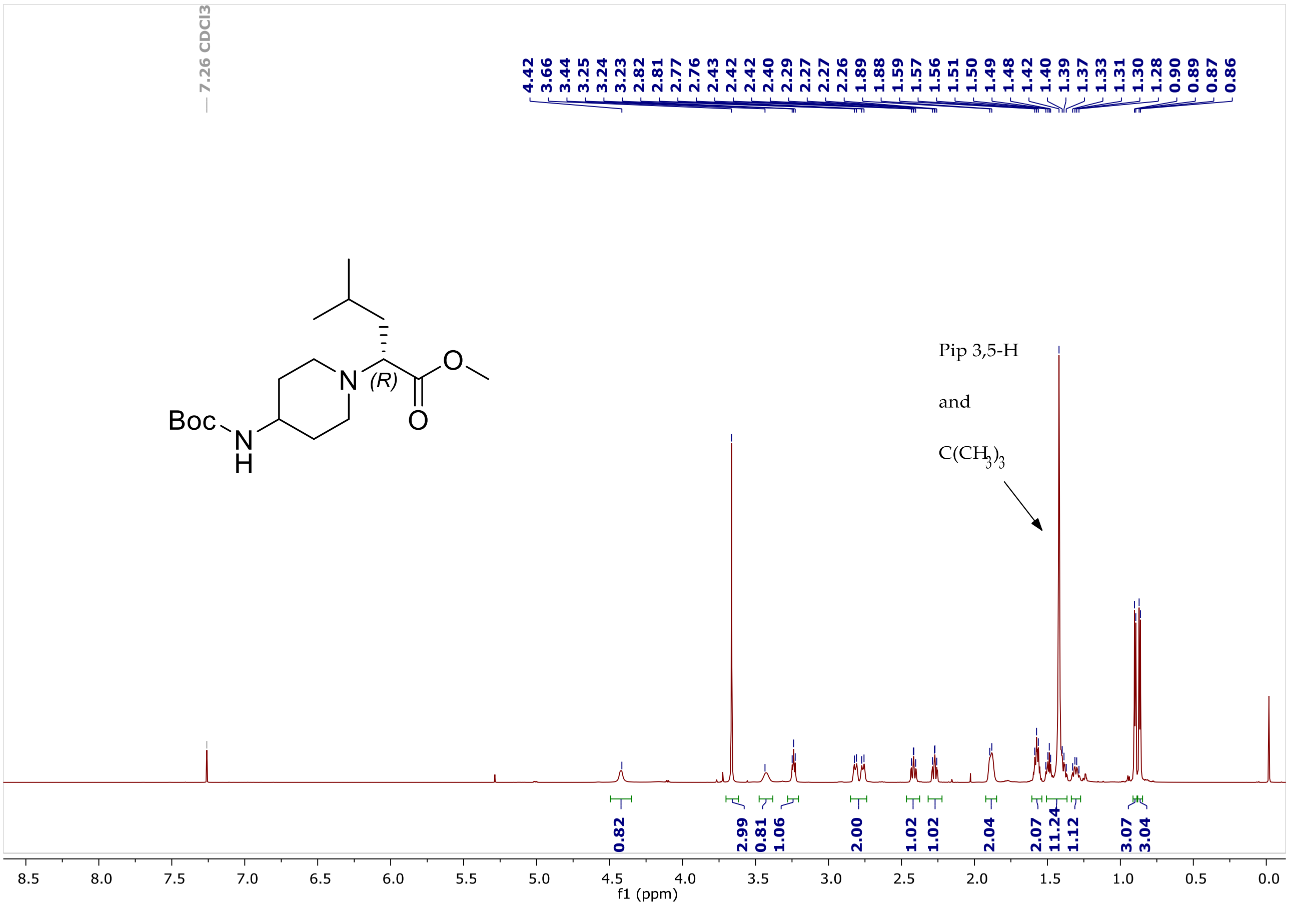


**Figure S23. Methyl (2*R*)-2-{4-[(*tert*-butoxycarbonyl)amino]piperidin-1-yl}-4-methylpentanoate ((*R*)-3c). $^{1}$H NMR spectrum (700 MHz, $CDCl_3$).**

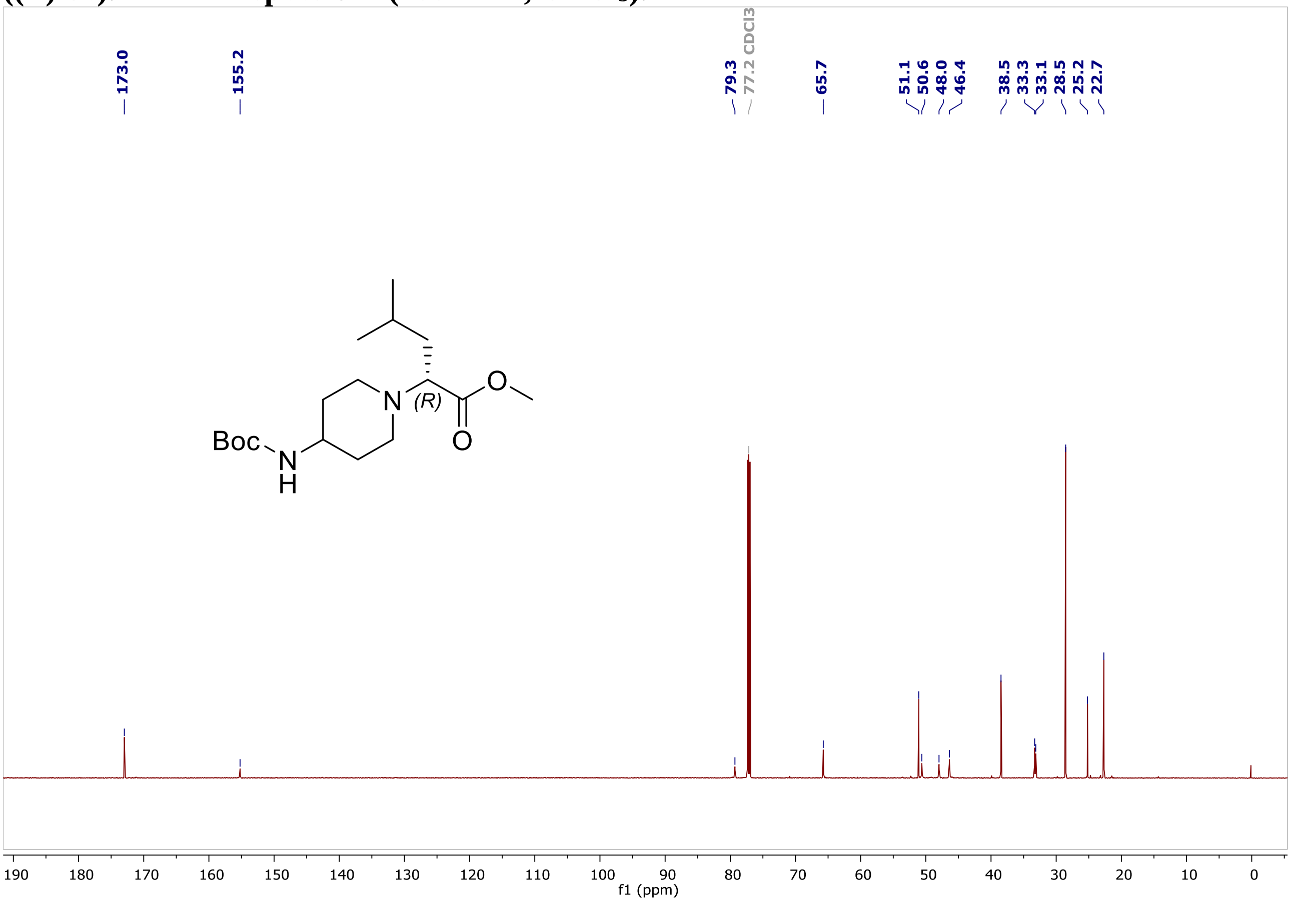


**Figure S24. Methyl (2*R*)-2-{4-[(*tert*-butoxycarbonyl)amino]piperidin-1-yl}-4-methylpentanoate ((*R*)-3c). $^{13}$C NMR spectrum (176 MHz, $CDCl_3$).**

# Mass Spectrum SmartFormula Report

**Analysis Info**

| | | | |
|---|---|---|---|
| Analysis Name | D:\Data\Organikai\2023_04_11\GMP_610_1-B,6_01_10219.d | Acquisition Date | 4/14/2023 4:23:42 PM |
| Method | organikai_esi_pos_2013_recover.m | Operator | Milda Pukalskiene |
| Sample Name | GMP_610 | Instrument / Ser# | maXis 4G 20218 |
| Comment | | | |

**Acquisition Parameter**

| | | | | | |
|---|---|---|---|---|---|
| Source Type | ESI | Ion Polarity | Positive | Set Nebulizer | 1.5 Bar |
| Focus | Not active | Set Capillary | 4500 V | Set Dry Heater | 180 °C |
| Scan Begin | 40 m/z | Set End Plate Offset | -500 V | Set Dry Gas | 8.0 l/min |
| Scan End | 1800 m/z | Set Collision Cell RF | 350.0 Vpp | Set Divert Valve | Waste |

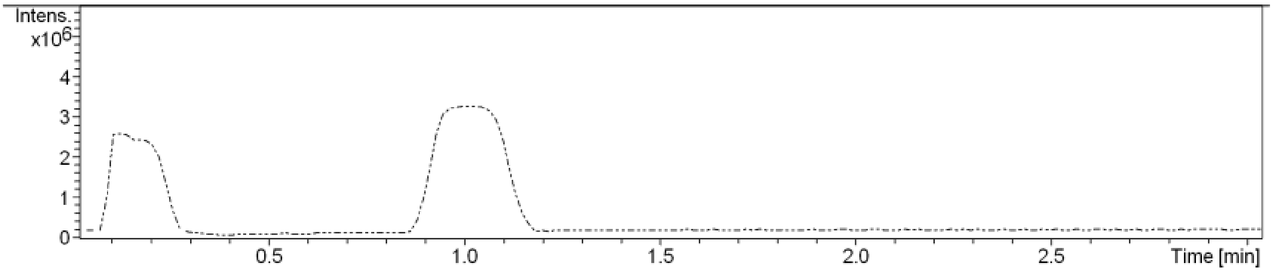


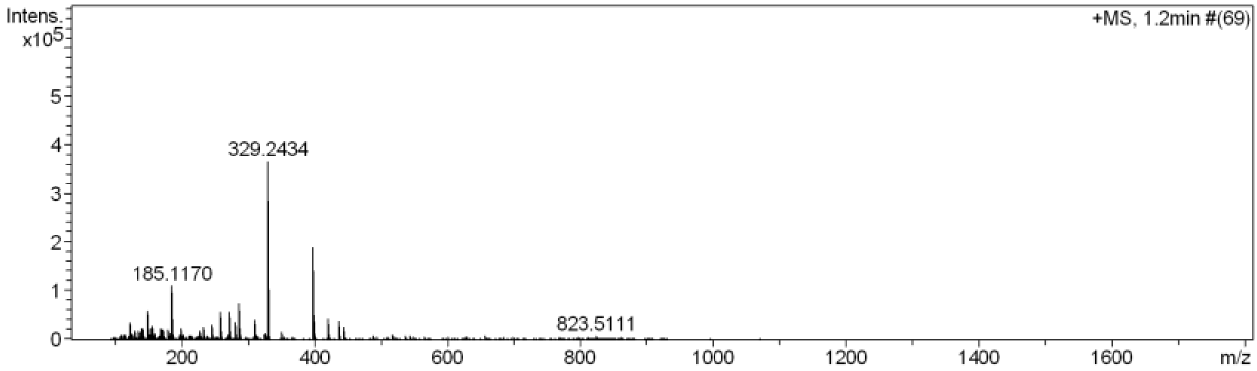


| Meas. m/z | # | Formula | Score | m/z | err [ppm] | Mean err [ppm] | mSigma | rdb | e⁻ Conf | N-Rule |
|---|---|---|---|---|---|---|---|---|---|---|
| 329.2434 | 1 | C 17 H 33 N 2 O 4 | 100.00 | 329.2435 | 0.4 | 0.7 | 15.1 | 2.5 | even | ok |
| | 2 | C 18 H 29 N 6 | 37.00 | 329.2448 | 4.5 | 4.7 | 27.8 | 7.5 | even | ok |

**Figure S25. Methyl (2*R*)-2-{4-[(*tert*-butoxycarbonyl)amino]piperidin-1-yl}-4-methylpentanoate ((*R*)-3c). HRMS (ESI-TOF).**

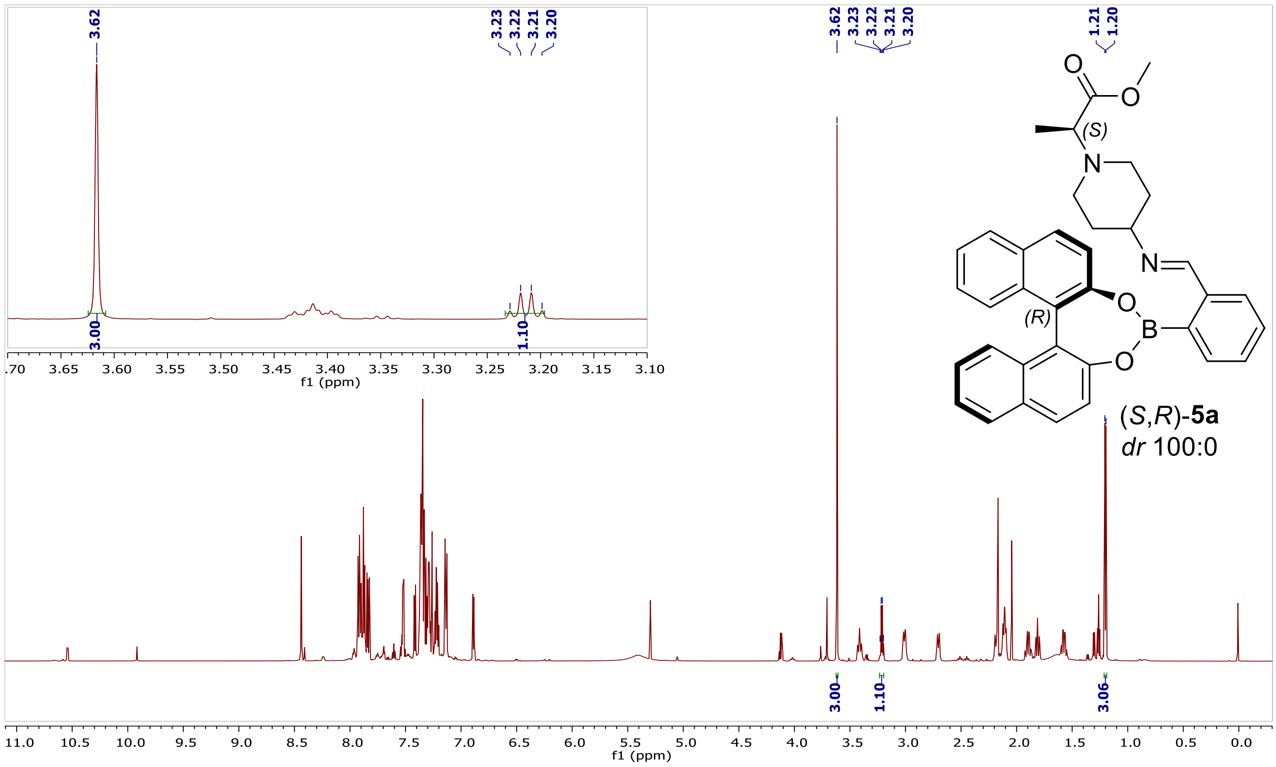


**Figure S26. Crude sample from reaction mixture of iminoboronate ester complex ((*S*,*R*)-5a). $^{1}$H NMR spectrum (700 MHz, $CDCl_3$).**

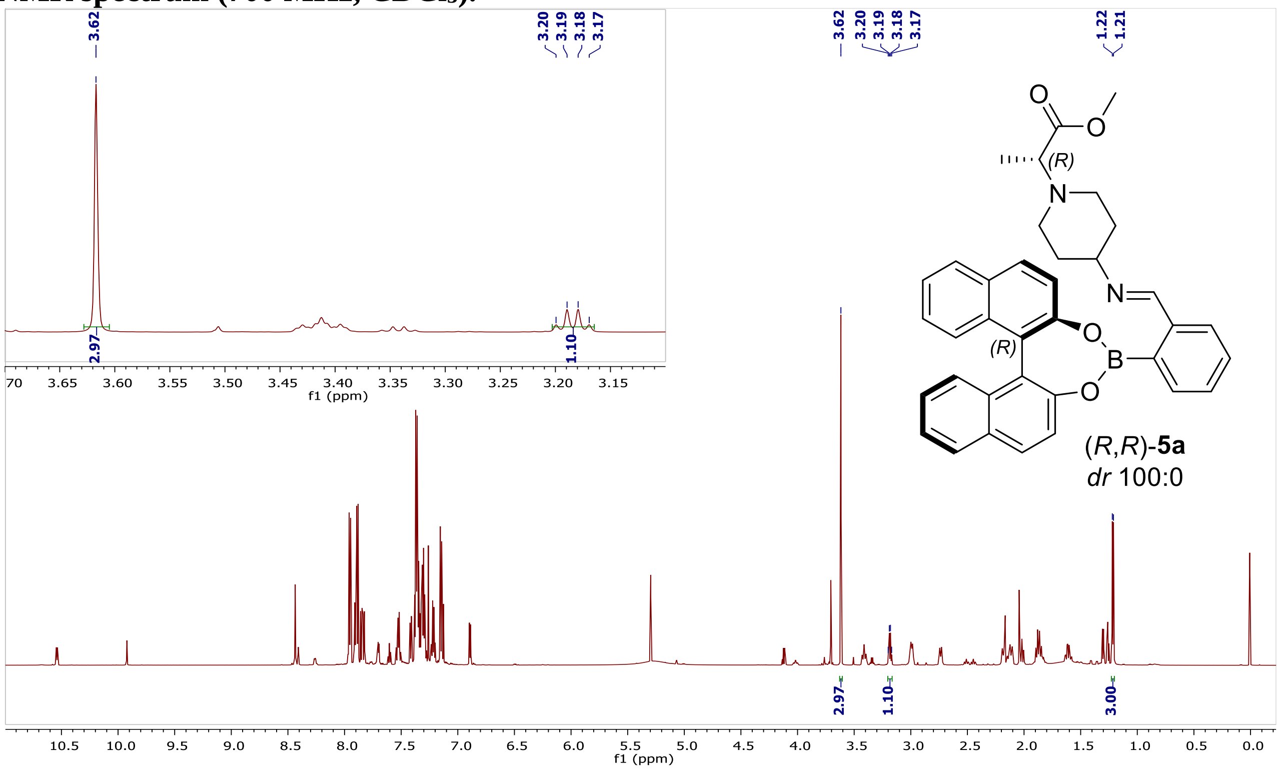


**Figure S27. Crude sample from reaction mixture of iminoboronate ester complex ((*R*,*R*)-5a). $^{1}$H NMR spectrum (700 MHz, $CDCl_3$).**

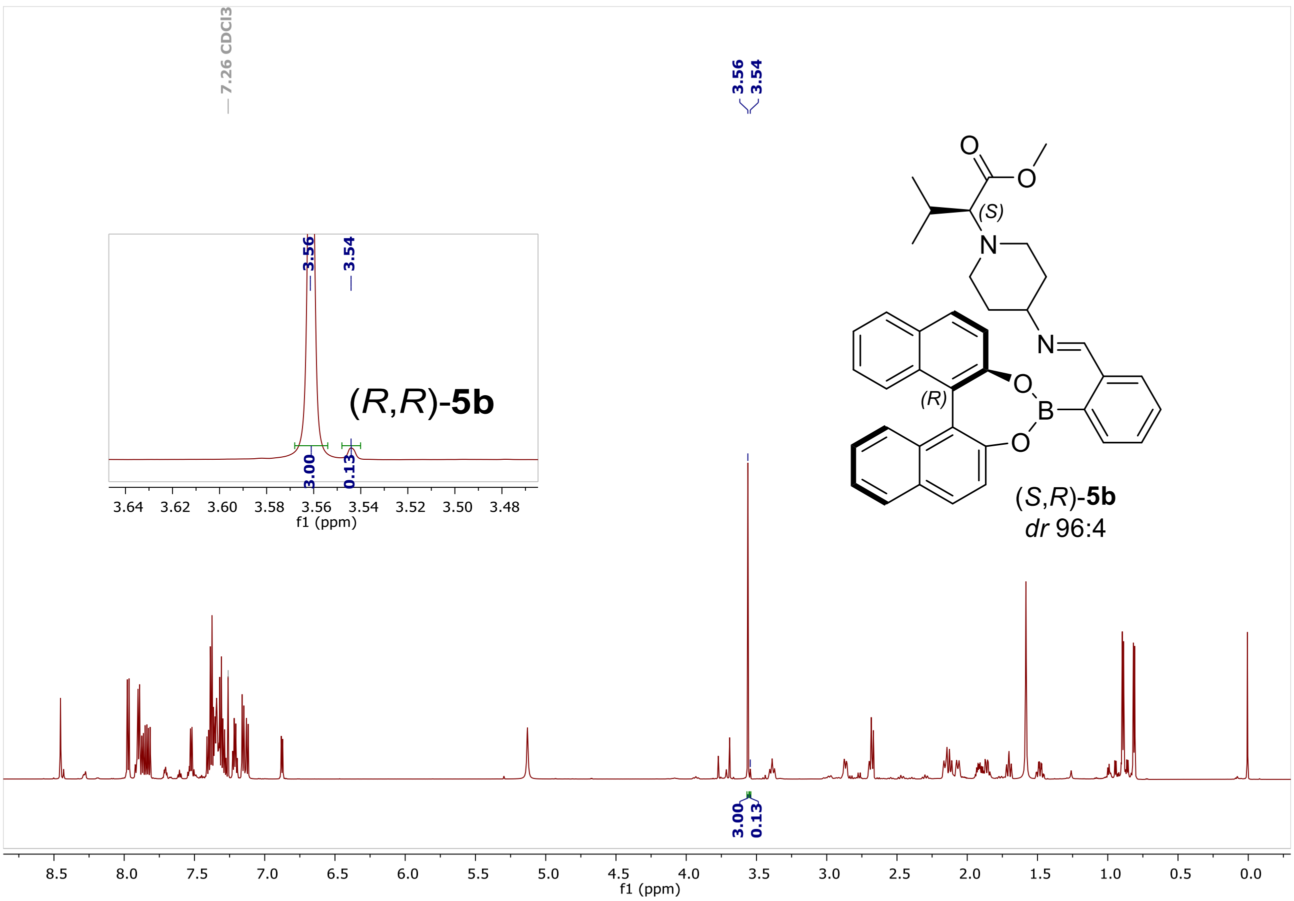


**Figure S28. Crude sample from reaction mixture of iminoboronate ester complex ((*S*,*R*)-5b). $^{1}$H NMR spectrum (700 MHz, CDCl$_3$).**

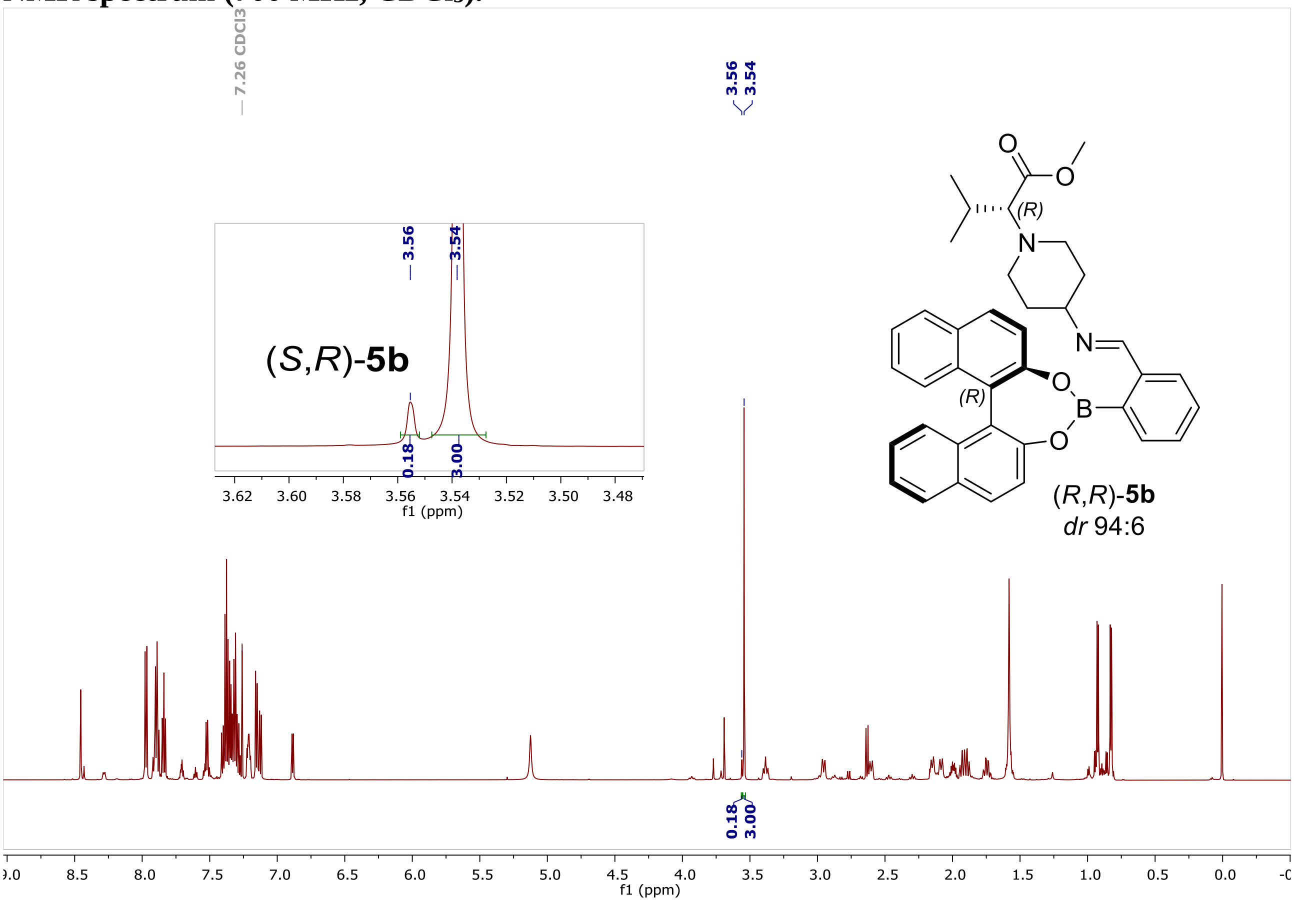


**Figure S29. Crude sample from reaction mixture of iminoboronate ester complex ((*R*,*R*)-5b). $^{1}$H NMR spectrum (700 MHz, CDCl$_3$).**

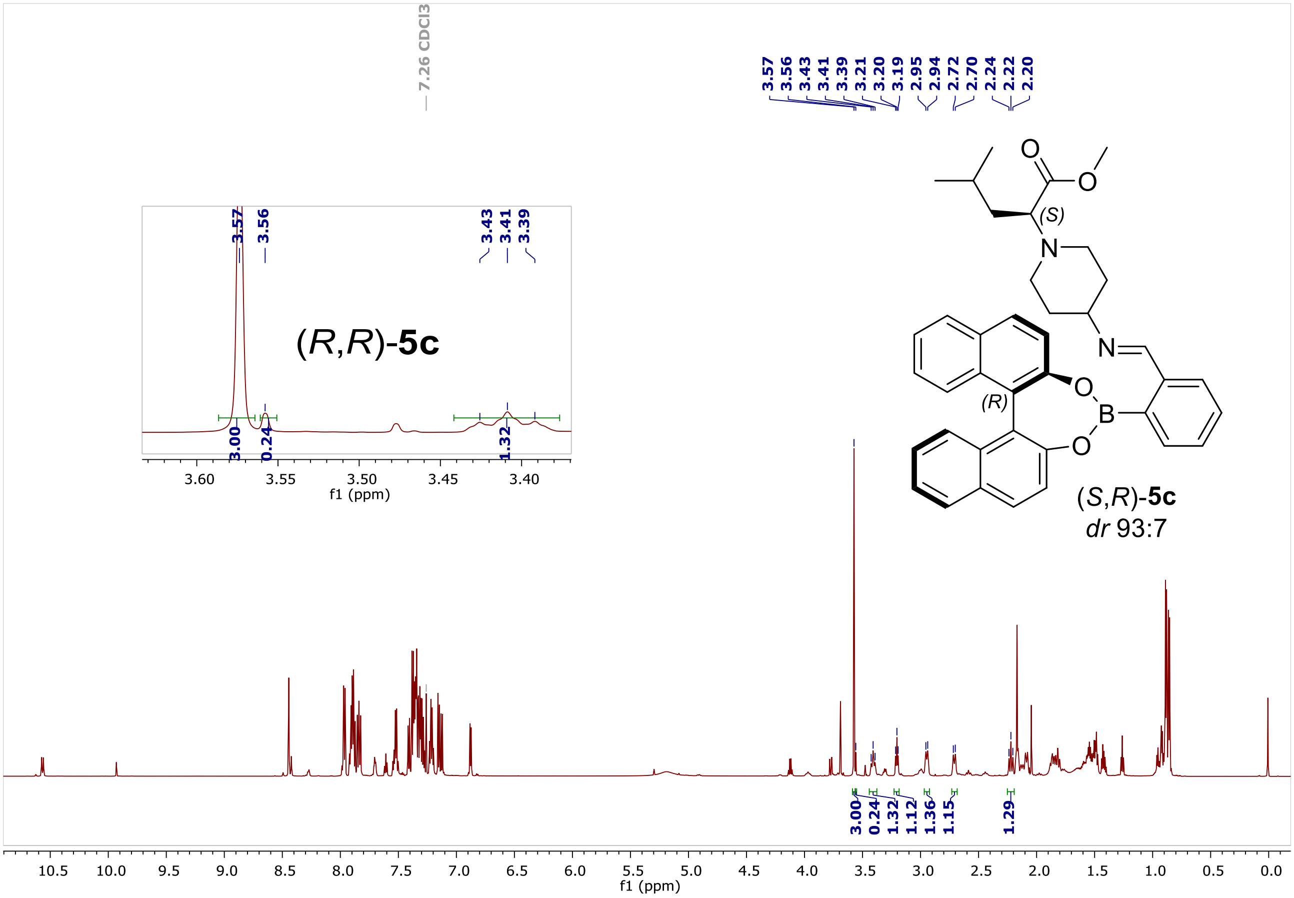


**Figure S30. Crude sample from reaction mixture of iminoboronate ester complex ((*S*,*R*)-5c). $^{1}$H NMR spectrum (700 MHz, $CDCl_3$).**

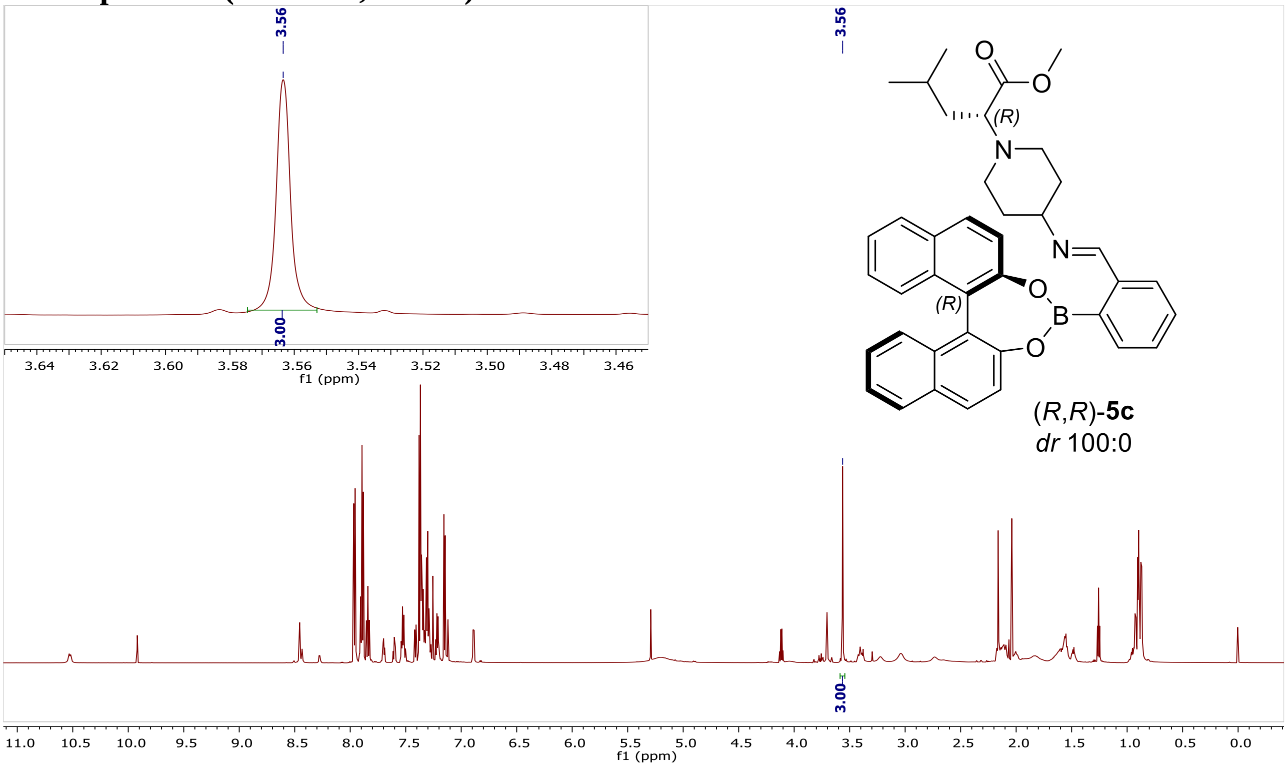


**Figure S31. Crude sample from reaction mixture of iminoboronate ester complex ((*R*,*R*)-5c). $^{1}$H NMR spectrum (700 MHz, $CDCl_3$).**

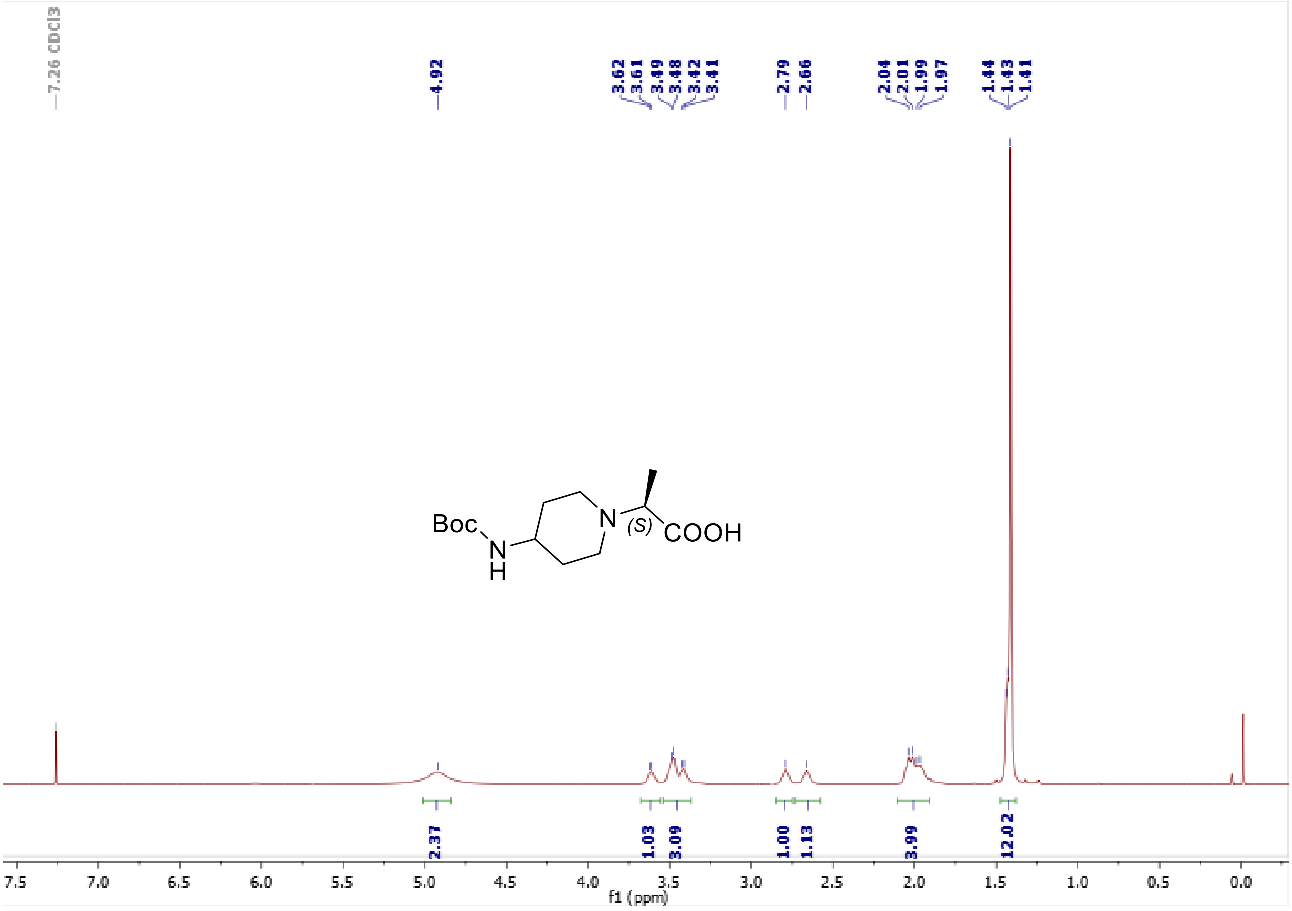


**Figure S32. (2*S*)-2-{4-[(*tert*-butoxycarbonyl)amino]piperidin-1-yl}propanoic acid ((*S*)-6). $^{1}$H NMR spectrum (700 MHz, $CDCl_3$).**

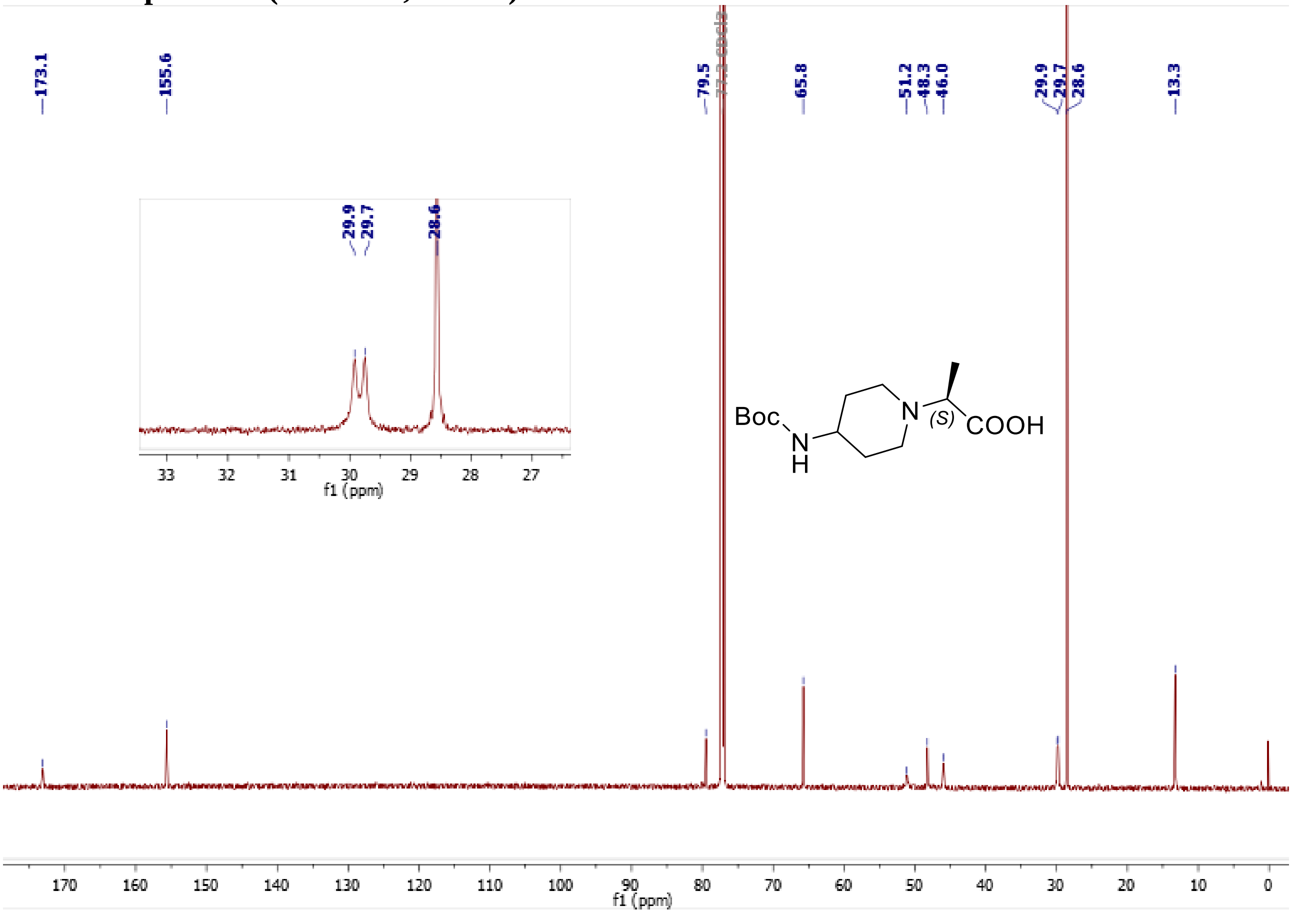


**Figure S33. (2*S*)-2-{4-[(*tert*-butoxycarbonyl)amino]piperidin-1-yl}propanoic acid ((*S*)-6). $^{13}$C NMR spectrum (176 MHz, $CDCl_3$).**

# Compound Spectrum SmartFormula Report

**Analysis Info**

| | | | |
|---|---|---|---|
| Analysis Name | D:\Data\GMP-1188.d | Acquisition Date | 5/2/2023 8:28:21 PM |
| Method | DirectInfusion_TuneLow_pos.m | Operator | hplc |
| Sample Name | GMP-1188 | Instrument | micrOTOF-Q III 8228888.20448 |
| Comment | AB | | |

**Acquisition Parameter**

| | | | | | |
|---|---|---|---|---|---|
| Source Type | ESI | Ion Polarity | Positive | Set Nebulizer | 0.4 Bar |
| Focus | Not active | Set Capillary | 4500 V | Set Dry Heater | 180 °C |
| Scan Begin | 50 m/z | Set End Plate Offset | -500 V | Set Dry Gas | 4.0 l/min |
| Scan End | 1000 m/z | Set Collision Cell RF | 140.0 Vpp | Set Divert Valve | Waste |

| # | RT [min] | Area | Int. Type | I | S/N | Chromatogram | Max. m/z | FWHM [min] |
|---|---|---|---|---|---|---|---|---|
| n.a. | 6.6 | n.a. | Single spectrum | n.a. | n.a. | n.a. | 295.1630 | n.a. |

**+MS, 6.6min #393**

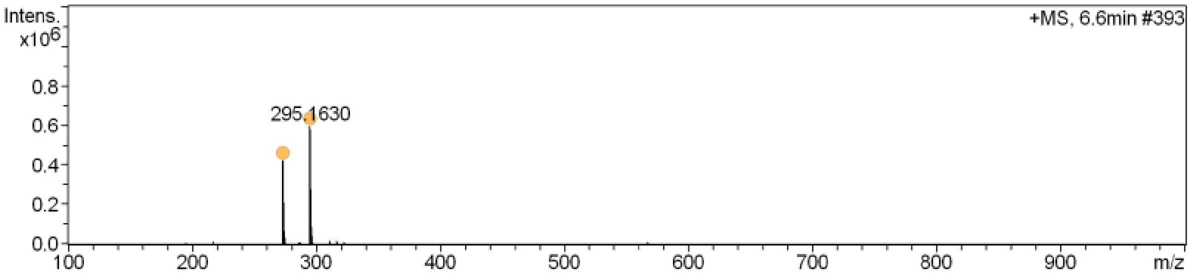


| Meas. m/z | # | Ion Formula | m/z | err [ppm] | mSigma | # Sigma | Score | rdb | e⁻ Conf | N-Rule |
|---|---|---|---|---|---|---|---|---|---|---|
| 273.1805 | 1 | C13H25N2O4 | 273.1809 | -1.4 | 6.9 | 1 | 100.00 | 2.5 | even | ok |
| 295.1630 | 1 | C13H24N2NaO4 | 295.1628 | -0.6 | 2.2 | 1 | 100.00 | 2.5 | even | ok |

**Figure S34. (2*S*)-2-{4-[(*tert*-butoxycarbonyl)amino]piperidin-1-yl}propanoic acid ((*S*)-6). HRMS (ESI-TOF).**

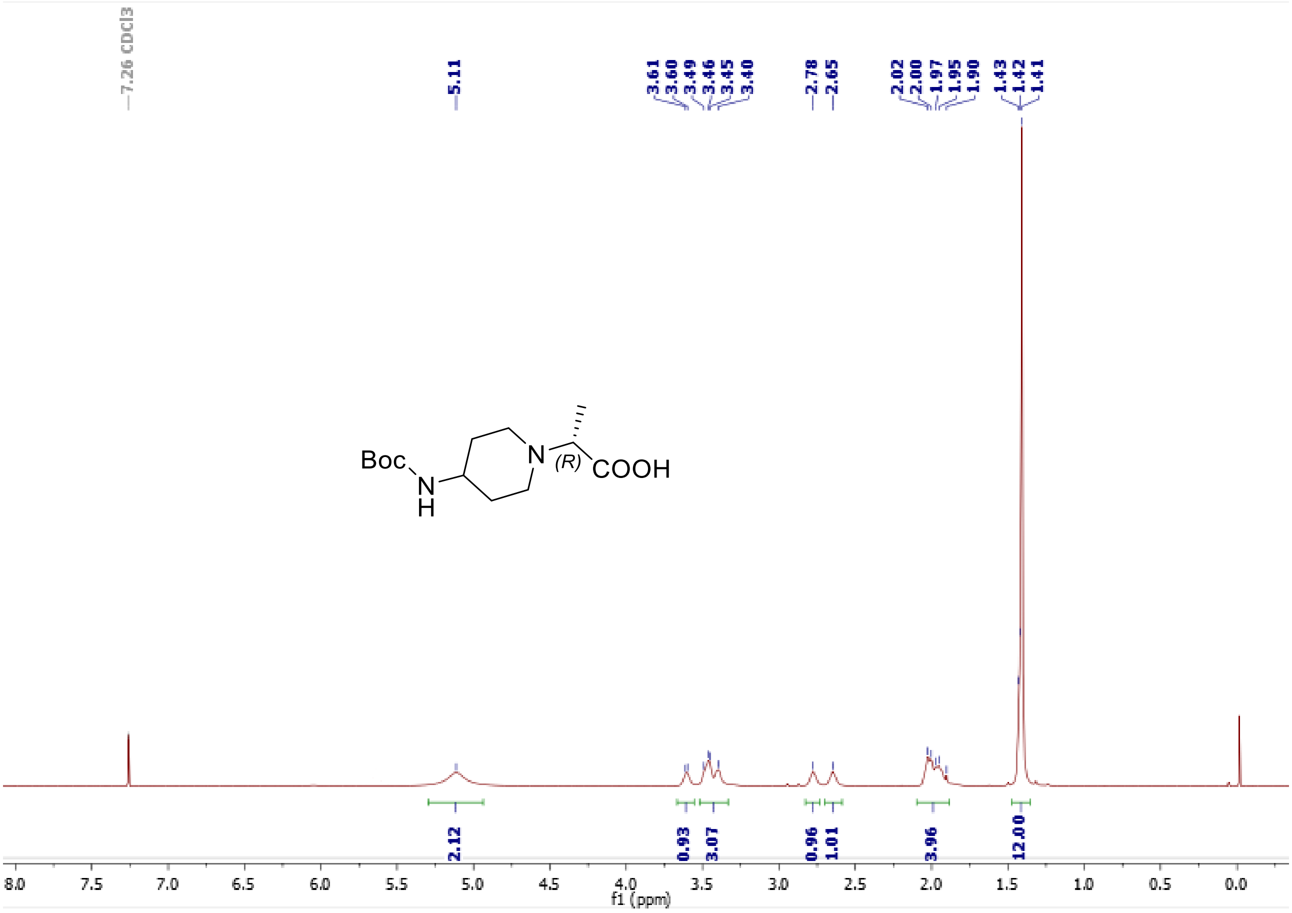


**Figure S35.** (2*R*)-2-{4-[(*tert*-butoxycarbonyl)amino]piperidin-1-yl}propanoic acid ((*R*)-6). $^{1}$H NMR spectrum (700 MHz, $CDCl_3$).

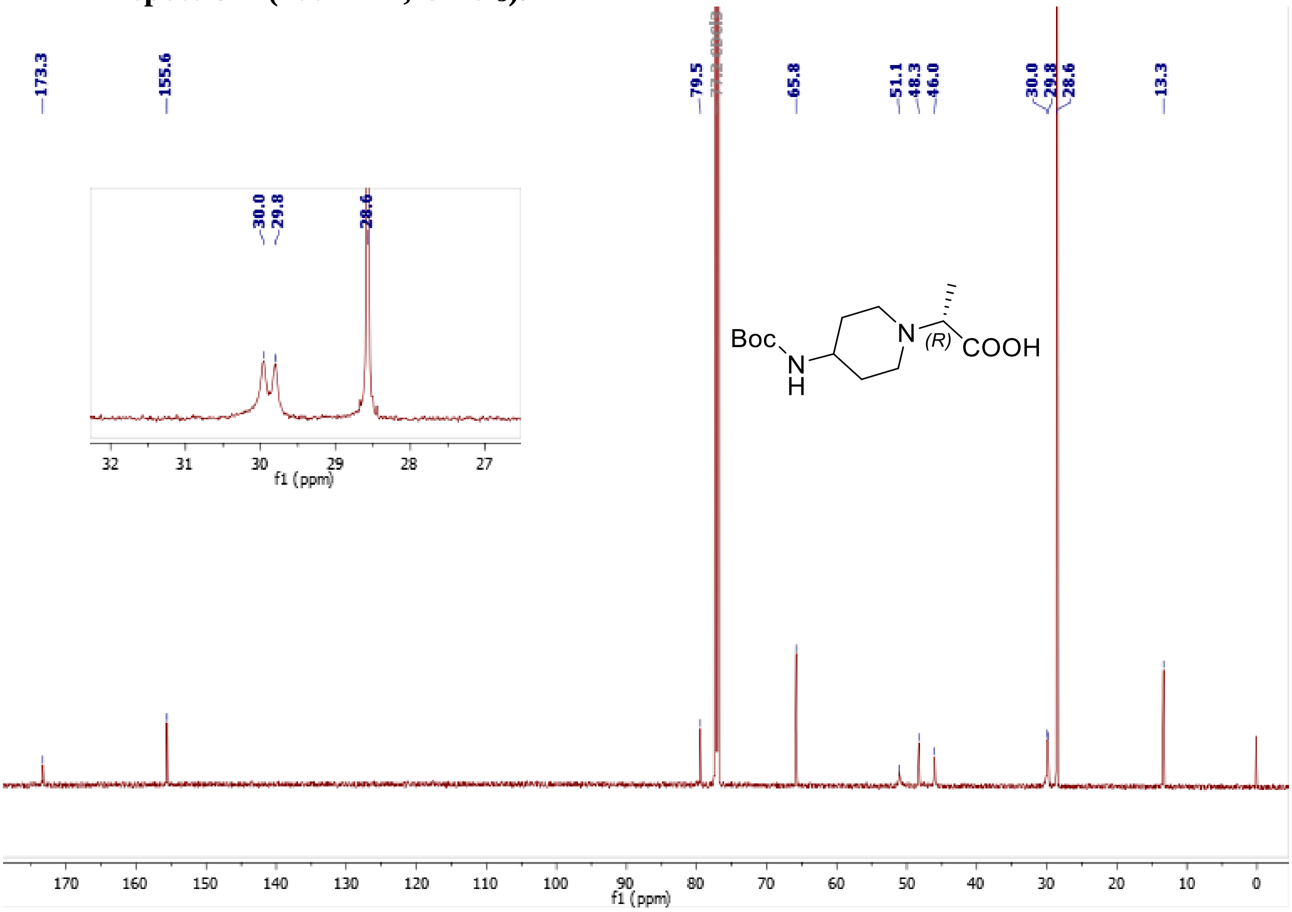


**Figure S36.** (2*R*)-2-{4-[(*tert*-butoxycarbonyl)amino]piperidin-1-yl}propanoic acid ((*R*)-6). $^{13}$C NMR spectrum (176 MHz, $CDCl_3$).

# Mass Spectrum SmartFormula Report

**Analysis Info**

| | | | |
|---|---|---|---|
| Analysis Name | D:\Data\Organikai\2023_04_11\GMP_1189_1-C,2_01_10223.d | Acquisition Date | 4/21/2023 11:18:48 AM |
| Method | organikai_esi_pos_2013_recover.m | Operator | Milda Pukalskiene |
| Sample Name | GMP_1189 | Instrument / Ser# | maXis 4G 20218 |
| Comment | | | |

**Acquisition Parameter**

| | | | | | |
|---|---|---|---|---|---|
| Source Type | ESI | Ion Polarity | Positive | Set Nebulizer | 1.5 Bar |
| Focus | Not active | Set Capillary | 4500 V | Set Dry Heater | 180 °C |
| Scan Begin | 40 m/z | Set End Plate Offset | -500 V | Set Dry Gas | 8.0 l/min |
| Scan End | 1800 m/z | Set Collision Cell RF | 350.0 Vpp | Set Divert Valve | Waste |

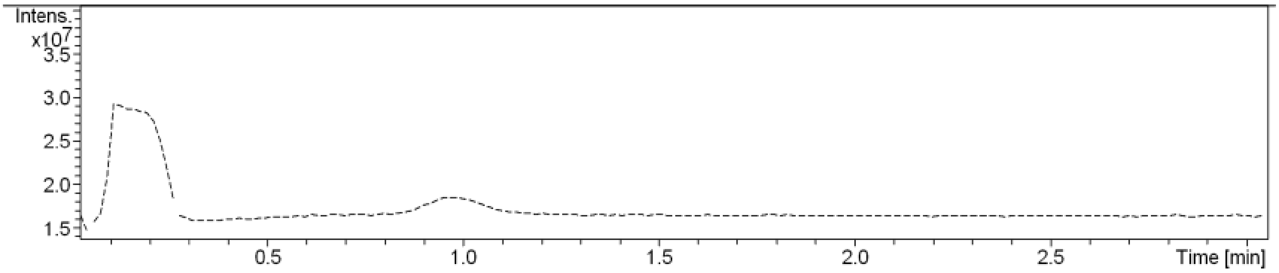


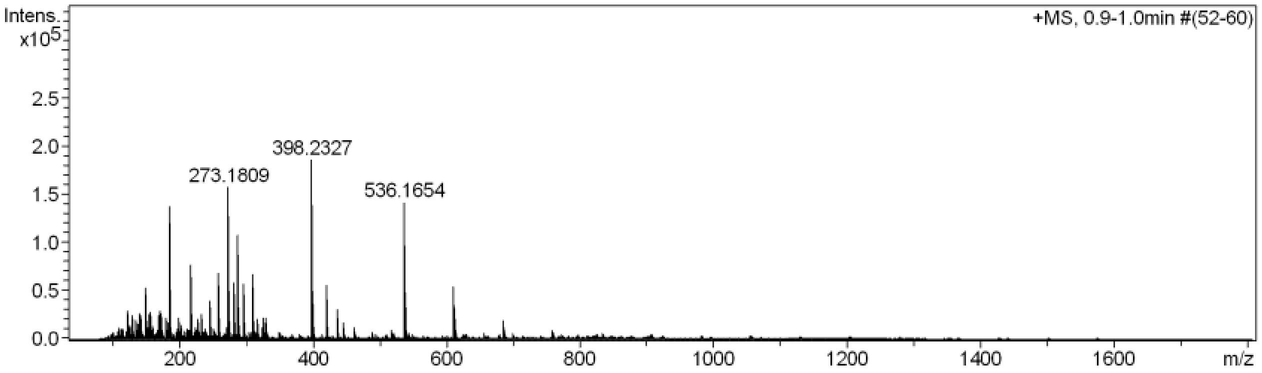


| Meas. m/z | # | Formula | Score | m/z | err [ppm] | Mean err [ppm] | mSigma | rdb | e⁻ Conf | N-Rule |
|---|---|---|---|---|---|---|---|---|---|---|
| 273.1809 | 1 | C 13 H 25 N 2 O 4 | 100.00 | 273.1809 | -0.1 | 0.1 | 1.0 | 2.5 | even | ok |

**Figure S37. (2*R*)-2-{4-[(*tert*-butoxycarbonyl)amino]piperidin-1-yl}propanoic acid ((*R*)-6). HRMS (ESI-TOF).**

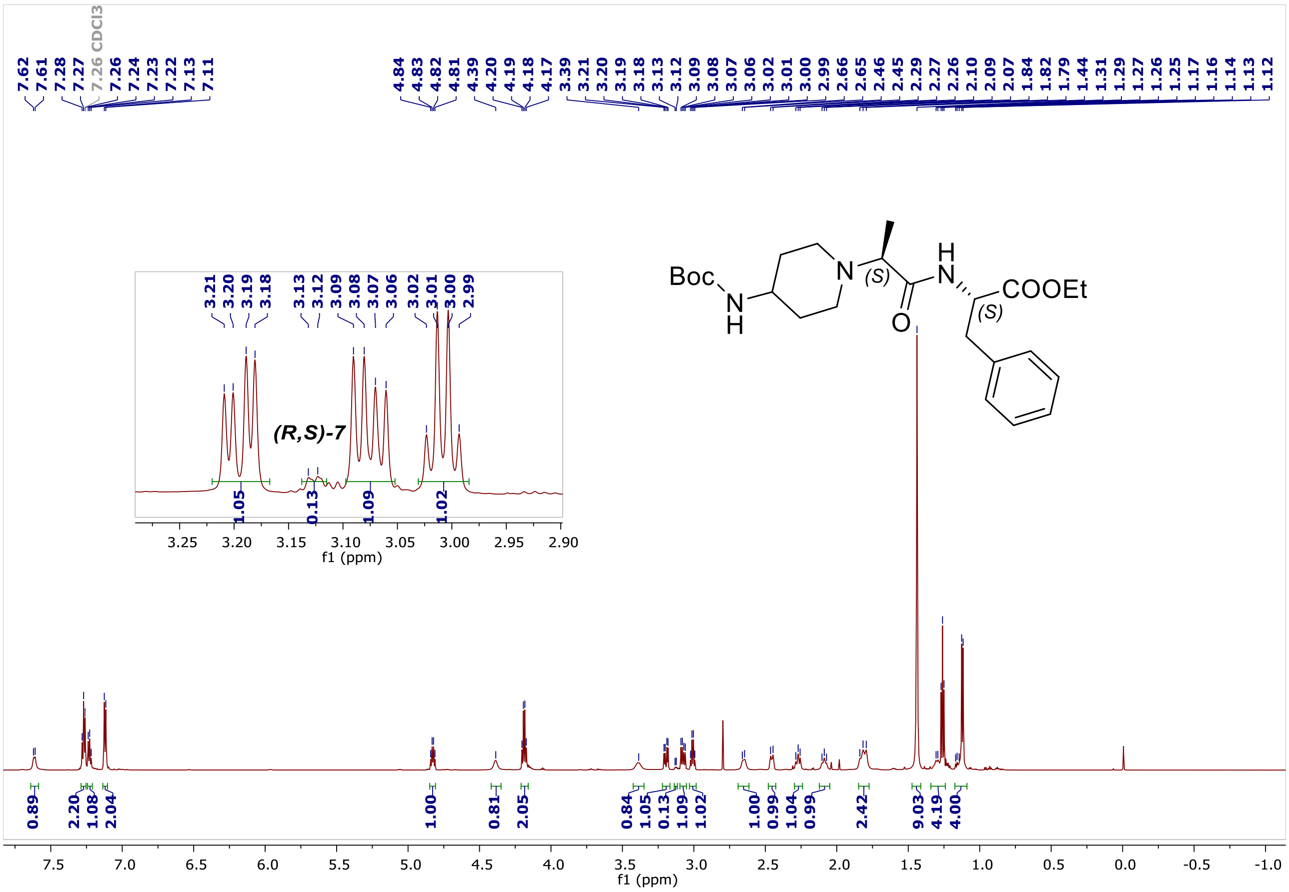


**Figure S38. Ethyl *N*-[(2*S*)-2-{4-[(*tert*-butoxycarbonyl)amino]piperidin-1-yl}propanoyl]-*L*-phenylalaninate ((*S*,*S*)-7). $^{1}$H NMR spectrum (700 MHz, $CDCl_3$).**

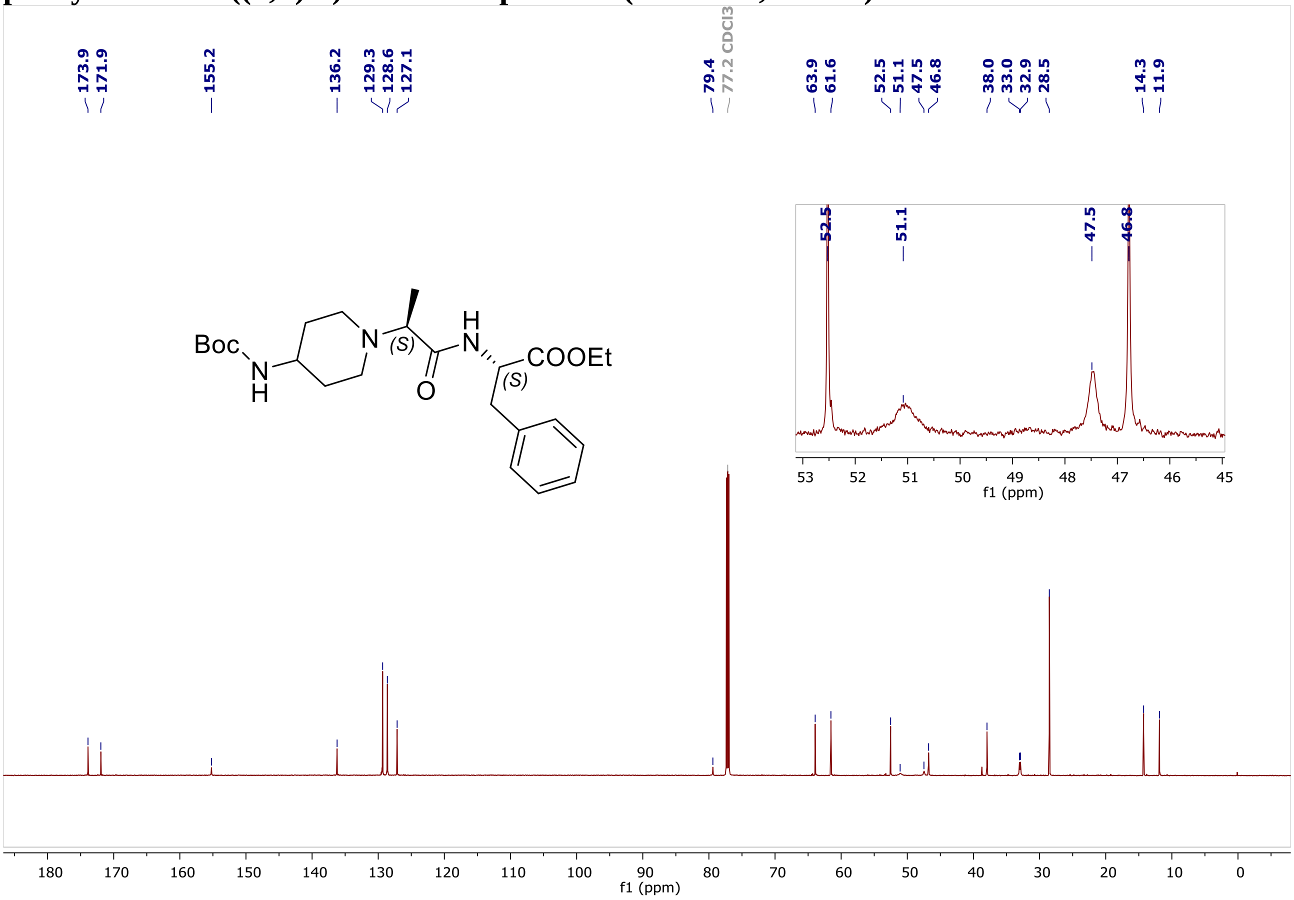


**Figure S39. Ethyl *N*-[(2*S*)-2-{4-[(*tert*-butoxycarbonyl)amino]piperidin-1-yl}propanoyl]-*L*-phenylalaninate ((*S*,*S*)-7). $^{13}$C NMR spectrum (176 MHz, $CDCl_3$).**

# Mass Spectrum SmartFormula Report

**Analysis Info**

| | | | |
|---|---|---|---|
| Analysis Name | D:\Data\Organikai\2023_04_11\GMP_1215_1-C,5_01_10227.d | Acquisition Date | 4/21/2023 1:47:44 PM |
| Method | organikai_esi_pos_2013_recover.m | Operator | Milda Pukalskiene |
| Sample Name | GMP_1215 | Instrument / Ser# | maXis 4G 20218 |
| Comment | | | |

**Acquisition Parameter**

| | | | | | |
|---|---|---|---|---|---|
| Source Type | ESI | Ion Polarity | Positive | Set Nebulizer | 1.5 Bar |
| Focus | Not active | Set Capillary | 4500 V | Set Dry Heater | 180 °C |
| Scan Begin | 40 m/z | Set End Plate Offset | -500 V | Set Dry Gas | 8.0 l/min |
| Scan End | 1800 m/z | Set Collision Cell RF | 350.0 Vpp | Set Divert Valve | Waste |

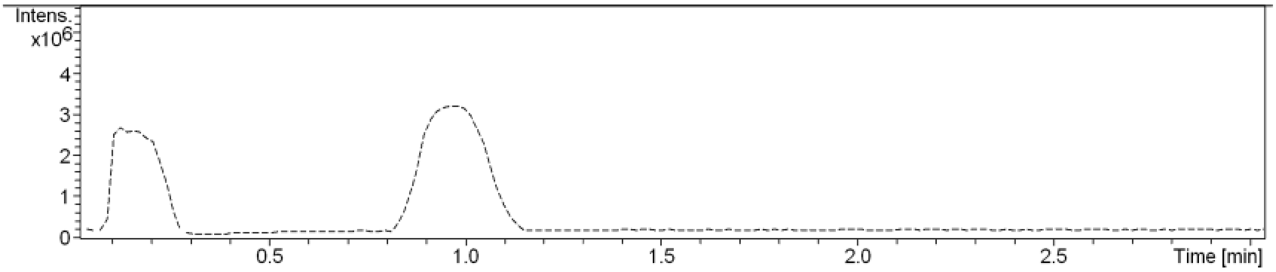


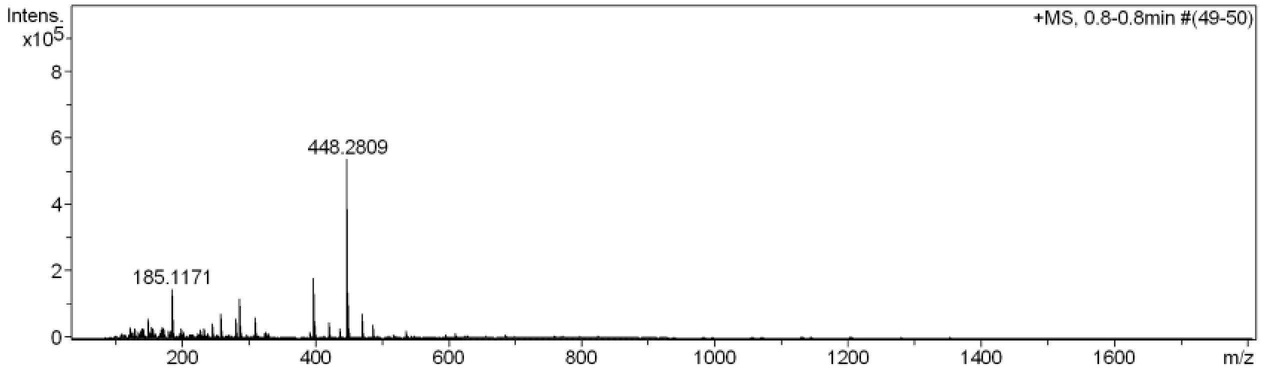


| Meas. m/z | # | Formula | Score | m/z | err [ppm] | Mean err [ppm] | mSigma | rdb | e⁻ Conf | N-Rule |
|---|---|---|---|---|---|---|---|---|---|---|
| 448.2809 | 1 | C 24 H 38 N 3 O 5 | 100.00 | 448.2806 | -0.6 | -0.3 | 14.8 | 7.5 | even | ok |

**Figure S40. Ethyl *N*-[(2*S*)-2-{4-[(*tert*-butoxycarbonyl)amino]piperidin-1-yl}propanoyl]-*L*-phenylalaninate ((*S*,*S*)-7). HRMS (ESI-TOF).**

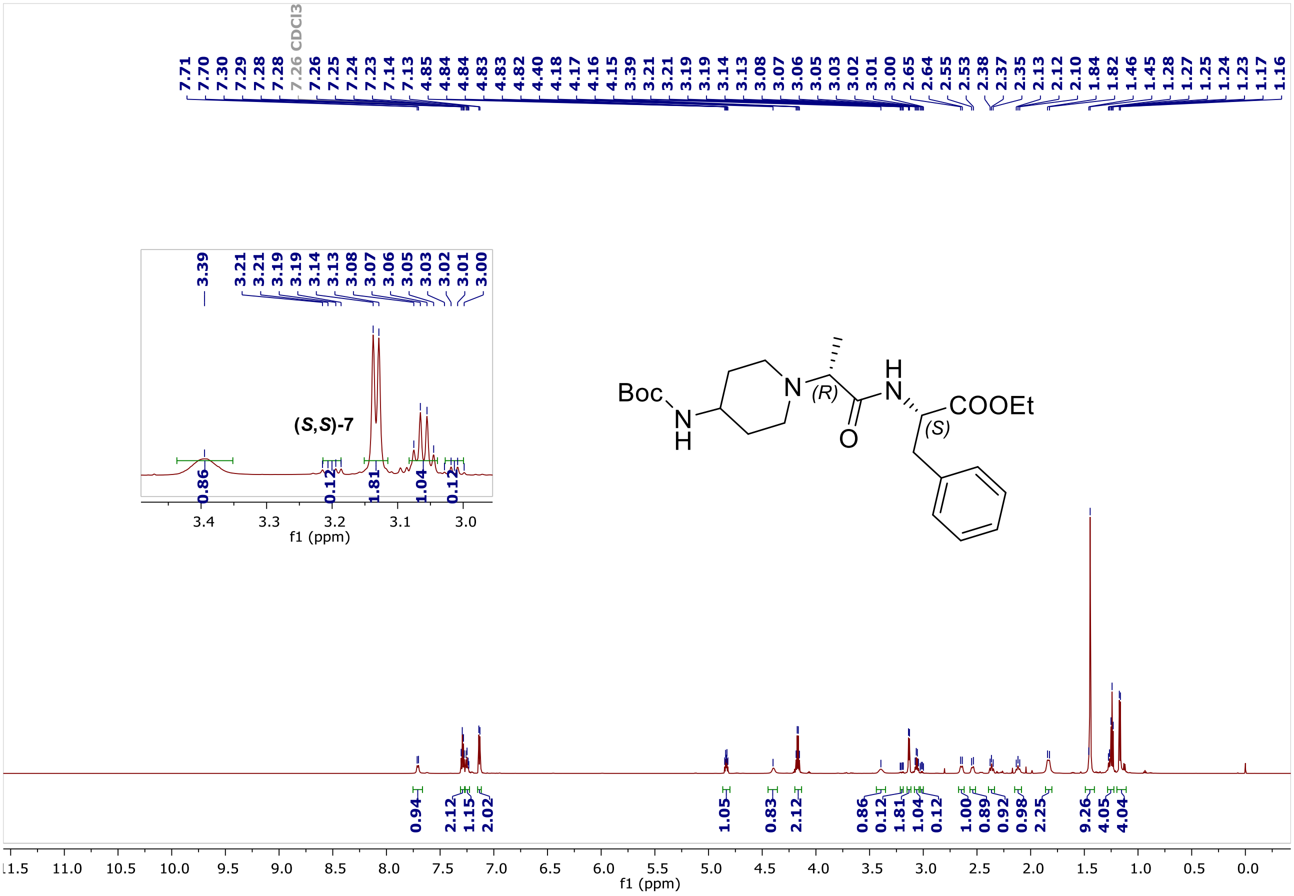

**Figure S41. Ethyl *N*-[(2*R*)-2-{4-[(*tert*-butoxycarbonyl)amino]piperidin-1-yl}propanoyl]-*L*-phenylalaninate ((*R*,*S*)-7). $^{1}$H NMR spectrum (700 MHz, $CDCl_3$).**

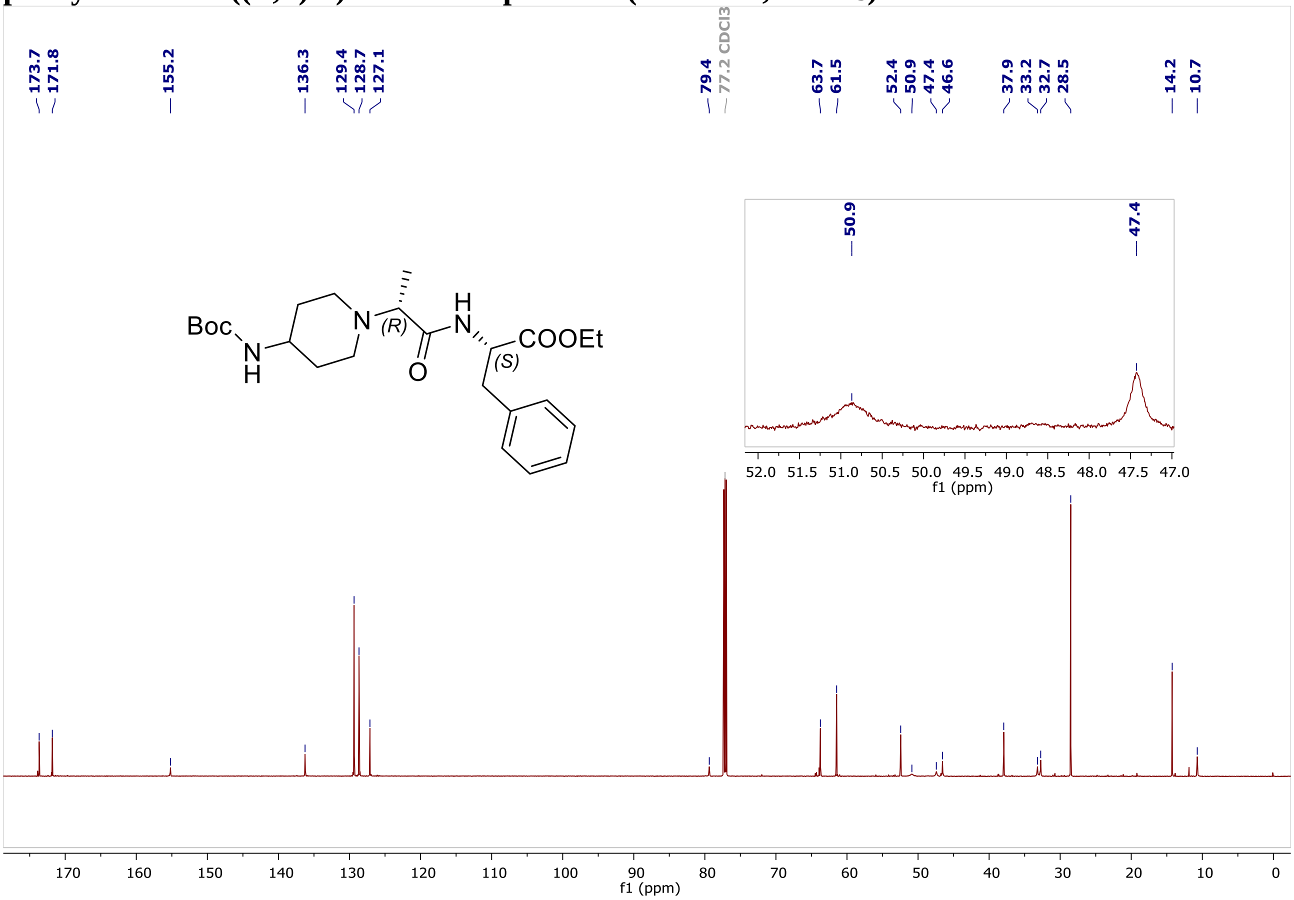

**Figure S42. Ethyl *N*-[(2*R*)-2-{4-[(*tert*-butoxycarbonyl)amino]piperidin-1-yl}propanoyl]-*L*-phenylalaninate ((*R*,*S*)-7). $^{13}$C NMR spectrum (176 MHz, $CDCl_3$).**

# Mass Spectrum SmartFormula Report

**Analysis Info**

| | | | |
|---|---|---|---|
| Analysis Name | D:\Data\Organikai\2023_04_11\GMP_1216_1-C,4_01_10226.d | Acquisition Date | 4/21/2023 1:43:17 PM |
| Method | organikai_esi_pos_2013_recover.m | Operator | Milda Pukalskiene |
| Sample Name | GMP_1216 | Instrument / Ser# | maXis 4G 20218 |
| Comment | | | |

**Acquisition Parameter**

| | | | | | |
|---|---|---|---|---|---|
| Source Type | ESI | Ion Polarity | Positive | Set Nebulizer | 1.5 Bar |
| Focus | Not active | Set Capillary | 4500 V | Set Dry Heater | 180 °C |
| Scan Begin | 40 m/z | Set End Plate Offset | -500 V | Set Dry Gas | 8.0 l/min |
| Scan End | 1800 m/z | Set Collision Cell RF | 350.0 Vpp | Set Divert Valve | Waste |

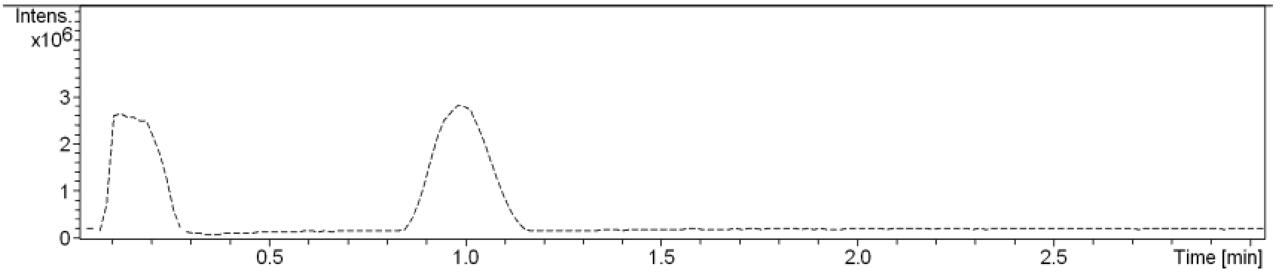


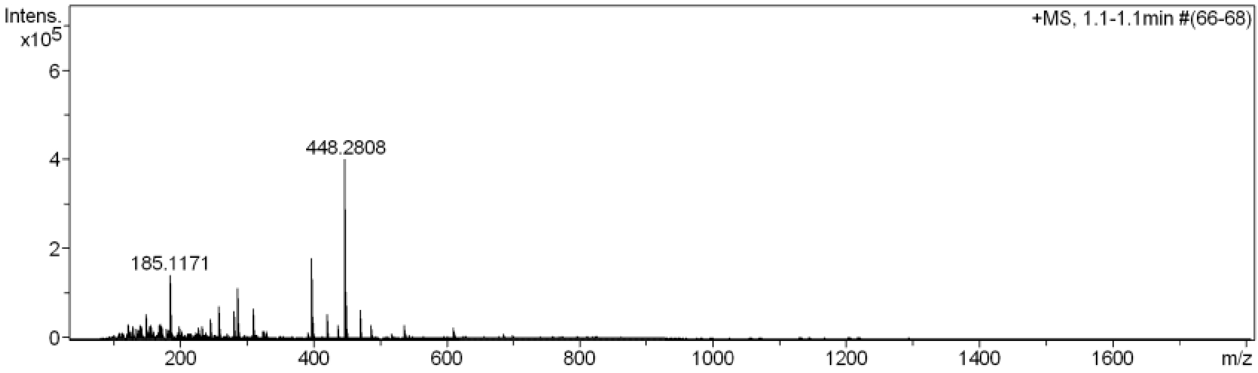


| Meas. m/z | # | Formula | Score | m/z | err [ppm] | Mean err [ppm] | mSigma | rdb | e⁻ Conf | N-Rule |
|---|---|---|---|---|---|---|---|---|---|---|
| 448.2808 | 1 | C 24 H 38 N 3 O 5 | 100.00 | 448.2806 | -0.4 | -0.1 | 9.2 | 7.5 | even | ok |

**Figure S43. Ethyl *N*-[(2*R*)-2-{4-[(*tert*-butoxycarbonyl)amino]piperidin-1-yl}propanoyl]-*L*-phenylalaninate ((*R*,*S*)-7). HRMS (ESI-TOF).**

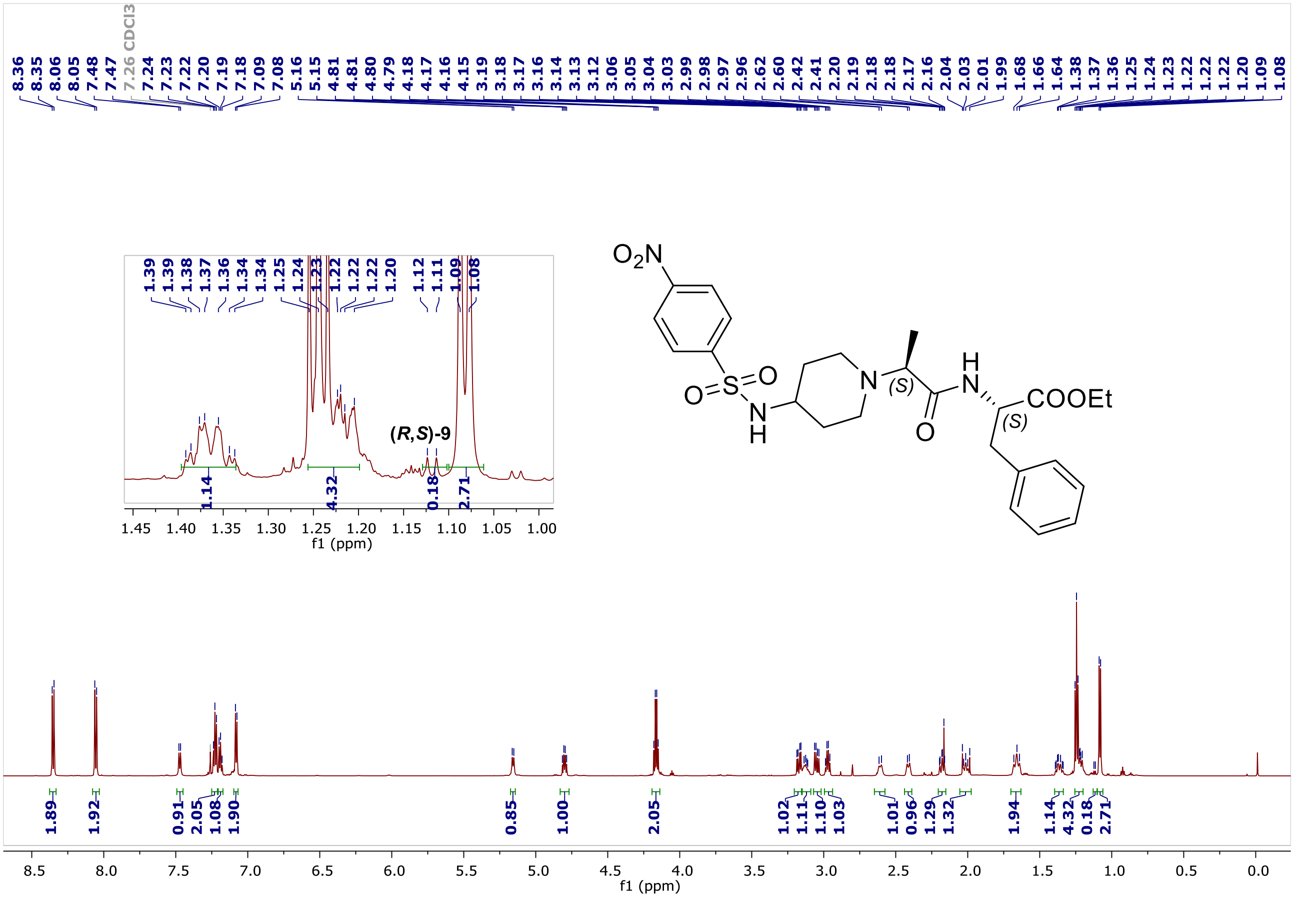

**Figure S44. Ethyl *N*-[(2*S*)-2-{4-[(4-nitrobenzene-1-sulfonyl)amino]piperidin-1-yl}propanoyl]-*L*-phenylalaninate ((*S*,*S*)-9). $^{1}H$ NMR spectrum (700 MHz, $CDCl_3$).**

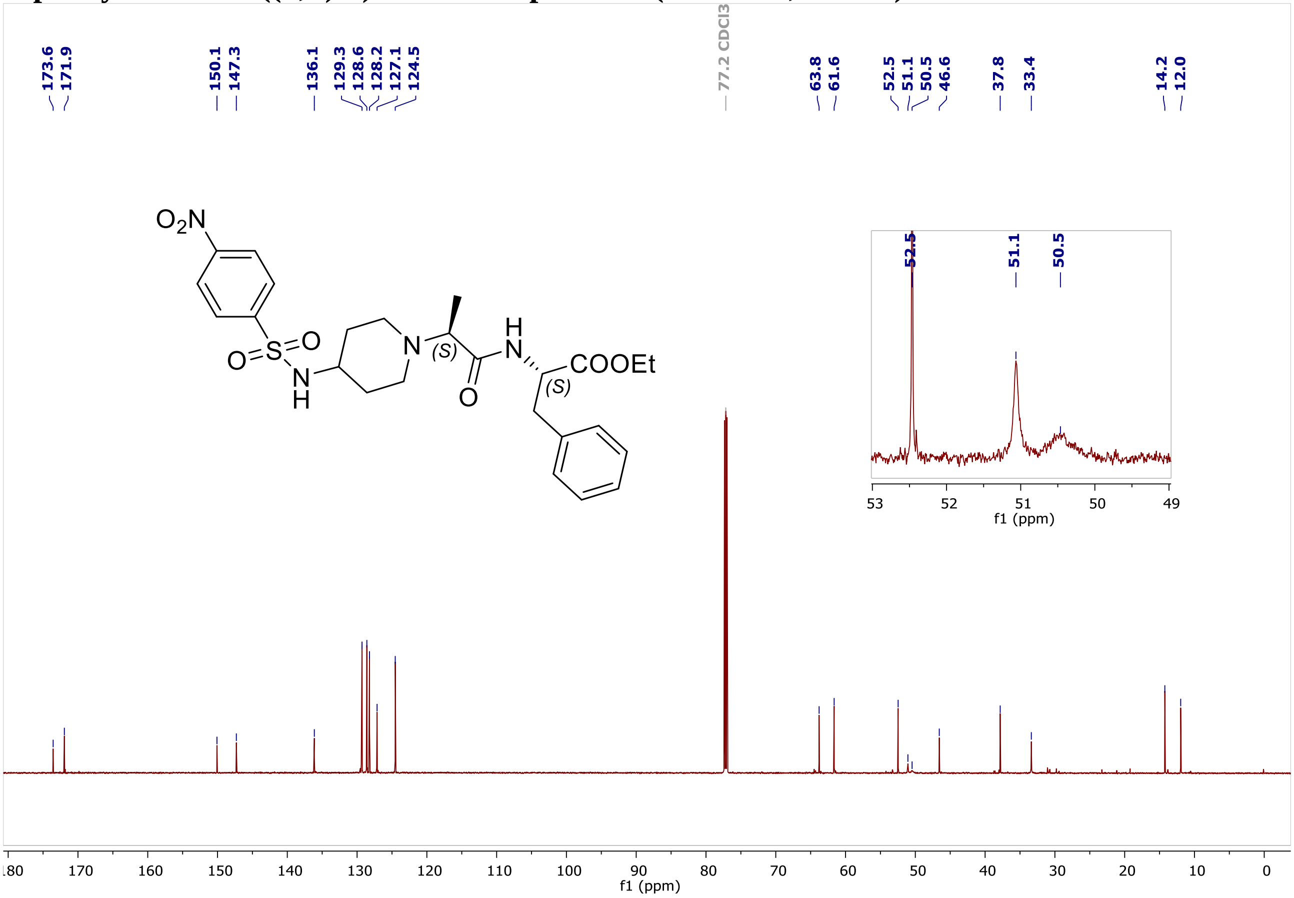

**Figure S45. Ethyl *N*-[(2*S*)-2-{4-[(4-nitrobenzene-1-sulfonyl)amino]piperidin-1-yl}propanoyl]-*L*-phenylalaninate ((*S*,*S*)-9). $^{13}C$ NMR spectrum (176 MHz, $CDCl_3$).**

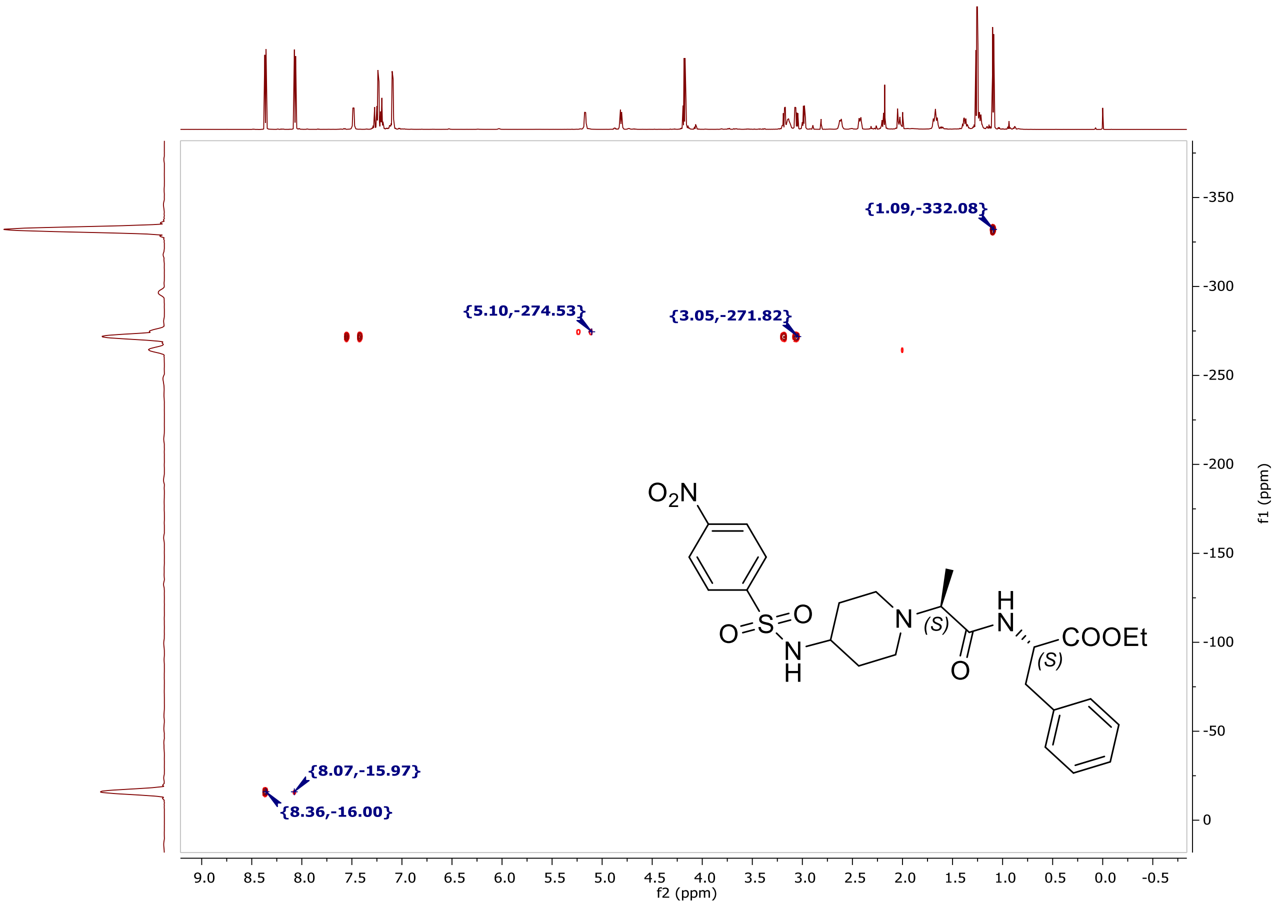


**Figure S46. Ethyl *N*-[(2*S*)-2-{4-[(4-nitrobenzene-1-sulfonyl)amino]piperidin-1-yl}propanoyl]-*L*-phenylalaninate ((*S*,*S*)-9). $^{1}$H-$^{15}$N HMBC spectrum (71 MHz, $CDCl_3$).**

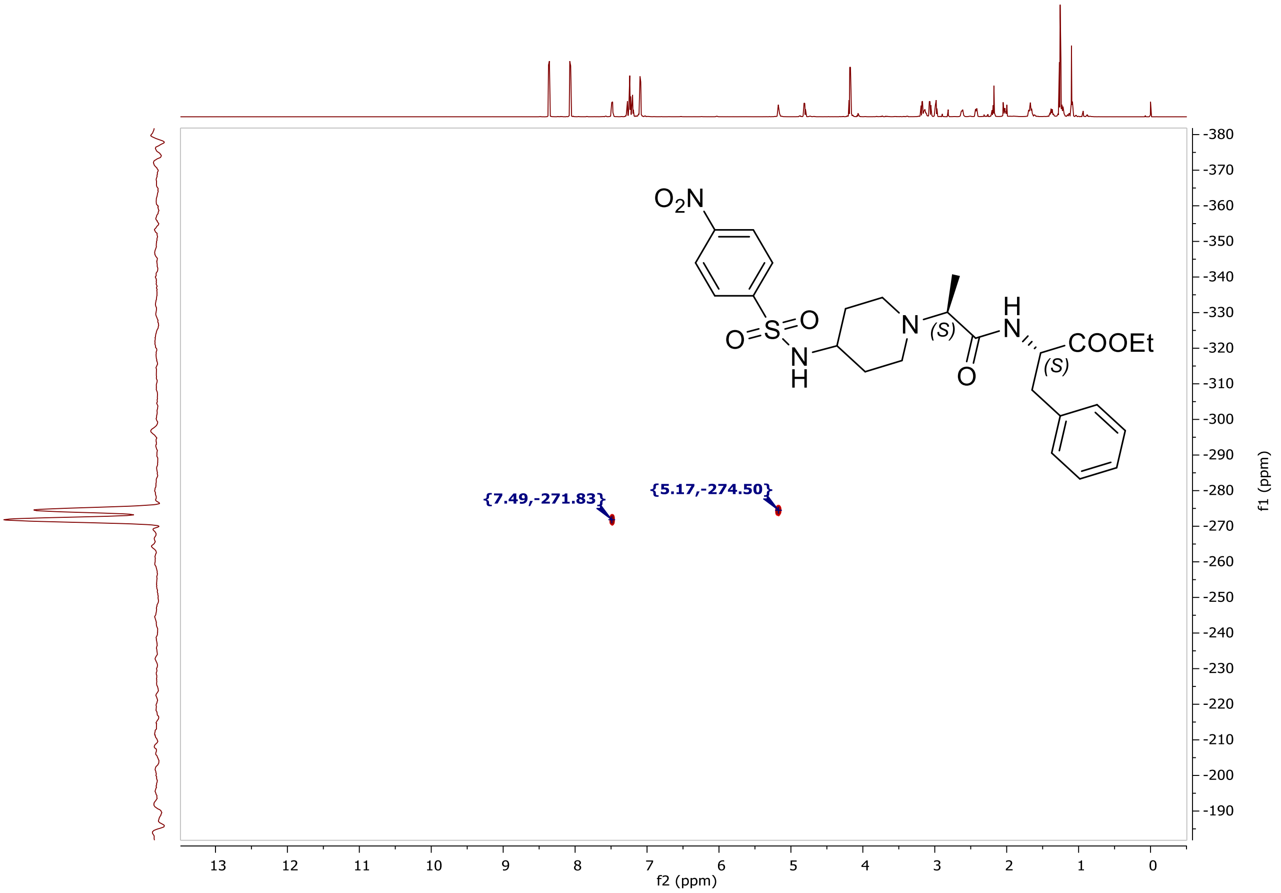


**Figure S47. Ethyl *N*-[(2*S*)-2-{4-[(4-nitrobenzene-1-sulfonyl)amino]piperidin-1-yl}propanoyl]-*L*-phenylalaninate ((*S*,*S*)-9). $^{1}$H-$^{15}$N HSQC spectrum (71 MHz, $CDCl_3$).**

# Mass Spectrum SmartFormula Report

**Analysis Info**

| | | | |
|---|---|---|---|
| Analysis Name | D:\Data\Organikai\2023_04_11\GMP_1217_1-C,7_01_10229.d | Acquisition Date | 4/21/2023 1:56:42 PM |
| Method | organikai_esi_pos_2013_recover.m | Operator | Milda Pukalskiene |
| Sample Name | GMP_1217 | Instrument / Ser# | maXis 4G 20218 |
| Comment | | | |

**Acquisition Parameter**

| | | | | | |
|---|---|---|---|---|---|
| Source Type | ESI | Ion Polarity | Positive | Set Nebulizer | 1.5 Bar |
| Focus | Not active | Set Capillary | 4500 V | Set Dry Heater | 180 °C |
| Scan Begin | 40 m/z | Set End Plate Offset | -500 V | Set Dry Gas | 8.0 l/min |
| Scan End | 1800 m/z | Set Collision Cell RF | 350.0 Vpp | Set Divert Valve | Waste |

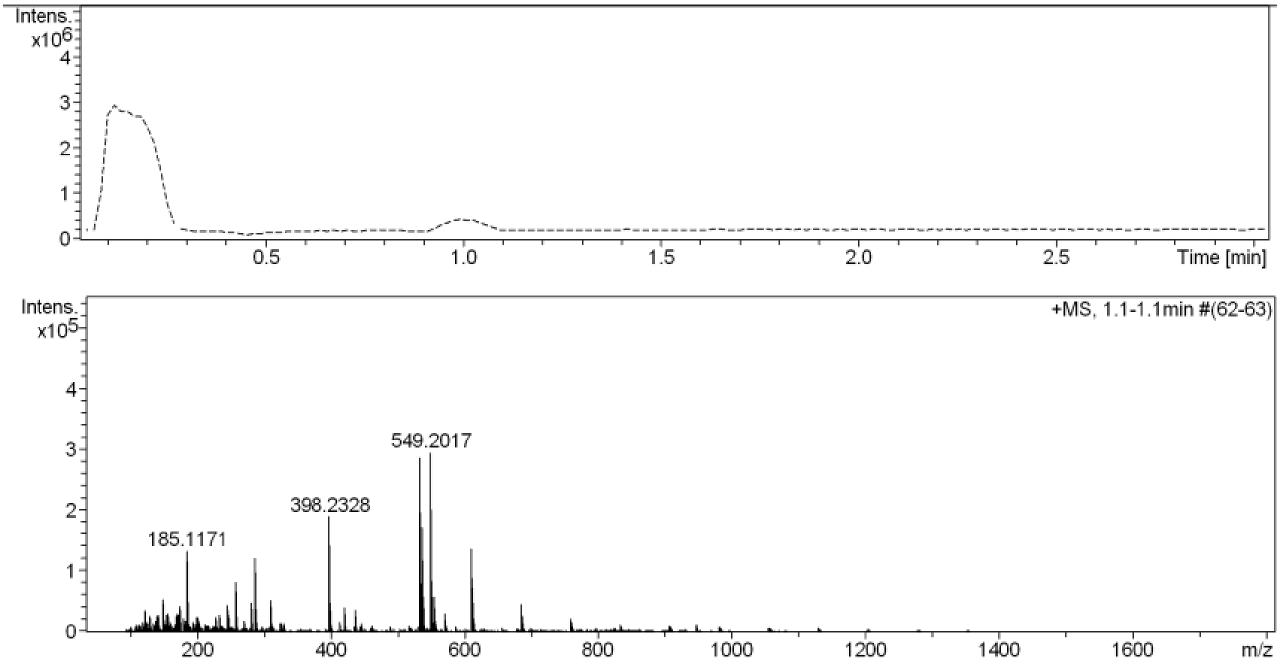


| Meas. m/z | # | Formula | Score | m/z | err [ppm] | Mean err [ppm] | mSigma | rdb | e⁻ Conf | N-Rule |
|---|---|---|---|---|---|---|---|---|---|---|
| 533.2068 | 1 | C 24 H 37 O 11 S | 49.10 | 533.2051 | -3.1 | -3.4 | 15.3 | 6.5 | even | ok |
| | 2 | C 25 H 33 N 4 O 7 S | 100.00 | 533.2064 | -0.6 | -0.9 | 17.9 | 11.5 | even | ok |
| | 3 | C 20 H 37 O 16 | 63.13 | 533.2076 | 1.6 | 2.2 | 27.1 | 2.5 | even | ok |
| | 4 | C 33 H 29 N 2 O 5 | 51.17 | 533.2071 | 0.6 | 1.5 | 44.9 | 20.5 | even | ok |

**Figure S48. Ethyl *N*-[(2*S*)-2-{4-[(4-nitrobenzene-1-sulfonyl)amino]piperidin-1-yl}propanoyl]-*L*-phenylalaninate ((*S*,*S*)-9). HRMS (ESI-TOF).**

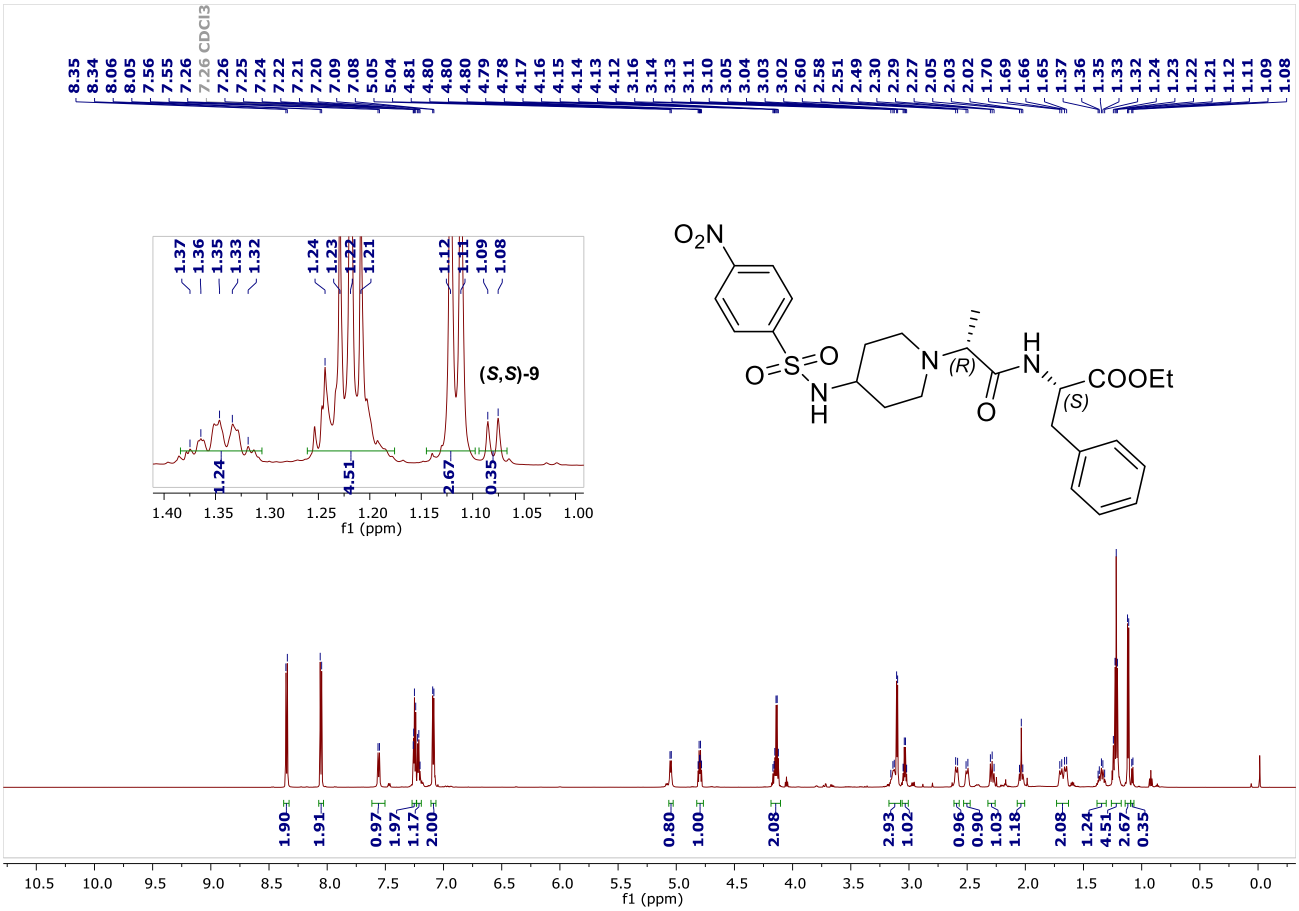


**Figure S49. Ethyl *N*-[(2*R*)-2-{4-[(4-nitrobenzene-1-sulfonyl)amino]piperidin-1-yl}propanoyl]-*L*-phenylalaninate ((*R*,*S*)-9). $^1$H NMR spectrum (700 MHz, $CDCl_3$).**

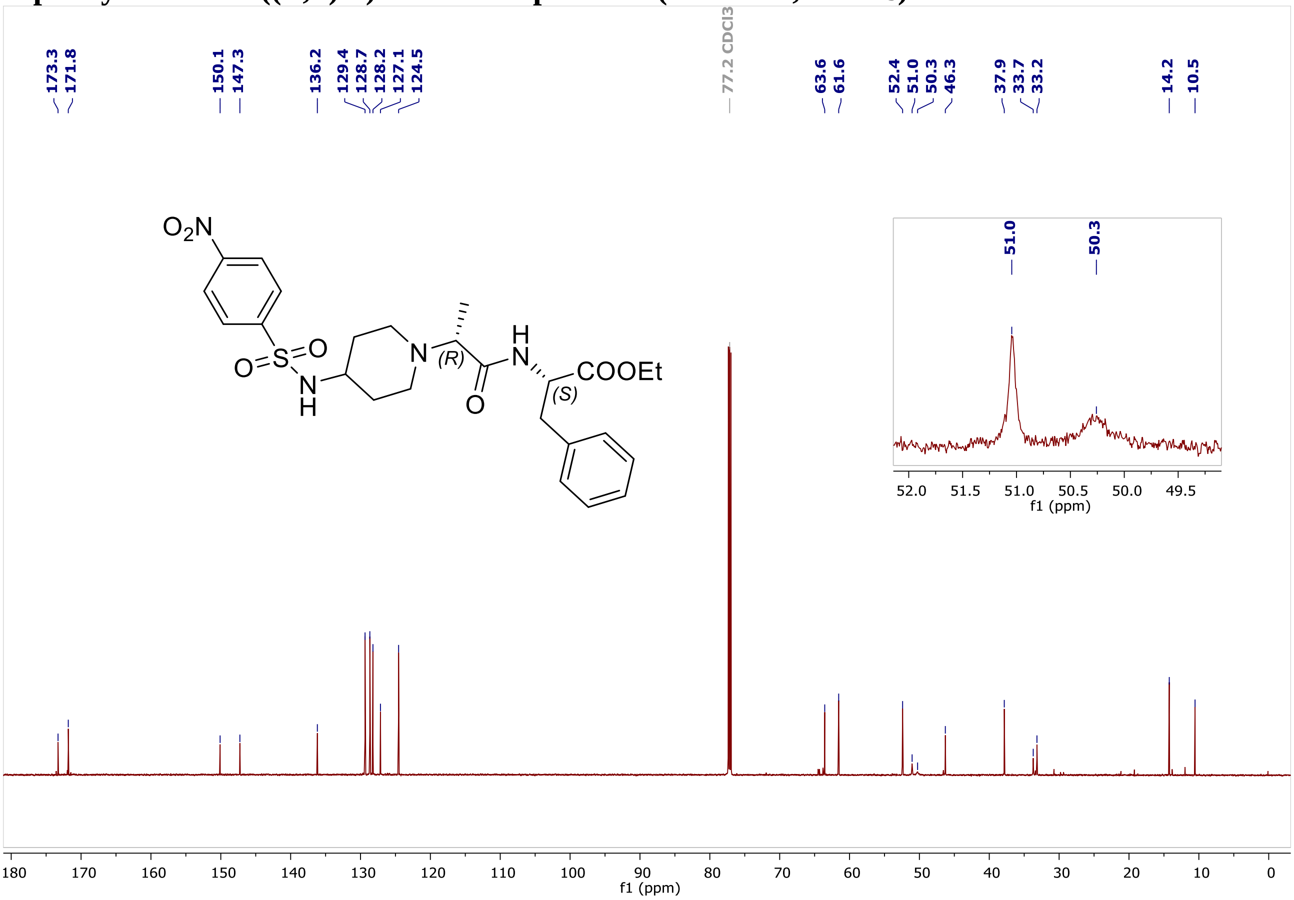


**Figure S50. Ethyl *N*-[(2*R*)-2-{4-[(4-nitrobenzene-1-sulfonyl)amino]piperidin-1-yl}propanoyl]-*L*-phenylalaninate ((*R*,*S*)-9). $^{13}$C NMR spectrum (176 MHz, $CDCl_3$).**

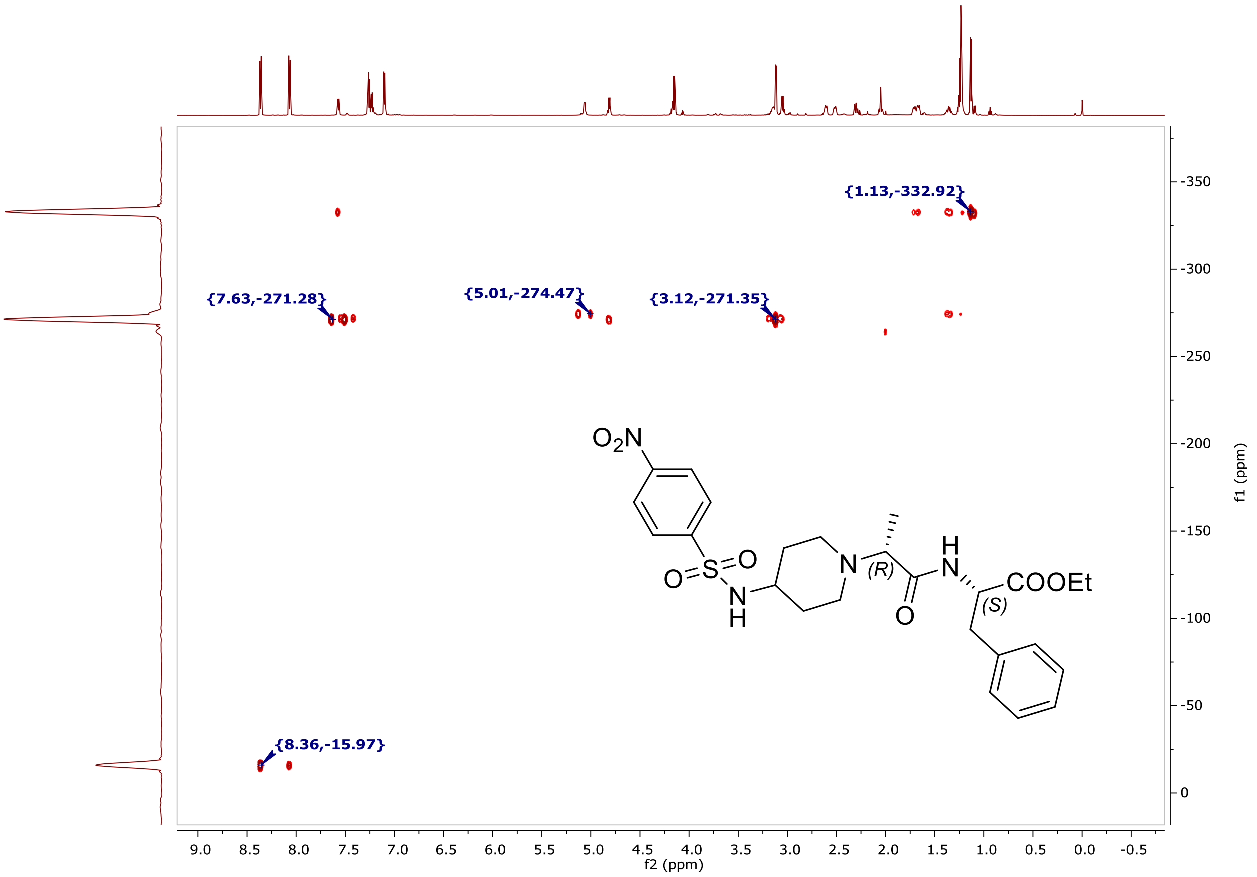


**Figure S51.** **Ethyl** ***N*****-[(2*****R*****)-2-{4-[(4-nitrobenzene-1-sulfonyl)amino]piperidin-1-yl}propanoyl]-*****L*****-phenylalaninate ((*****R*****,*****S*****)-9).** $^{1}H$-$^{15}N$ **HMBC spectrum (71 MHz, $CDCl_3$).**

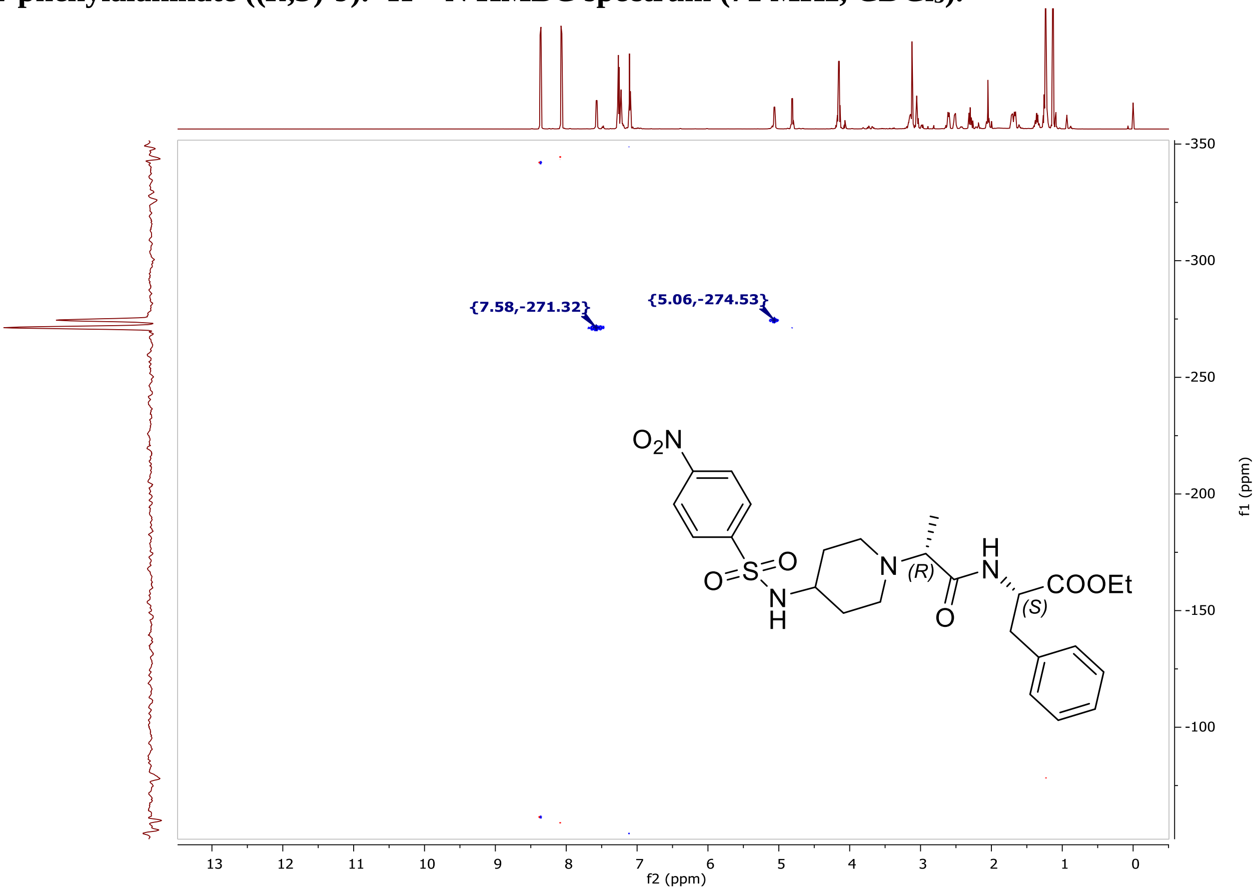


**Figure S52.** **Ethyl** ***N*****-[(2*****R*****)-2-{4-[(4-nitrobenzene-1-sulfonyl)amino]piperidin-1-yl}propanoyl]-*****L*****-phenylalaninate ((*****R*****,*****S*****)-9).** $^{1}H$-$^{15}N$ **HSQC spectrum (71 MHz, $CDCl_3$).**

# Mass Spectrum SmartFormula Report

**Analysis Info**

| | | | |
|---|---|---|---|
| Analysis Name | D:\Data\Organikai\2023_04_11\GMP_1218_1-C,6_01_10228.d | Acquisition Date | 4/21/2023 1:52:14 PM |
| Method | organikai_esi_pos_2013_recover.m | Operator | Milda Pukalskiene |
| Sample Name | GMP_1218 | Instrument / Ser# | maXis 4G 20218 |
| Comment | | | |

**Acquisition Parameter**

| | | | | | |
|---|---|---|---|---|---|
| Source Type | ESI | Ion Polarity | Positive | Set Nebulizer | 1.5 Bar |
| Focus | Not active | Set Capillary | 4500 V | Set Dry Heater | 180 °C |
| Scan Begin | 40 m/z | Set End Plate Offset | -500 V | Set Dry Gas | 8.0 l/min |
| Scan End | 1800 m/z | Set Collision Cell RF | 350.0 Vpp | Set Divert Valve | Waste |

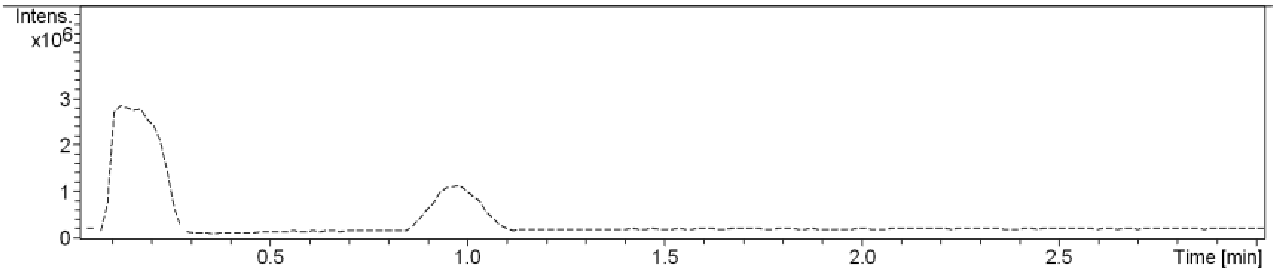


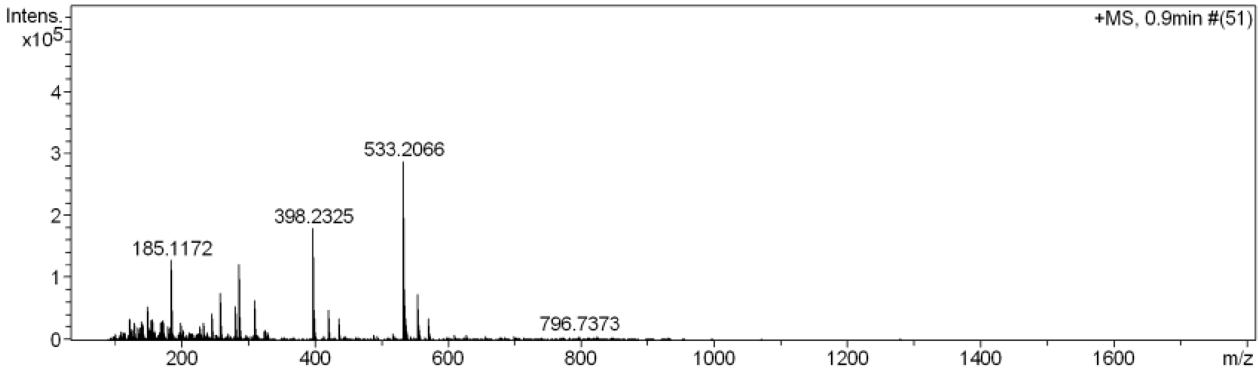


| Meas. m/z | # | Formula | Score | m/z | err [ppm] | Mean err [ppm] | mSigma | rdb | e⁻ Conf | N-Rule |
|---|---|---|---|---|---|---|---|---|---|---|
| 533.2066 | 1 | C 24 H 37 O 11 S | 48.79 | 533.2051 | -2.8 | -3.1 | 17.8 | 6.5 | even | ok |
| | 2 | C 25 H 33 N 4 O 7 S | 100.00 | 533.2064 | -0.3 | -0.6 | 18.0 | 11.5 | even | ok |
| | 3 | C 20 H 37 O 16 | 50.43 | 533.2076 | 1.9 | 2.4 | 29.4 | 2.5 | even | ok |
| | 4 | C 33 H 29 N 2 O 5 | 48.33 | 533.2071 | 0.9 | 1.7 | 41.4 | 20.5 | even | ok |

**Figure S53. Ethyl *N*-[(2*R*)-2-{4-[(4-nitrobenzene-1-sulfonyl)amino]piperidin-1-yl}propanoyl]-*L*-phenylalaninate ((*R*,*S*)-9). HRMS (ESI-TOF).**

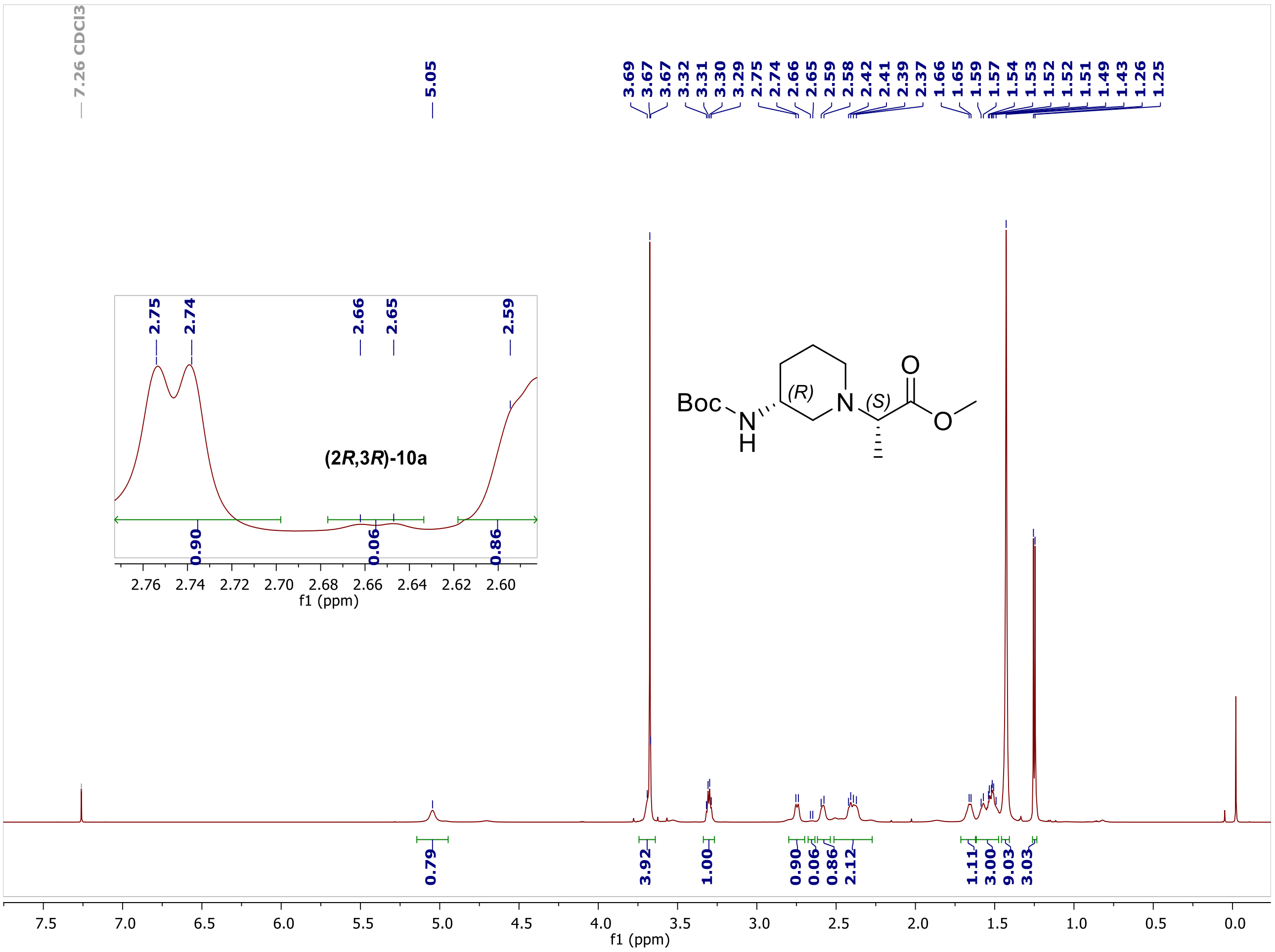


**Figure S54.** **Methyl (2*S*)-2-{(3*R*)-3-[(*tert*-butoxycarbonyl)amino]piperidin-1-yl}propanoate ((2*S*,3*R*)-10a). $^{1}$H NMR spectrum (700 MHz, $CDCl_3$).**

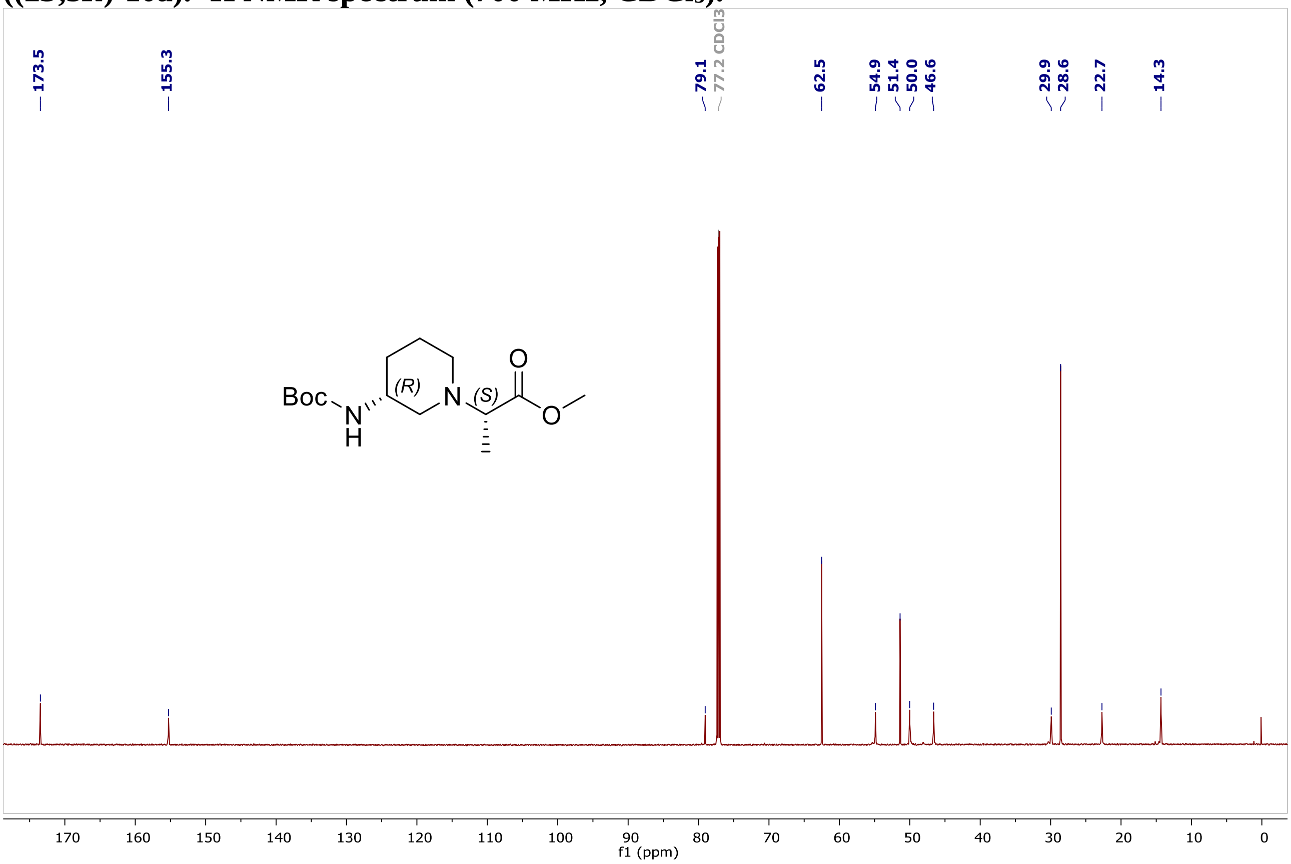


**Figure S55.** **Methyl (2*S*)-2-{(3*R*)-3-[(*tert*-butoxycarbonyl)amino]piperidin-1-yl}propanoate ((2*S*,3*R*)-10a). $^{13}$C NMR spectrum (176 MHz, $CDCl_3$).**

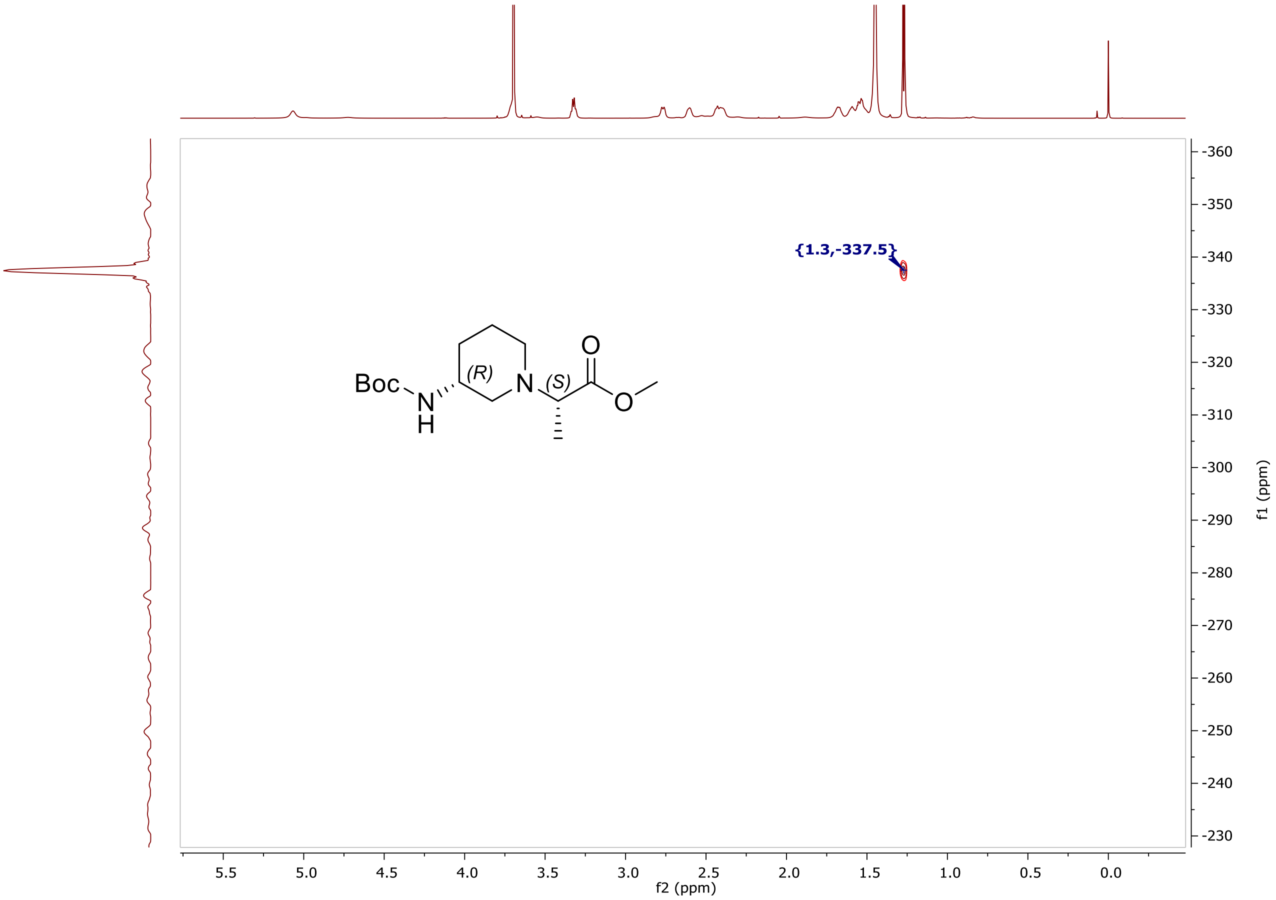


**Figure S56. Methyl (2*S*)-2-{(3*R*)-3-[(*tert*-butoxycarbonyl)amino]piperidin-1-yl}propanoate ((2*S*,3*R*)-10a). $^{1}H$-$^{15}N$ HMBC spectrum (71 MHz, $CDCl_3$).**

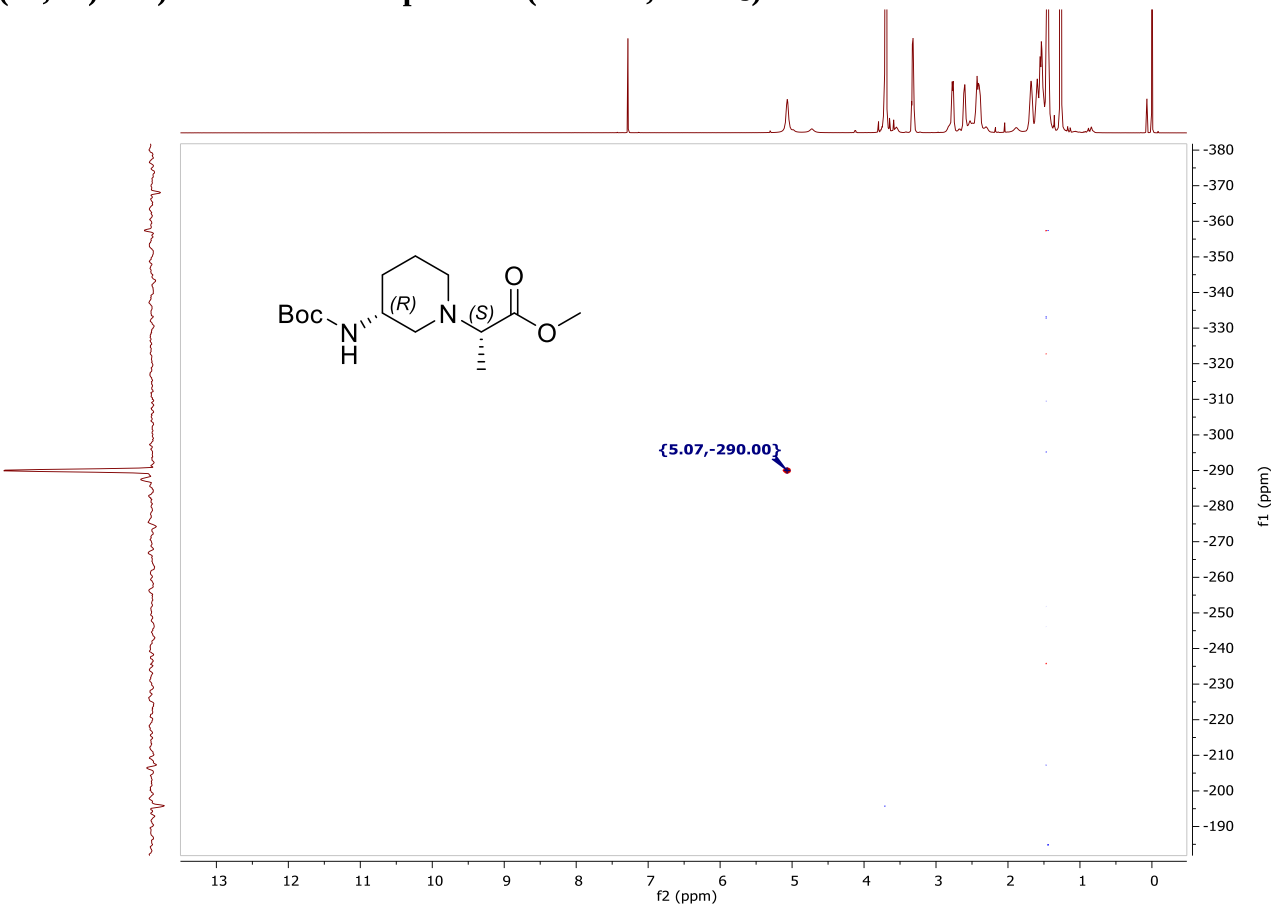


**Figure S57. Methyl (2*S*)-2-{(3*R*)-3-[(*tert*-butoxycarbonyl)amino]piperidin-1-yl}propanoate ((2*S*,3*R*)-10a). $^{1}H$-$^{15}N$ HSQC spectrum (71 MHz, $CDCl_3$).**

# Mass Spectrum SmartFormula Report

**Analysis Info**

| | | | |
|---|---|---|---|
| Analysis Name | D:\Data\Organikai\2023_04_11\GMP_367_1-C,8_01_10230.d | Acquisition Date | 4/21/2023 2:01:08 PM |
| Method | organikai_esi_pos_2013_recover.m | Operator | Milda Pukalskiene |
| Sample Name | GMP_367 | Instrument / Ser# | maXis 4G 20218 |
| Comment | | | |

**Acquisition Parameter**

| | | | | | |
|---|---|---|---|---|---|
| Source Type | ESI | Ion Polarity | Positive | Set Nebulizer | 1.5 Bar |
| Focus | Not active | Set Capillary | 4500 V | Set Dry Heater | 180 °C |
| Scan Begin | 40 m/z | Set End Plate Offset | -500 V | Set Dry Gas | 8.0 l/min |
| Scan End | 1800 m/z | Set Collision Cell RF | 350.0 Vpp | Set Divert Valve | Waste |

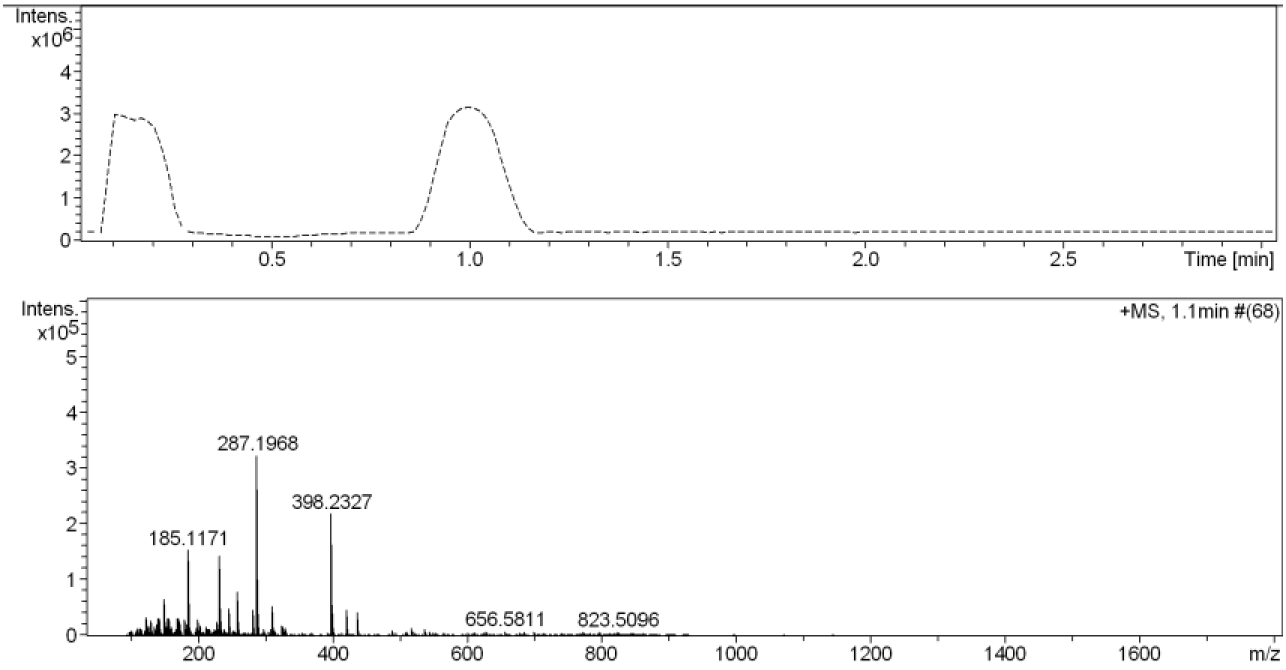


| Meas. m/z | # | Formula | Score | m/z | err [ppm] | Mean err [ppm] | mSigma | rdb | e⁻ Conf | N-Rule |
|---|---|---|---|---|---|---|---|---|---|---|
| 287.1968 | 1 | C 14 H 27 N 2 O 4 | 100.00 | 287.1965 | -0.9 | -0.7 | 13.3 | 2.5 | even | ok |

**Figure S58. Methyl (2*S*)-2-{(3*R*)-3-[(*tert*-butoxycarbonyl)amino]piperidin-1-yl}propanoate ((2*S*,3*R*)-10a). HRMS (ESI-TOF).**

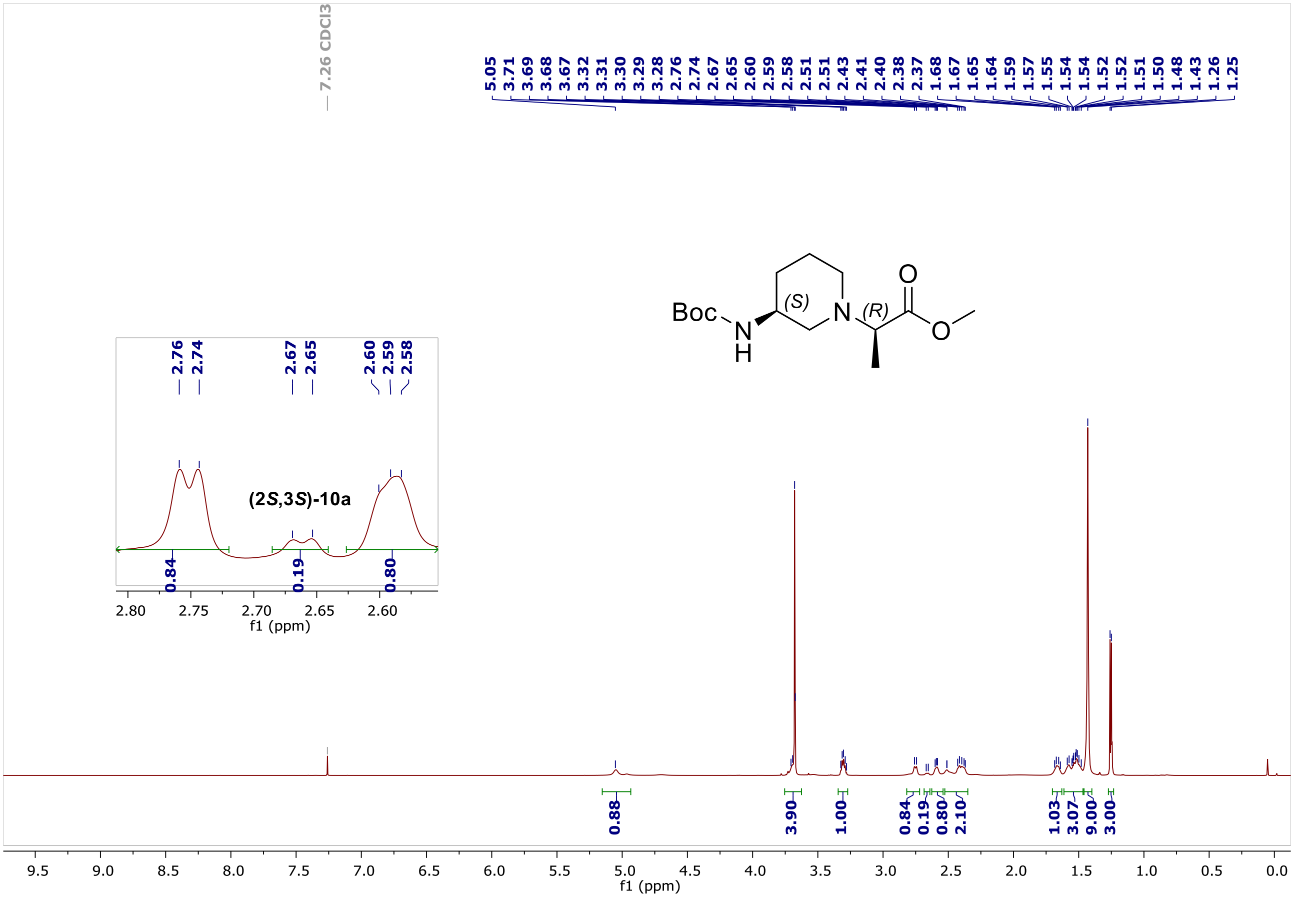


**Figure S59. Methyl (2*R*)-2-{(3*S*)-3-[(*tert*-butoxycarbonyl)amino]piperidin-1-yl}propanoate ((2*R*,3*S*)-10a). $^{1}$H NMR spectrum (700 MHz, $CDCl_3$).**

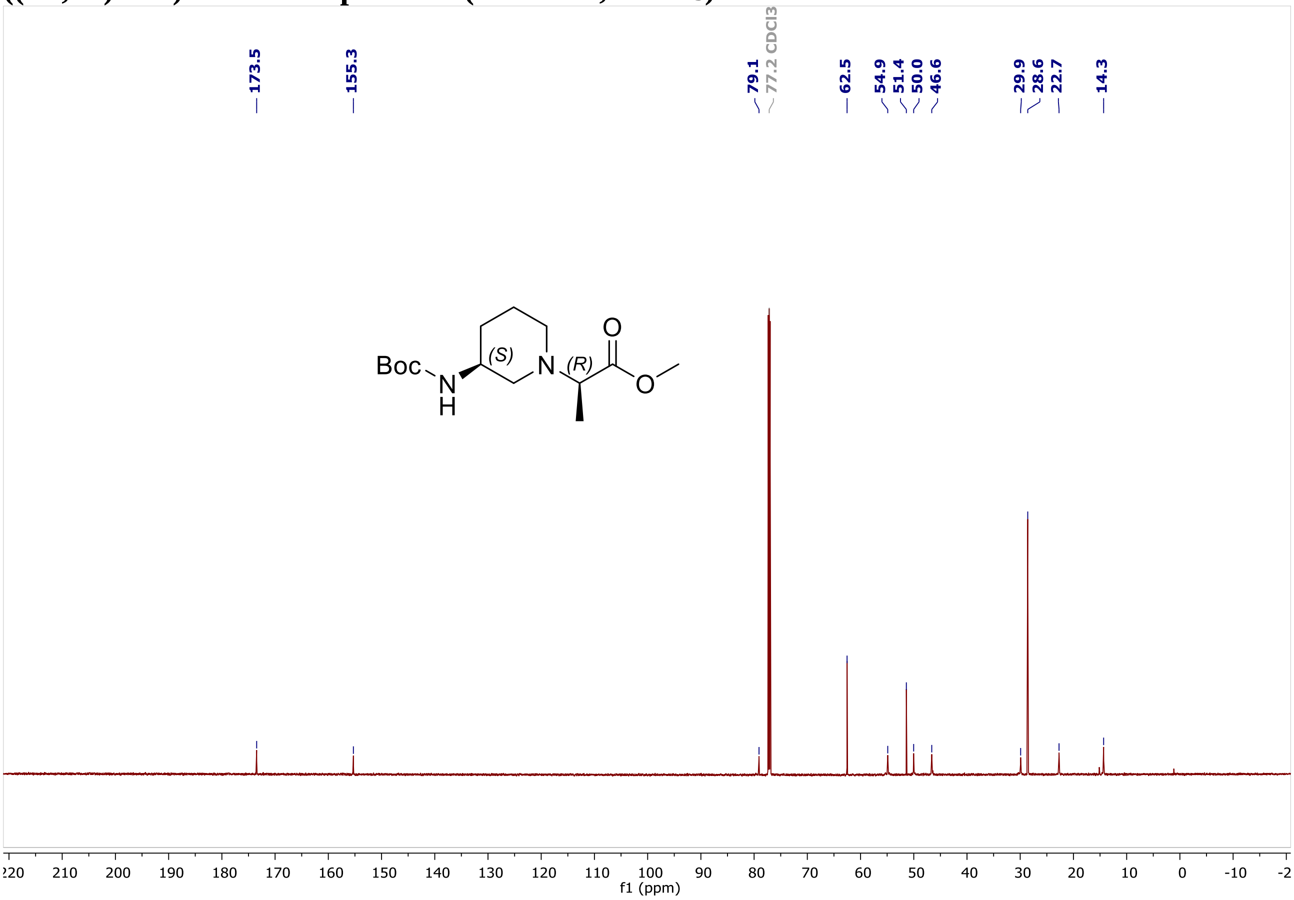


**Figure S60. Methyl (2*R*)-2-{(3*S*)-3-[(*tert*-butoxycarbonyl)amino]piperidin-1-yl}propanoate ((2*R*,3*S*)-10a). $^{13}$C NMR spectrum (176 MHz, $CDCl_3$).**

# Mass Spectrum SmartFormula Report

**Analysis Info**

| | | | |
|---|---|---|---|
| Analysis Name | D:\Data\Organikai\2023_04_11\GMP_462_1-D,3_01_10233.d | Acquisition Date | 4/21/2023 2:14:25 PM |
| Method | organikai_esi_pos_2013_recover.m | Operator | Milda Pukalskiene |
| Sample Name | GMP_462 | Instrument / Ser# | maXis 4G 20218 |
| Comment | | | |

**Acquisition Parameter**

| | | | | | |
|---|---|---|---|---|---|
| Source Type | ESI | Ion Polarity | Positive | Set Nebulizer | 1.5 Bar |
| Focus | Not active | Set Capillary | 4500 V | Set Dry Heater | 180 °C |
| Scan Begin | 40 m/z | Set End Plate Offset | -500 V | Set Dry Gas | 8.0 l/min |
| Scan End | 1800 m/z | Set Collision Cell RF | 350.0 Vpp | Set Divert Valve | Waste |

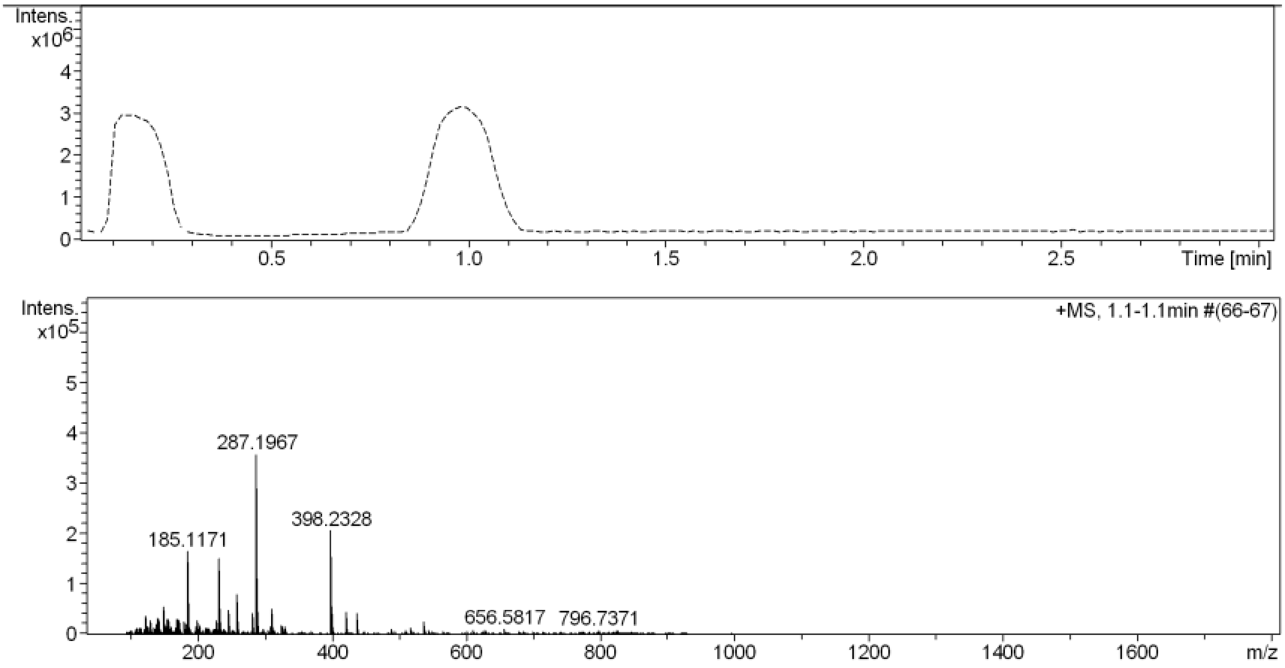


| Meas. m/z | # | Formula | Score | m/z | err [ppm] | Mean err [ppm] | mSigma | rdb | e⁻ Conf | N-Rule |
|---|---|---|---|---|---|---|---|---|---|---|
| 287.1967 | 1 | C 14 H 27 N 2 O 4 | 100.00 | 287.1965 | -0.6 | -0.2 | 1.0 | 2.5 | even | ok |

**Figure S61. Methyl (2*R*)-2-{(3*S*)-3-[(*tert*-butoxycarbonyl)amino]piperidin-1-yl}propanoate ((2*R*,3*S*)-10a). HRMS (ESI-TOF).**

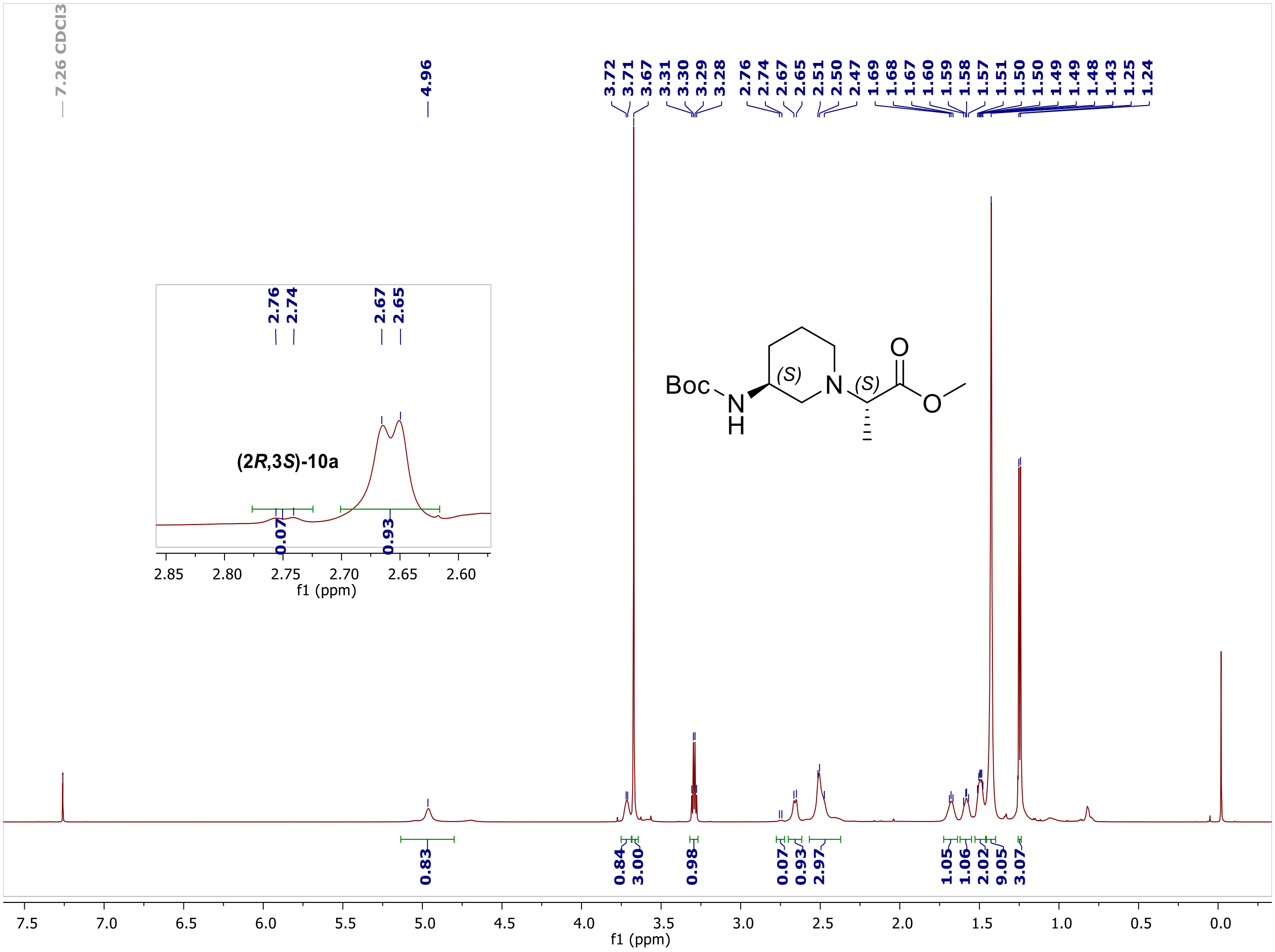


**Figure S62. Methyl (2*S*)-2-{(3*S*)-3-[(*tert*-butoxycarbonyl)amino]piperidin-1-yl}propanoate ((2*S*,3*S*)-10a). $^{1}$H NMR spectrum (700 MHz, $CDCl_3$).**

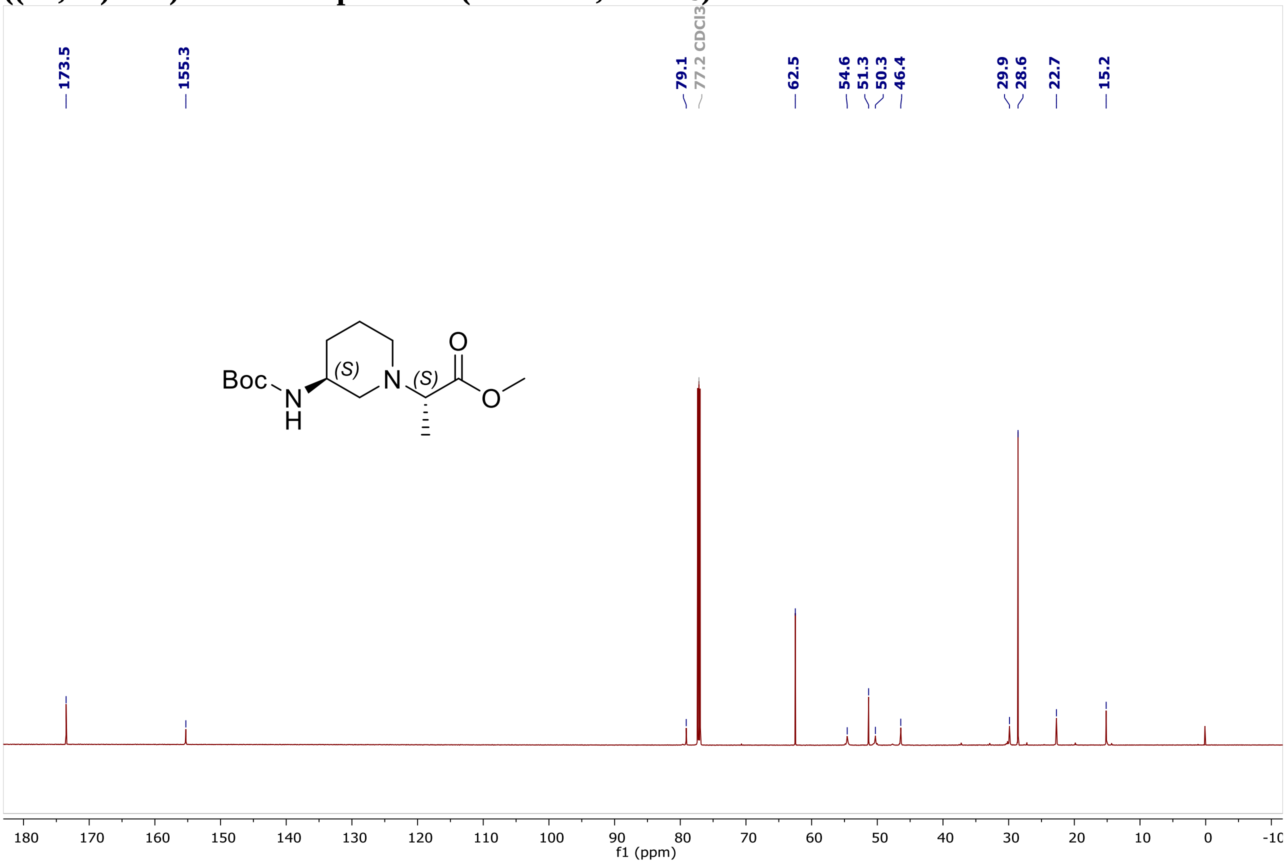


**Figure S63. Methyl (2*S*)-2-{(3*S*)-3-[(*tert*-butoxycarbonyl)amino]piperidin-1-yl}propanoate ((2*S*,3*S*)-10a). $^{13}$C NMR spectrum (176 MHz, $CDCl_3$).**

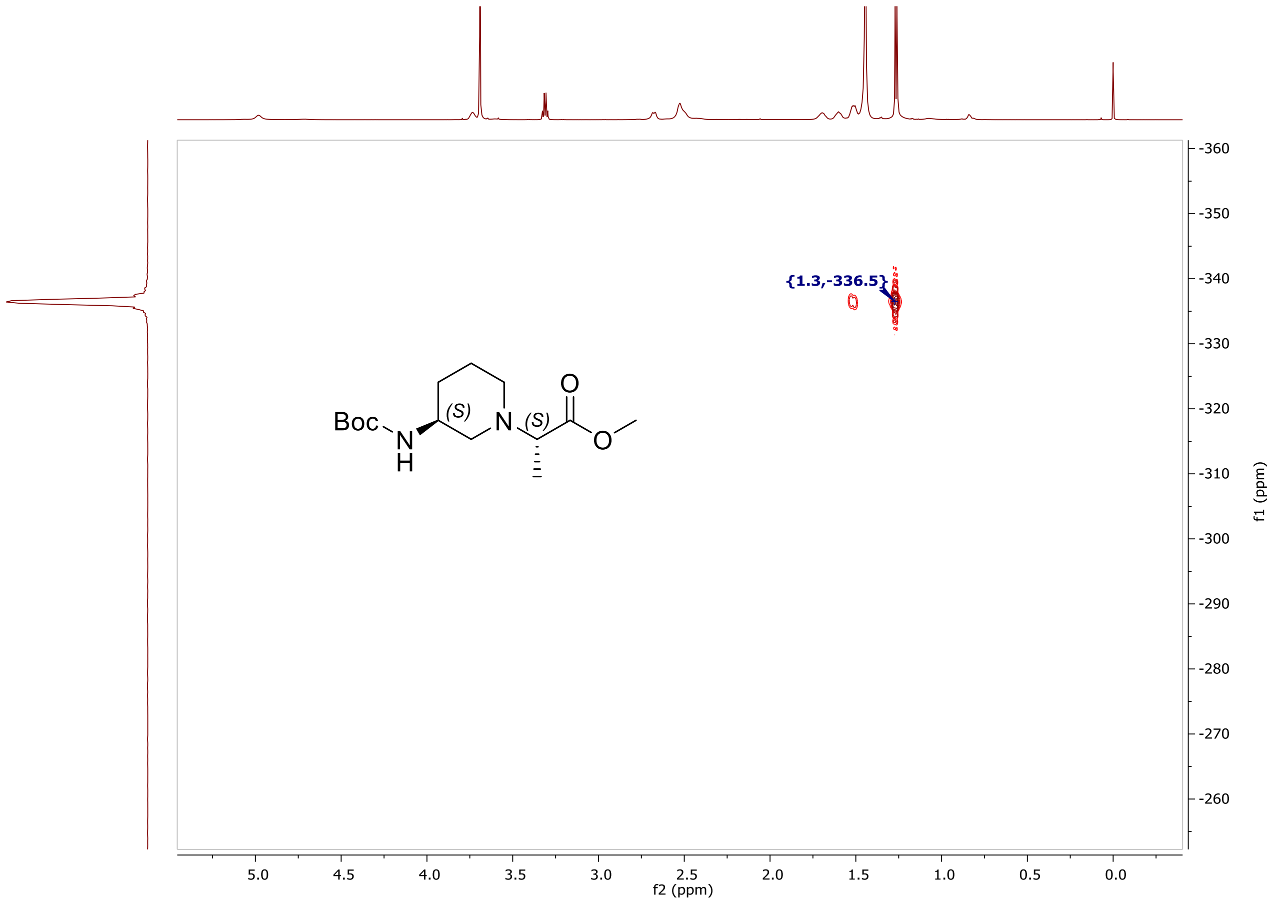


**Figure S64. Methyl (2*S*)-2-{(3*S*)-3-[(*tert*-butoxycarbonyl)amino]piperidin-1-yl}propanoate ((2*S*,3*S*)-10a). $^{1}H$-$^{15}N$ HMBC spectrum (71 MHz, $CDCl_3$).**

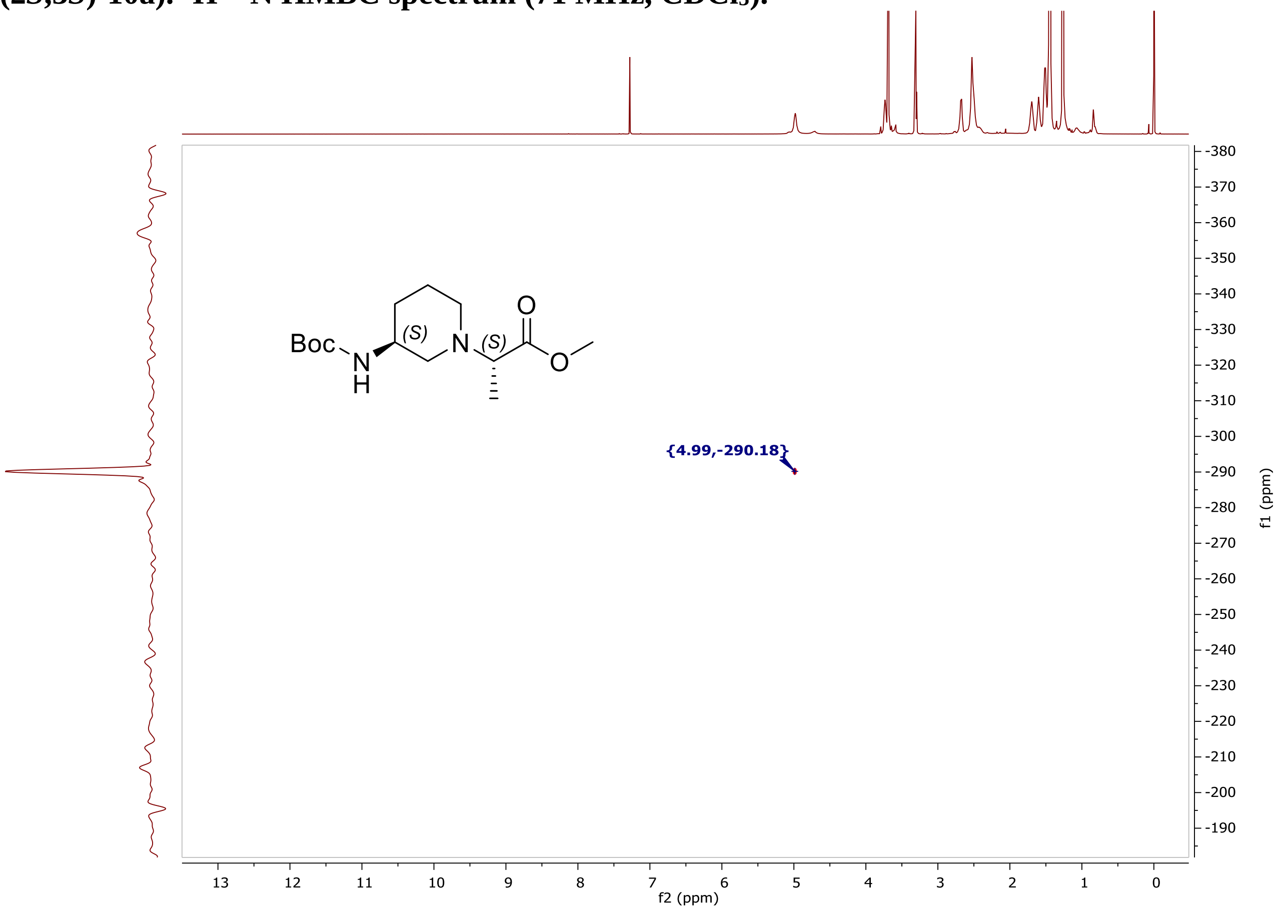


**Figure S65. Methyl (2*S*)-2-{(3*S*)-3-[(*tert*-butoxycarbonyl)amino]piperidin-1-yl}propanoate ((2*S*,3*S*)-10a). $^{1}H$-$^{15}N$ HSQC spectrum (71 MHz, $CDCl_3$).**

# Mass Spectrum SmartFormula Report

**Analysis Info**

| | | | |
|---|---|---|---|
| Analysis Name | D:\Data\Organikai\2023_04_11\GMP_372_1-D,2_01_10232.d | Acquisition Date | 4/21/2023 2:09:59 PM |
| Method | organikai_esi_pos_2013_recover.m | Operator | Milda Pukalskiene |
| Sample Name | GMP_372 | Instrument / Ser# | maXis 4G 20218 |
| Comment | | | |

**Acquisition Parameter**

| | | | | | |
|---|---|---|---|---|---|
| Source Type | ESI | Ion Polarity | Positive | Set Nebulizer | 1.5 Bar |
| Focus | Not active | Set Capillary | 4500 V | Set Dry Heater | 180 °C |
| Scan Begin | 40 m/z | Set End Plate Offset | -500 V | Set Dry Gas | 8.0 l/min |
| Scan End | 1800 m/z | Set Collision Cell RF | 350.0 Vpp | Set Divert Valve | Waste |

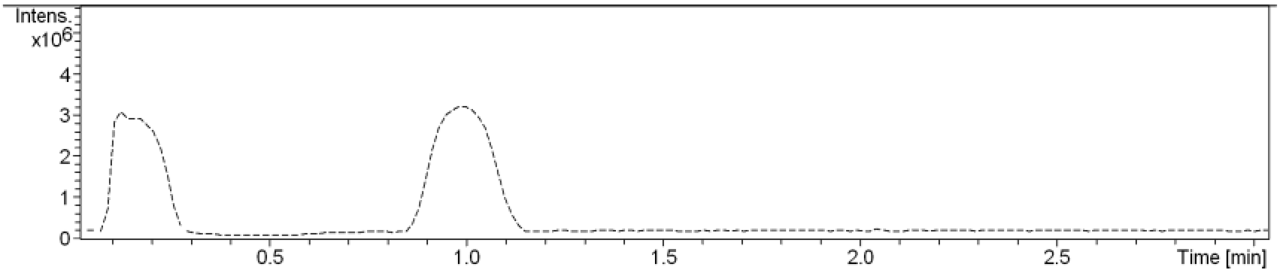


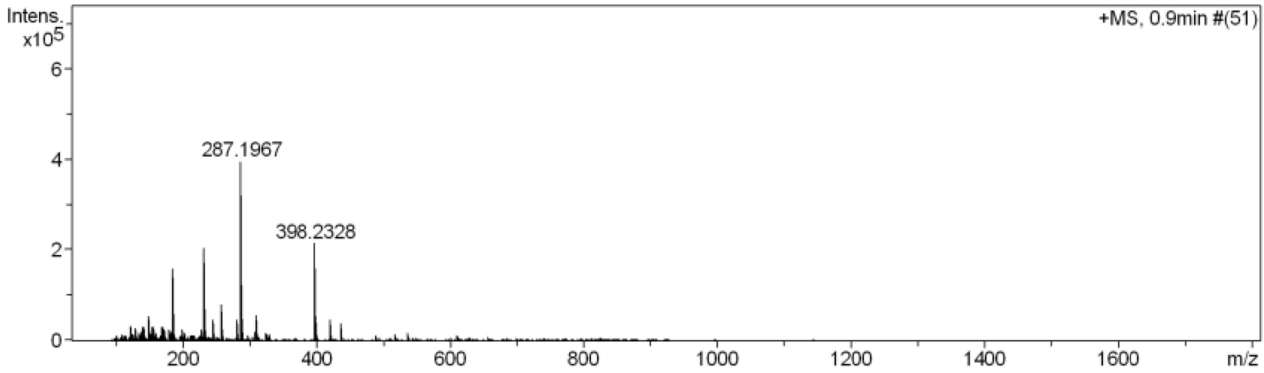


| Meas. m/z | # | Formula | Score | m/z | err [ppm] | Mean err [ppm] | mSigma | rdb | e⁻ Conf | N-Rule |
|---|---|---|---|---|---|---|---|---|---|---|
| 287.1967 | 1 | C 14 H 27 N 2 O 4 | 100.00 | 287.1965 | -0.6 | -0.5 | 13.0 | 2.5 | even | ok |

**Figure S66. Methyl (2*S*)-2-{(3*S*)-3-[(*tert*-butoxycarbonyl)amino]piperidin-1-yl}propanoate ((2*S*,3*S*)-10a). HRMS (ESI-TOF).**

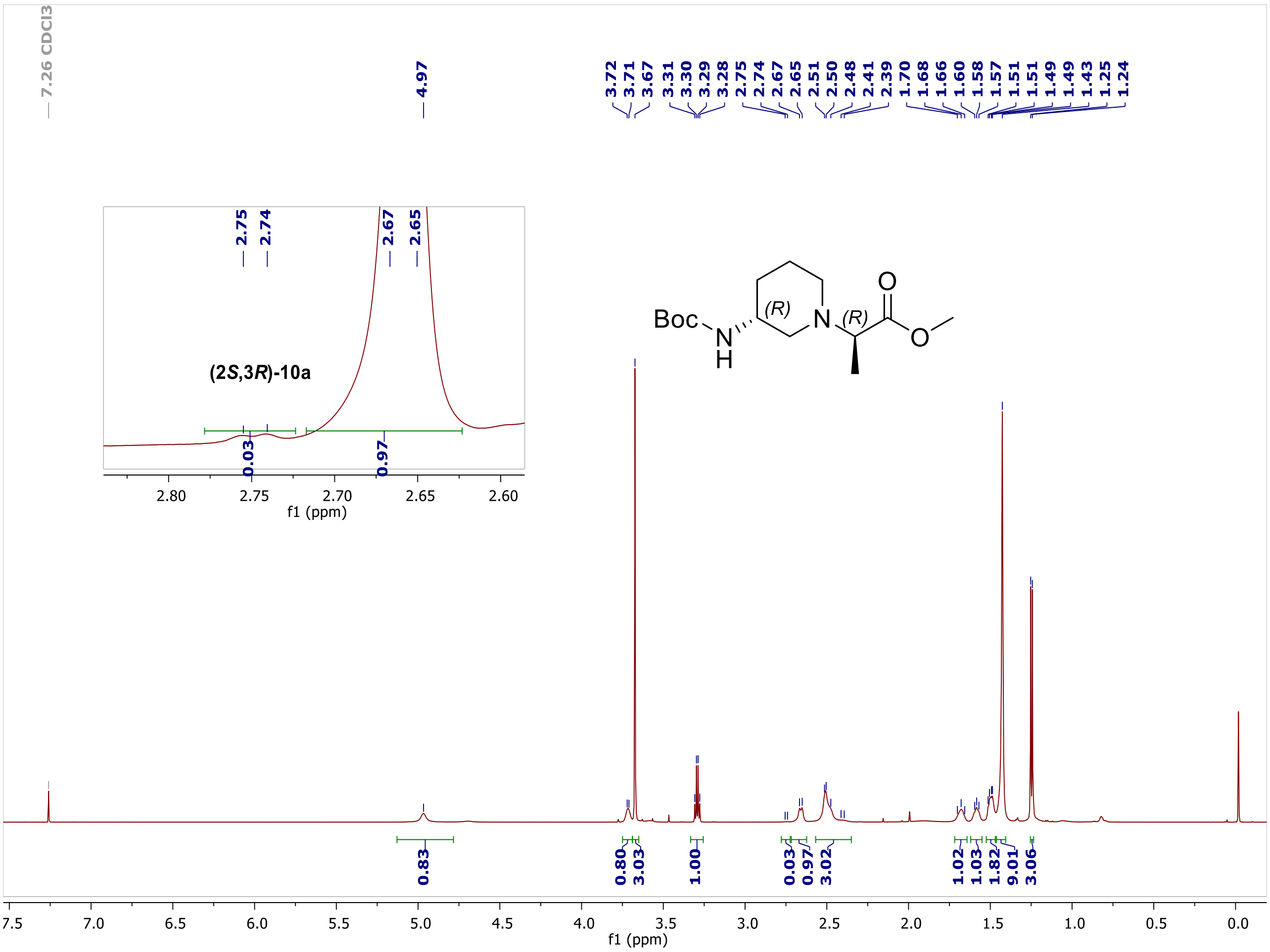


**Figure S67.** **Methyl (2*R*)-2-{(3*R*)-3-[(*tert*-butoxycarbonyl)amino]piperidin-1-yl}propanoate ((2*R*,3*R*)-10a). $^{1}$H NMR spectrum (700 MHz, $CDCl_3$).**

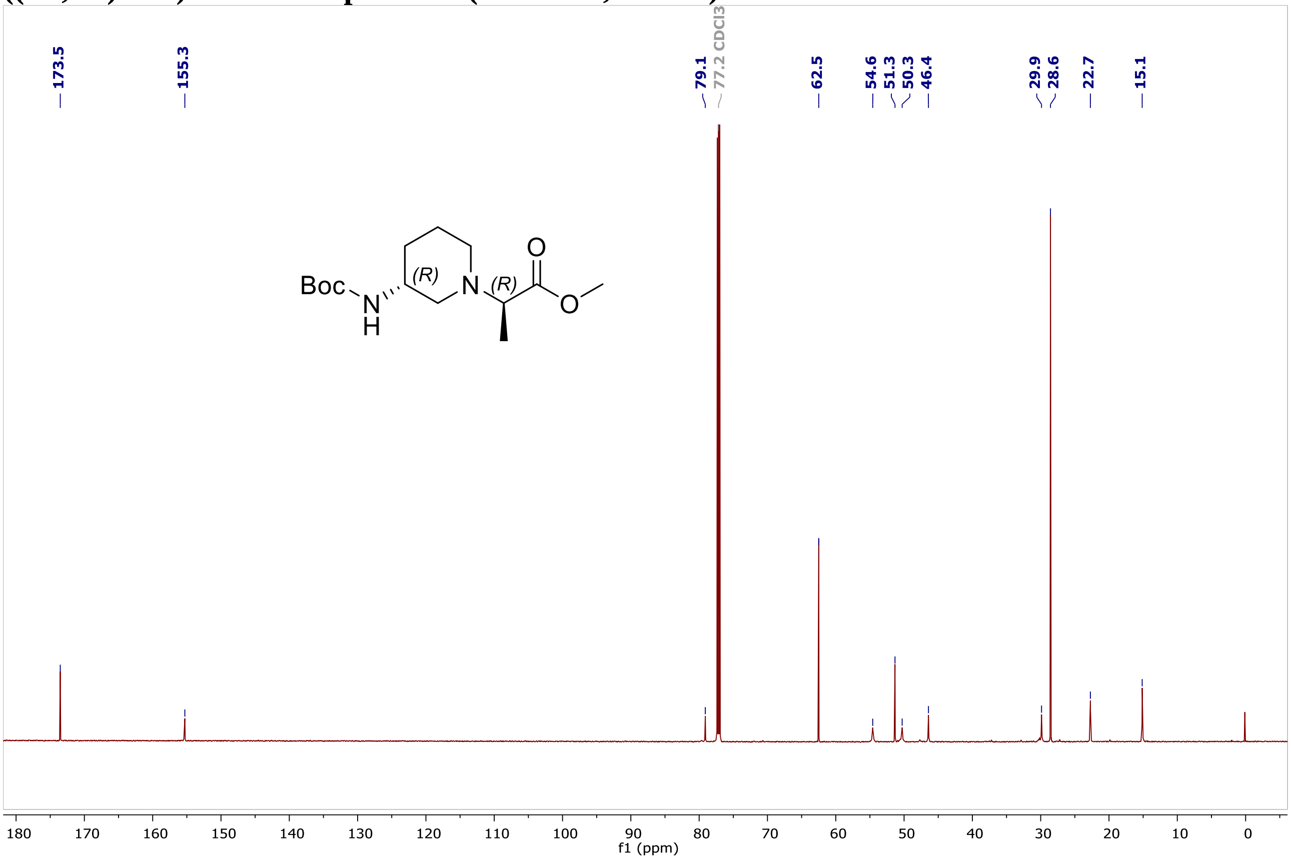


**Figure S68.** **Methyl (2*R*)-2-{(3*R*)-3-[(*tert*-butoxycarbonyl)amino]piperidin-1-yl}propanoate ((2*R*,3*R*)-10a). $^{13}$C NMR spectrum (176 MHz, $CDCl_3$).**

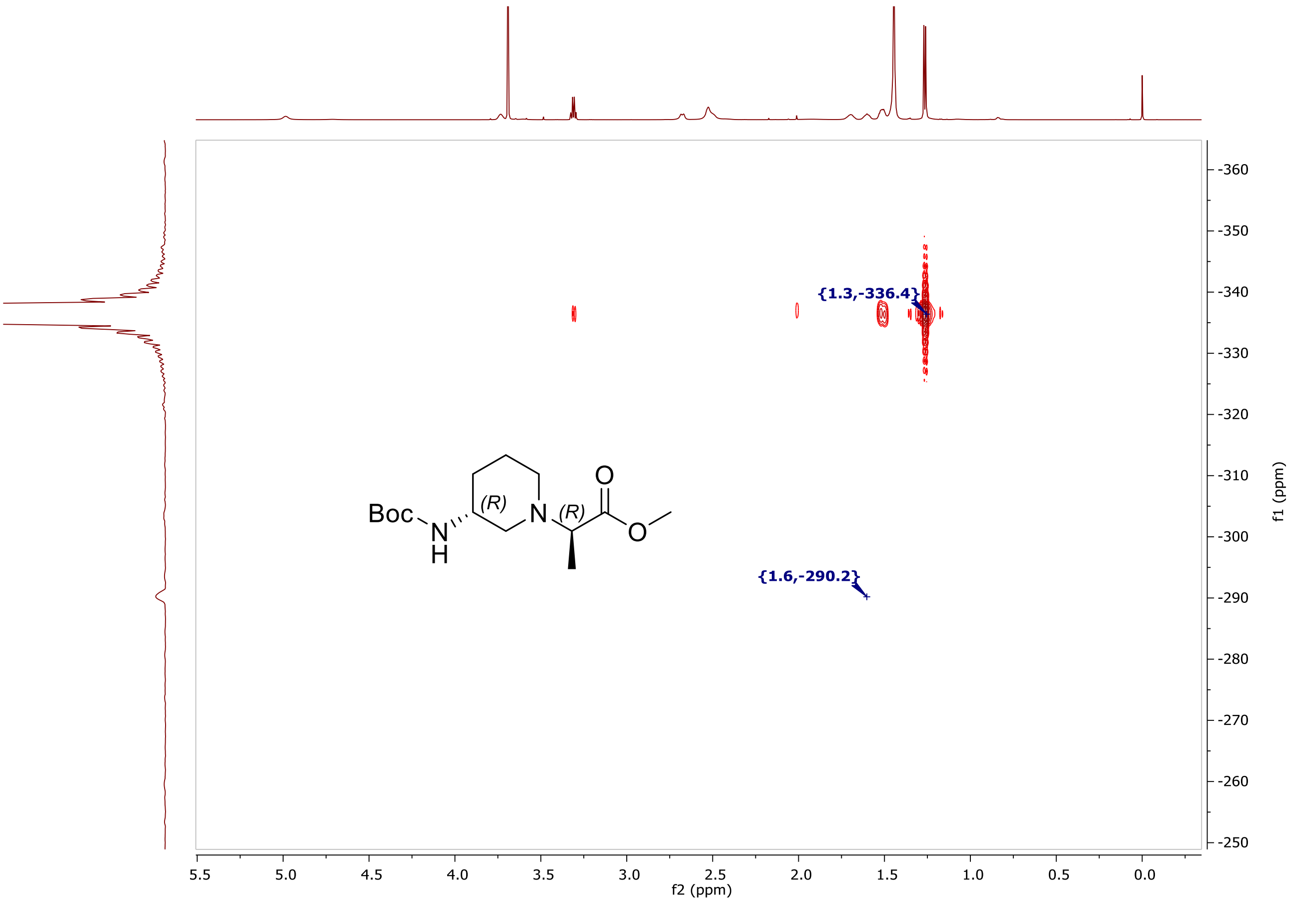


**Figure S69. Methyl (2*R*)-2-{(3*R*)-3-[(*tert*-butoxycarbonyl)amino]piperidin-1-yl}propanoate ((2*R*,3*R*)-10a). $^{1}H$-$^{15}N$ HMBC spectrum (71 MHz, $CDCl_3$).**

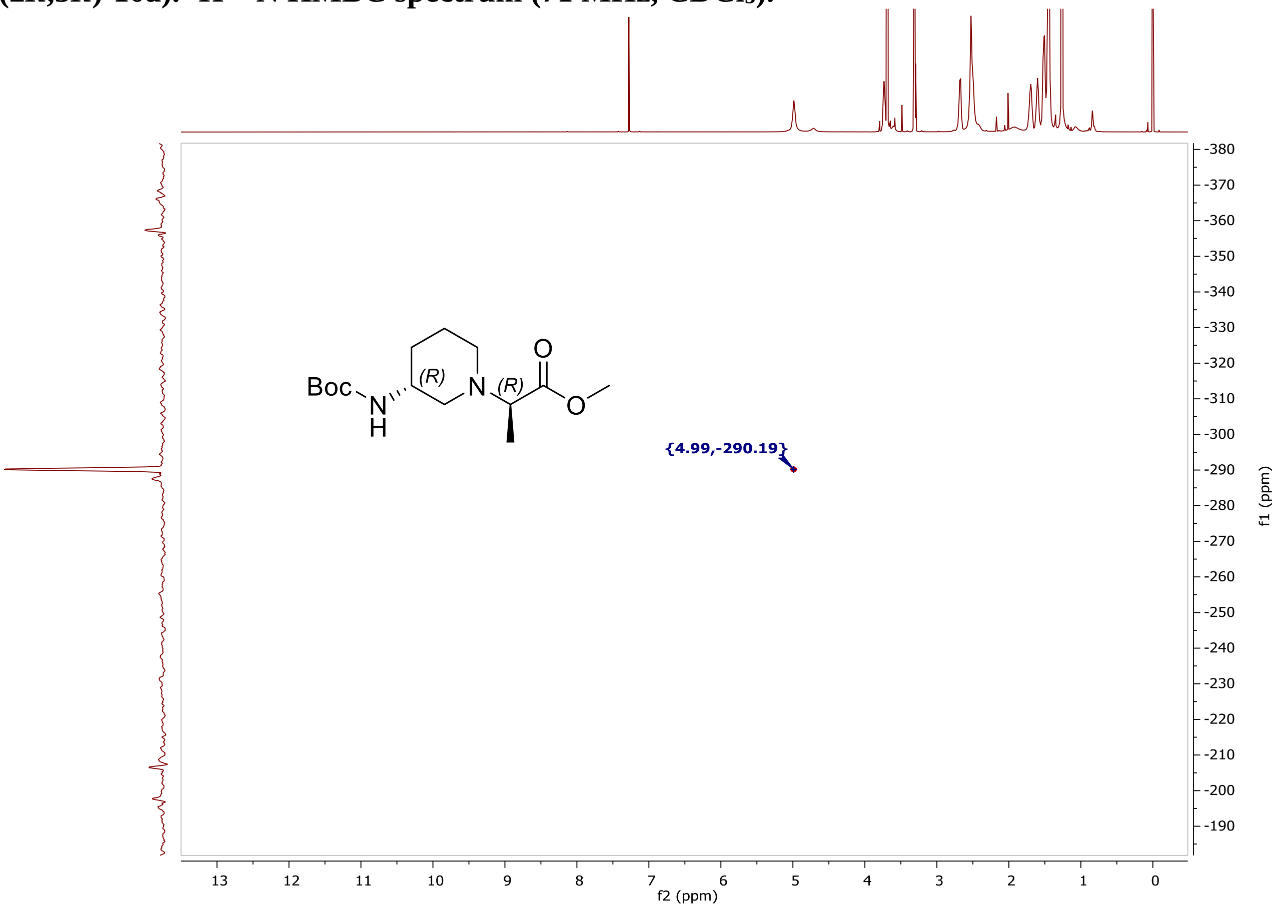


**Figure S70. Methyl (2*R*)-2-{(3*R*)-3-[(*tert*-butoxycarbonyl)amino]piperidin-1-yl}propanoate ((2*R*,3*R*)-10a). $^{1}H$-$^{15}N$ HSQC spectrum (71 MHz, $CDCl_3$).**

# Mass Spectrum SmartFormula Report

**Analysis Info**

| | | | |
|---|---|---|---|
| Analysis Name | D:\Data\Organikai\2023_04_11\GMP_379_1-D,1_01_10231.d | Acquisition Date | 4/21/2023 2:05:33 PM |
| Method | organikai_esi_pos_2013_recover.m | Operator | Milda Pukalskiene |
| Sample Name | GMP_379 | Instrument / Ser# | maXis 4G 20218 |
| Comment | | | |

**Acquisition Parameter**

| | | | | | |
|---|---|---|---|---|---|
| Source Type | ESI | Ion Polarity | Positive | Set Nebulizer | 1.5 Bar |
| Focus | Not active | Set Capillary | 4500 V | Set Dry Heater | 180 °C |
| Scan Begin | 40 m/z | Set End Plate Offset | -500 V | Set Dry Gas | 8.0 l/min |
| Scan End | 1800 m/z | Set Collision Cell RF | 350.0 Vpp | Set Divert Valve | Waste |

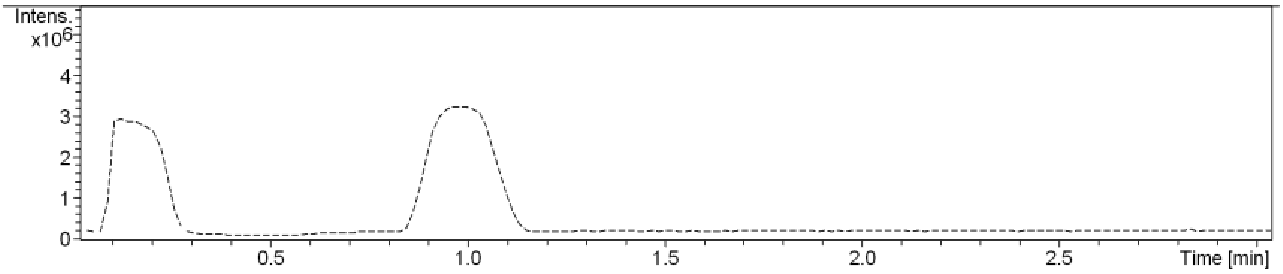


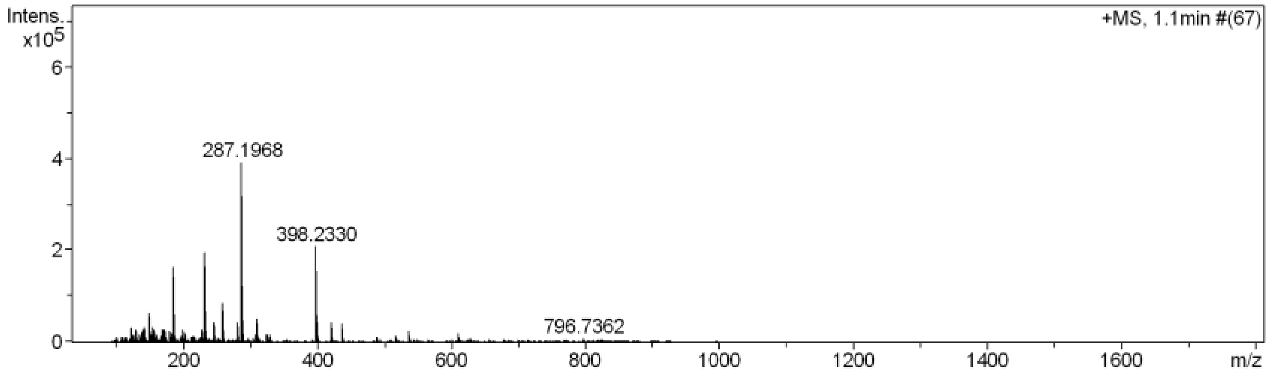


| Meas. m/z | # | Formula | Score | m/z | err [ppm] | Mean err [ppm] | mSigma | rdb | e⁻ Conf | N-Rule |
|---|---|---|---|---|---|---|---|---|---|---|
| 287.1968 | 1 | C 14 H 27 N 2 O 4 | 100.00 | 287.1965 | -1.0 | -0.9 | 13.4 | 2.5 | even | ok |

**Figure S71. Methyl (2*R*)-2-{(3*R*)-3-[(*tert*-butoxycarbonyl)amino]piperidin-1-yl}propanoate ((2*R*,3*R*)-10a). HRMS (ESI-TOF).**

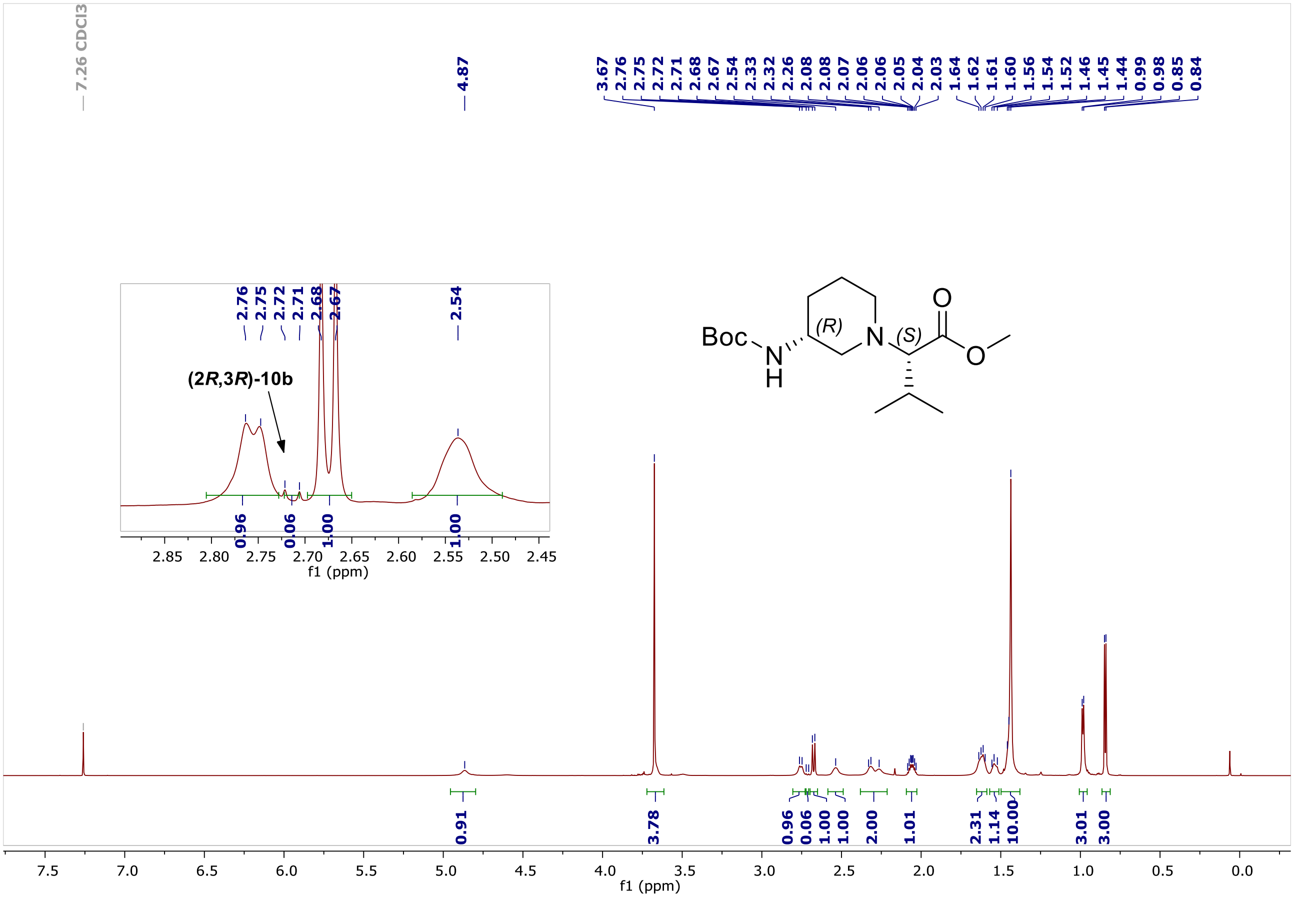

**Figure S72. Methyl (2*S*)-2-{(3*R*)-3-[(*tert*-butoxycarbonyl)amino]piperidin-1-yl}-3-methylbutanoate ((2*S*,3*R*)-10b). $^{1}$H NMR spectrum (700 MHz, $CDCl_3$).**

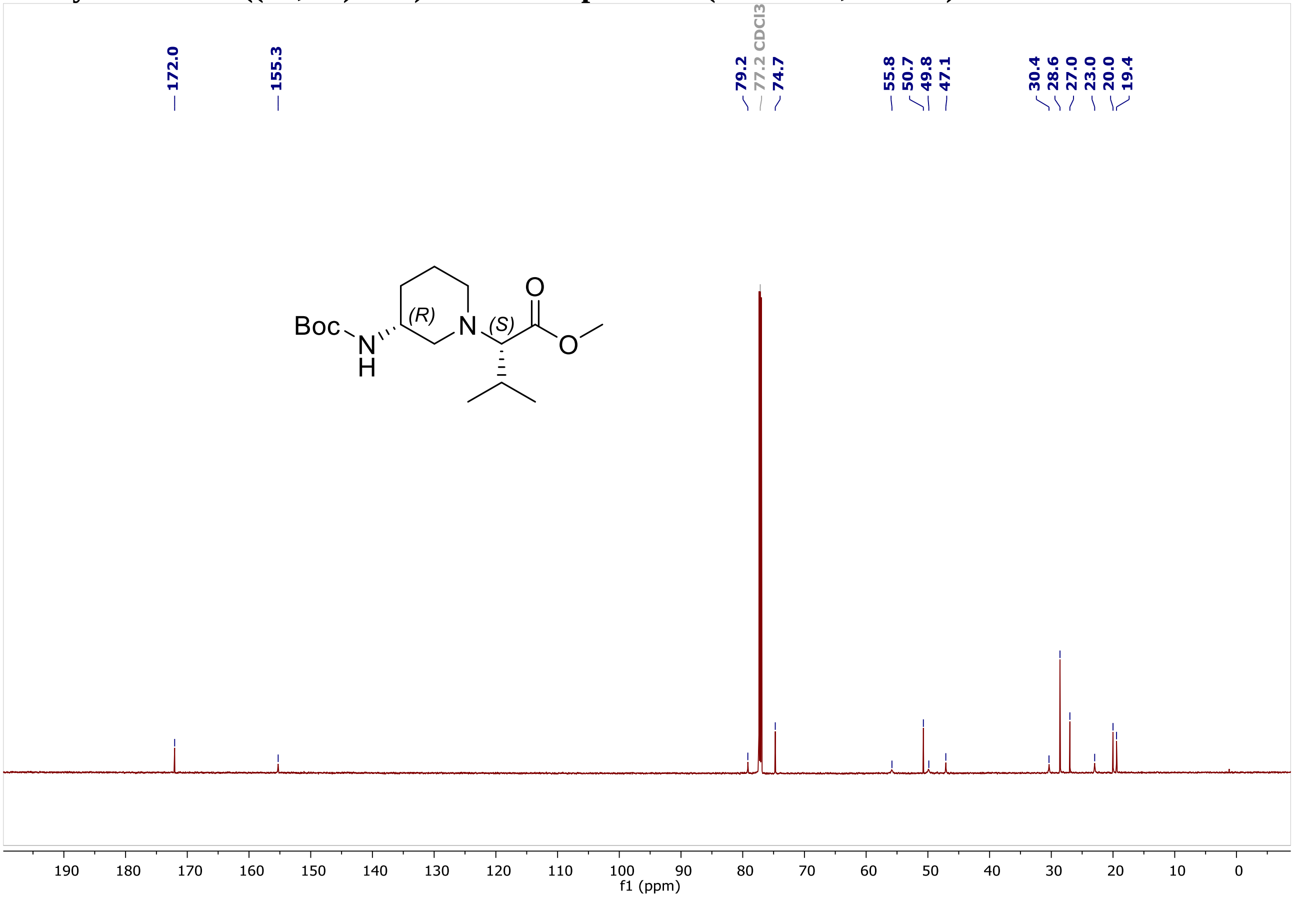

**Figure S73. Methyl (2*S*)-2-{(3*R*)-3-[(*tert*-butoxycarbonyl)amino]piperidin-1-yl}-3-methylbutanoate ((2*S*,3*R*)-10b). $^{13}$C NMR spectrum (176 MHz, $CDCl_3$).**

# Mass Spectrum SmartFormula Report

| | | | |
|---|---|---|---|
| **Analysis Info** | | Acquisition Date | 4/14/2023 1:10:31 PM |
| Analysis Name | D:\Data\Organikai\2023_04_11\GMP_574_1-B,1_01_10214.d | | |
| Method | organikai_esi_pos_2013_recover.m | Operator | Milda Pukalskiene |
| Sample Name | GMP_574 | Instrument / Ser# | maXis 4G 20218 |
| Comment | | | |

**Acquisition Parameter**

| | | | | | |
|---|---|---|---|---|---|
| Source Type | ESI | Ion Polarity | Positive | Set Nebulizer | 1.5 Bar |
| Focus | Not active | Set Capillary | 4500 V | Set Dry Heater | 180 °C |
| Scan Begin | 40 m/z | Set End Plate Offset | -500 V | Set Dry Gas | 8.0 l/min |
| Scan End | 1800 m/z | Set Collision Cell RF | 350.0 Vpp | Set Divert Valve | Waste |

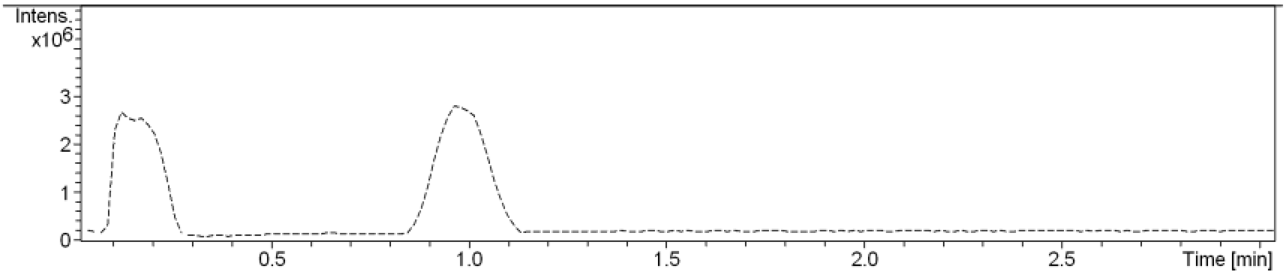


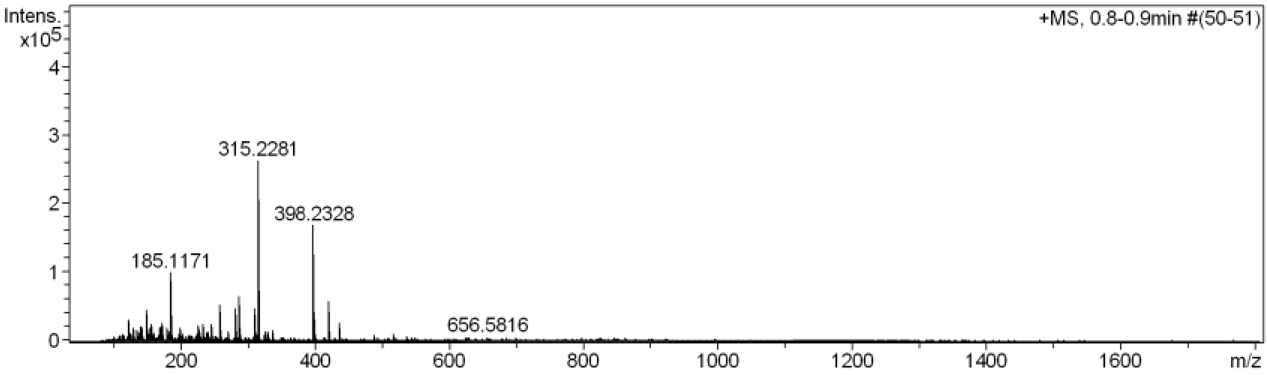


| Meas. m/z | # | Formula | Score | m/z | err [ppm] | Mean err [ppm] | mSigma | rdb | e⁻ Conf | N-Rule |
|---|---|---|---|---|---|---|---|---|---|---|
| 315.2281 | 1 | C 16 H 31 N 2 O 4 | 100.00 | 315.2278 | -0.9 | -0.5 | 9.9 | 2.5 | even | ok |

**Figure S74. Methyl (2*S*)-2-{(3*R*)-3-[(*tert*-butoxycarbonyl)amino]piperidin-1-yl}-3-methylbutanoate ((2*S*,3*R*)-10b). HRMS (ESI-TOF).**

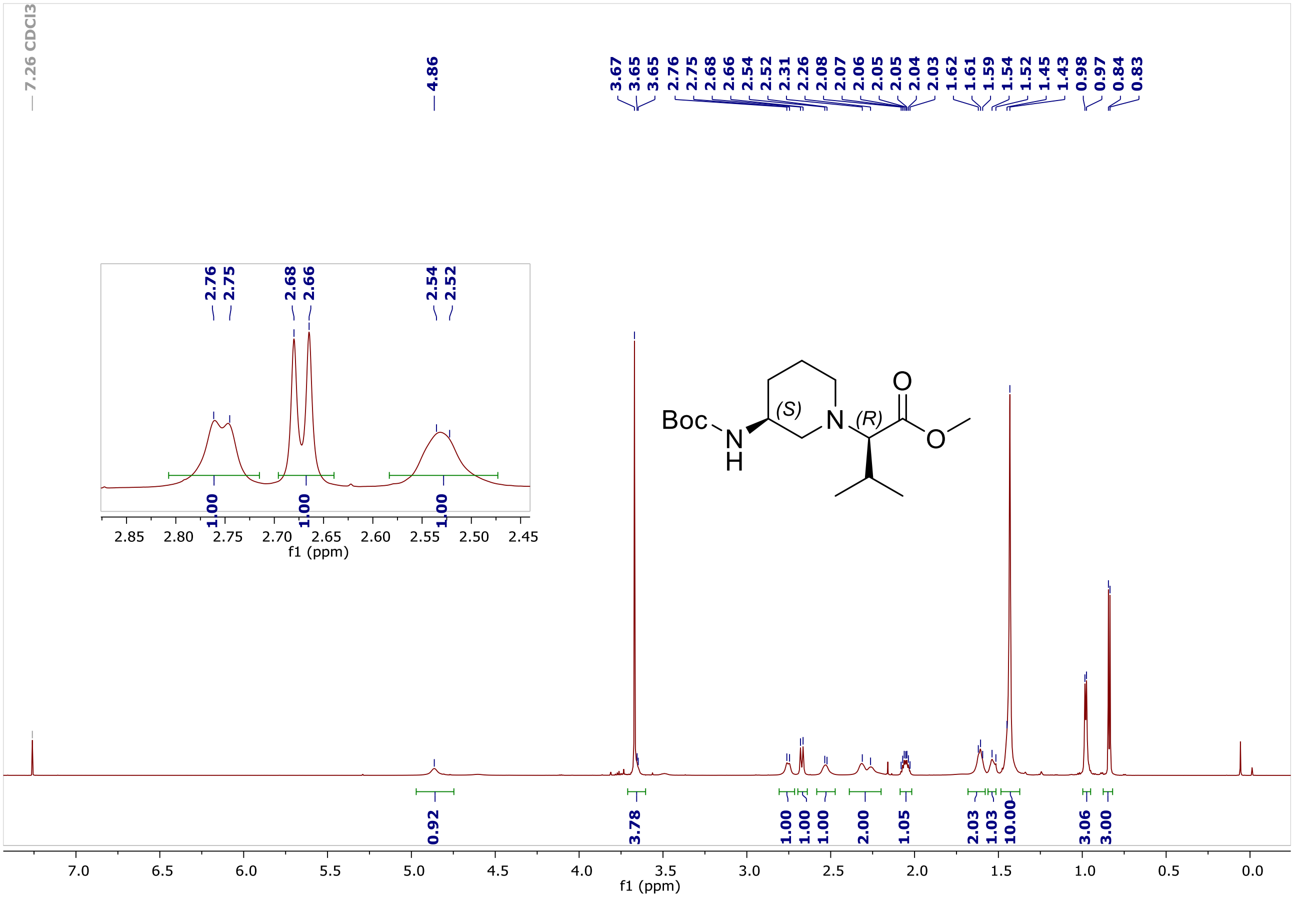

**Figure S75. Methyl (2*R*)-2-{(3*S*)-3-[(*tert*-butoxycarbonyl)amino]piperidin-1-yl}-3-methylbutanoate ((2*R*,3*S*)-10b). $^{1}$H NMR spectrum (700 MHz, $CDCl_3$).**

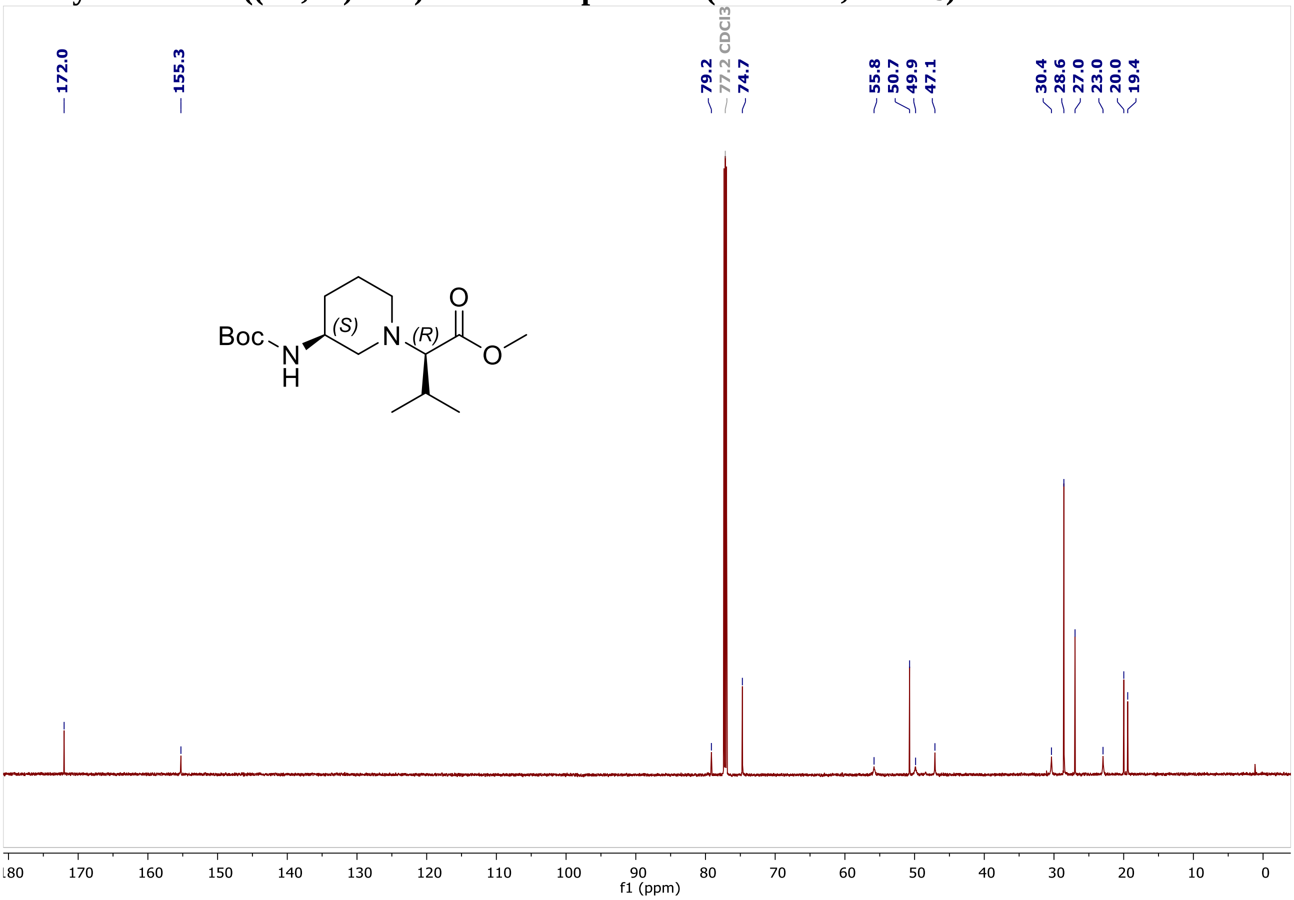

**Figure S76. Methyl (2*R*)-2-{(3*S*)-3-[(*tert*-butoxycarbonyl)amino]piperidin-1-yl}-3-methylbutanoate ((2*R*,3*S*)-10b). $^{13}$C NMR spectrum (176 MHz, $CDCl_3$).**

# Mass Spectrum SmartFormula Report

**Analysis Info**

| | | | |
|---|---|---|---|
| Analysis Name | D:\Data\Organikai\2023_04_11\GMP_564_1-A,6_01_10211.d | Acquisition Date | 4/14/2023 12:57:05 PM |
| Method | organikai_esi_pos_2013_recover.m | Operator | Milda Pukalskiene |
| Sample Name | GMP_564 | Instrument / Ser# | maXis 4G 20218 |
| Comment | | | |

**Acquisition Parameter**

| | | | | | |
|---|---|---|---|---|---|
| Source Type | ESI | Ion Polarity | Positive | Set Nebulizer | 1.5 Bar |
| Focus | Not active | Set Capillary | 4500 V | Set Dry Heater | 180 °C |
| Scan Begin | 40 m/z | Set End Plate Offset | -500 V | Set Dry Gas | 8.0 l/min |
| Scan End | 1800 m/z | Set Collision Cell RF | 350.0 Vpp | Set Divert Valve | Waste |

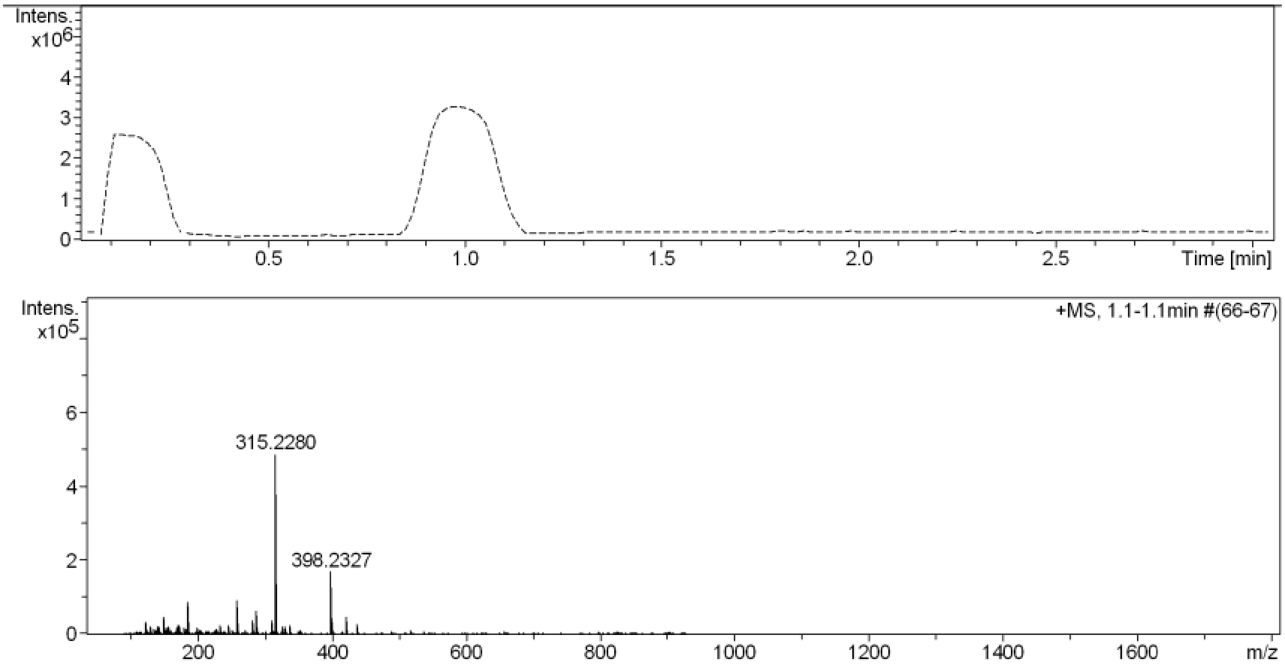


| Meas. m/z | # | Formula | Score | m/z | err [ppm] | Mean err [ppm] | mSigma | rdb | e⁻ Conf | N-Rule |
|---|---|---|---|---|---|---|---|---|---|---|
| 315.2280 | 1 | C 16 H 31 N 2 O 4 | 100.00 | 315.2278 | -0.7 | -0.4 | 14.0 | 2.5 | even | ok |

**Figure S77. Methyl (2*R*)-2-{(3*S*)-3-[(*tert*-butoxycarbonyl)amino]piperidin-1-yl}-3-methylbutanoate ((2*R*,3*S*)-10b). HRMS (ESI-TOF).**

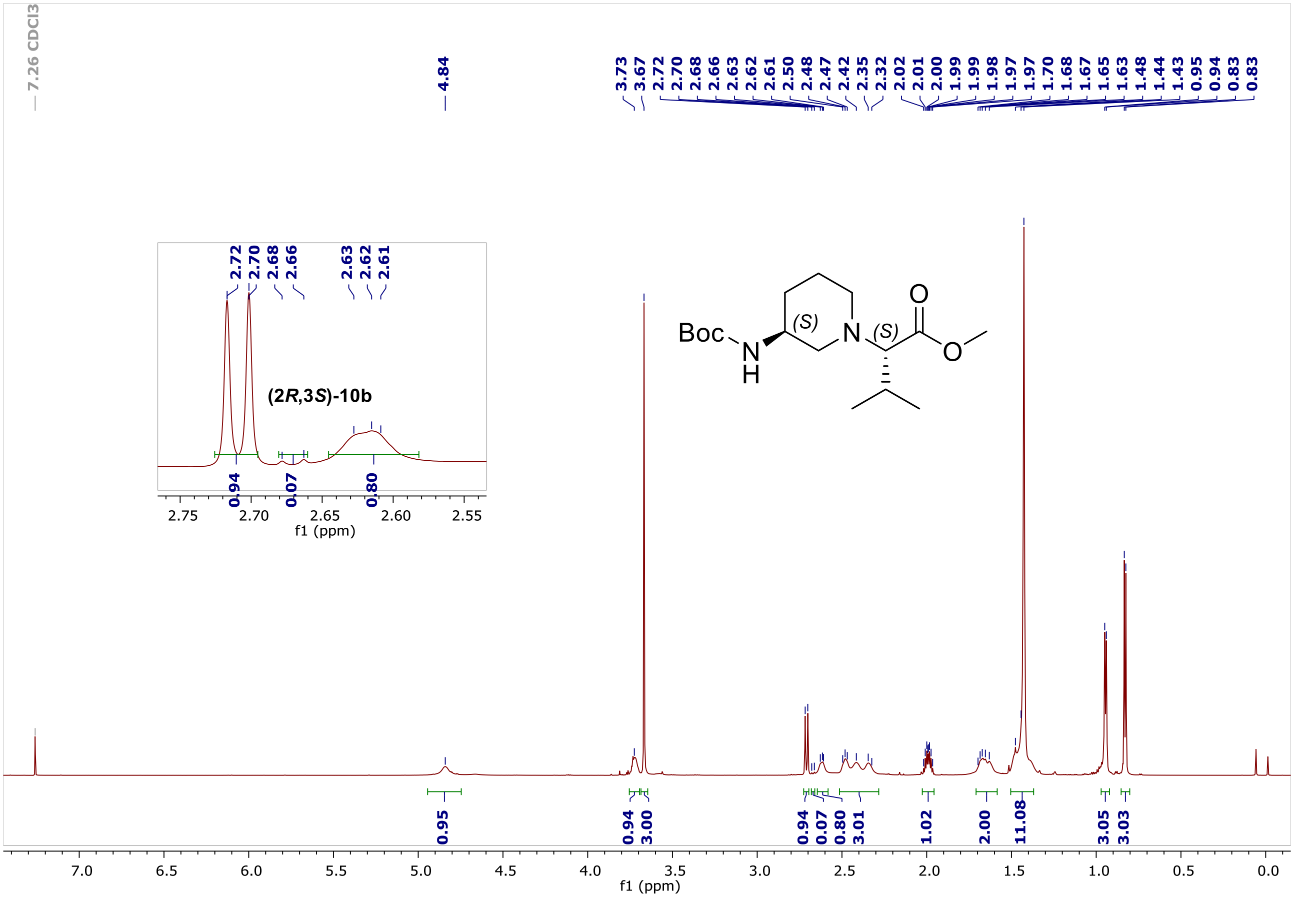

**Figure S78. Methyl (2*S*)-2-{(3*S*)-3-[(*tert*-butoxycarbonyl)amino]piperidin-1-yl}-3-methylbutanoate ((2*S*,3*S*)-10b). $^{1}$H NMR spectrum (700 MHz, $CDCl_3$).**

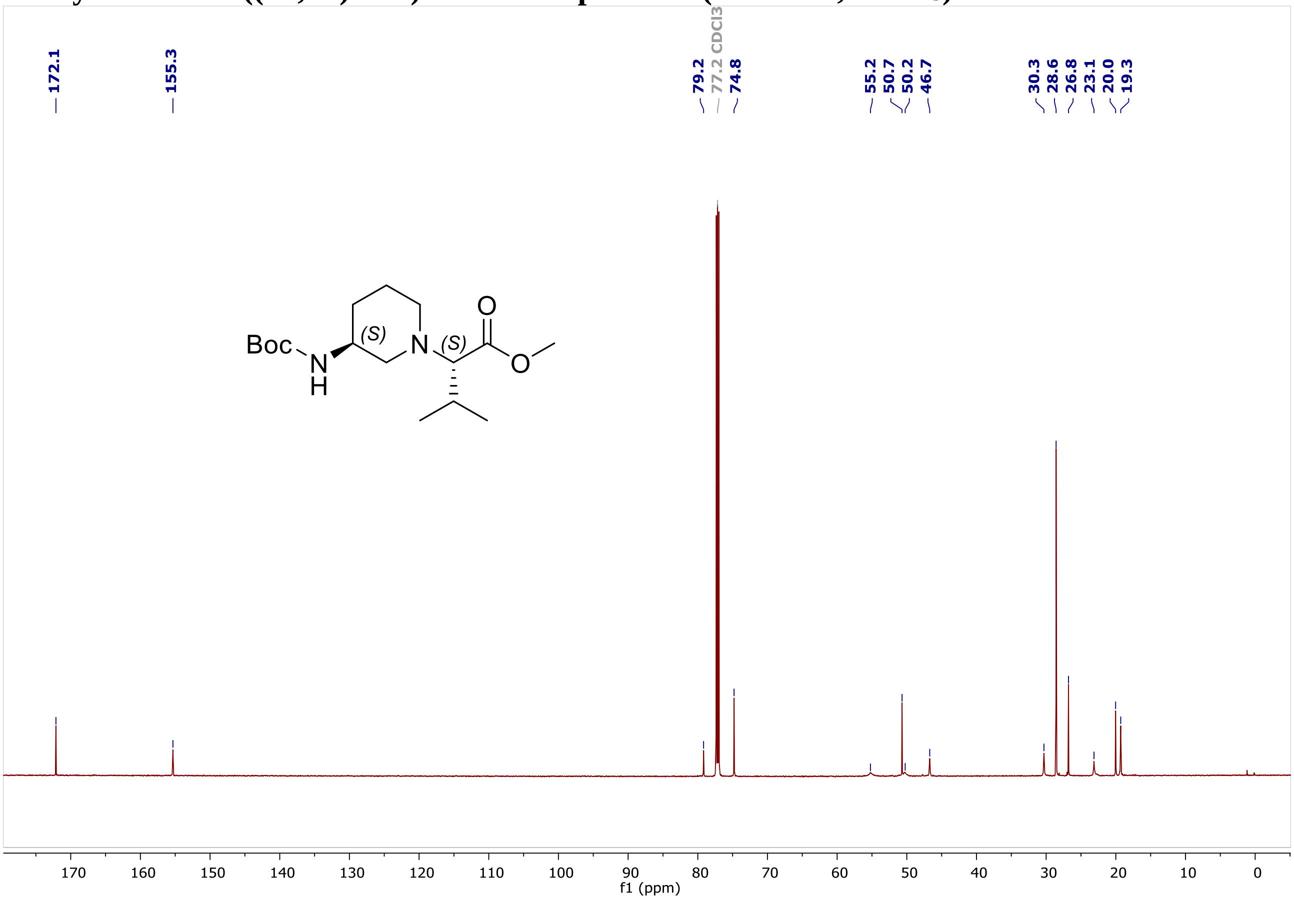

**Figure S79. Methyl (2*S*)-2-{(3*S*)-3-[(*tert*-butoxycarbonyl)amino]piperidin-1-yl}-3-methylbutanoate ((2*S*,3*S*)-10b). $^{13}$C NMR spectrum (176 MHz, $CDCl_3$).**

# Mass Spectrum SmartFormula Report

**Analysis Info**

| | | | |
|---|---|---|---|
| Analysis Name | D:\Data\Organikai\2023_04_11\GMP_567_1-A,7_01_10212.d | Acquisition Date | 4/14/2023 1:01:35 PM |
| Method | organikai_esi_pos_2013_recover.m | Operator | Milda Pukalskiene |
| Sample Name | GMP_567 | Instrument / Ser# | maXis 4G 20218 |
| Comment | | | |

**Acquisition Parameter**

| | | | | | |
|---|---|---|---|---|---|
| Source Type | ESI | Ion Polarity | Positive | Set Nebulizer | 1.5 Bar |
| Focus | Not active | Set Capillary | 4500 V | Set Dry Heater | 180 °C |
| Scan Begin | 40 m/z | Set End Plate Offset | -500 V | Set Dry Gas | 8.0 l/min |
| Scan End | 1800 m/z | Set Collision Cell RF | 350.0 Vpp | Set Divert Valve | Waste |

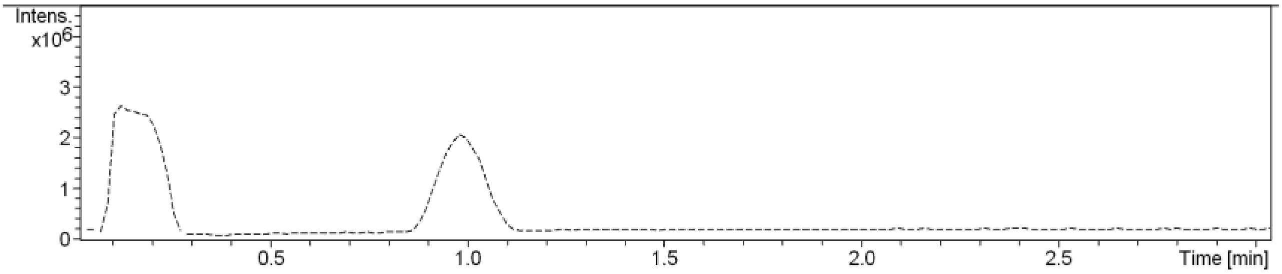


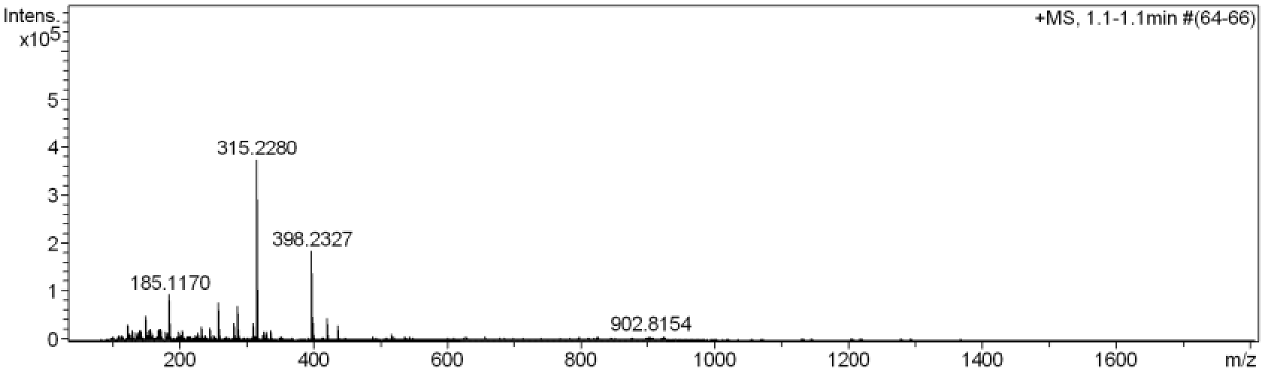


| Meas. m/z | # | Formula | Score | m/z | err [ppm] | Mean err [ppm] | mSigma | rdb | e⁻ Conf | N-Rule |
|---|---|---|---|---|---|---|---|---|---|---|
| 315.2280 | 1 | C 16 H 31 N 2 O 4 | 100.00 | 315.2278 | -0.6 | -0.3 | 19.2 | 2.5 | even | ok |

**Figure S80. Methyl (2*S*)-2-{(3*S*)-3-[(*tert*-butoxycarbonyl)amino]piperidin-1-yl}-3-methylbutanoate ((2*S*,3*S*)-10b). HRMS (ESI-TOF).**

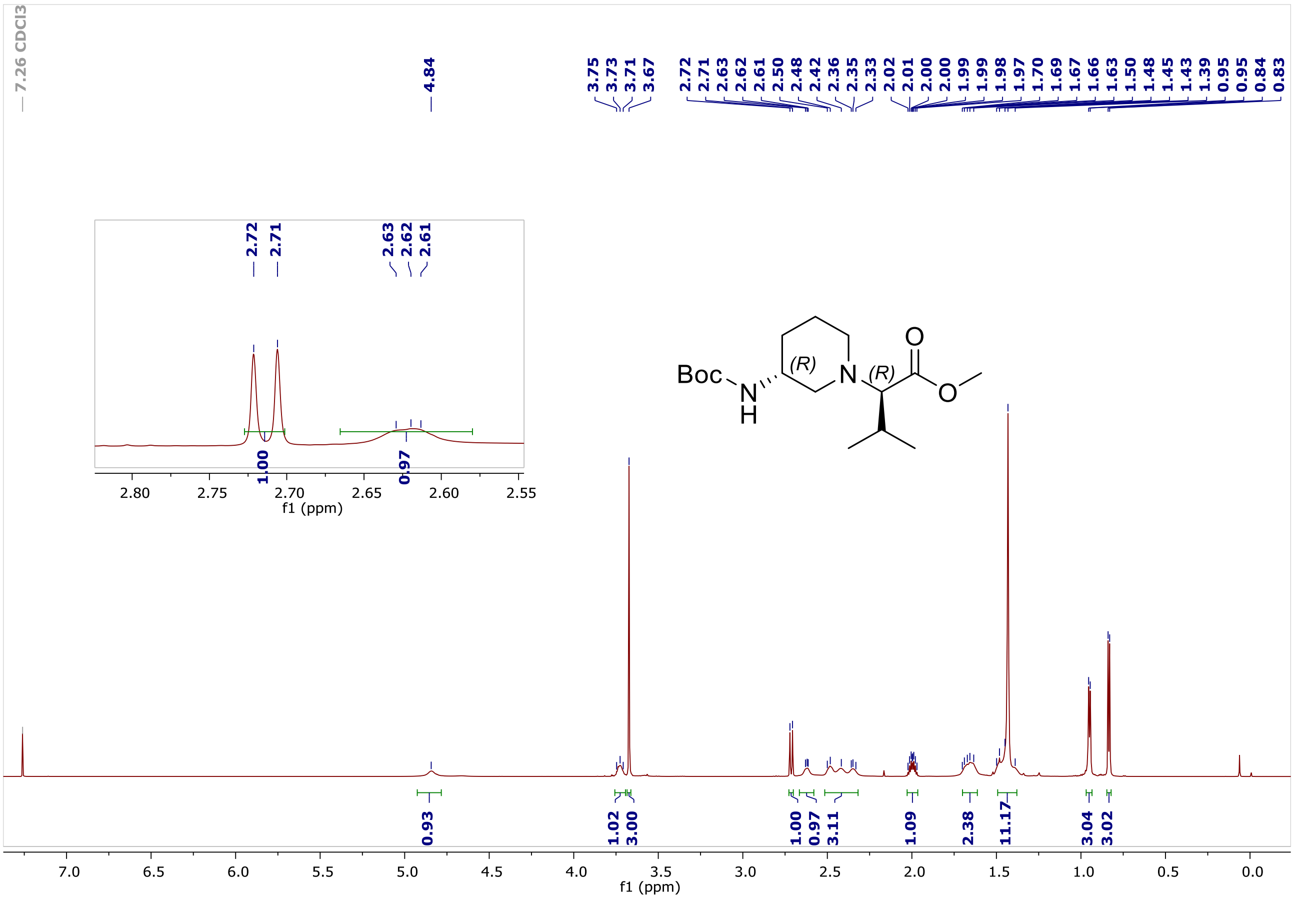

**Figure S81. Methyl (2*R*)-2-{(3*R*)-3-[(*tert*-butoxycarbonyl)amino]piperidin-1-yl}-3-methylbutanoate ((2*R*,3*R*)-10b).** $^{1}H$ **NMR spectrum (700 MHz,** $CDCl_3$**).**

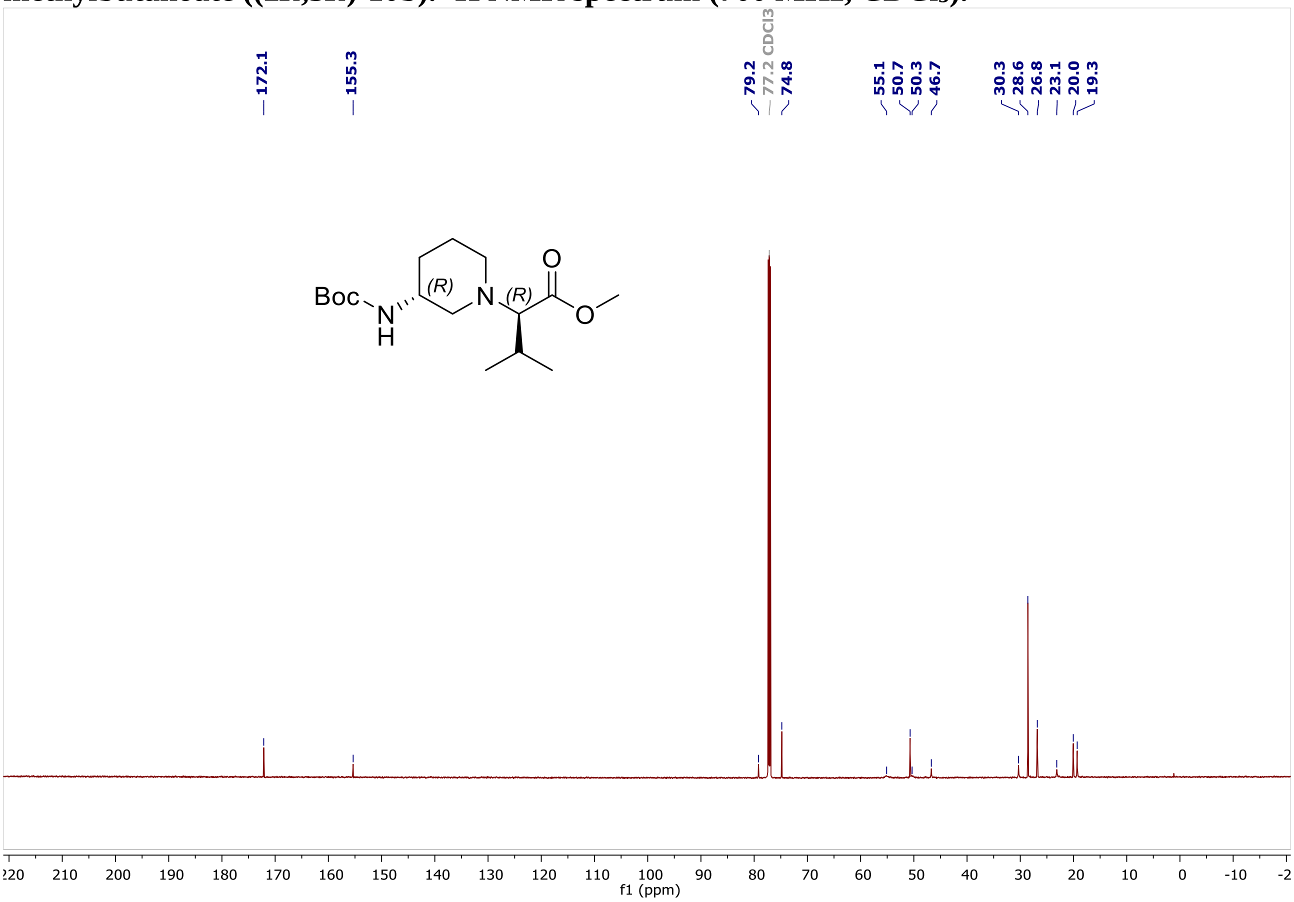

**Figure S82. Methyl (2*R*)-2-{(3*R*)-3-[(*tert*-butoxycarbonyl)amino]piperidin-1-yl}-3-methylbutanoate ((2*R*,3*R*)-10b).** $^{13}C$ **NMR spectrum (176 MHz,** $CDCl_3$**).**

# Mass Spectrum SmartFormula Report

**Analysis Info**

| | | | |
|---|---|---|---|
| Analysis Name | D:\Data\Organikai\2023_04_11\GMP_570_1-A,8_01_10213.d | Acquisition Date | 4/14/2023 1:06:04 PM |
| Method | organikai_esi_pos_2013_recover.m | Operator | Milda Pukalskiene |
| Sample Name | GMP_570 | Instrument / Ser# | maXis 4G 20218 |
| Comment | | | |

**Acquisition Parameter**

| | | | | | |
|---|---|---|---|---|---|
| Source Type | ESI | Ion Polarity | Positive | Set Nebulizer | 1.5 Bar |
| Focus | Not active | Set Capillary | 4500 V | Set Dry Heater | 180 °C |
| Scan Begin | 40 m/z | Set End Plate Offset | -500 V | Set Dry Gas | 8.0 l/min |
| Scan End | 1800 m/z | Set Collision Cell RF | 350.0 Vpp | Set Divert Valve | Waste |

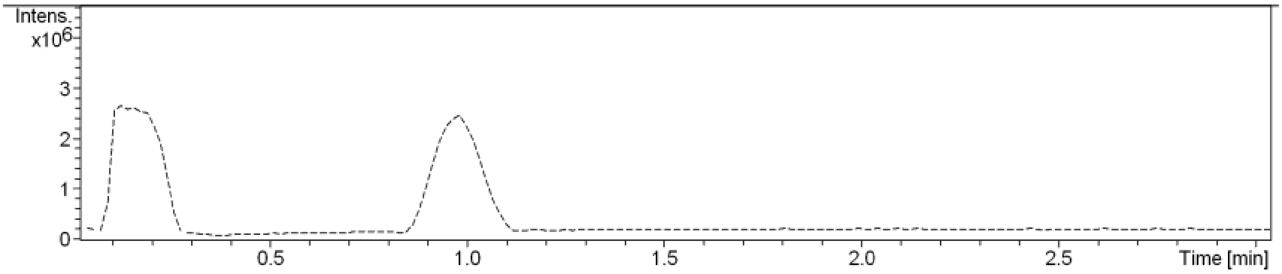


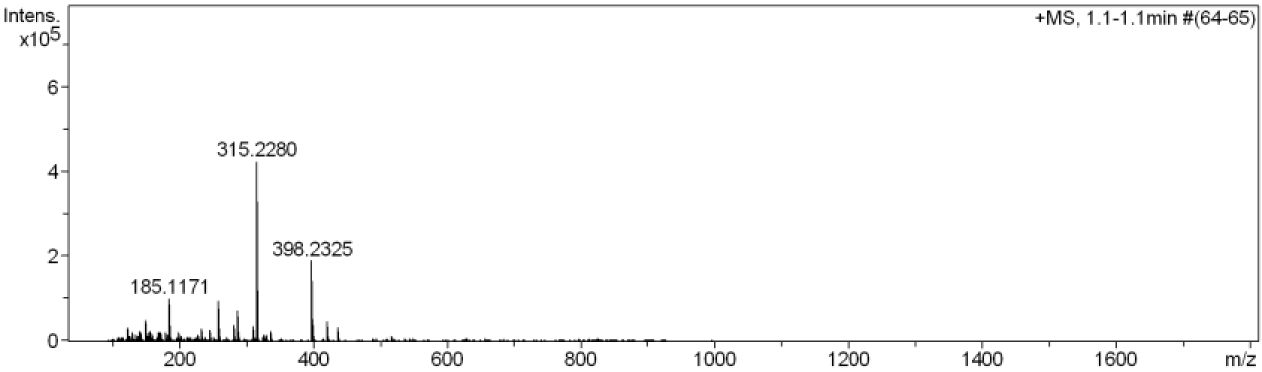


| Meas. m/z | # | Formula | Score | m/z | err [ppm] | Mean err [ppm] | mSigma | rdb | e⁻ Conf | N-Rule |
|---|---|---|---|---|---|---|---|---|---|---|
| 315.2280 | 1 | C 16 H 31 N 2 O 4 | 100.00 | 315.2278 | -0.6 | -0.3 | 11.3 | 2.5 | even | ok |

**Figure S83. Methyl (2*R*)-2-{(3*R*)-3-[(*tert*-butoxycarbonyl)amino]piperidin-1-yl}-3-methylbutanoate ((2*R*,3*R*)-10b). HRMS (ESI-TOF).**

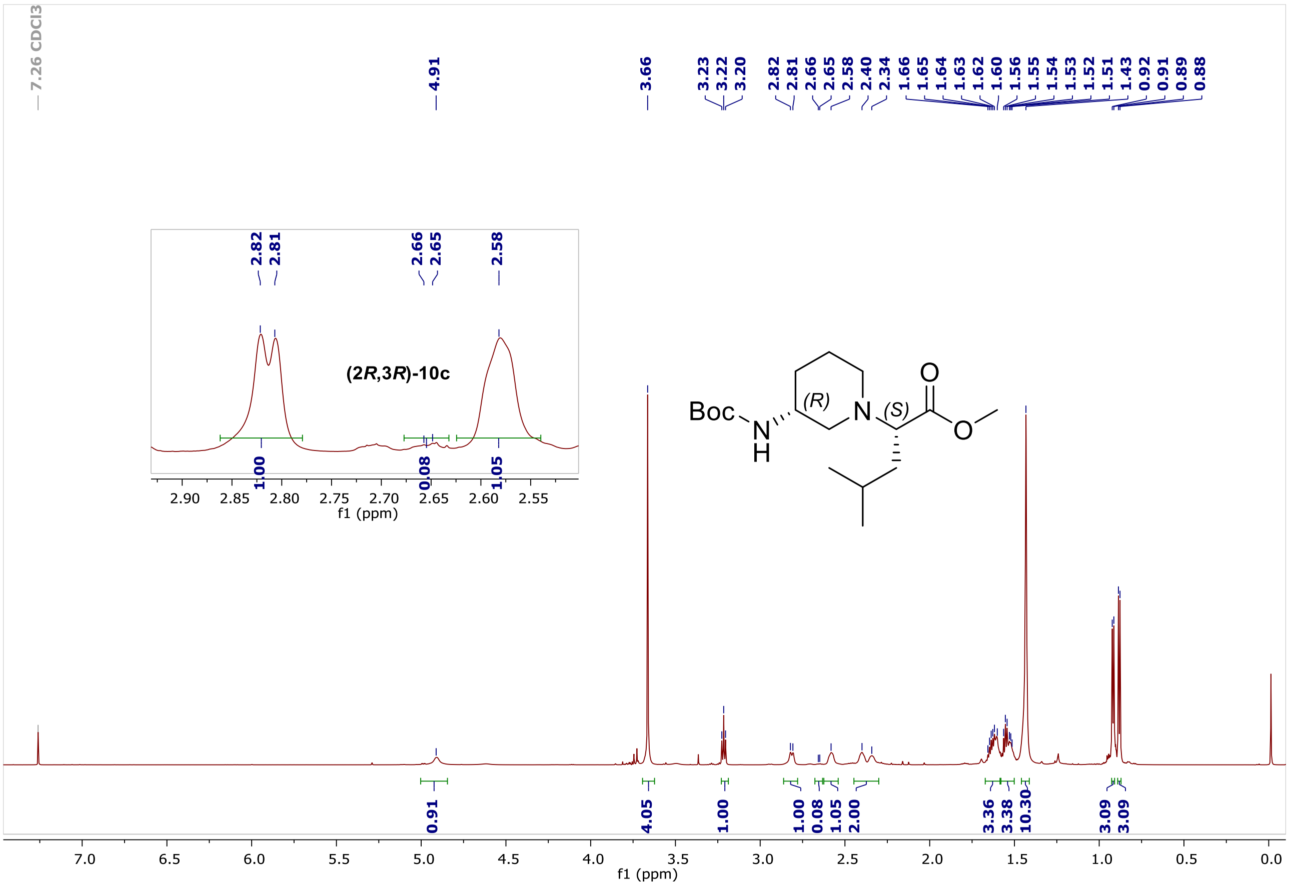

**Figure S84. Methyl (2*S*)-2-{(3*R*)-3-[(*tert*-butoxycarbonyl)amino]piperidin-1-yl}-4-methylpentanoate ((2*S*,3*R*)-10c). $^{1}$H NMR spectrum (700 MHz, $CDCl_3$).**

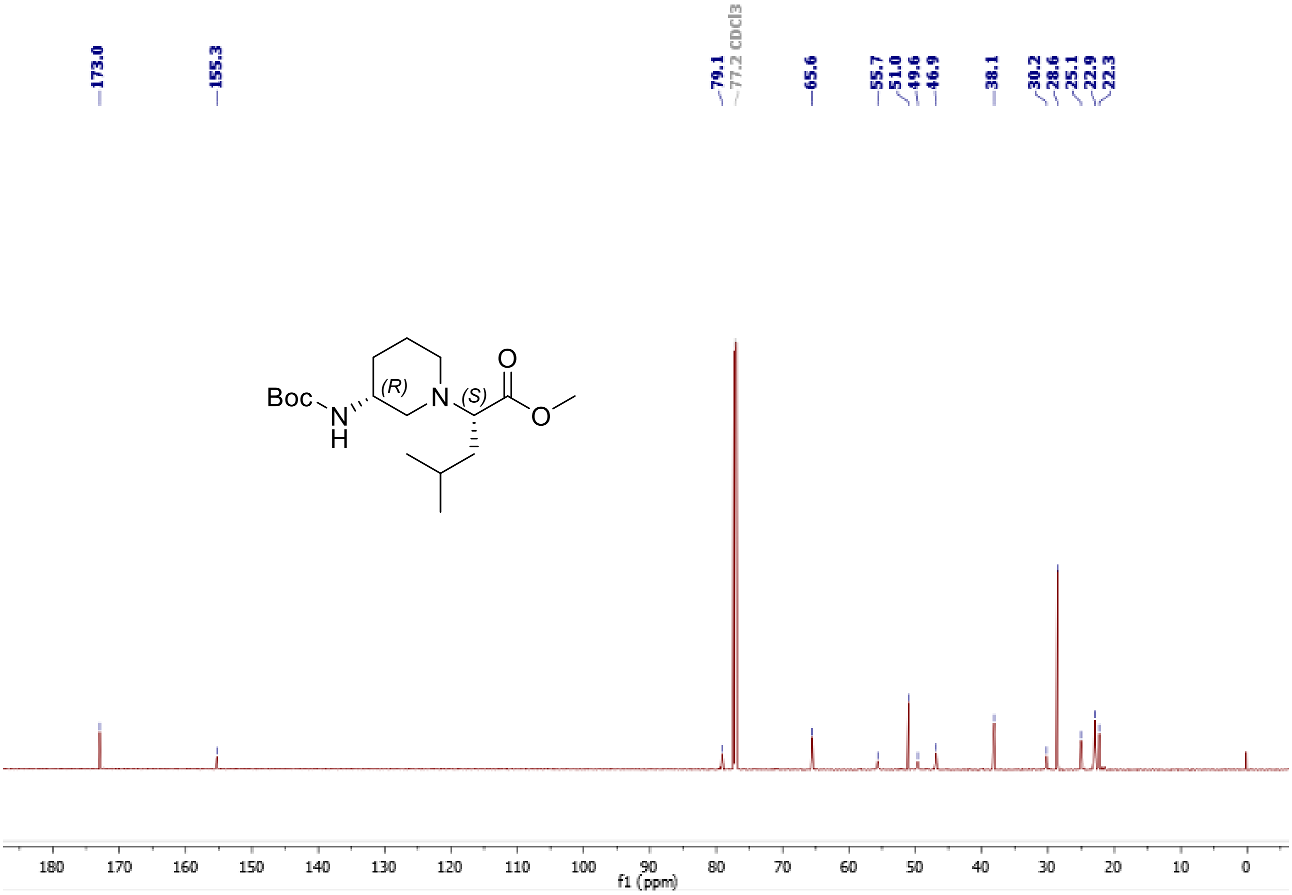

**Figure S85. Methyl (2*S*)-2-{(3*R*)-3-[(*tert*-butoxycarbonyl)amino]piperidin-1-yl}-4-methylpentanoate ((2*S*,3*R*)-10c). $^{13}$C NMR spectrum (176 MHz, $CDCl_3$).**

# Mass Spectrum SmartFormula Report

**Analysis Info**

| | | | |
|---|---|---|---|
| Analysis Name | D:\Data\Organikai\2023_04_11\GMP_600_1-E,6_01_10263.d | Acquisition Date | 4/27/2023 3:48:47 PM |
| Method | organikai_esi_pos_2013_recover.m | Operator | Milda Pukalskiene |
| Sample Name | GMP_600 | Instrument / Ser# | maXis 4G 20218 |
| Comment | | | |

**Acquisition Parameter**

| | | | | | |
|---|---|---|---|---|---|
| Source Type | ESI | Ion Polarity | Positive | Set Nebulizer | 1.5 Bar |
| Focus | Not active | Set Capillary | 4500 V | Set Dry Heater | 180 °C |
| Scan Begin | 40 m/z | Set End Plate Offset | -500 V | Set Dry Gas | 8.0 l/min |
| Scan End | 1800 m/z | Set Collision Cell RF | 350.0 Vpp | Set Divert Valve | Waste |

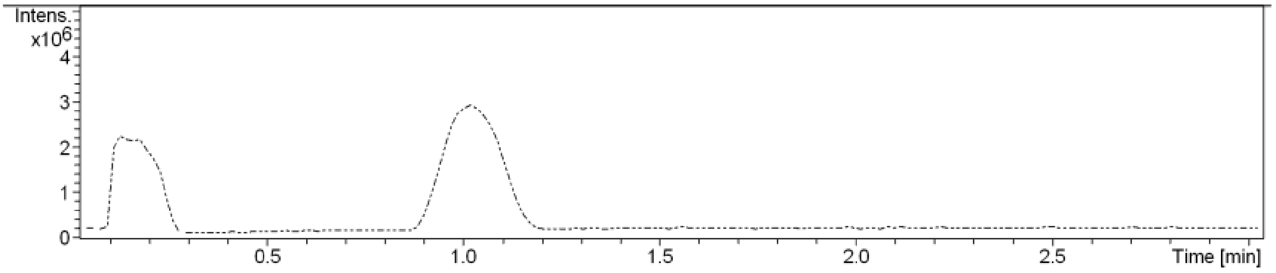


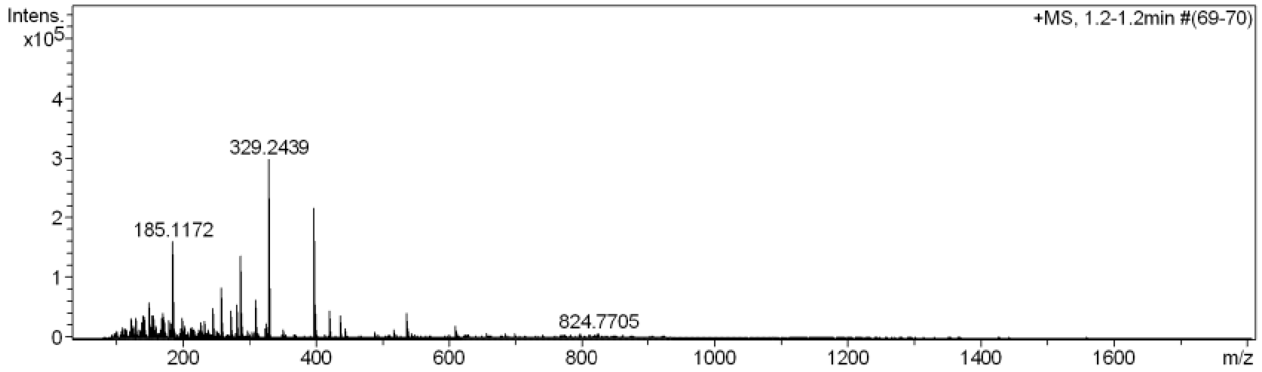


| Meas. m/z | # | Formula | Score | m/z | err [ppm] | Mean err [ppm] | mSigma | rdb | e⁻ Conf | N-Rule |
|---|---|---|---|---|---|---|---|---|---|---|
| 329.2439 | 1 | C 17 H 33 N 2 O 4 | 100.00 | 329.2435 | -1.2 | -1.0 | 15.2 | 2.5 | even | ok |

**Figure S86. Methyl (2*S*)-2-{(3*R*)-3-[(*tert*-butoxycarbonyl)amino]piperidin-1-yl}-4-methylpentanoate ((2*S*,3*R*)-10c). HRMS (ESI-TOF).**

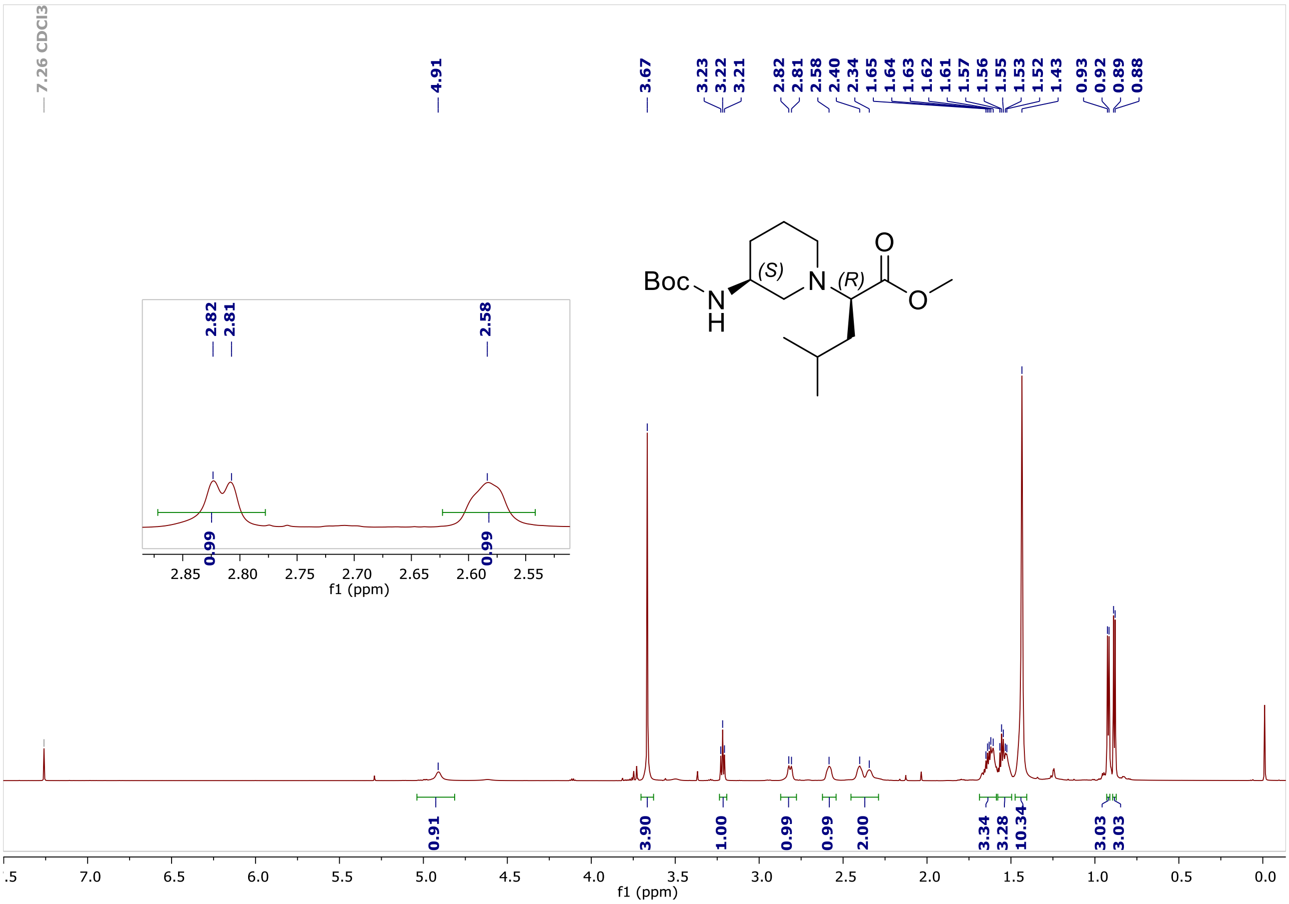

**Figure S87. Methyl (2*R*)-2-{(3*S*)-3-[(*tert*-butoxycarbonyl)amino]piperidin-1-yl}-4-methylpentanoate ((2*R*,3*S*)-10c). $^{1}$H NMR spectrum (700 MHz, $CDCl_3$).**

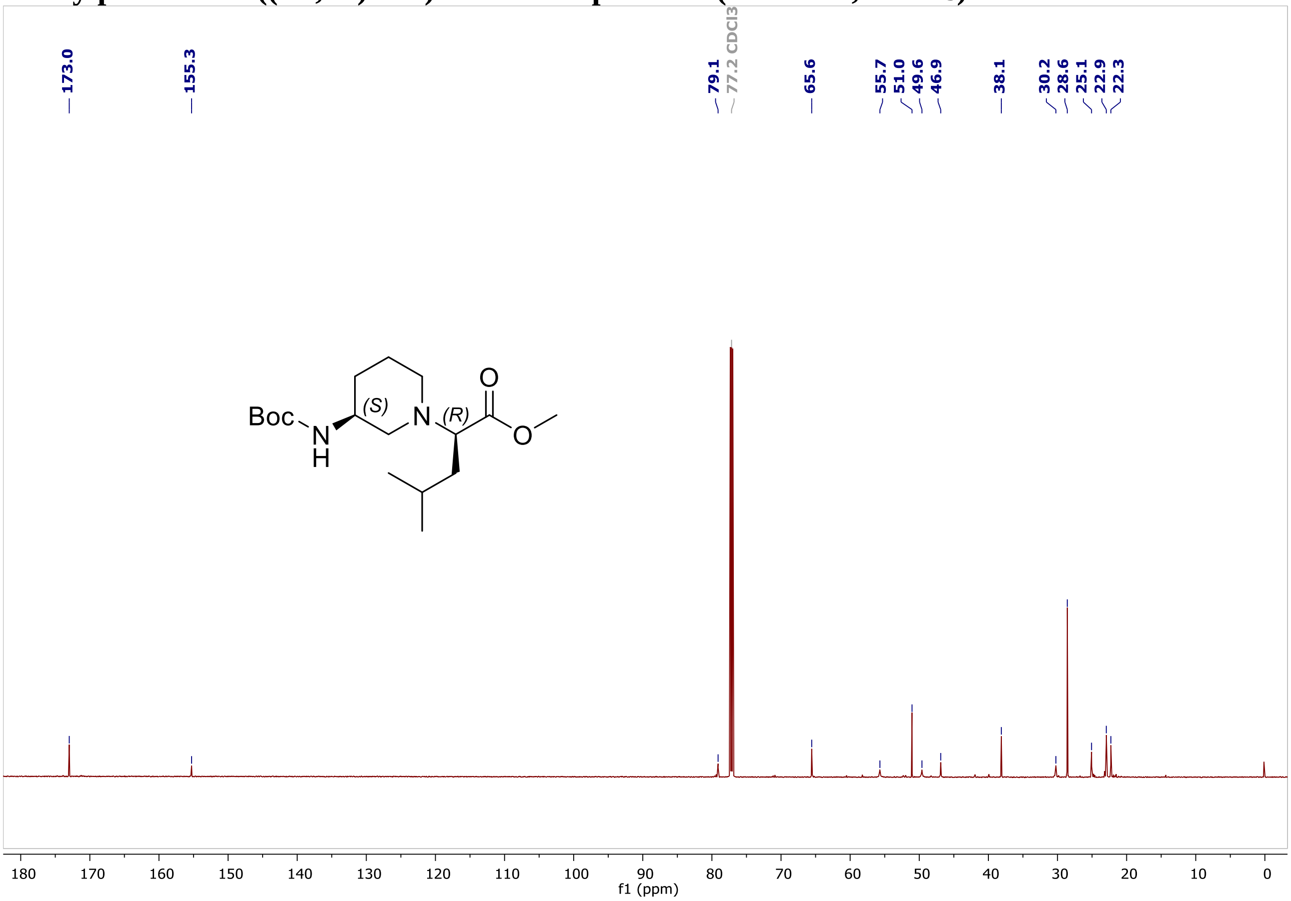

**Figure S88. Methyl (2*R*)-2-{(3*S*)-3-[(*tert*-butoxycarbonyl)amino]piperidin-1-yl}-4-methylpentanoate ((2*R*,3*S*)-10c). $^{13}$C NMR spectrum (176 MHz, $CDCl_3$).**

# Mass Spectrum SmartFormula Report

| Analysis Info | | | |
|---|---|---|---|
| Analysis Name | D:\Data\Organikai\2023_04_11\GMP_593_1-E,5_01_10262.d | Acquisition Date | 4/27/2023 3:44:18 PM |
| Method | organikai_esi_pos_2013_recover.m | Operator | Milda Pukalskiene |
| Sample Name | GMP_593 | Instrument / Ser# | maXis 4G 20218 |
| Comment | | | |

**Acquisition Parameter**

| | | | | | |
|---|---|---|---|---|---|
| Source Type | ESI | Ion Polarity | Positive | Set Nebulizer | 1.5 Bar |
| Focus | Not active | Set Capillary | 4500 V | Set Dry Heater | 180 °C |
| Scan Begin | 40 m/z | Set End Plate Offset | -500 V | Set Dry Gas | 8.0 l/min |
| Scan End | 1800 m/z | Set Collision Cell RF | 350.0 Vpp | Set Divert Valve | Waste |

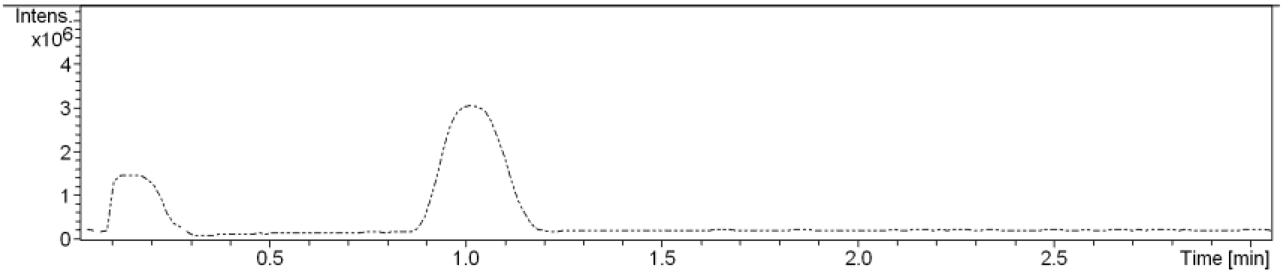


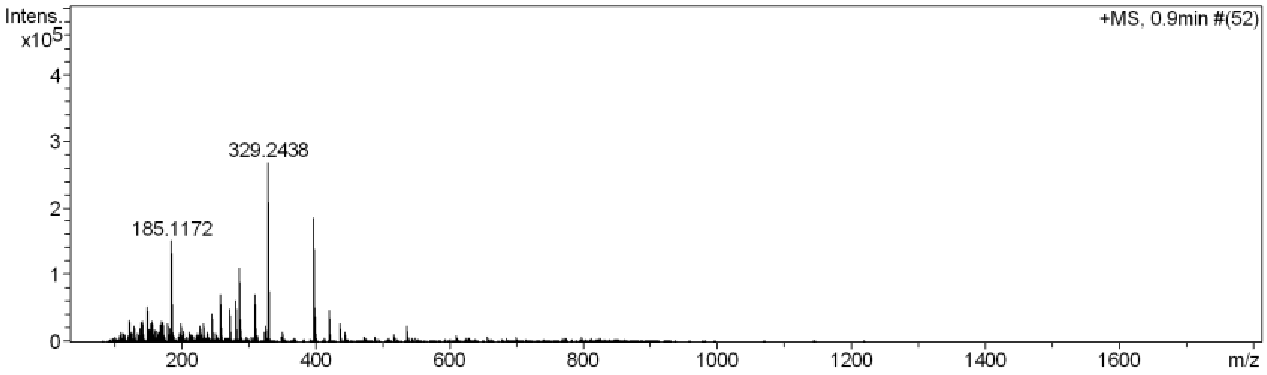


| Meas. m/z | # | Formula | Score | m/z | err [ppm] | Mean err [ppm] | mSigma | rdb | e⁻ Conf | N-Rule |
|---|---|---|---|---|---|---|---|---|---|---|
| 329.2438 | 1 | C 17 H 33 N 2 O 4 | 100.00 | 329.2435 | -1.1 | -0.9 | 15.2 | 2.5 | even | ok |

**Figure S89. Methyl (2*R*)-2-{(3*S*)-3-[(*tert*-butoxycarbonyl)amino]piperidin-1-yl}-4-methylpentanoate ((2*R*,3*S*)-10c). HRMS (ESI-TOF).**

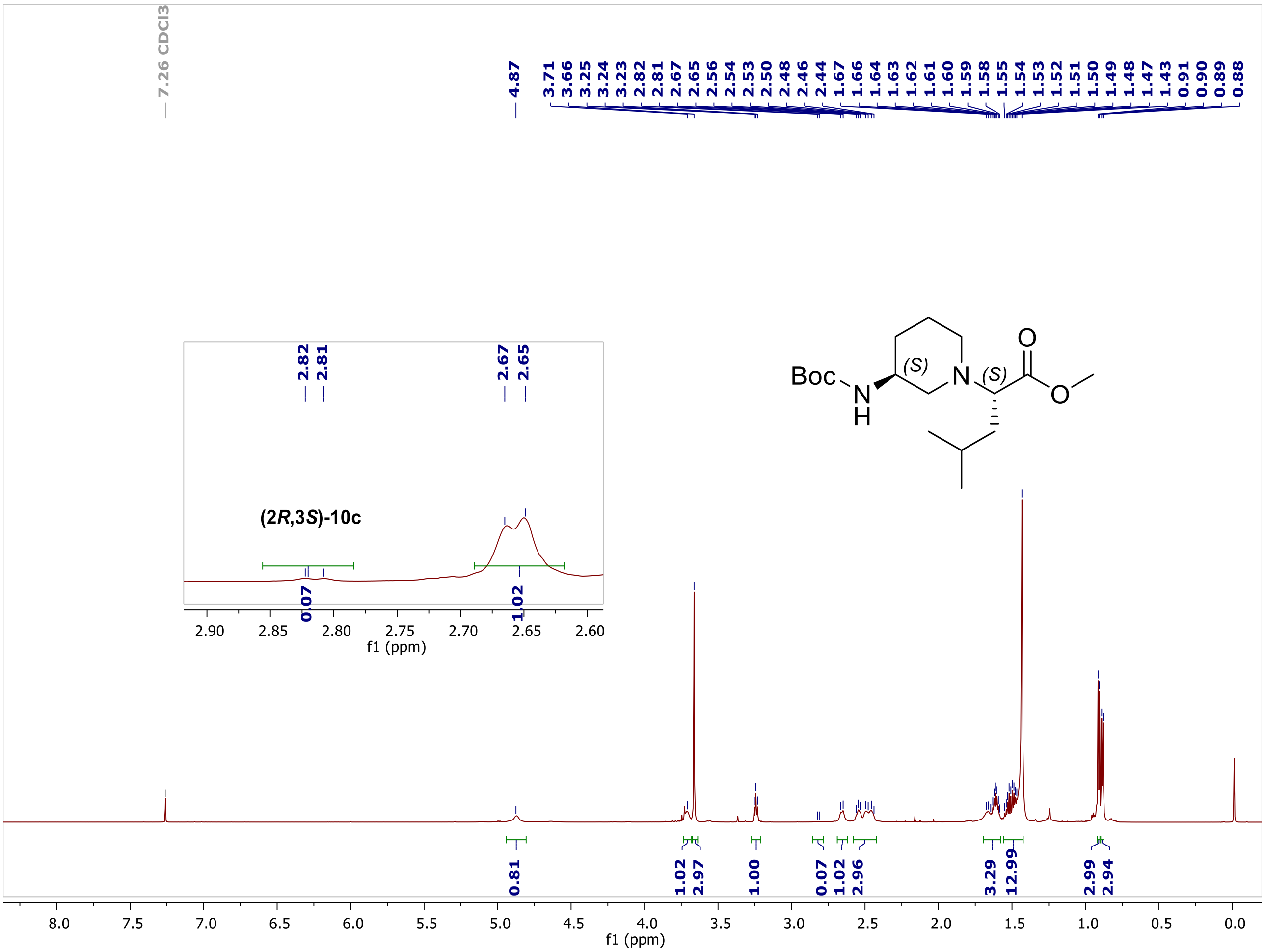


**Figure S90. Methyl (2*S*)-2-{(3*S*)-3-[(*tert*-butoxycarbonyl)amino]piperidin-1-yl}-4-methylpentanoate ((2*S*,3*S*)-10c). $^{1}$H NMR spectrum (700 MHz, $CDCl_3$).**

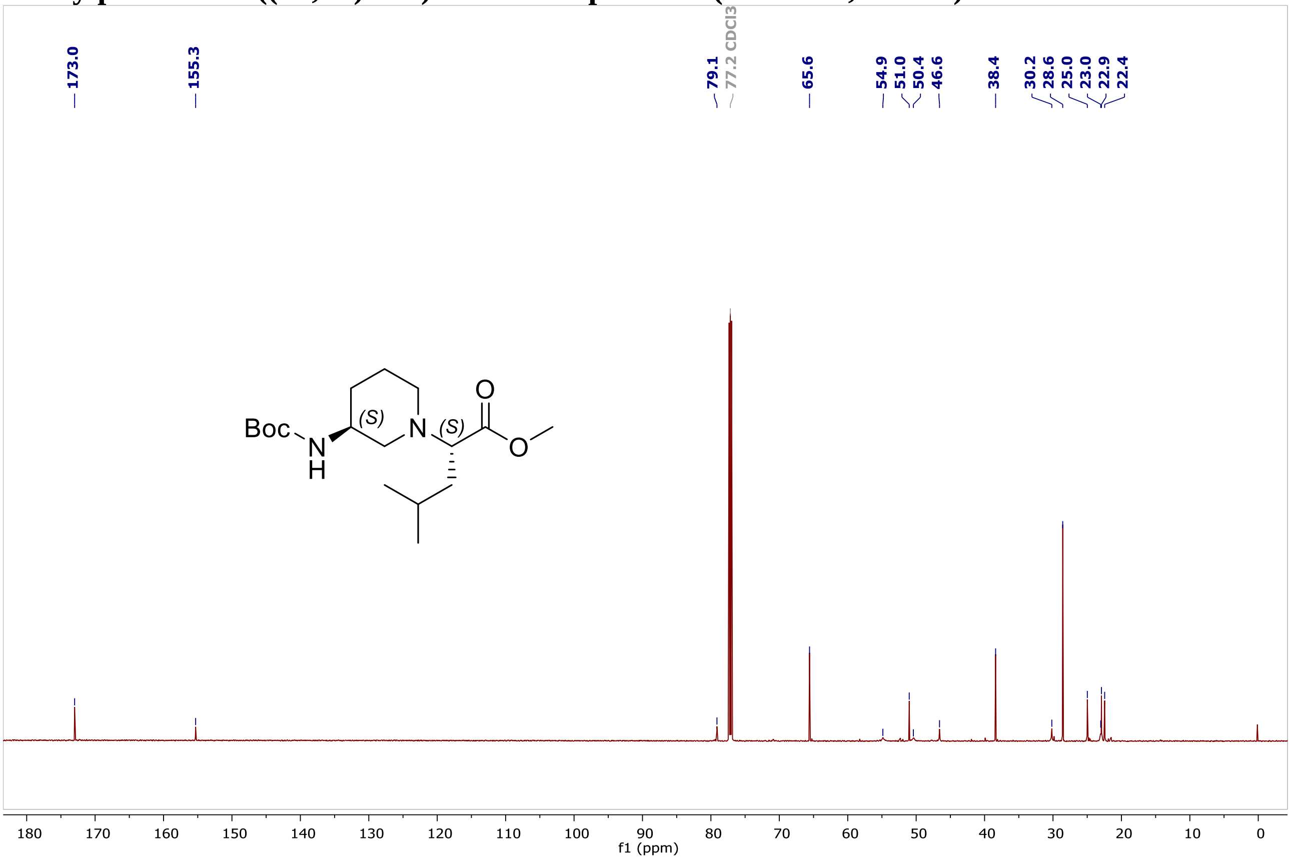


**Figure S91. Methyl (2*S*)-2-{(3*S*)-3-[(*tert*-butoxycarbonyl)amino]piperidin-1-yl}-4-methylpentanoate ((2*S*,3*S*)-10c). $^{13}$C NMR spectrum (176 MHz, $CDCl_3$).**

# Mass Spectrum SmartFormula Report

**Analysis Info**

| | | | |
|---|---|---|---|
| Analysis Name | D:\Data\Organikai\2023_04_11\GMP_598_1-E,4_01_10261.d | Acquisition Date | 4/27/2023 3:39:50 PM |
| Method | organikai_esi_pos_2013_recover.m | Operator | Milda Pukalskiene |
| Sample Name | GMP_598 | Instrument / Ser# | maXis 4G 20218 |
| Comment | | | |

**Acquisition Parameter**

| | | | | | |
|---|---|---|---|---|---|
| Source Type | ESI | Ion Polarity | Positive | Set Nebulizer | 1.5 Bar |
| Focus | Not active | Set Capillary | 4500 V | Set Dry Heater | 180 °C |
| Scan Begin | 40 m/z | Set End Plate Offset | -500 V | Set Dry Gas | 8.0 l/min |
| Scan End | 1800 m/z | Set Collision Cell RF | 350.0 Vpp | Set Divert Valve | Waste |

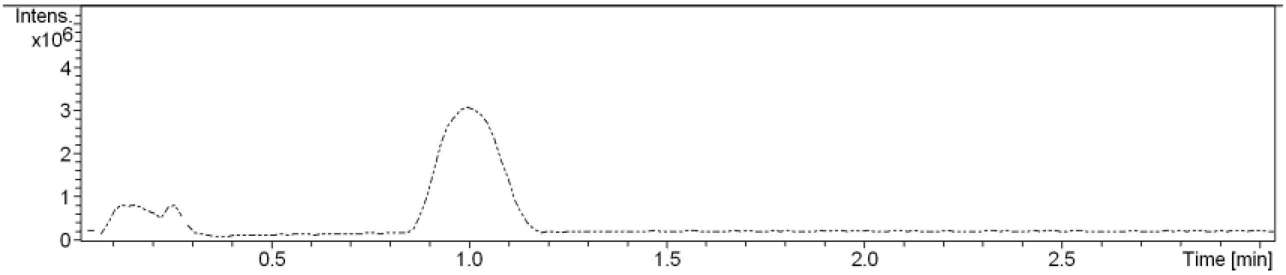


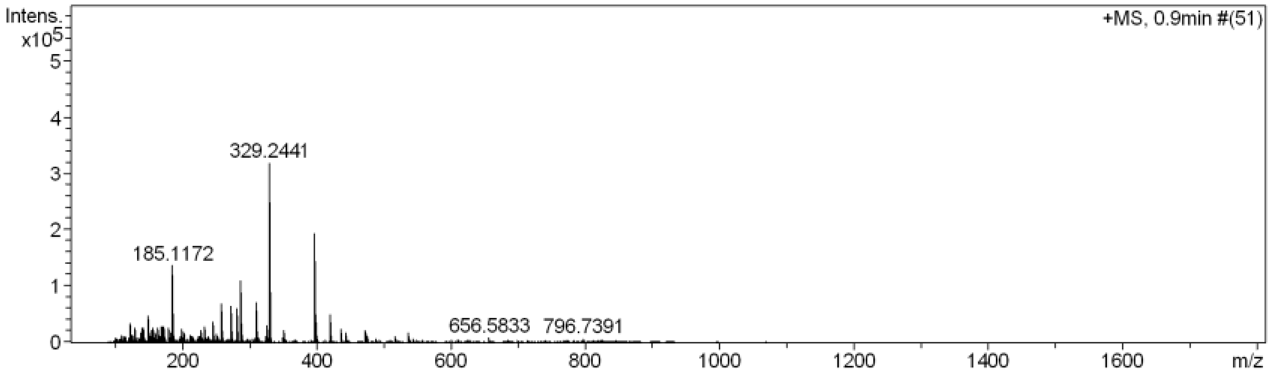


| Meas. m/z | # | Formula | Score | m/z | err [ppm] | Mean err [ppm] | mSigma | rdb | e⁻ Conf | N-Rule |
|---|---|---|---|---|---|---|---|---|---|---|
| 329.2441 | 1 | C 17 H 33 N 2 O 4 | 100.00 | 329.2435 | -1.8 | -1.6 | 16.5 | 2.5 | even | ok |

**Figure S92. Methyl (2*S*)-2-{(3*S*)-3-[(*tert*-butoxycarbonyl)amino]piperidin-1-yl}-4-methylpentanoate ((2*S*,3*S*)-10c). HRMS (ESI-TOF).**

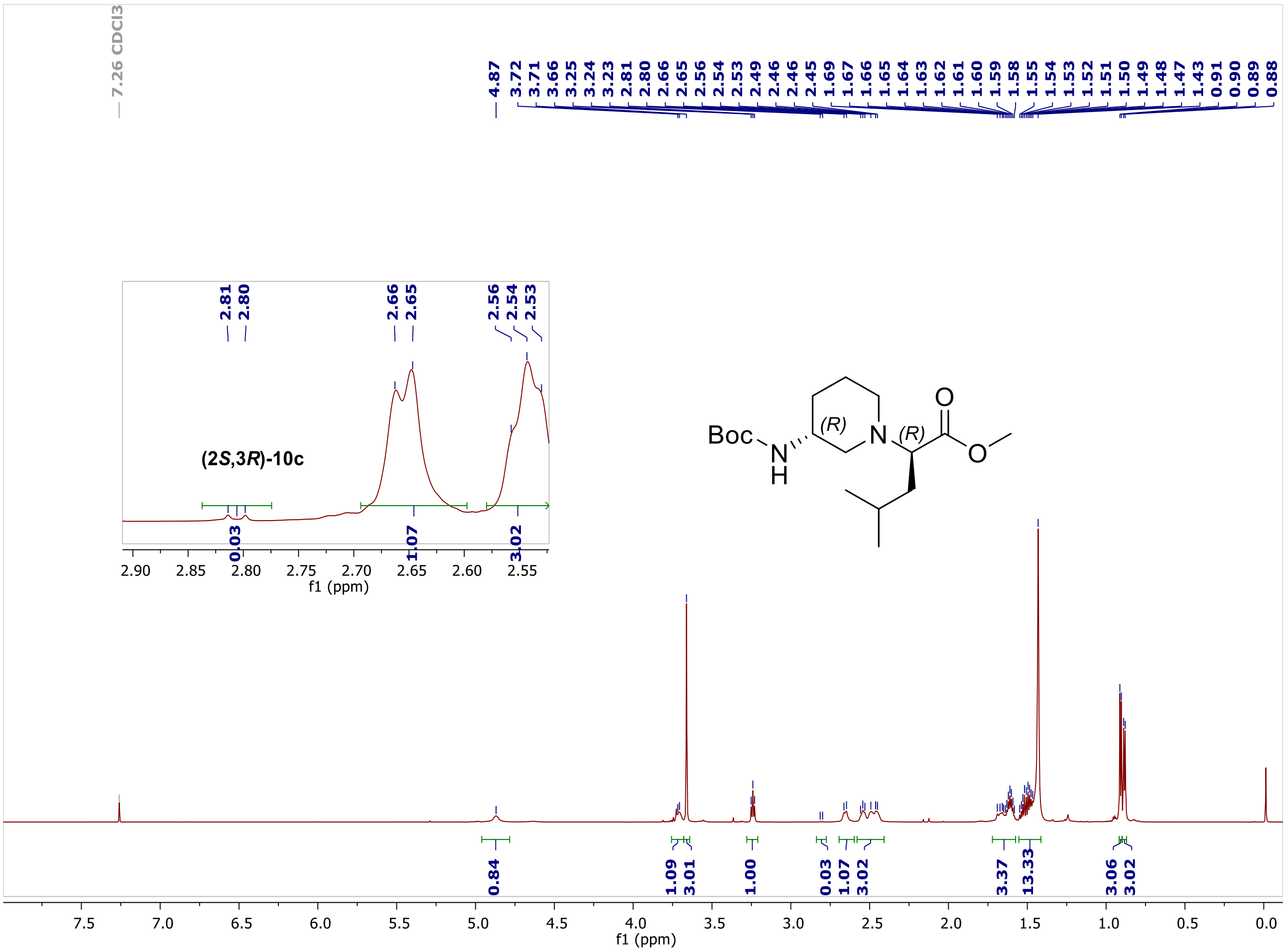

**Figure S93. Methyl (2*R*)-2-{(3*R*)-3-[(*tert*-butoxycarbonyl)amino]piperidin-1-yl}-4-methylpentanoate ((2*R*,3*R*)-10c). $^{1}$H NMR spectrum (700 MHz, $CDCl_3$).**

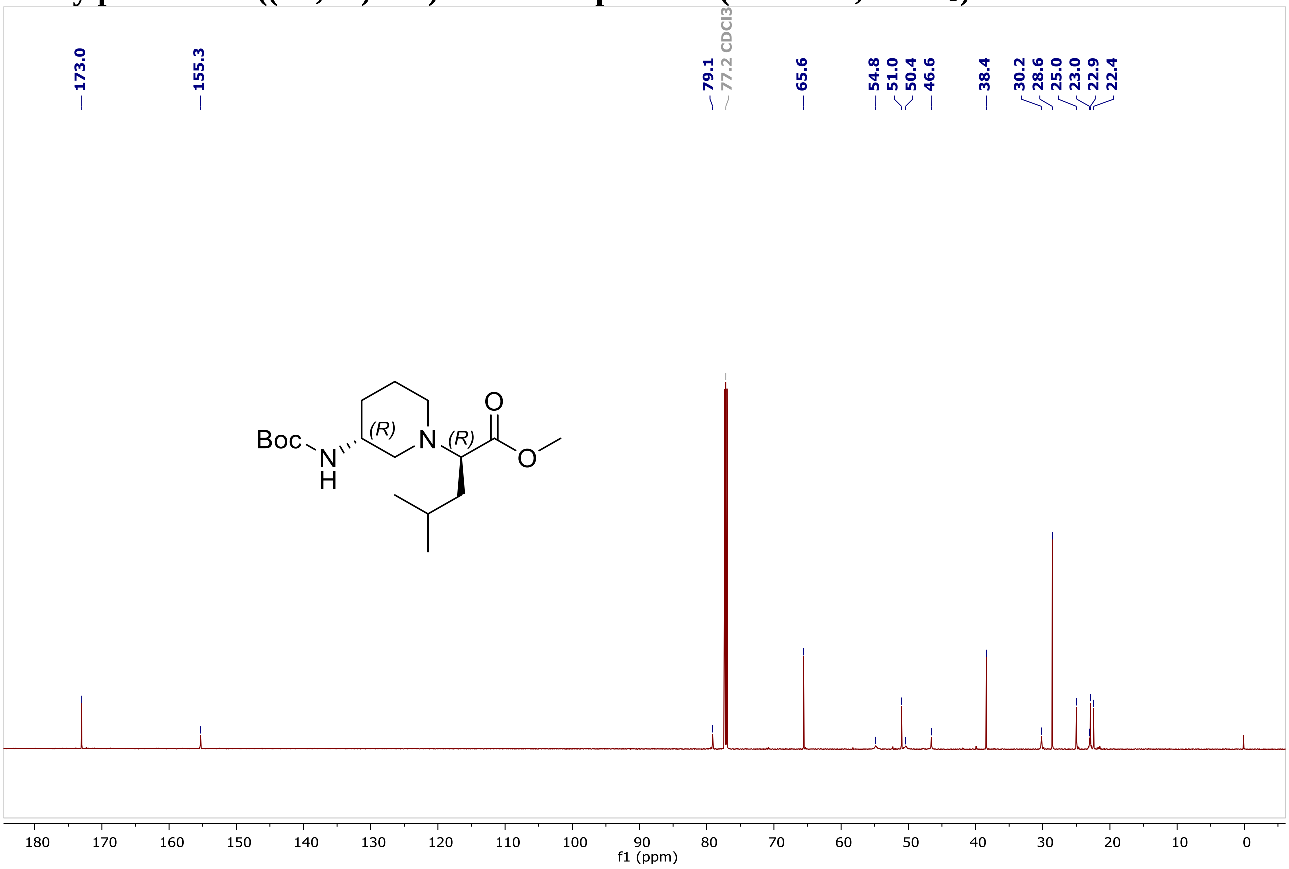

**Figure S94. Methyl (2*R*)-2-{(3*R*)-3-[(*tert*-butoxycarbonyl)amino]piperidin-1-yl}-4-methylpentanoate ((2*R*,3*R*)-10c). $^{13}$C NMR spectrum (176 MHz, $CDCl_3$).**

# Mass Spectrum SmartFormula Report

**Analysis Info**

| | | | |
|---|---|---|---|
| Analysis Name | D:\Data\Organikai\2023_04_11\GMP_595_1-E,7_01_10264.d | Acquisition Date | 4/27/2023 3:53:15 PM |
| Method | organikai_esi_pos_2013_recover.m | Operator | Milda Pukalskiene |
| Sample Name | GMP_595 | Instrument / Ser# | maXis 4G 20218 |
| Comment | | | |

**Acquisition Parameter**

| | | | | | |
|---|---|---|---|---|---|
| Source Type | ESI | Ion Polarity | Positive | Set Nebulizer | 1.5 Bar |
| Focus | Not active | Set Capillary | 4500 V | Set Dry Heater | 180 °C |
| Scan Begin | 40 m/z | Set End Plate Offset | -500 V | Set Dry Gas | 8.0 l/min |
| Scan End | 1800 m/z | Set Collision Cell RF | 350.0 Vpp | Set Divert Valve | Waste |

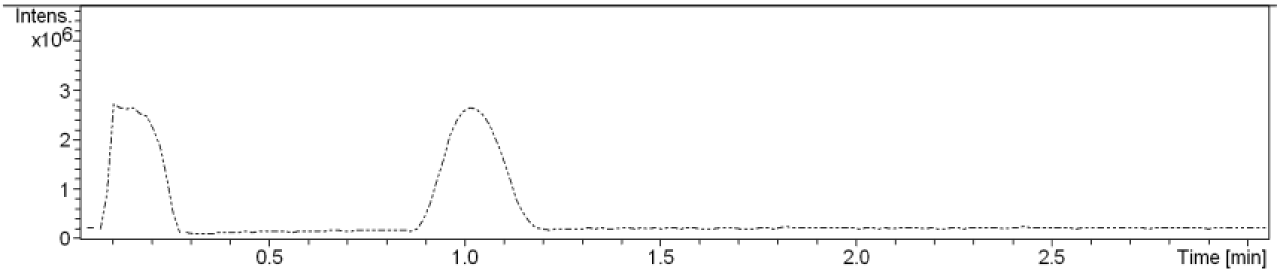


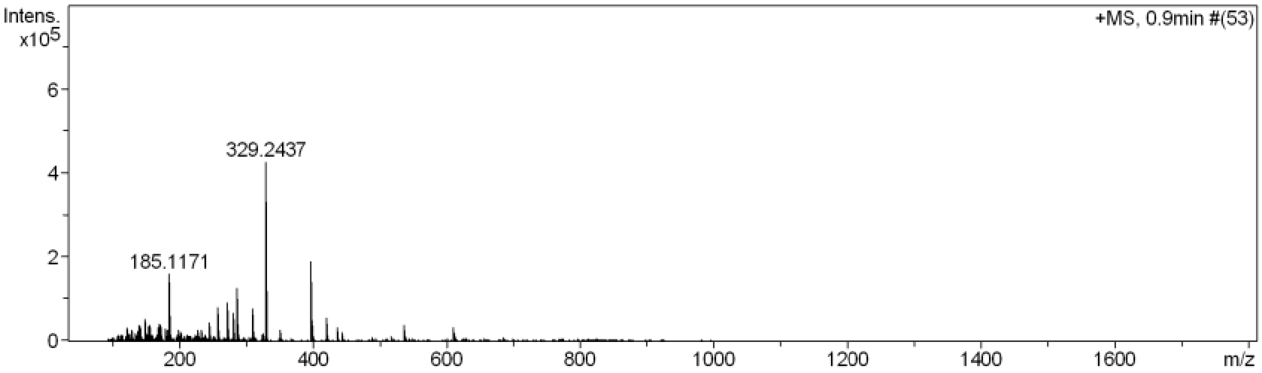


| Meas. m/z | # | Formula | Score | m/z | err [ppm] | Mean err [ppm] | mSigma | rdb | e⁻ Conf | N-Rule |
|---|---|---|---|---|---|---|---|---|---|---|
| 329.2437 | 1 | C 17 H 33 N 2 O 4 | 100.00 | 329.2435 | -0.8 | -0.6 | 15.6 | 2.5 | even | ok |

**Figure S95. Methyl (2*R*)-2-{(3*R*)-3-[(*tert*-butoxycarbonyl)amino]piperidin-1-yl}-4-methylpentanoate ((2*R*,3*R*)-10c). HRMS (ESI-TOF).**

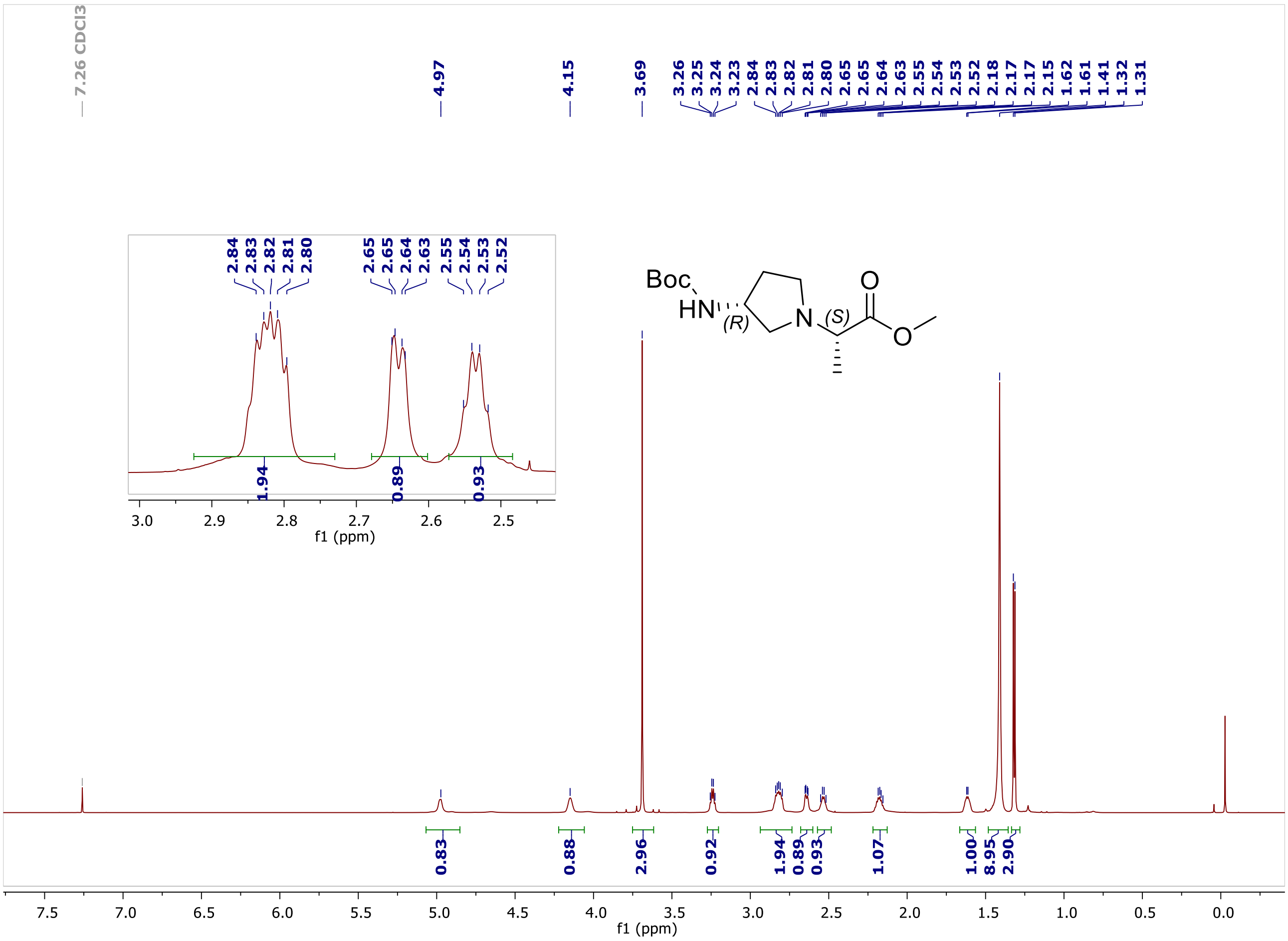

**Figure S96. Methyl (2*S*)-2-{(3*R*)-3-[(*tert*-butoxycarbonyl)amino]pyrrolidin-1-yl}propanoate ((2*S*,3*R*)-11a). $^{1}$H NMR spectrum (700 MHz, $CDCl_3$).**

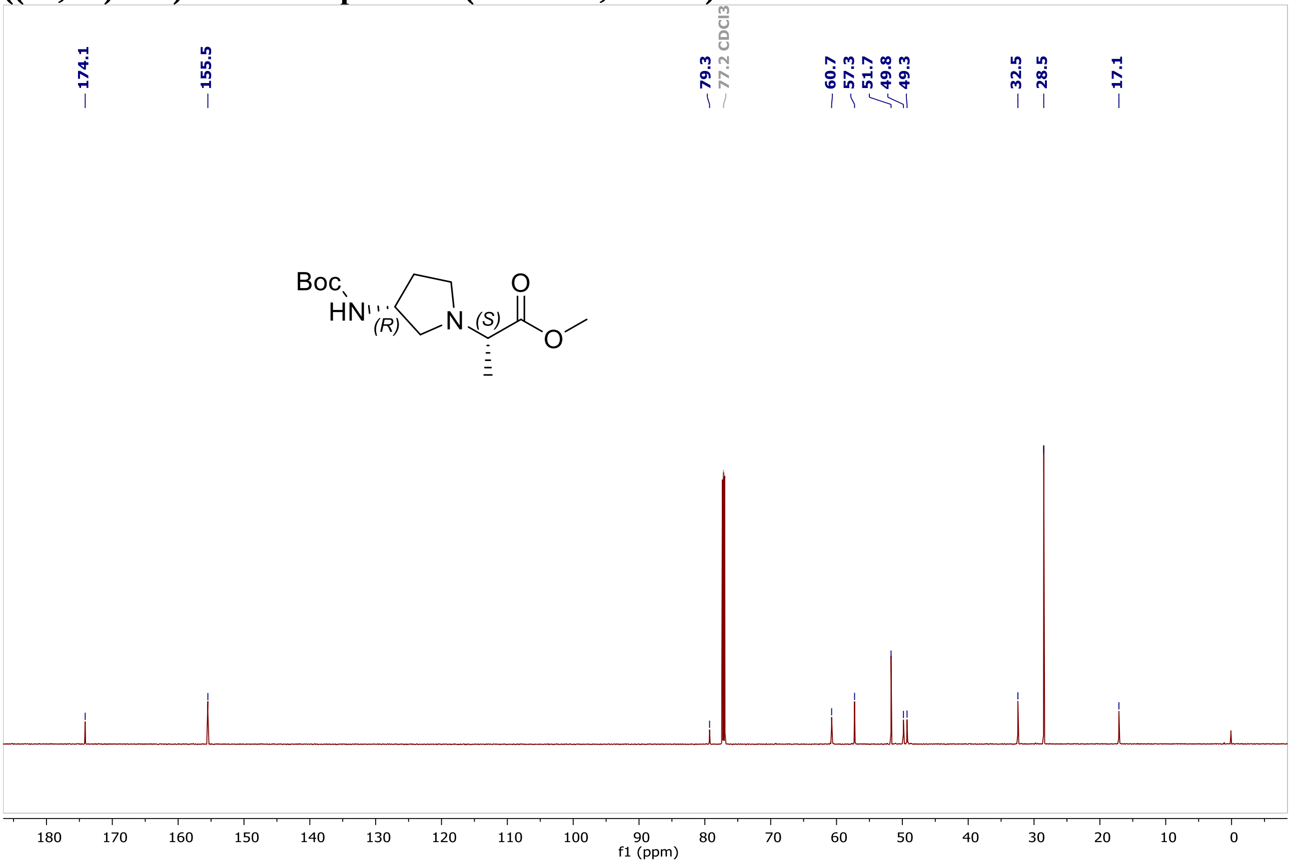

**Figure S97. Methyl (2*S*)-2-{(3*R*)-3-[(*tert*-butoxycarbonyl)amino]pyrrolidin-1-yl}propanoate ((2*S*,3*R*)-11a). $^{13}$C NMR spectrum (176 MHz, $CDCl_3$).**

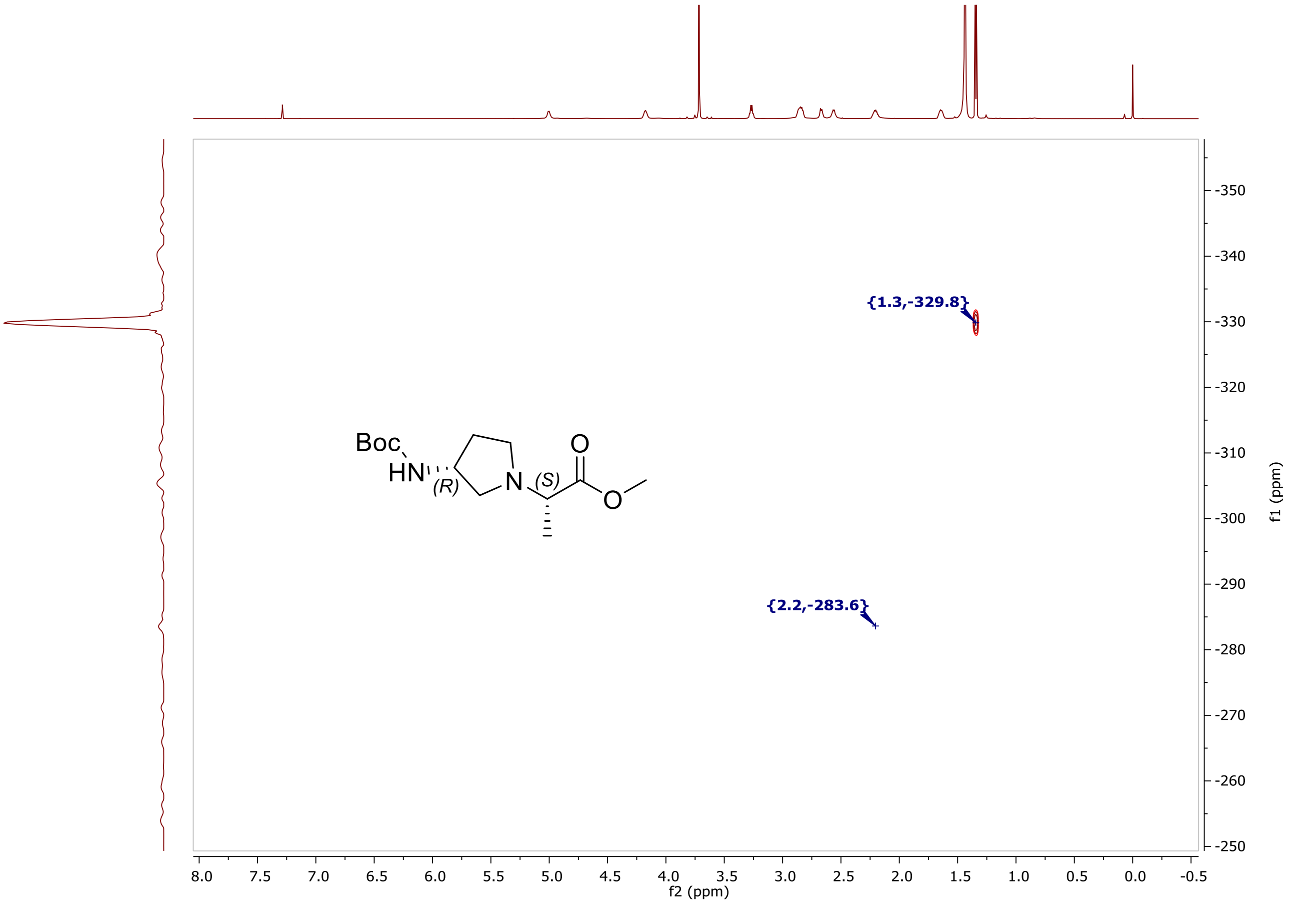


**Figure S98. Methyl (2*S*)-2-{(3*R*)-3-[(*tert*-butoxycarbonyl)amino]pyrrolidin-1-yl}propanoate ((2*S*,3*R*)-11a). $^{1}H$-$^{15}N$ HMBC spectrum (71 MHz, $CDCl_3$).**

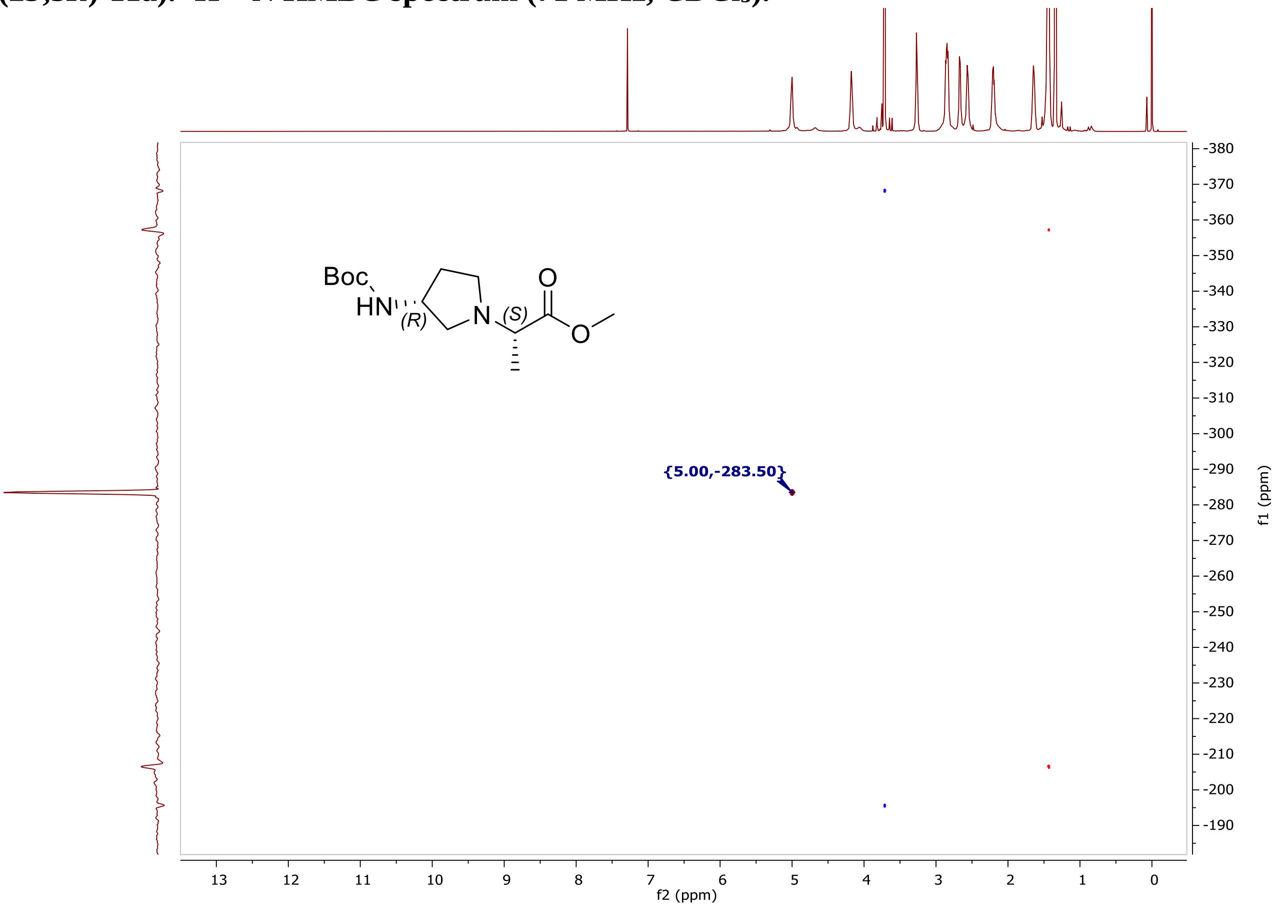


**Figure S99. Methyl (2*S*)-2-{(3*R*)-3-[(*tert*-butoxycarbonyl)amino]pyrrolidin-1-yl}propanoate ((2*S*,3*R*)-11a). $^{1}H$-$^{15}N$ HSQC spectrum (71 MHz, $CDCl_3$).**

# Mass Spectrum SmartFormula Report

**Analysis Info**

| | | | |
|---|---|---|---|
| Analysis Name | D:\Data\Organikai\2023_04_11\GMP_368_1-D,4_01_10234.d | Acquisition Date | 4/21/2023 2:18:52 PM |
| Method | organikai_esi_pos_2013_recover.m | Operator | Milda Pukalskiene |
| Sample Name | GMP_368 | Instrument / Ser# | maXis 4G 20218 |
| Comment | | | |

**Acquisition Parameter**

| | | | | | |
|---|---|---|---|---|---|
| Source Type | ESI | Ion Polarity | Positive | Set Nebulizer | 1.5 Bar |
| Focus | Not active | Set Capillary | 4500 V | Set Dry Heater | 180 °C |
| Scan Begin | 40 m/z | Set End Plate Offset | -500 V | Set Dry Gas | 8.0 l/min |
| Scan End | 1800 m/z | Set Collision Cell RF | 350.0 Vpp | Set Divert Valve | Waste |

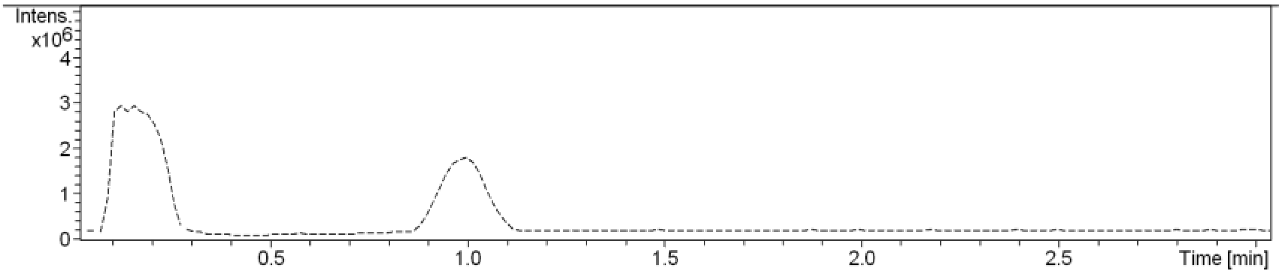


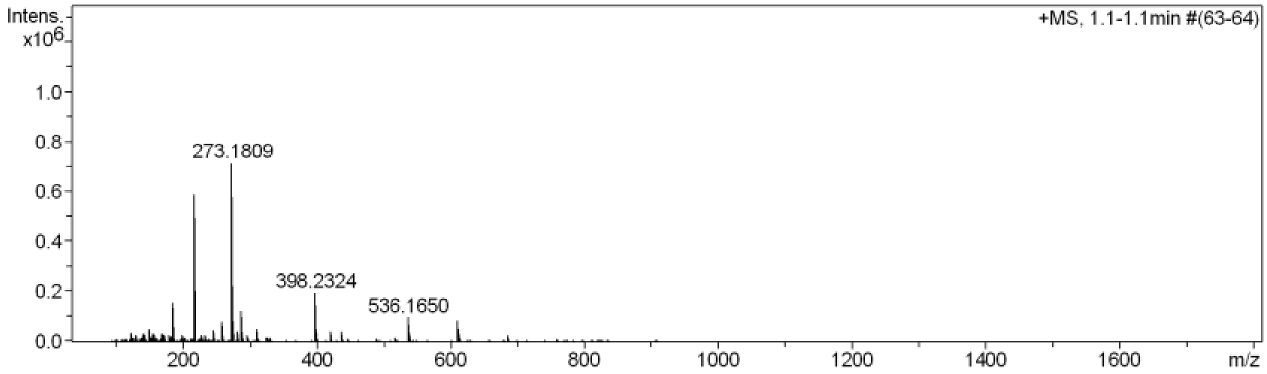


| Meas. m/z | # | Formula | Score | m/z | err [ppm] | Mean err [ppm] | mSigma | rdb | e⁻ Conf | N-Rule |
|---|---|---|---|---|---|---|---|---|---|---|
| 273.1809 | 1 | C 13 H 25 N 2 O 4 | 100.00 | 273.1809 | -0.1 | 0.2 | 3.1 | 2.5 | even | ok |

**Figure S100. Methyl (2*S*)-2-{(3*R*)-3-[(*tert*-butoxycarbonyl)amino]pyrrolidin-1-yl}propanoate ((2*S*,3*R*)-11a). HRMS (ESI-TOF).**

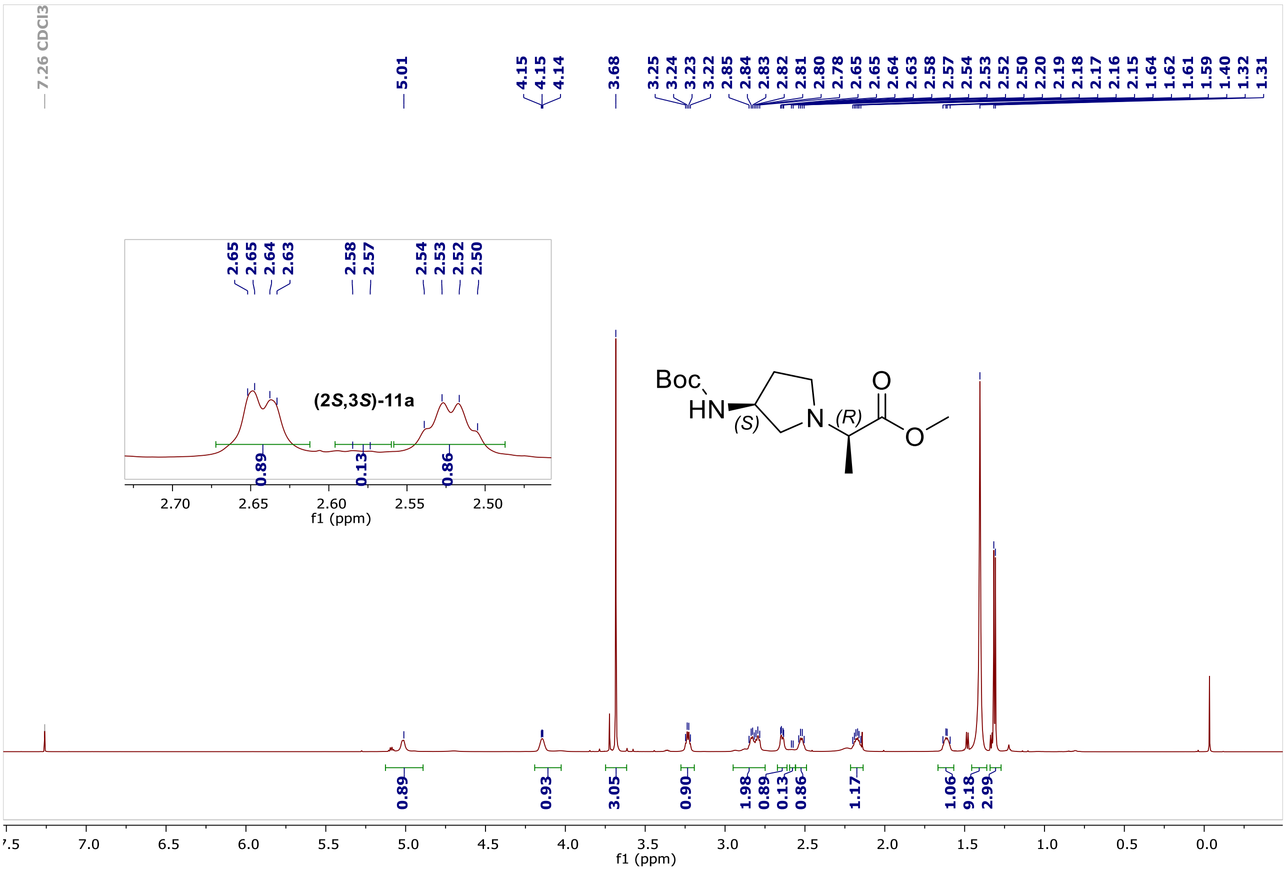

**Figure S101. Methyl (2*R*)-2-{(3*S*)-3-[(*tert*-butoxycarbonyl)amino]pyrrolidin-1-yl}propanoate ((2*R*,3*S*)-11a). $^{1}$H NMR spectrum (700 MHz, $CDCl_3$).**

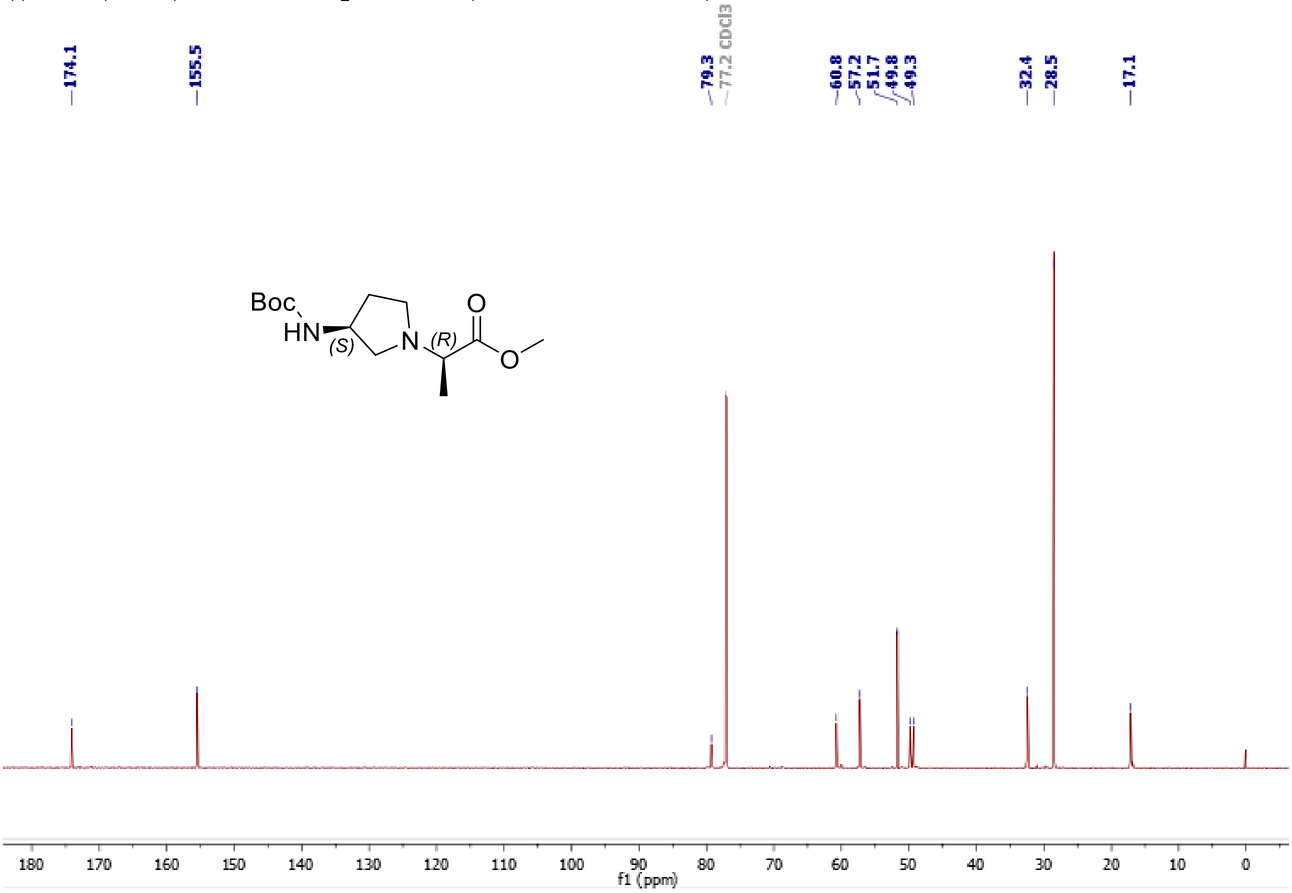

**Figure S102. Methyl (2*R*)-2-{(3*S*)-3-[(*tert*-butoxycarbonyl)amino]pyrrolidin-1-yl}propanoate ((2*R*,3*S*)-11a). $^{13}$C NMR spectrum (176 MHz, $CDCl_3$).**

# Mass Spectrum SmartFormula Report

**Analysis Info**

| | | | |
|---|---|---|---|
| Analysis Name | D:\Data\Organikai\2023_04_11\GMP_482_1-D,7_01_10237.d | Acquisition Date | 4/21/2023 2:32:19 PM |
| Method | organikai_esi_pos_2013_recover.m | Operator | Milda Pukalskiene |
| Sample Name | GMP_482 | Instrument / Ser# | maXis 4G 20218 |
| Comment | | | |

**Acquisition Parameter**

| | | | | | |
|---|---|---|---|---|---|
| Source Type | ESI | Ion Polarity | Positive | Set Nebulizer | 1.5 Bar |
| Focus | Not active | Set Capillary | 4500 V | Set Dry Heater | 180 °C |
| Scan Begin | 40 m/z | Set End Plate Offset | -500 V | Set Dry Gas | 8.0 l/min |
| Scan End | 1800 m/z | Set Collision Cell RF | 350.0 Vpp | Set Divert Valve | Waste |

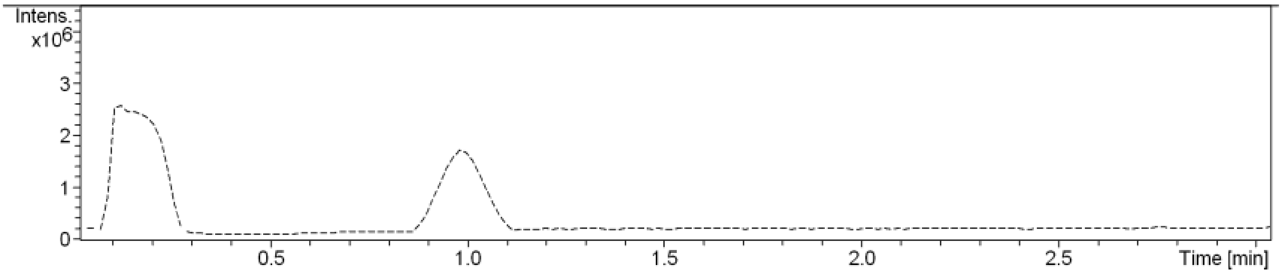


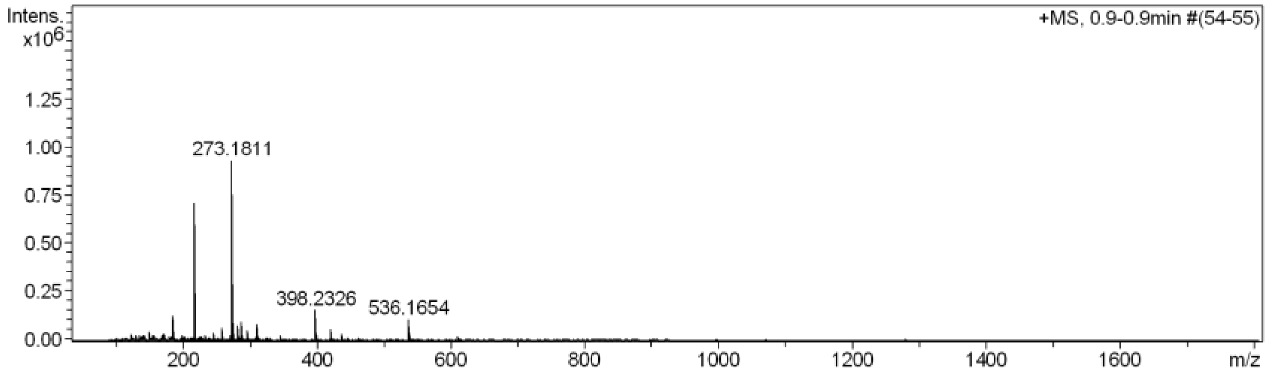


| Meas. m/z | # | Formula | Score | m/z | err [ppm] | Mean err [ppm] | mSigma | rdb | e⁻ Conf | N-Rule |
|---|---|---|---|---|---|---|---|---|---|---|
| 273.1811 | 1 | C 13 H 25 N 2 O 4 | 100.00 | 273.1809 | -0.8 | -0.5 | 18.3 | 2.5 | even | ok |

**Figure S103. Methyl (2*R*)-2-{(3*S*)-3-[(*tert*-butoxycarbonyl)amino]pyrrolidin-1-yl}propanoate ((2*R*,3*S*)-11a). HRMS (ESI-TOF).**

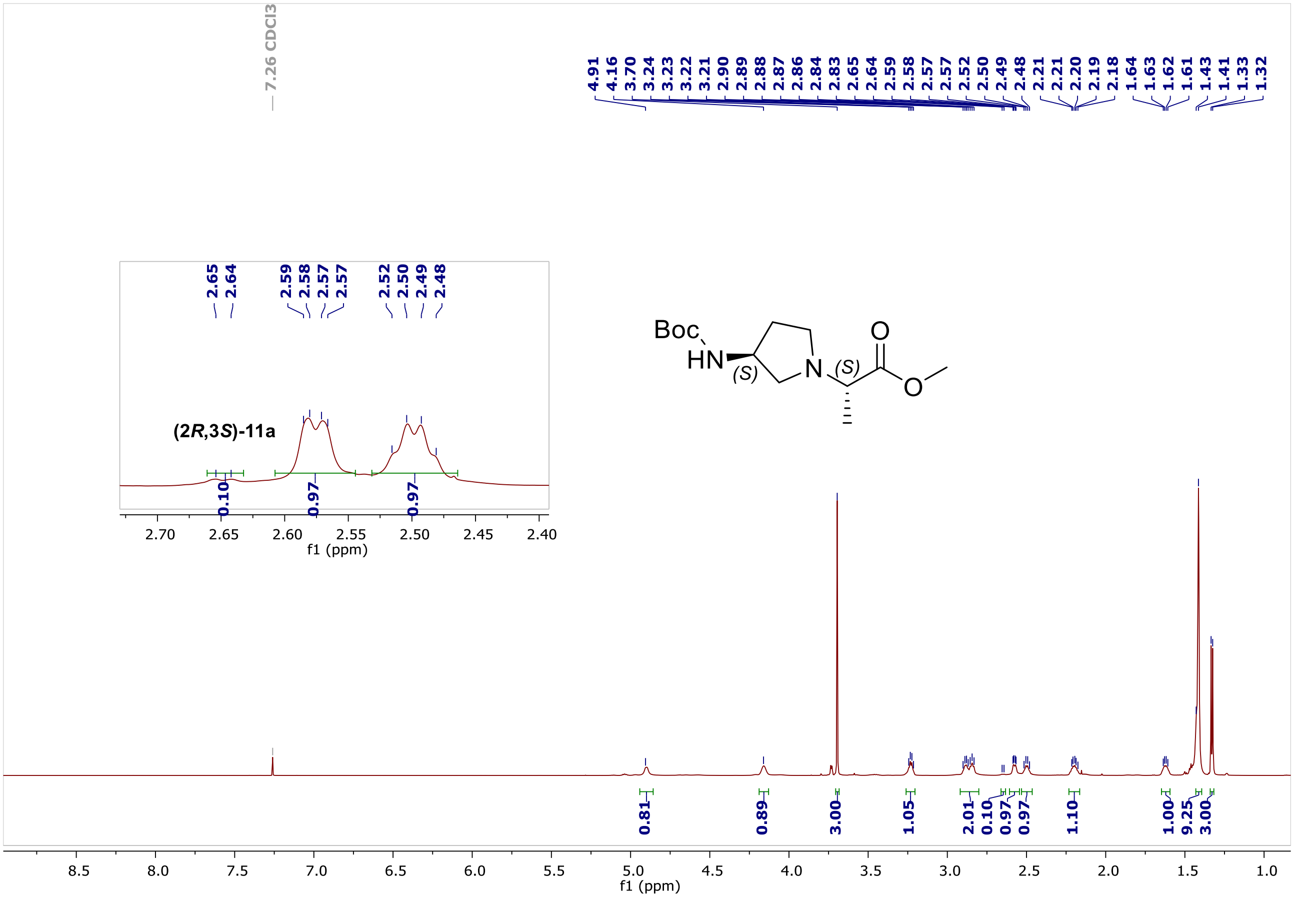


**Figure S104. Methyl (2*S*)-2-{(3*S*)-3-[(*tert*-butoxycarbonyl)amino]pyrrolidin-1-yl}propanoate ((2*S*,3*S*)-11a). $^{1}$H NMR spectrum (700 MHz, $CDCl_3$).**

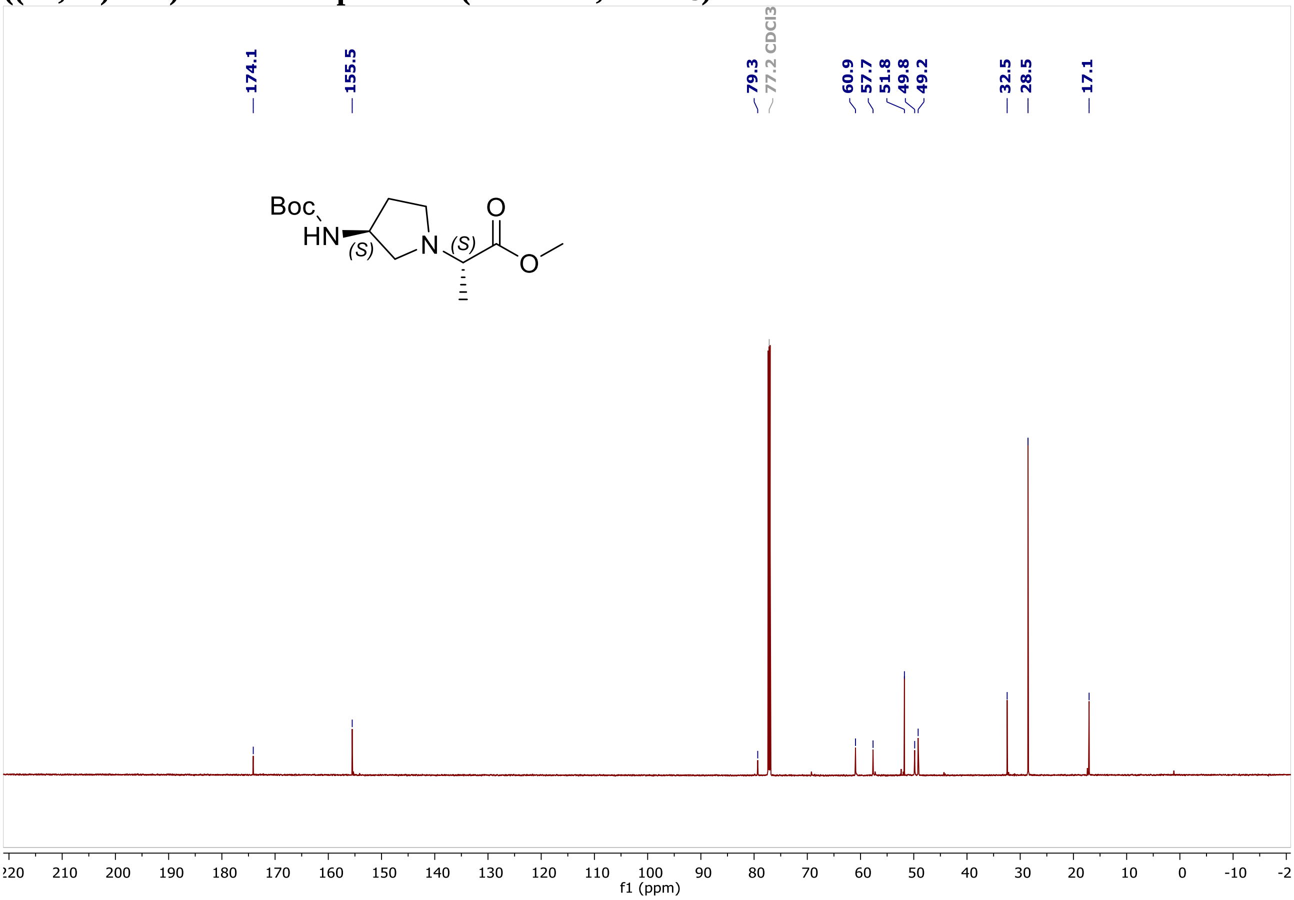


**Figure S105. Methyl (2*S*)-2-{(3*S*)-3-[(*tert*-butoxycarbonyl)amino]pyrrolidin-1-yl}propanoate ((2*S*,3*S*)-11a). $^{13}$C NMR spectrum (176 MHz, $CDCl_3$).**

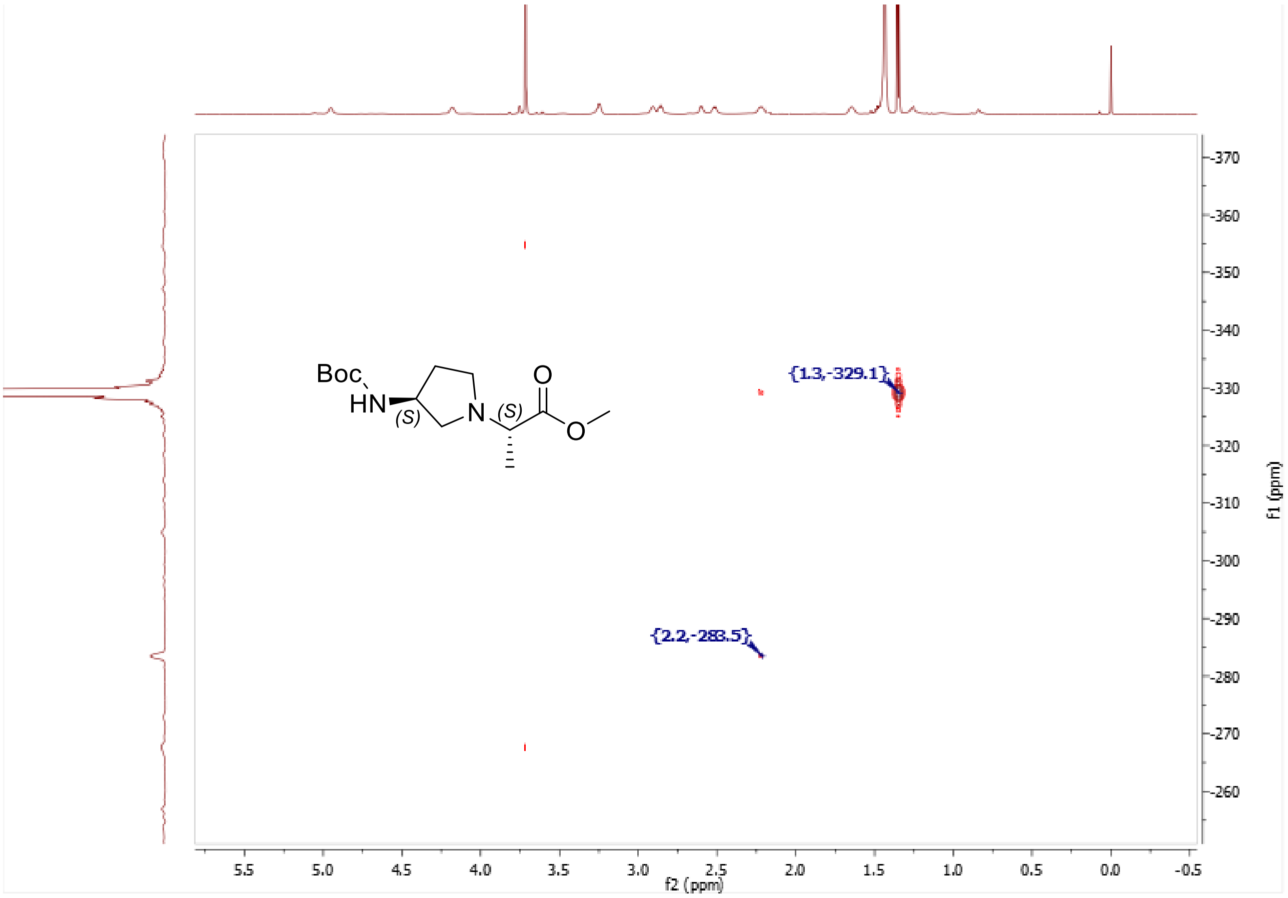


**Figure S106. Methyl (2*S*)-2-{(3*S*)-3-[(*tert*-butoxycarbonyl)amino]pyrrolidin-1-yl}propanoate ((2*S*,3*S*)-11a). $^{1}H$-$^{15}N$ HMBC spectrum (71 MHz, $CDCl_3$).**

# Mass Spectrum SmartFormula Report

**Analysis Info**

| | | | |
|---|---|---|---|
| Analysis Name | D:\Data\Organikai\2023_04_11\GMP_373_1-D,6_01_10236.d | Acquisition Date | 4/21/2023 2:27:50 PM |
| Method | organikai_esi_pos_2013_recover.m | Operator | Milda Pukalskiene |
| Sample Name | GMP_373 | Instrument / Ser# | maXis 4G 20218 |
| Comment | | | |

**Acquisition Parameter**

| | | | | | |
|---|---|---|---|---|---|
| Source Type | ESI | Ion Polarity | Positive | Set Nebulizer | 1.5 Bar |
| Focus | Not active | Set Capillary | 4500 V | Set Dry Heater | 180 °C |
| Scan Begin | 40 m/z | Set End Plate Offset | -500 V | Set Dry Gas | 8.0 l/min |
| Scan End | 1800 m/z | Set Collision Cell RF | 350.0 Vpp | Set Divert Valve | Waste |

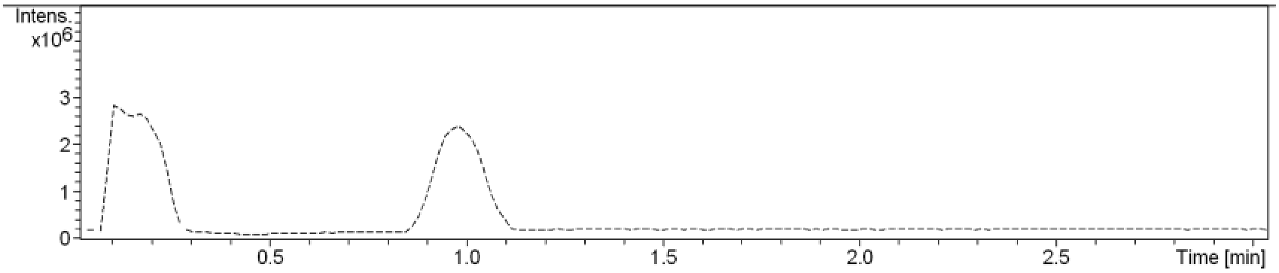


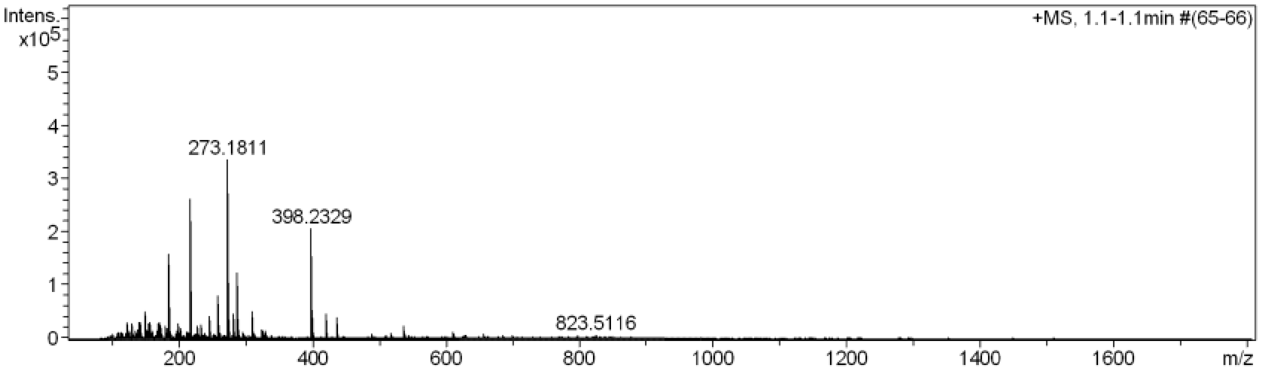


| Meas. m/z | # | Formula | Score | m/z | err [ppm] | Mean err [ppm] | mSigma | rdb | e⁻ Conf | N-Rule |
|---|---|---|---|---|---|---|---|---|---|---|
| 273.1811 | 1 | C 13 H 25 N 2 O 4 | 100.00 | 273.1809 | -0.7 | -0.5 | 8.1 | 2.5 | even | ok |

**Figure S107. Methyl (2*S*)-2-{(3*S*)-3-[(*tert*-butoxycarbonyl)amino]pyrrolidin-1-yl}propanoate ((2*S*,3*S*)-11a). HRMS (ESI-TOF).**

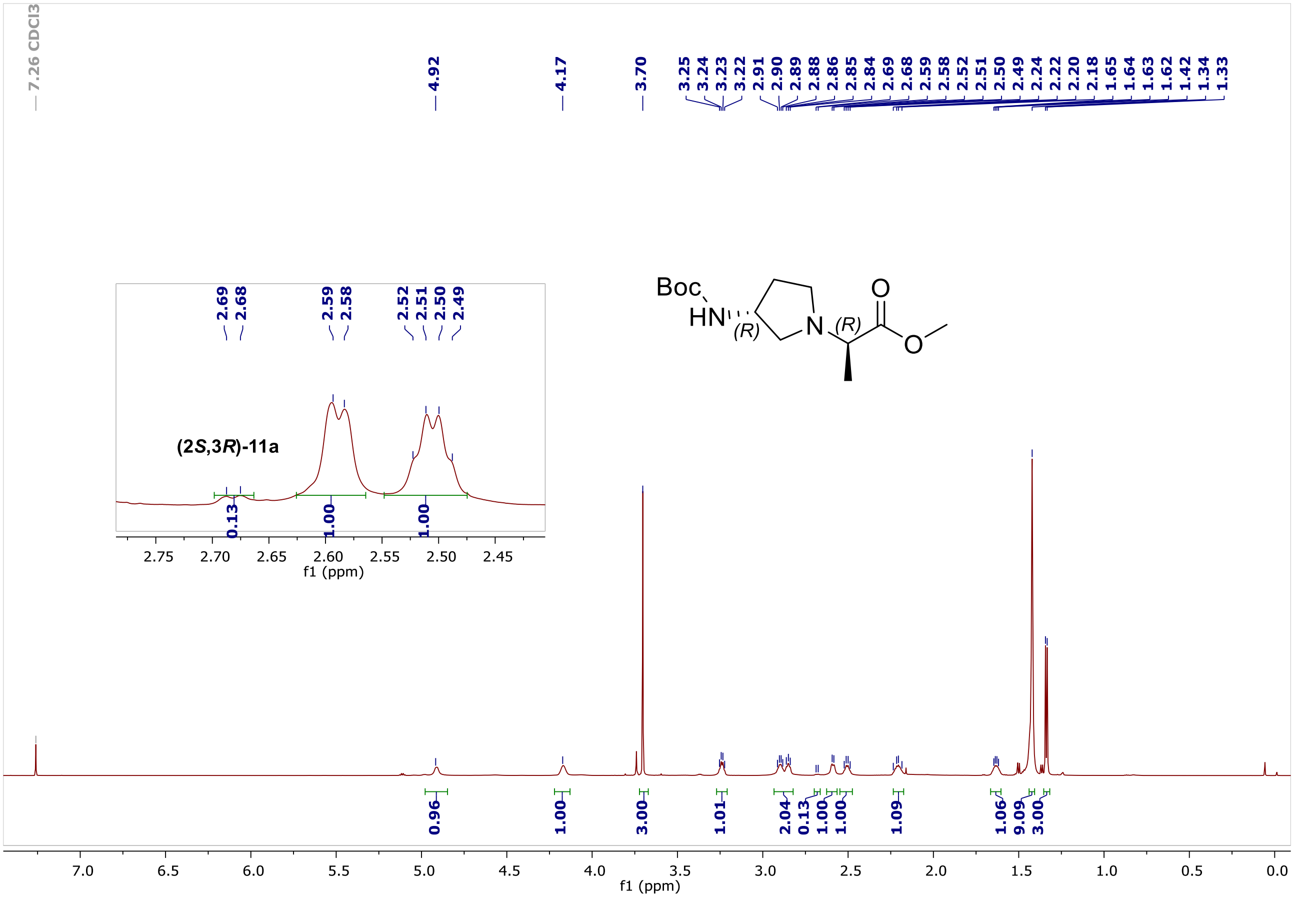


**Figure S108.** **Methyl (2*R*)-2-{(3*R*)-3-[(*tert*-butoxycarbonyl)amino]pyrrolidin-1-yl}propanoate ((2*R*,3*R*)-11a). $^{1}$H NMR spectrum (700 MHz, $CDCl_3$).**

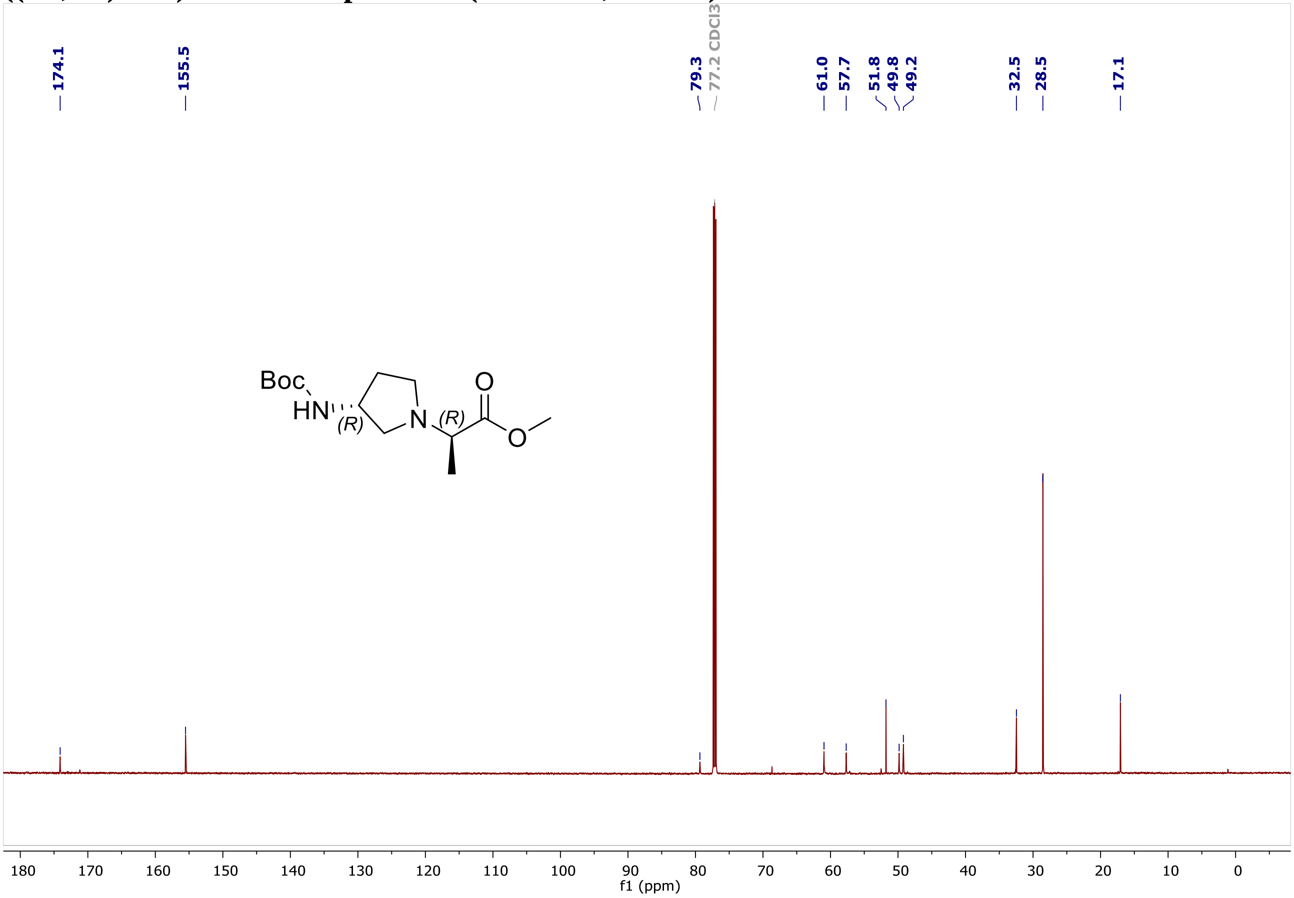


**Figure S109.** **Methyl (2*R*)-2-{(3*R*)-3-[(*tert*-butoxycarbonyl)amino]pyrrolidin-1-yl}propanoate ((2*R*,3*R*)-11a). $^{13}$C NMR spectrum (176 MHz, $CDCl_3$).**

# Mass Spectrum SmartFormula Report

**Analysis Info**

| | | | |
|---|---|---|---|
| Analysis Name | D:\Data\Organikai\2023_04_11\GMP_476_1-D,5_01_10235.d | Acquisition Date | 4/21/2023 2:23:19 PM |
| Method | organikai_esi_pos_2013_recover.m | Operator | Milda Pukalskiene |
| Sample Name | GMP_476 | Instrument / Ser# | maXis 4G 20218 |
| Comment | | | |

**Acquisition Parameter**

| | | | | | |
|---|---|---|---|---|---|
| Source Type | ESI | Ion Polarity | Positive | Set Nebulizer | 1.5 Bar |
| Focus | Not active | Set Capillary | 4500 V | Set Dry Heater | 180 °C |
| Scan Begin | 40 m/z | Set End Plate Offset | -500 V | Set Dry Gas | 8.0 l/min |
| Scan End | 1800 m/z | Set Collision Cell RF | 350.0 Vpp | Set Divert Valve | Waste |

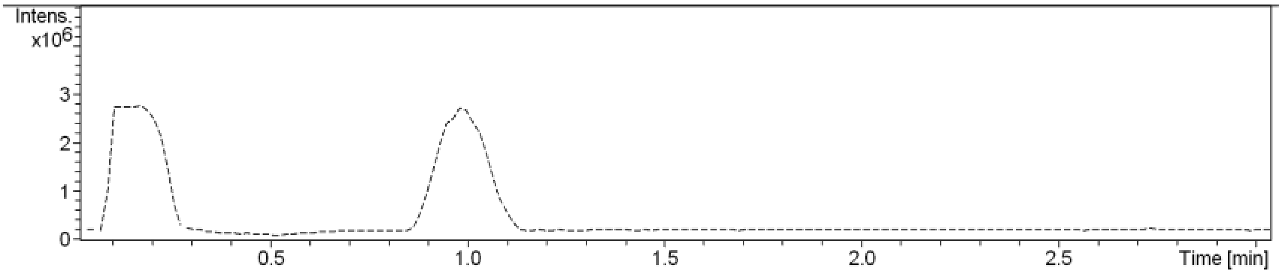


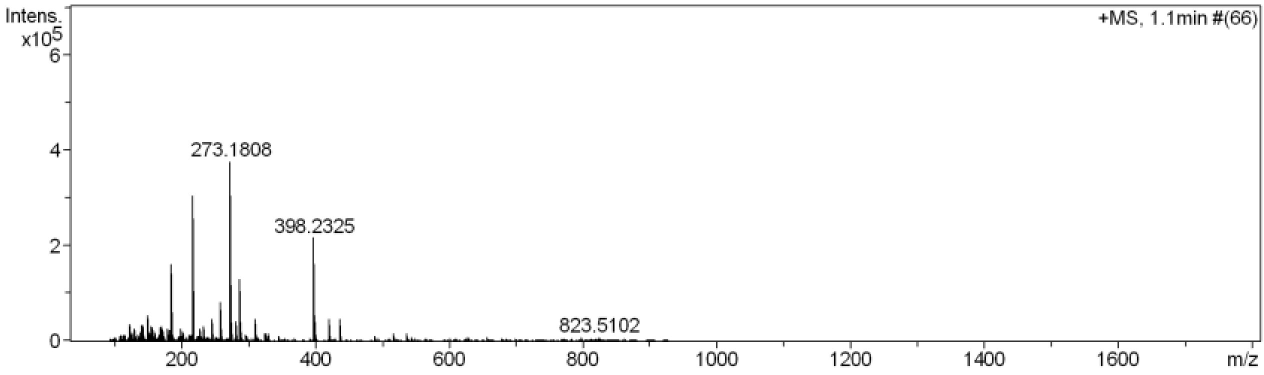


| Meas. m/z | # | Formula | Score | m/z | err [ppm] | Mean err [ppm] | mSigma | rdb | e⁻ Conf | N-Rule |
|---|---|---|---|---|---|---|---|---|---|---|
| 273.1808 | 1 | C 13 H 25 N 2 O 4 | 100.00 | 273.1809 | 0.2 | 0.4 | 14.7 | 2.5 | even | ok |

**Figure S110. Methyl (2*R*)-2-{(3*R*)-3-[(*tert*-butoxycarbonyl)amino]pyrrolidin-1-yl}propanoate ((2*R*,3*R*)-11a). HRMS (ESI-TOF).**

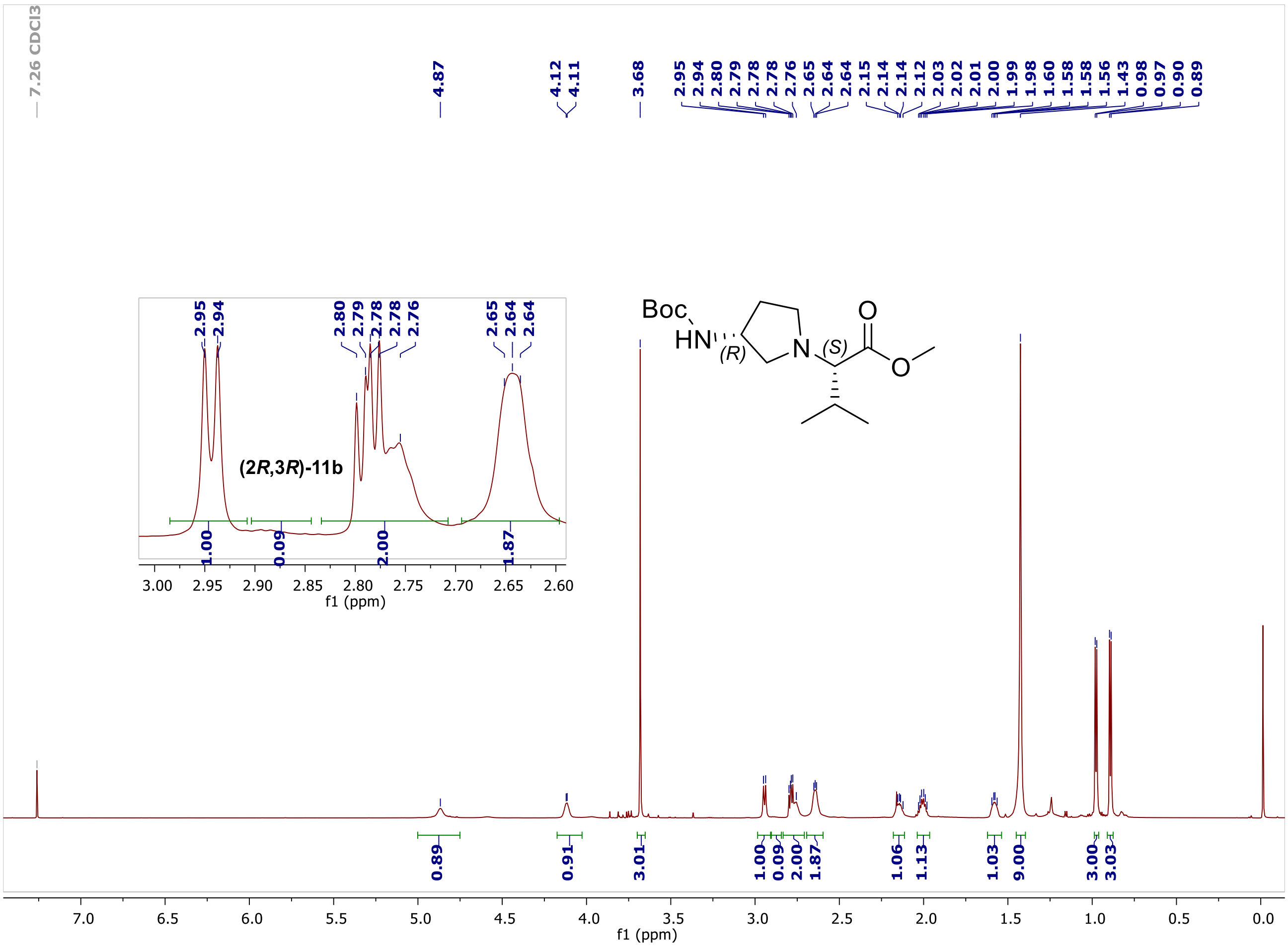


**Figure S111. Methyl (2*S*)-2-{(3*R*)-3-[(*tert*-butoxycarbonyl)amino]pyrrolidin-1-yl}-3-methylbutanoate ((2*S*,3*R*)-11b). $^{1}$H NMR spectrum (700 MHz, $CDCl_3$).**

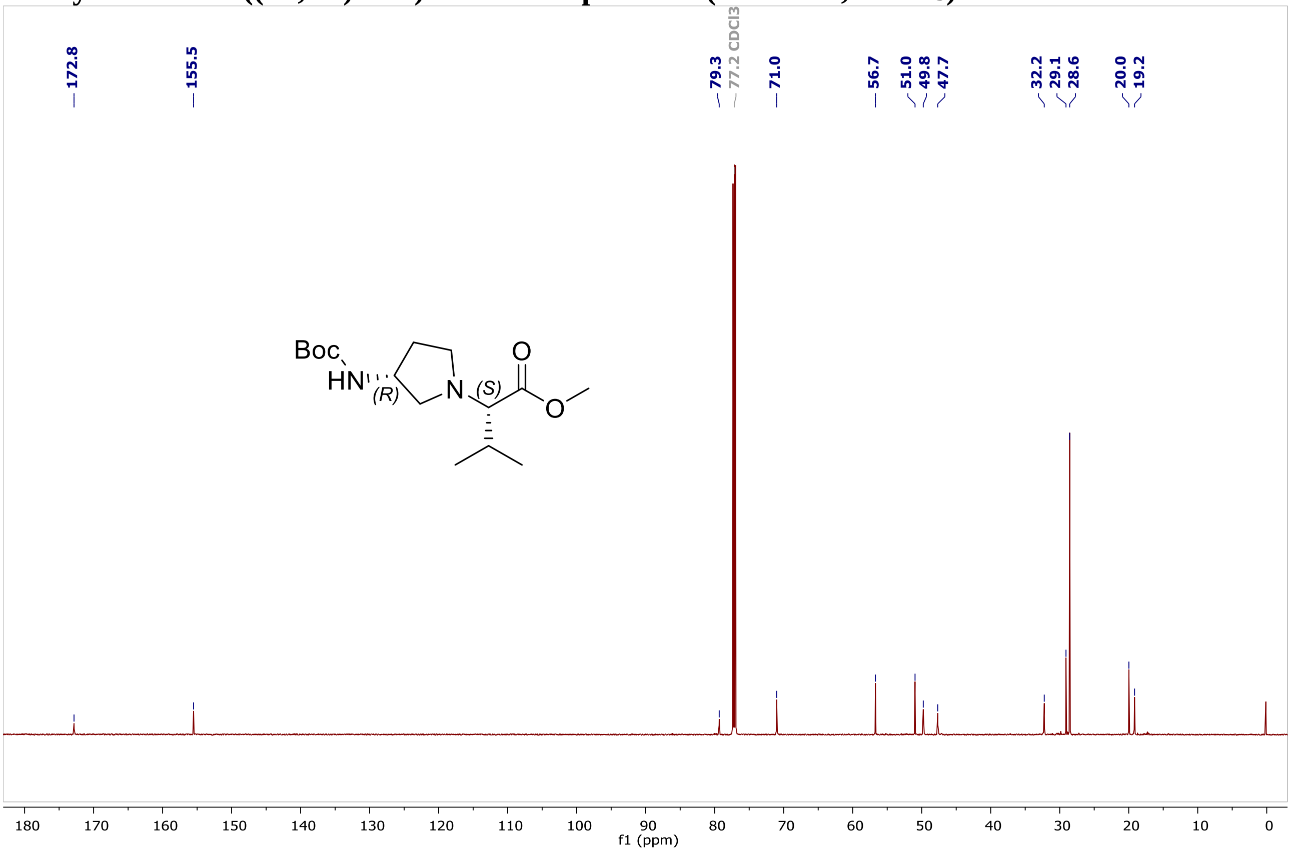


**Figure S112. Methyl (2*S*)-2-{(3*R*)-3-[(*tert*-butoxycarbonyl)amino]pyrrolidin-1-yl}-3-methylbutanoate ((2*S*,3*R*)-11b). $^{13}$C NMR spectrum (176 MHz, $CDCl_3$).**

# Mass Spectrum SmartFormula Report

**Analysis Info**

| | | | |
|---|---|---|---|
| Analysis Name | D:\Data\Organikai\2023_04_11\GMP_576_1-A,5_01_10210.d | Acquisition Date | 4/14/2023 12:52:37 PM |
| Method | organikai_esi_pos_2013_recover.m | Operator | Milda Pukalskiene |
| Sample Name | GMP_576 | Instrument / Ser# | maXis 4G 20218 |
| Comment | | | |

**Acquisition Parameter**

| | | | | | |
|---|---|---|---|---|---|
| Source Type | ESI | Ion Polarity | Positive | Set Nebulizer | 1.5 Bar |
| Focus | Not active | Set Capillary | 4500 V | Set Dry Heater | 180 °C |
| Scan Begin | 40 m/z | Set End Plate Offset | -500 V | Set Dry Gas | 8.0 l/min |
| Scan End | 1800 m/z | Set Collision Cell RF | 350.0 Vpp | Set Divert Valve | Waste |

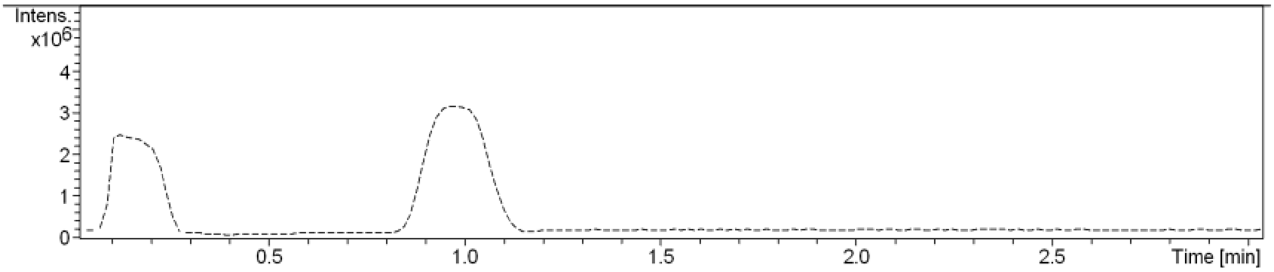


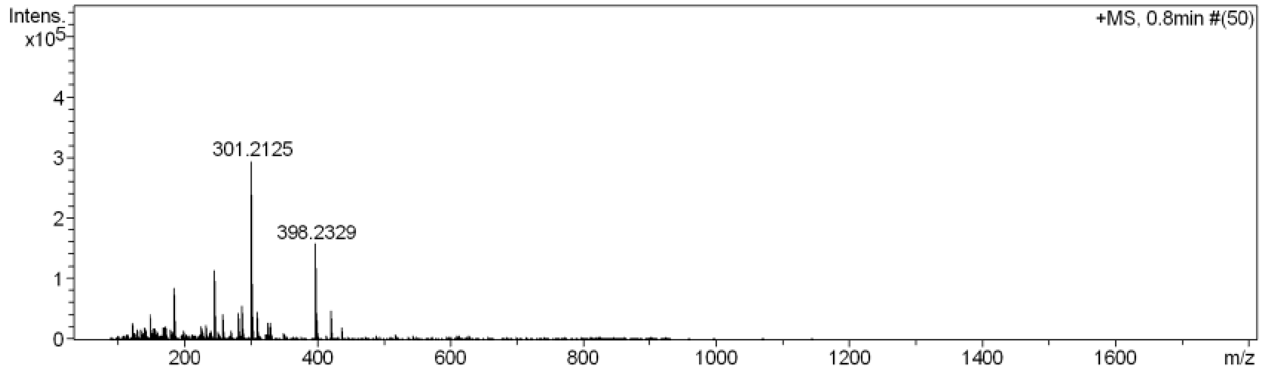


| Meas. m/z | # | Formula | Score | m/z | err [ppm] | Mean err [ppm] | mSigma | rdb | e⁻ Conf | N-Rule |
|---|---|---|---|---|---|---|---|---|---|---|
| 301.2125 | 1 | C 15 H 29 N 2 O 4 | 100.00 | 301.2122 | -0.9 | -0.7 | 13.2 | 2.5 | even | ok |

**Figure S113. Methyl (2*S*)-2-{(3*R*)-3-[(*tert*-butoxycarbonyl)amino]pyrrolidin-1-yl}-3-methylbutanoate ((2*S*,3*R*)-11b). HRMS (ESI-TOF).**

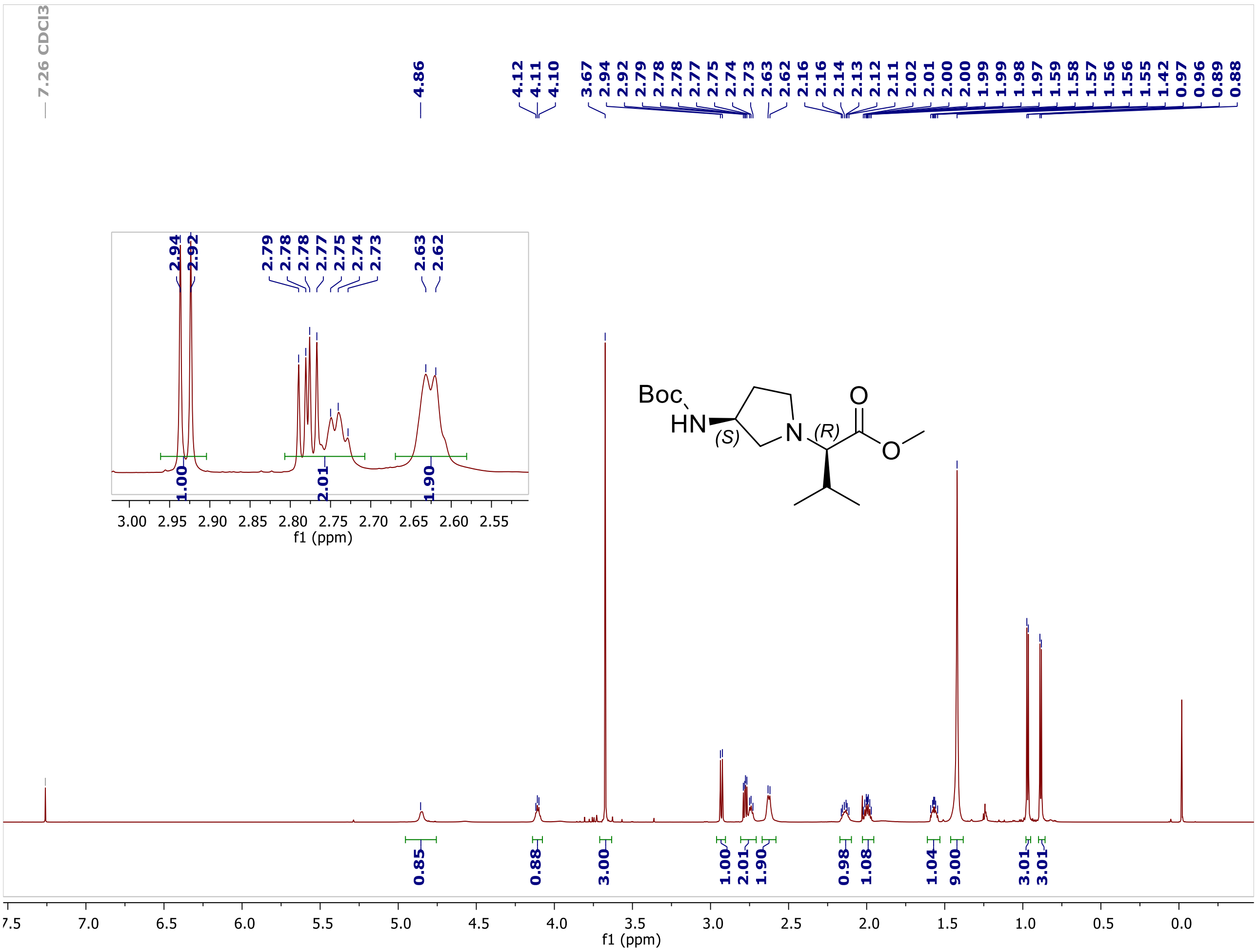


**Figure S114. Methyl (2*R*)-2-{(3*S*)-3-[(*tert*-butoxycarbonyl)amino]pyrrolidin-1-yl}-3-methylbutanoate ((2*R*,3*S*)-11b). $^{1}$H NMR spectrum (700 MHz, $CDCl_3$).**

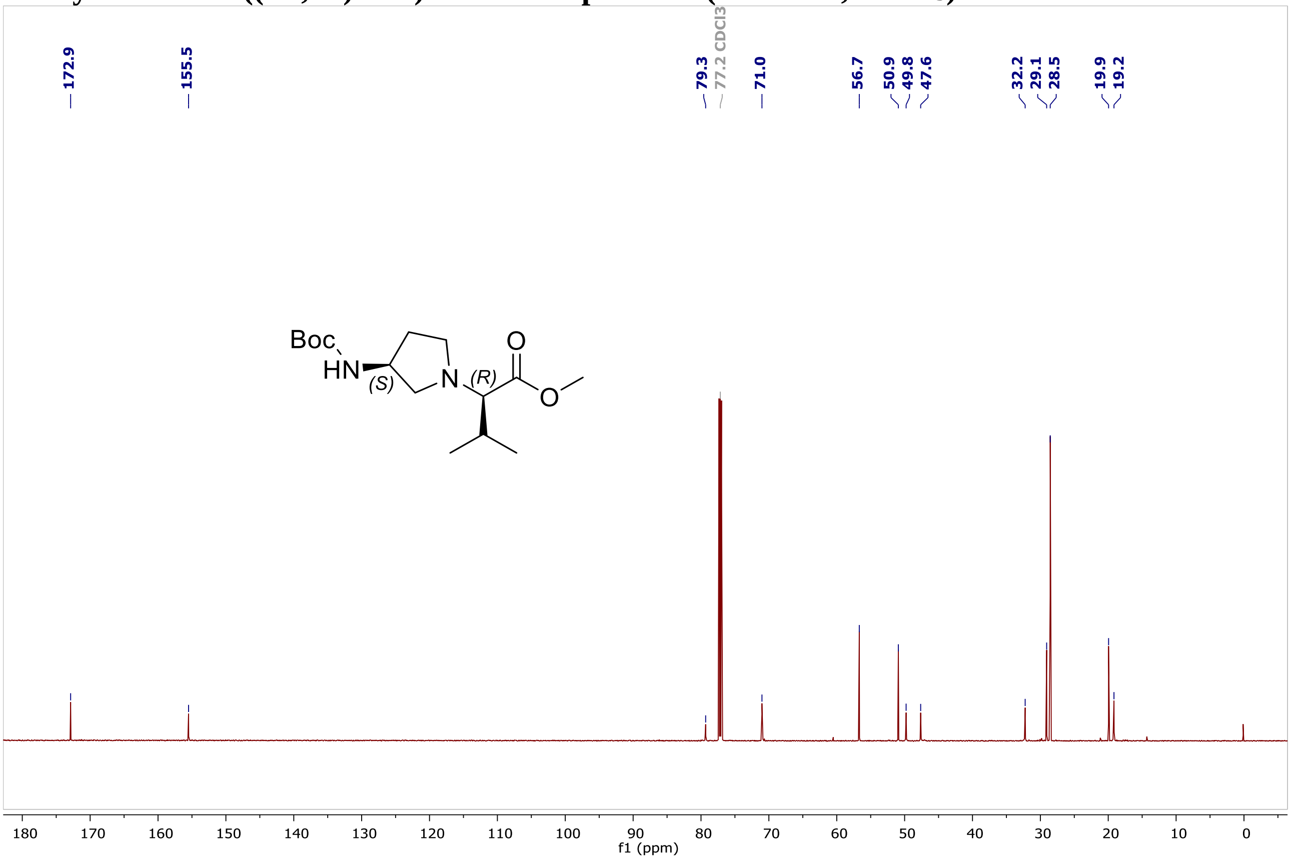


**Figure S115. Methyl (2*R*)-2-{(3*S*)-3-[(*tert*-butoxycarbonyl)amino]pyrrolidin-1-yl}-3-methylbutanoate ((2*R*,3*S*)-11b). $^{13}$C NMR spectrum (176 MHz, $CDCl_3$).**

# Mass Spectrum SmartFormula Report

**Analysis Info**

| | | | |
|---|---|---|---|
| Analysis Name | D:\Data\Organikai\2023_04_11\GMP_566_1-A,2_01_10203.d | Acquisition Date | 4/13/2023 11:07:30 AM |
| Method | organikai_esi_pos_2013_recover.m | Operator | Milda Pukalskiene |
| Sample Name | GMP_566 | Instrument / Ser# | maXis 4G 20218 |
| Comment | | | |

**Acquisition Parameter**

| | | | | | |
|---|---|---|---|---|---|
| Source Type | ESI | Ion Polarity | Positive | Set Nebulizer | 1.5 Bar |
| Focus | Not active | Set Capillary | 4500 V | Set Dry Heater | 180 °C |
| Scan Begin | 40 m/z | Set End Plate Offset | -500 V | Set Dry Gas | 8.0 l/min |
| Scan End | 1800 m/z | Set Collision Cell RF | 350.0 Vpp | Set Divert Valve | Waste |

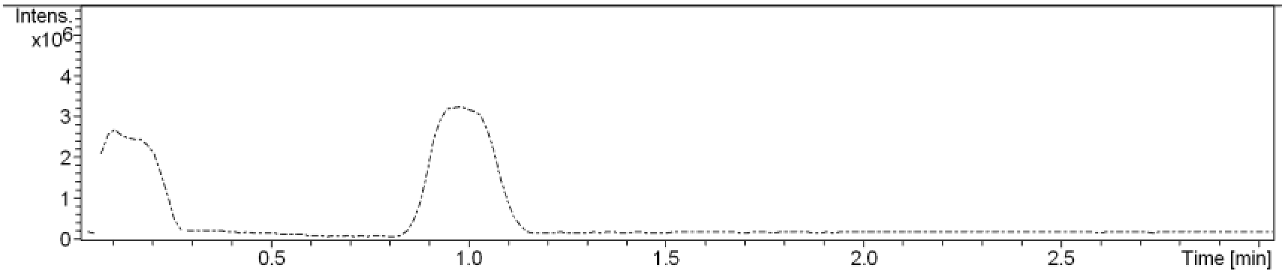


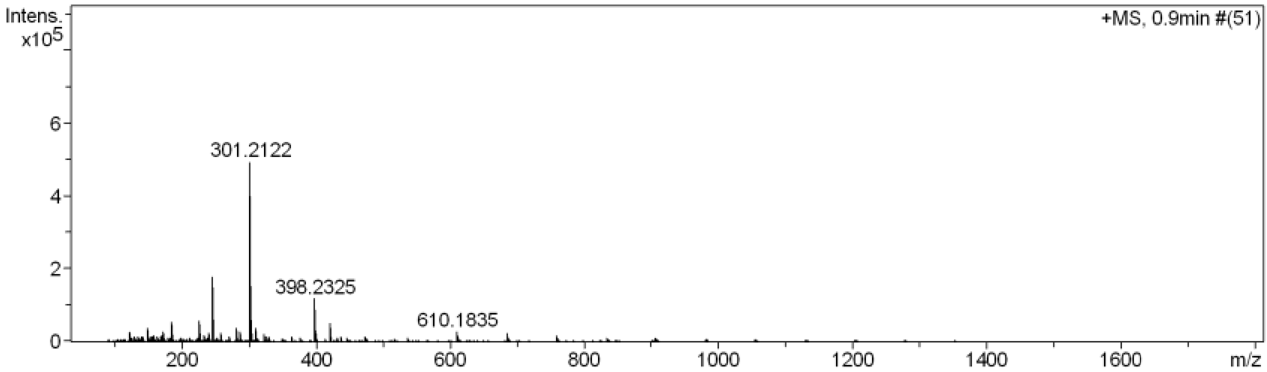


| Meas. m/z | # | Formula | Score | m/z | err [ppm] | Mean err [ppm] | mSigma | rdb | e⁻ Conf | N-Rule |
|---|---|---|---|---|---|---|---|---|---|---|
| 301.2122 | 1 | C 15 H 29 N 2 O 4 | 100.00 | 301.2122 | 0.0 | 0.2 | 16.8 | 2.5 | even | ok |

**Figure S116. Methyl (2*R*)-2-{(3*S*)-3-[(*tert*-butoxycarbonyl)amino]pyrrolidin-1-yl}-3-methylbutanoate ((2*R*,3*S*)-11b). HRMS (ESI-TOF).**

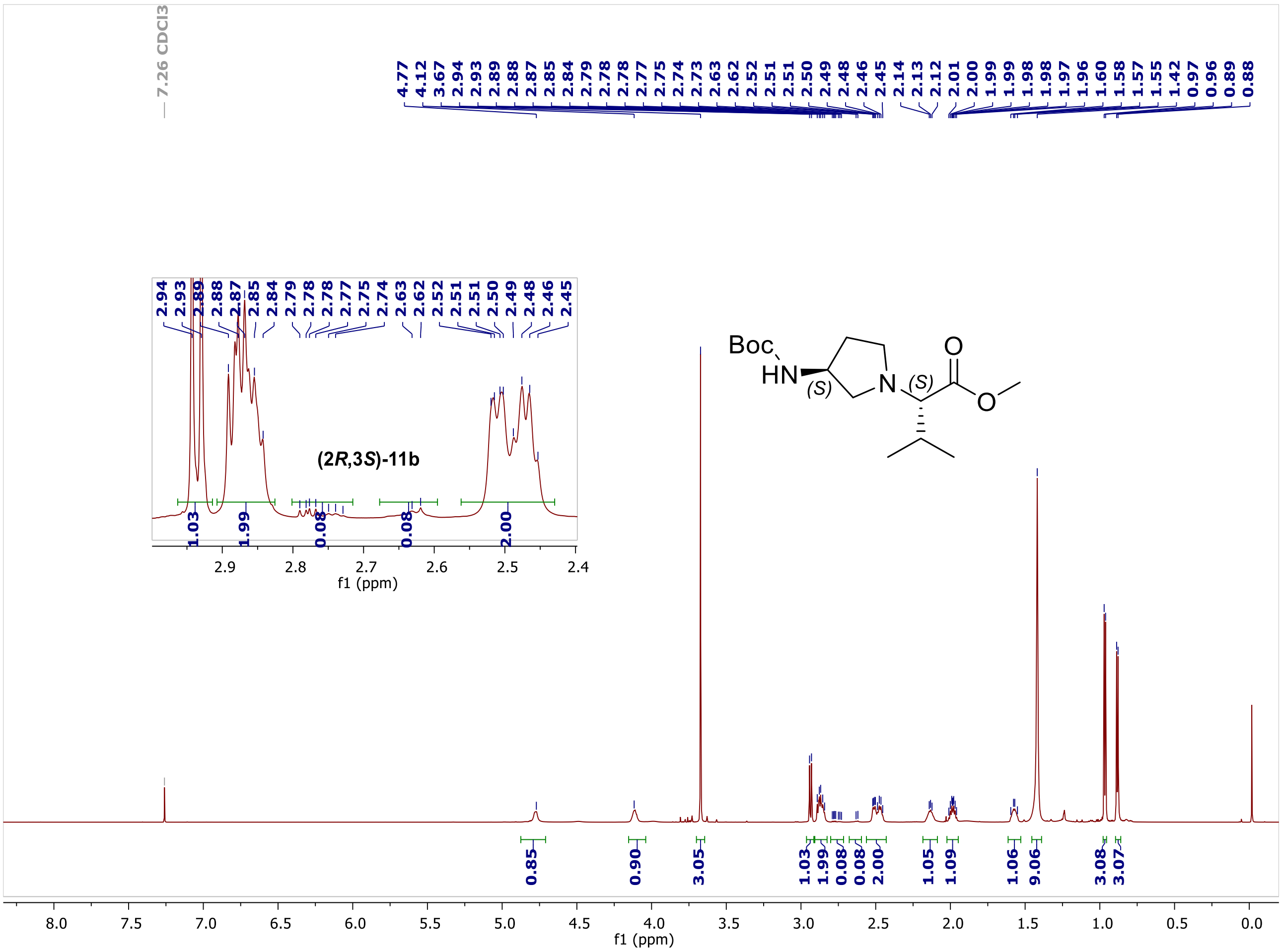

**Figure S117. Methyl (2*S*)-2-{(3*S*)-3-[(*tert*-butoxycarbonyl)amino]pyrrolidin-1-yl}-3-methylbutanoate ((2*S*,3*S*)-11b). $^{1}$H NMR spectrum (700 MHz, $CDCl_3$).**

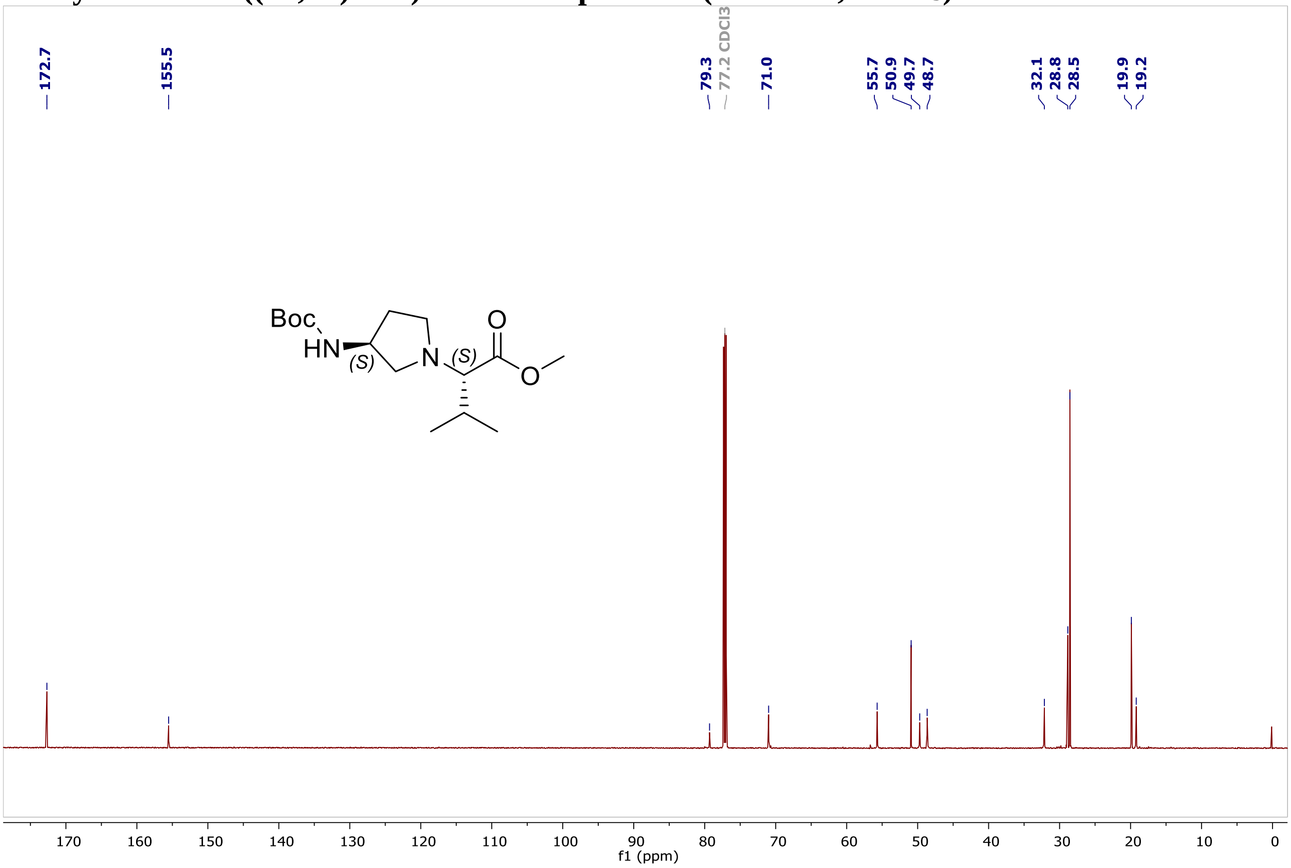

**Figure S118. Methyl (2*S*)-2-{(3*S*)-3-[(*tert*-butoxycarbonyl)amino]pyrrolidin-1-yl}-3-methylbutanoate ((2*S*,3*S*)-11b). $^{13}$C NMR spectrum (176 MHz, $CDCl_3$).**

# Compound Spectrum SmartFormula Report

**Analysis Info**

| | | | |
|---|---|---|---|
| Analysis Name | D:\Data\GMP-569.d | Acquisition Date | 5/2/2023 7:57:07 PM |
| Method | DirectInfusion_TuneLow_pos.m | Operator | hplc |
| Sample Name | GMP-569 | Instrument | micrOTOF-Q III 8228888.20448 |
| Comment | AB | | |

**Acquisition Parameter**

| | | | | | |
|---|---|---|---|---|---|
| Source Type | ESI | Ion Polarity | Positive | Set Nebulizer | 0.4 Bar |
| Focus | Not active | Set Capillary | 4500 V | Set Dry Heater | 180 °C |
| Scan Begin | 50 m/z | Set End Plate Offset | -500 V | Set Dry Gas | 4.0 l/min |
| Scan End | 1000 m/z | Set Collision Cell RF | 140.0 Vpp | Set Divert Valve | Waste |

| # | RT [min] | Area | Int. Type | I | S/N | Chromatogram | Max. m/z | FWHM [min] |
|---|---|---|---|---|---|---|---|---|
| n.a. | 6.0 | n.a. | Single spectrum | n.a. | n.a. | n.a. | 323.1940 | n.a. |

**+MS, 6.0min #360**

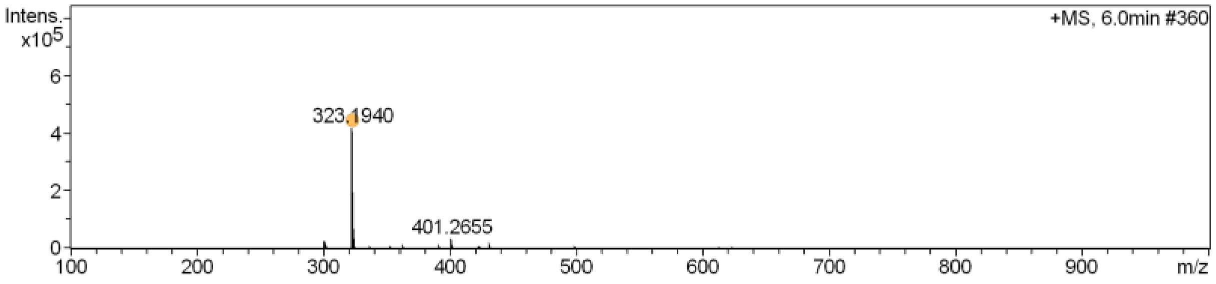


| Meas. m/z | # | Ion Formula | m/z | err [ppm] | mSigma | # Sigma | Score | rdb | e⁻ Conf | N-Rule |
|---|---|---|---|---|---|---|---|---|---|---|
| 323.1940 | 1 | C15H28N2NaO4 | 323.1941 | -0.5 | 2.4 | 1 | 100.00 | 2.5 | even | ok |

**Figure S119. Methyl (2*S*)-2-{(3*S*)-3-[(*tert*-butoxycarbonyl)amino]pyrrolidin-1-yl}-3-methylbutanoate ((2*S*,3*S*)-11b). HRMS (ESI-TOF).**

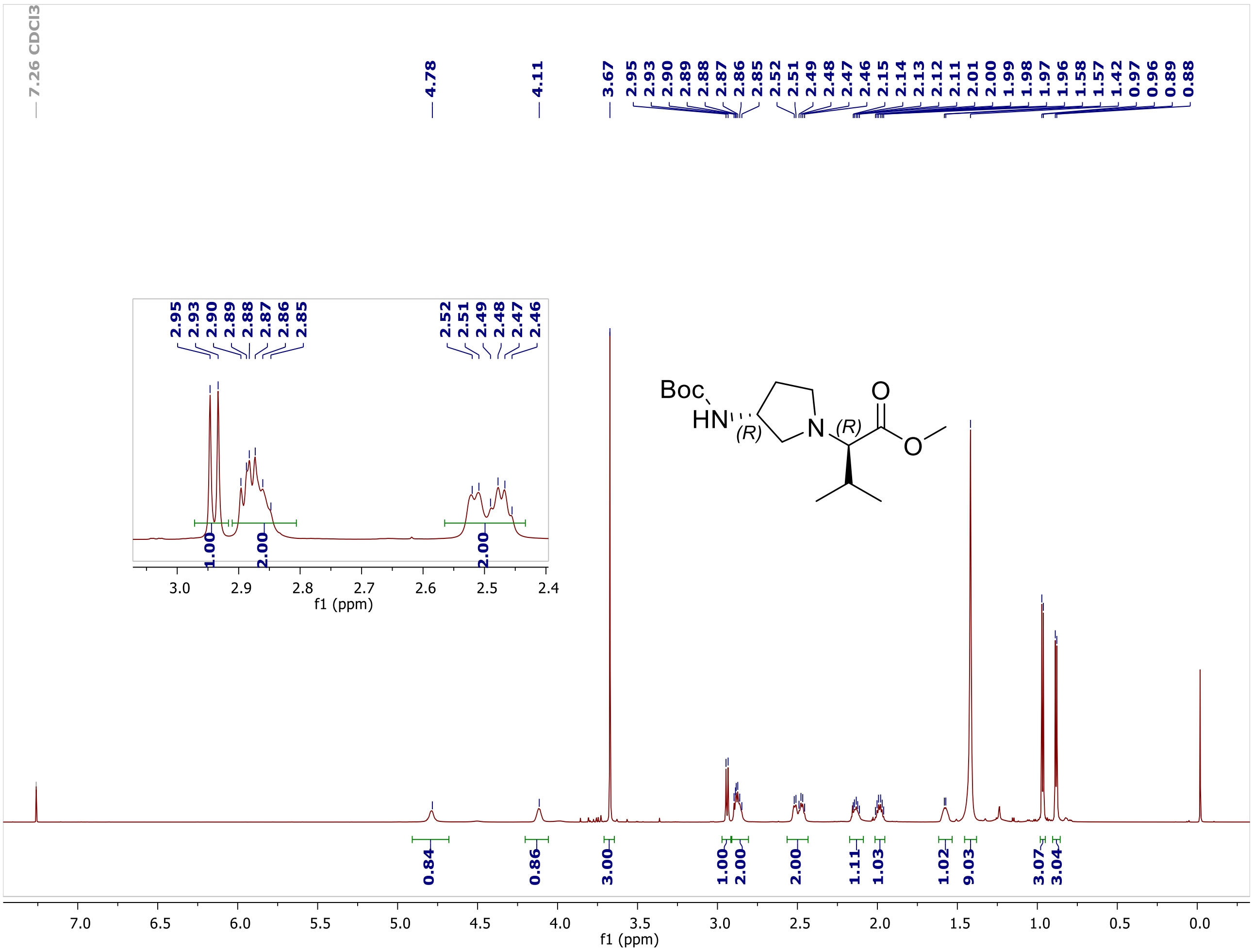

**Figure S120. Methyl (2*R*)-2-{(3*R*)-3-[(*tert*-butoxycarbonyl)amino]pyrrolidin-1-yl}-3-methylbutanoate ((2*R*,3*R*)-11b). $^{1}$H NMR spectrum (700 MHz, $CDCl_3$).**

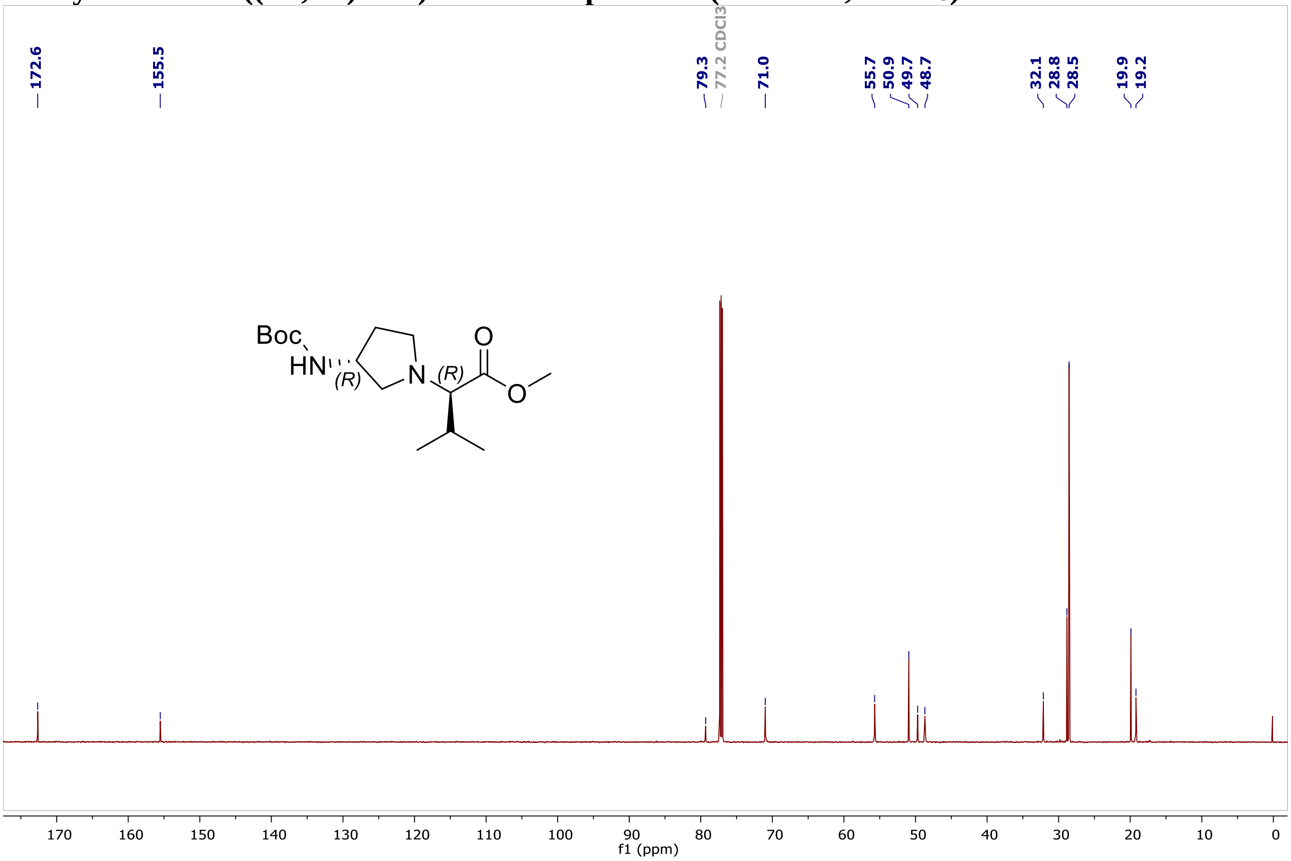

**Figure S121. Methyl (2*R*)-2-{(3*R*)-3-[(*tert*-butoxycarbonyl)amino]pyrrolidin-1-yl}-3-methylbutanoate ((2*R*,3*R*)-11b). $^{13}$C NMR spectrum (176 MHz, $CDCl_3$).**

# Mass Spectrum SmartFormula Report

**Analysis Info**

| | | | |
|---|---|---|---|
| Analysis Name | D:\Data\Organikai\2023_04_11\GMP_572_1-A,4_01_10209.d | Acquisition Date | 4/14/2023 12:48:09 PM |
| Method | organikai_esi_pos_2013_recover.m | Operator | Milda Pukalskiene |
| Sample Name | GMP_572 | Instrument / Ser# | maXis 4G 20218 |
| Comment | | | |

**Acquisition Parameter**

| | | | | | |
|---|---|---|---|---|---|
| Source Type | ESI | Ion Polarity | Positive | Set Nebulizer | 1.5 Bar |
| Focus | Not active | Set Capillary | 4500 V | Set Dry Heater | 180 °C |
| Scan Begin | 40 m/z | Set End Plate Offset | -500 V | Set Dry Gas | 8.0 l/min |
| Scan End | 1800 m/z | Set Collision Cell RF | 350.0 Vpp | Set Divert Valve | Waste |

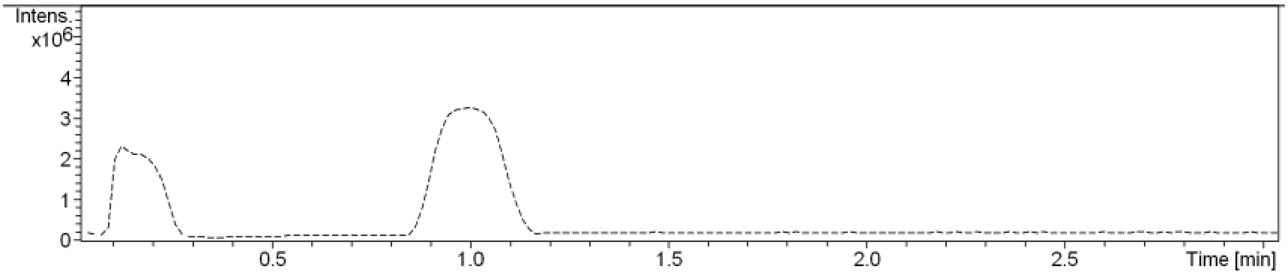


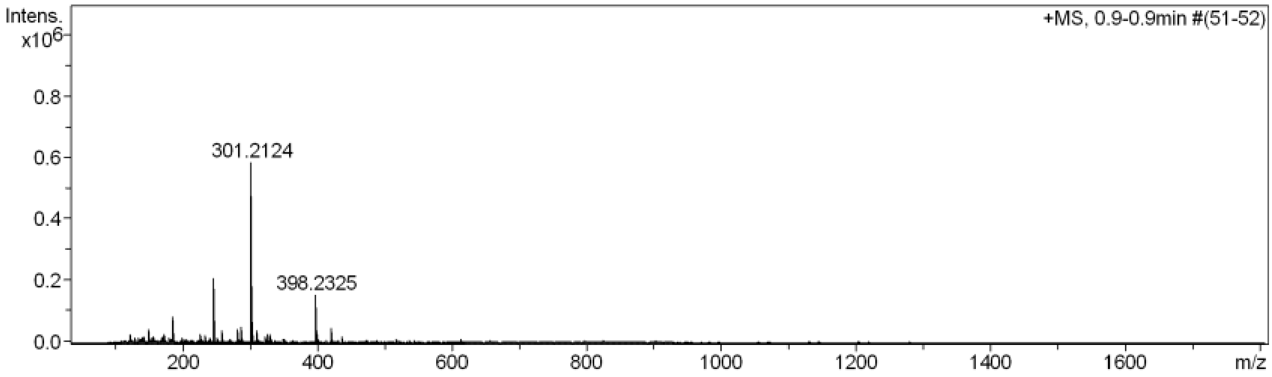


| Meas. m/z | # | Formula | Score | m/z | err [ppm] | Mean err [ppm] | mSigma | rdb | e⁻ Conf | N-Rule |
|---|---|---|---|---|---|---|---|---|---|---|
| 301.2124 | 1 | C 15 H 29 N 2 O 4 | 100.00 | 301.2122 | -0.6 | -0.3 | 15.7 | 2.5 | even | ok |

**Figure S122. Methyl (2*R*)-2-{(3*R*)-3-[(*tert*-butoxycarbonyl)amino]pyrrolidin-1-yl}-3-methylbutanoate ((2*R*,3*R*)-11b). HRMS (ESI-TOF).**

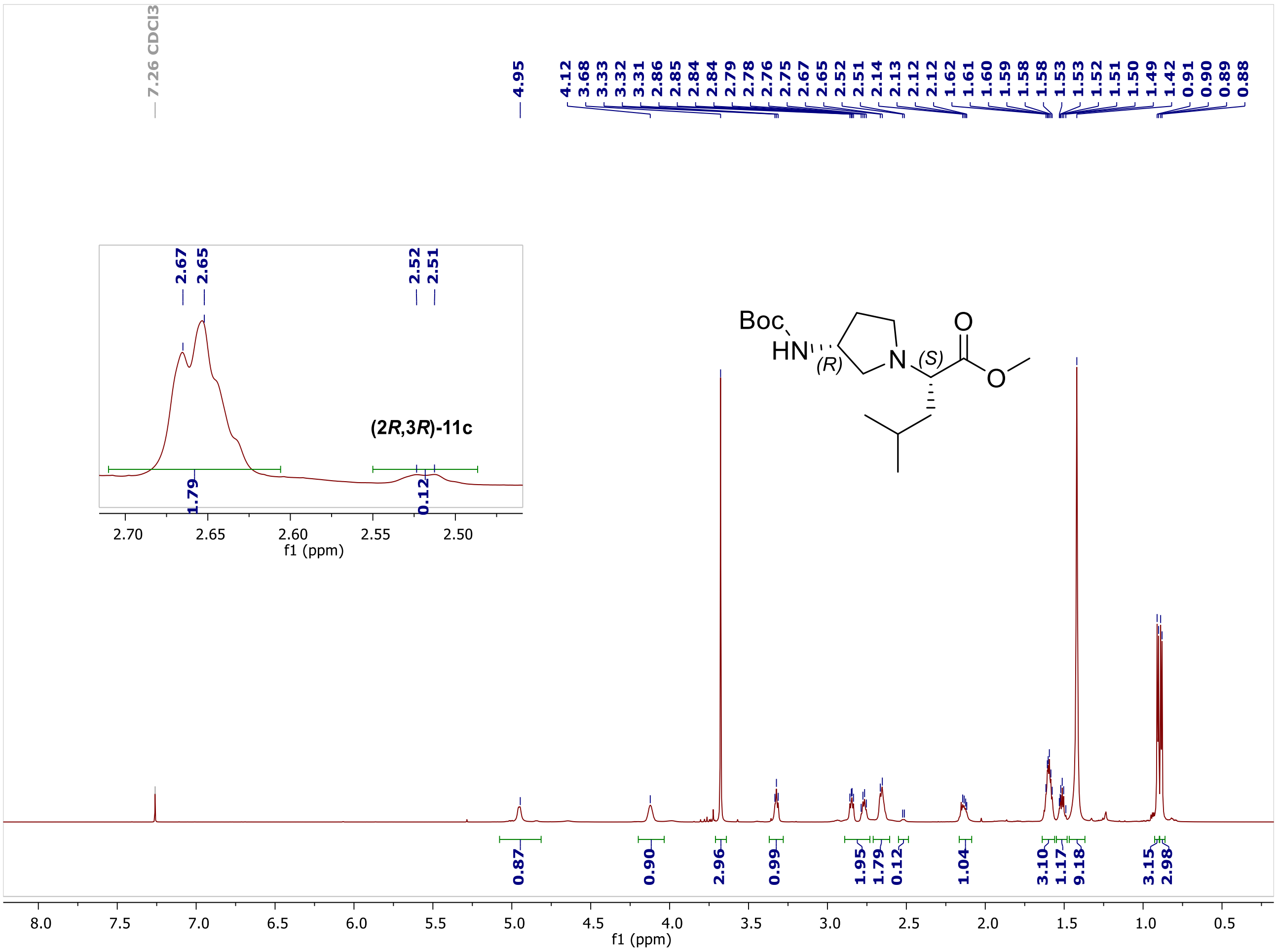

**Figure S123. Methyl (2*S*)-2-{(3*R*)-3-[(*tert*-butoxycarbonyl)amino]pyrrolidin-1-yl}-4-methylpentanoate ((2*S*,3*R*)-11c). $^{1}$H NMR spectrum (700 MHz, CDCl$_3$).**

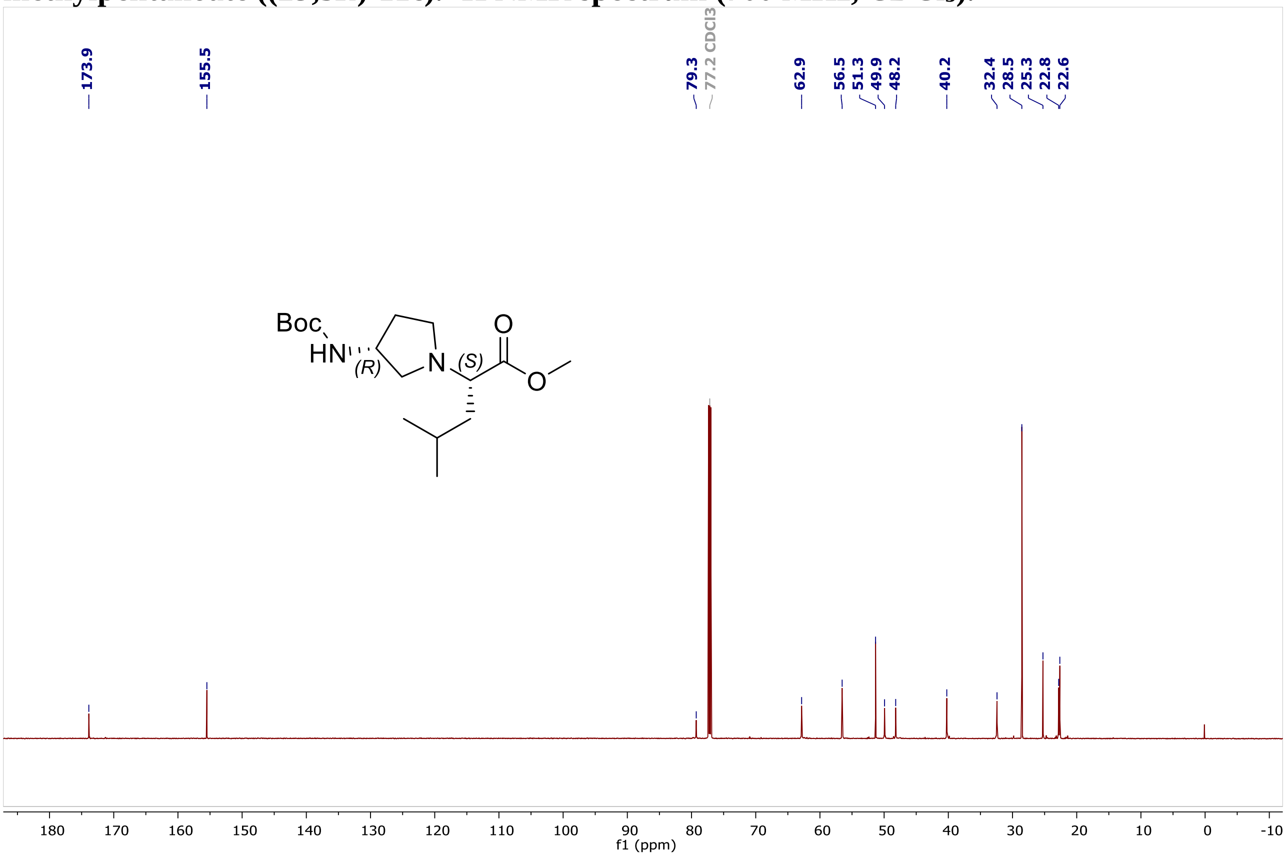

**Figure S124. Methyl (2*S*)-2-{(3*R*)-3-[(*tert*-butoxycarbonyl)amino]pyrrolidin-1-yl}-4-methylpentanoate ((2*S*,3*R*)-11c). $^{13}$C NMR spectrum (176 MHz, CDCl$_3$).**

# Mass Spectrum SmartFormula Report

**Analysis Info**

| | | | |
|---|---|---|---|
| Analysis Name | D:\Data\Organikai\2023_04_11\GMP_599_1-E,2_01_10259.d | Acquisition Date | 4/27/2023 3:30:54 PM |
| Method | organikai_esi_pos_2013_recover.m | Operator | Milda Pukalskiene |
| Sample Name | GMP_599 | Instrument / Ser# | maXis 4G 20218 |
| Comment | | | |

**Acquisition Parameter**

| | | | | | |
|---|---|---|---|---|---|
| Source Type | ESI | Ion Polarity | Positive | Set Nebulizer | 1.5 Bar |
| Focus | Not active | Set Capillary | 4500 V | Set Dry Heater | 180 °C |
| Scan Begin | 40 m/z | Set End Plate Offset | -500 V | Set Dry Gas | 8.0 l/min |
| Scan End | 1800 m/z | Set Collision Cell RF | 350.0 Vpp | Set Divert Valve | Waste |

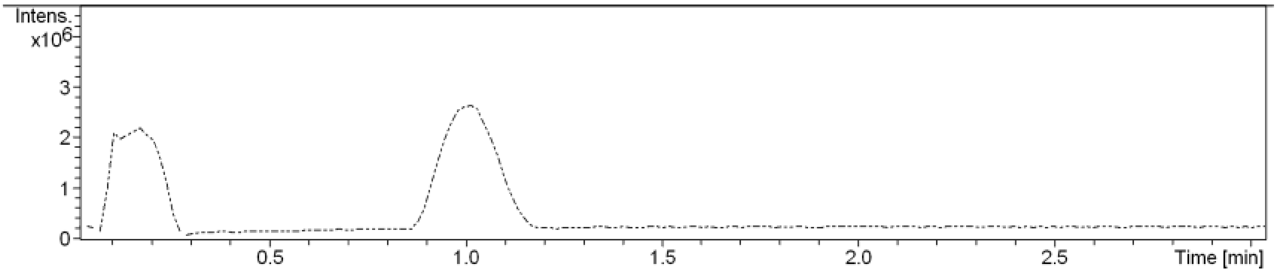


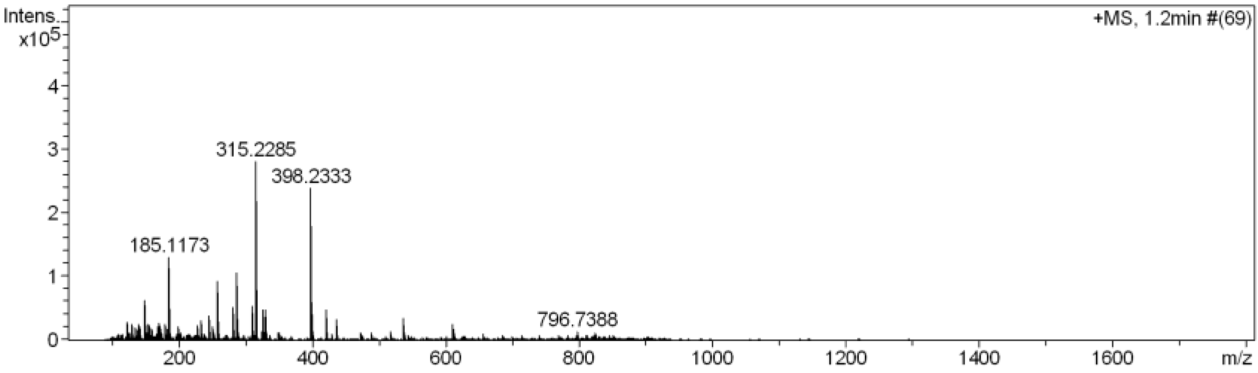


| Meas. m/z | # | Formula | Score | m/z | err [ppm] | Mean err [ppm] | mSigma | rdb | e⁻ Conf | N-Rule |
|---|---|---|---|---|---|---|---|---|---|---|
| 315.2285 | 1 | C 16 H 31 N 2 O 4 | 100.00 | 315.2278 | -2.0 | -1.8 | 15.0 | 2.5 | even | ok |

**Figure S125. Methyl (2*S*)-2-{(3*R*)-3-[(*tert*-butoxycarbonyl)amino]pyrrolidin-1-yl}-4-methylpentanoate ((2*S*,3*R*)-11c). HRMS (ESI-TOF).**

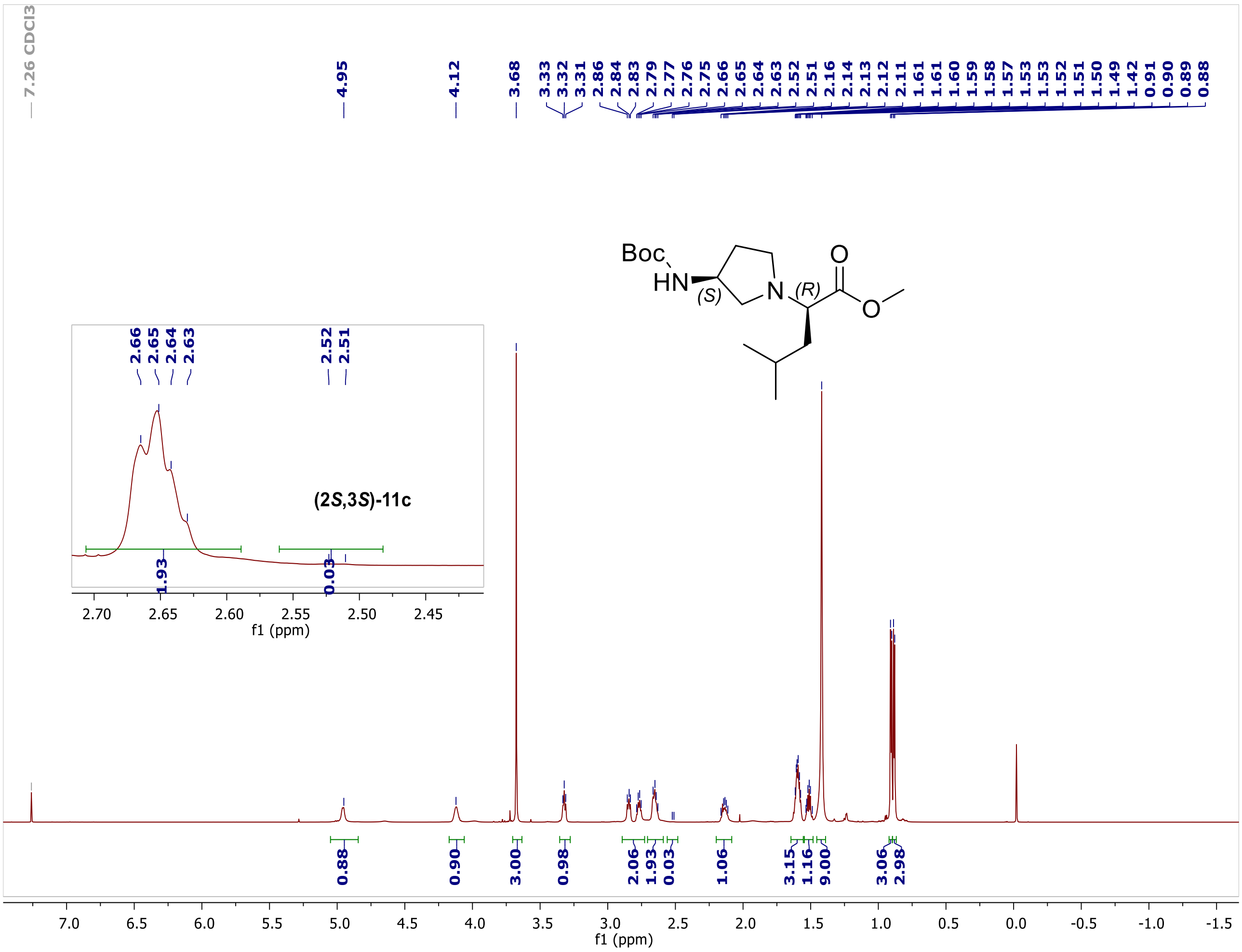

**Figure S126. Methyl (2*R*)-2-{(3*S*)-3-[(*tert*-butoxycarbonyl)amino]pyrrolidin-1-yl}-4-methylpentanoate ((2*R*,3*S*)-11c). $^{1}$H NMR spectrum (700 MHz, $CDCl_3$).**

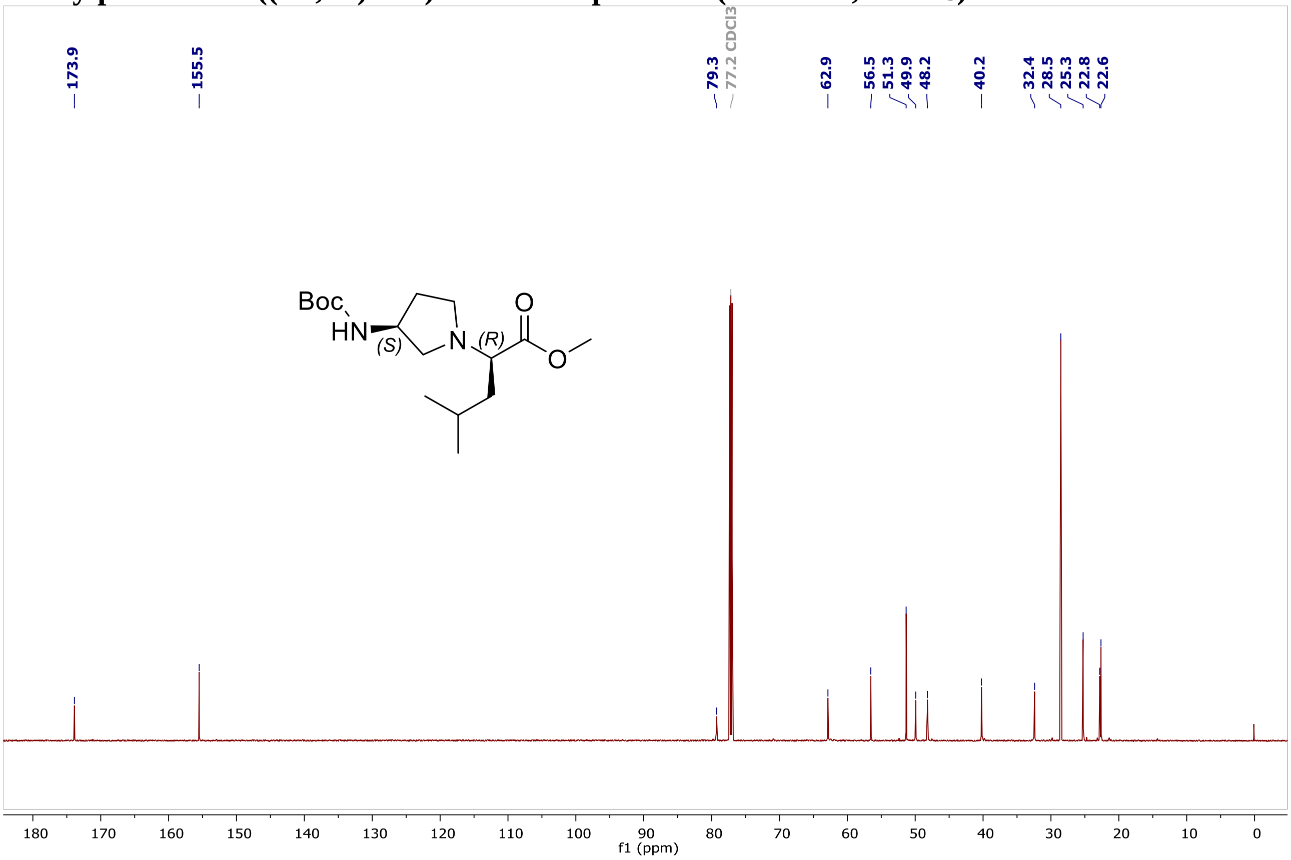

**Figure S127. Methyl (2*R*)-2-{(3*S*)-3-[(*tert*-butoxycarbonyl)amino]pyrrolidin-1-yl}-4-methylpentanoate ((2*R*,3*S*)-11c). $^{13}$C NMR spectrum (176 MHz, $CDCl_3$).**

# Mass Spectrum SmartFormula Report

**Analysis Info**

| | | | |
|---|---|---|---|
| Analysis Name | D:\Data\Organikai\2023_04_11\GMP_592_1-E,1_01_10258.d | Acquisition Date | 4/27/2023 3:26:28 PM |
| Method | organikai_esi_pos_2013_recover.m | Operator | Milda Pukalskiene |
| Sample Name | GMP_592 | Instrument / Ser# | maXis 4G 20218 |
| Comment | | | |

**Acquisition Parameter**

| | | | | | |
|---|---|---|---|---|---|
| Source Type | ESI | Ion Polarity | Positive | Set Nebulizer | 1.5 Bar |
| Focus | Not active | Set Capillary | 4500 V | Set Dry Heater | 180 °C |
| Scan Begin | 40 m/z | Set End Plate Offset | -500 V | Set Dry Gas | 8.0 l/min |
| Scan End | 1800 m/z | Set Collision Cell RF | 350.0 Vpp | Set Divert Valve | Waste |

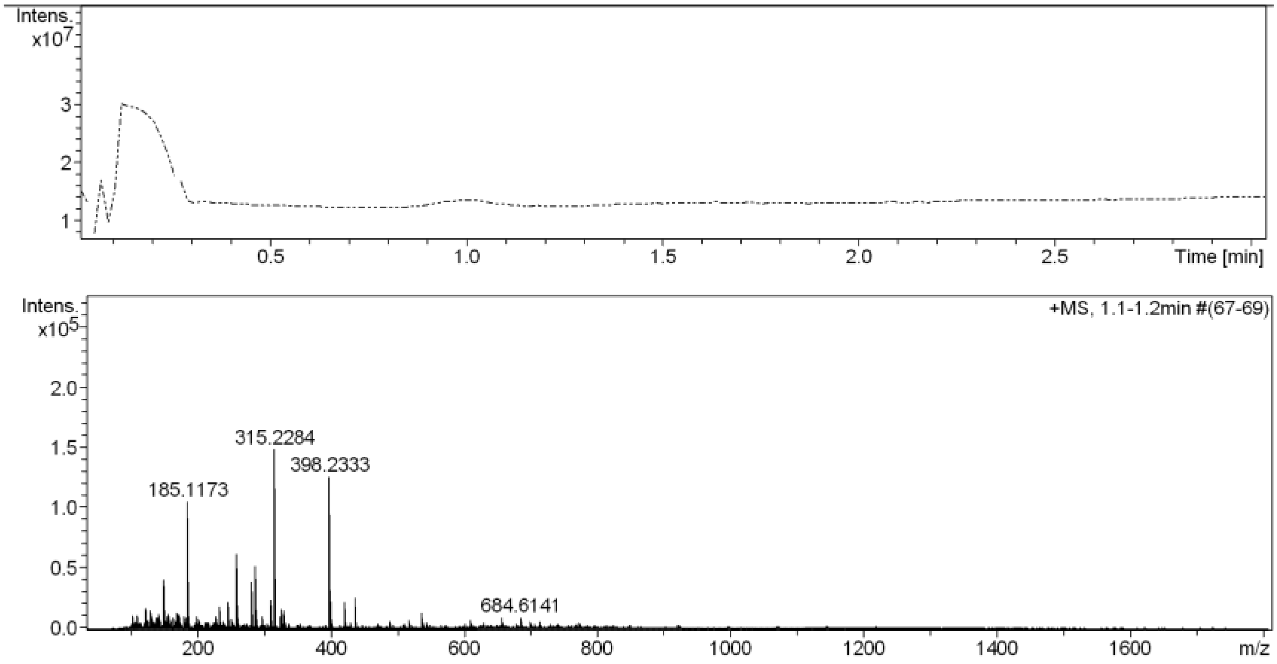


| Meas. m/z | # | Formula | Score | m/z | err [ppm] | Mean err [ppm] | mSigma | rdb | e⁻ Conf | N-Rule |
|---|---|---|---|---|---|---|---|---|---|---|
| 315.2284 | 1 | C 16 H 31 N 2 O 4 | 100.00 | 315.2278 | -1.8 | -1.6 | 7.3 | 2.5 | even | ok |

**Figure S128. Methyl (2*R*)-2-{(3*S*)-3-[(*tert*-butoxycarbonyl)amino]pyrrolidin-1-yl}-4-methylpentanoate ((2*R*,3*S*)-11c). HRMS (ESI-TOF).**

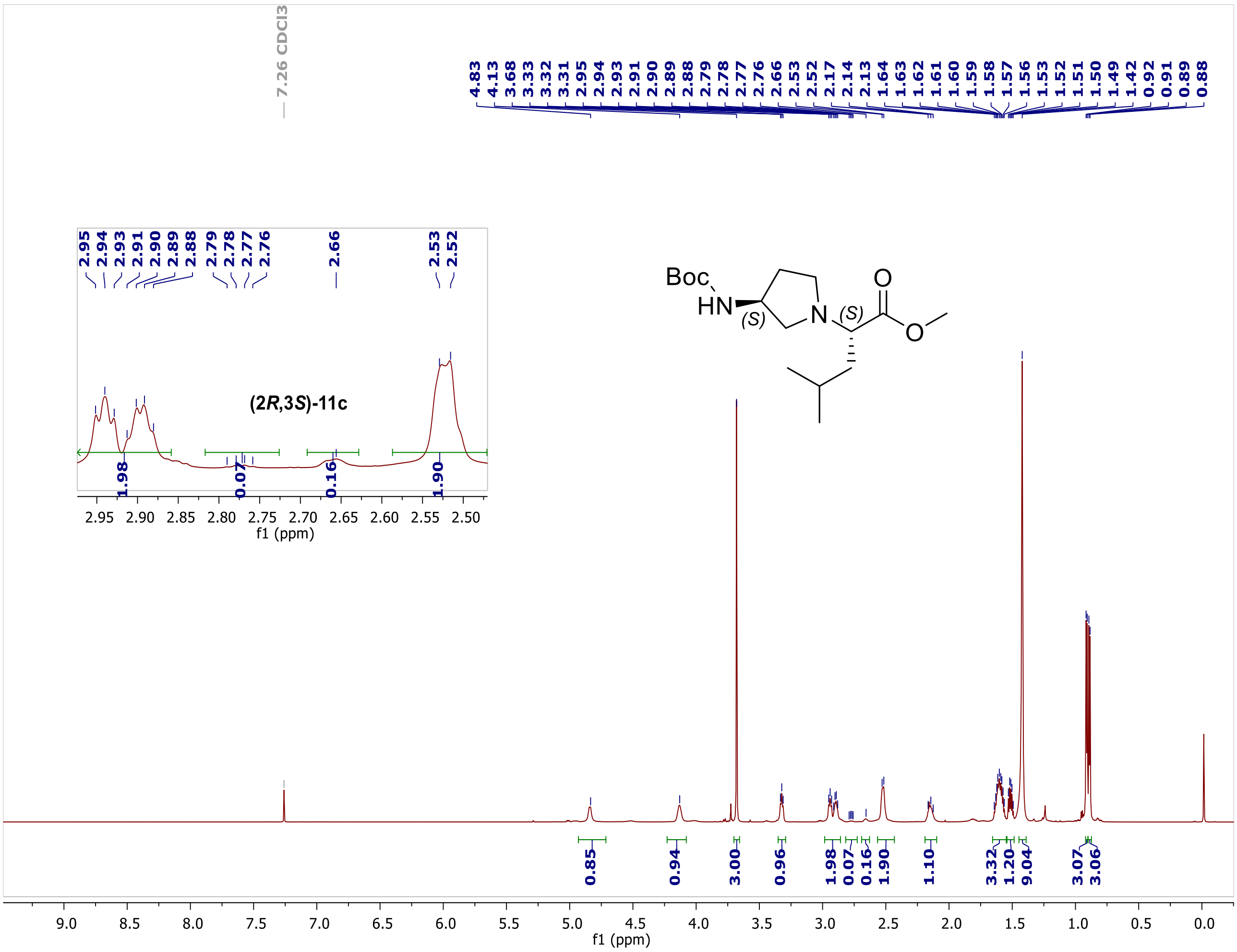

**Figure S129. Methyl (2*S*)-2-{(3*S*)-3-[(*tert*-butoxycarbonyl)amino]pyrrolidin-1-yl}-4-methylpentanoate ((2*S*,3*S*)-11c). $^{1}$H NMR spectrum (700 MHz, $CDCl_3$).**

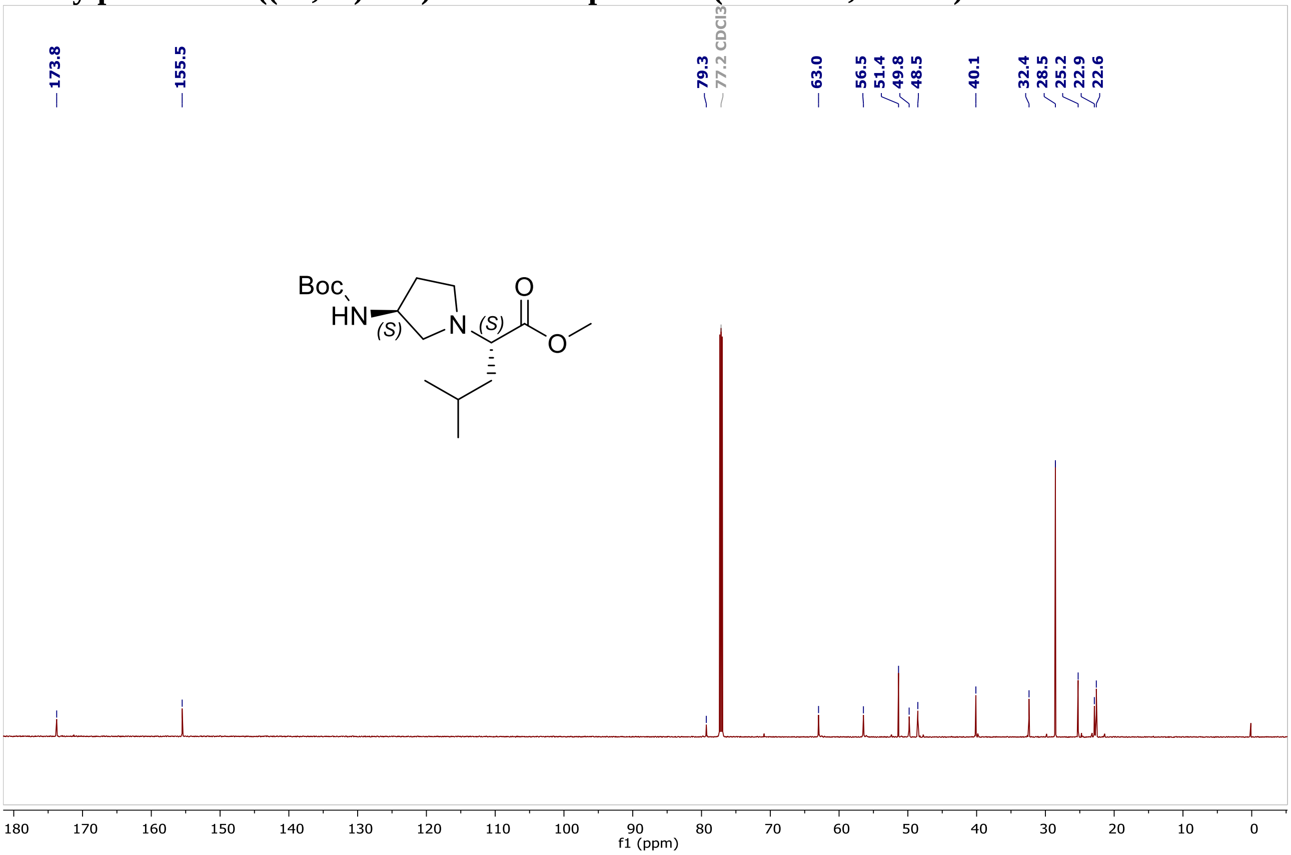

**Figure S130. Methyl (2*S*)-2-{(3*S*)-3-[(*tert*-butoxycarbonyl)amino]pyrrolidin-1-yl}-4-methylpentanoate ((2*S*,3*S*)-11c). $^{13}$C NMR spectrum (176 MHz, $CDCl_3$).**

# Mass Spectrum SmartFormula Report

**Analysis Info**

| | | | |
|---|---|---|---|
| Analysis Name | D:\Data\Organikai\2023_04_11\GMP_597_1-D,8_01_10238.d | Acquisition Date | 4/21/2023 2:36:49 PM |
| Method | organikai_esi_pos_2013_recover.m | Operator | Milda Pukalskiene |
| Sample Name | GMP_597 | Instrument / Ser# | maXis 4G 20218 |
| Comment | | | |

**Acquisition Parameter**

| | | | | | |
|---|---|---|---|---|---|
| Source Type | ESI | Ion Polarity | Positive | Set Nebulizer | 1.5 Bar |
| Focus | Not active | Set Capillary | 4500 V | Set Dry Heater | 180 °C |
| Scan Begin | 40 m/z | Set End Plate Offset | -500 V | Set Dry Gas | 8.0 l/min |
| Scan End | 1800 m/z | Set Collision Cell RF | 350.0 Vpp | Set Divert Valve | Waste |

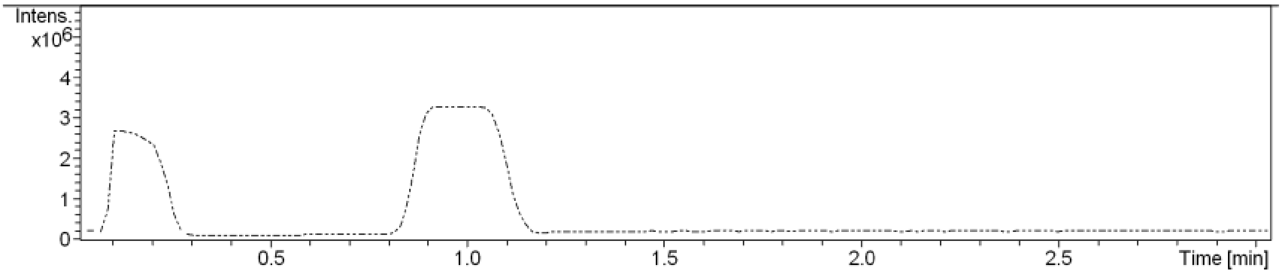


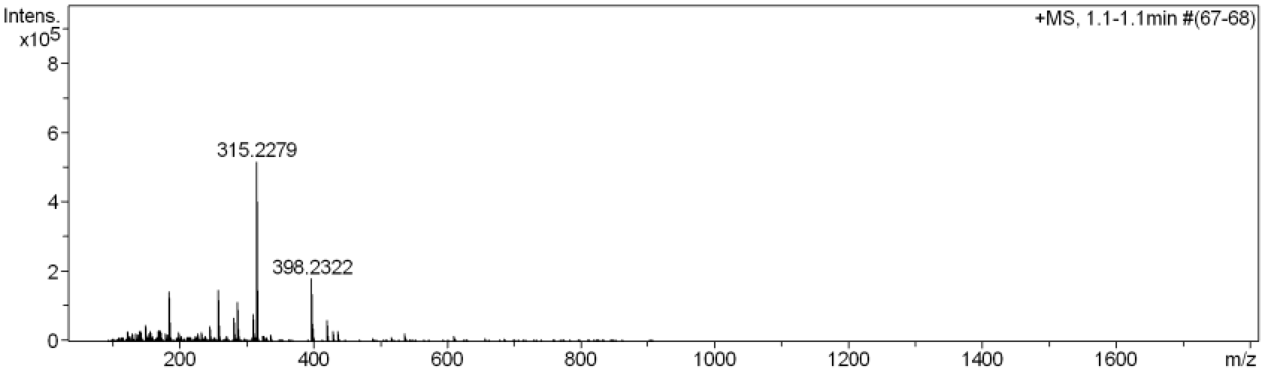


| Meas. m/z | # | Formula | Score | m/z | err [ppm] | Mean err [ppm] | mSigma | rdb | e⁻ Conf | N-Rule |
|---|---|---|---|---|---|---|---|---|---|---|
| 315.2279 | 1 | C 16 H 31 N 2 O 4 | 100.00 | 315.2278 | -0.3 | 0.1 | 16.0 | 2.5 | even | ok |
| | 2 | C 17 H 27 N 6 | 41.89 | 315.2292 | 4.0 | 4.3 | 28.5 | 7.5 | even | ok |

**Figure S131. Methyl (2*S*)-2-{(3*S*)-3-[(*tert*-butoxycarbonyl)amino]pyrrolidin-1-yl}-4-methylpentanoate ((2*S*,3*S*)-11c). HRMS (ESI-TOF).**

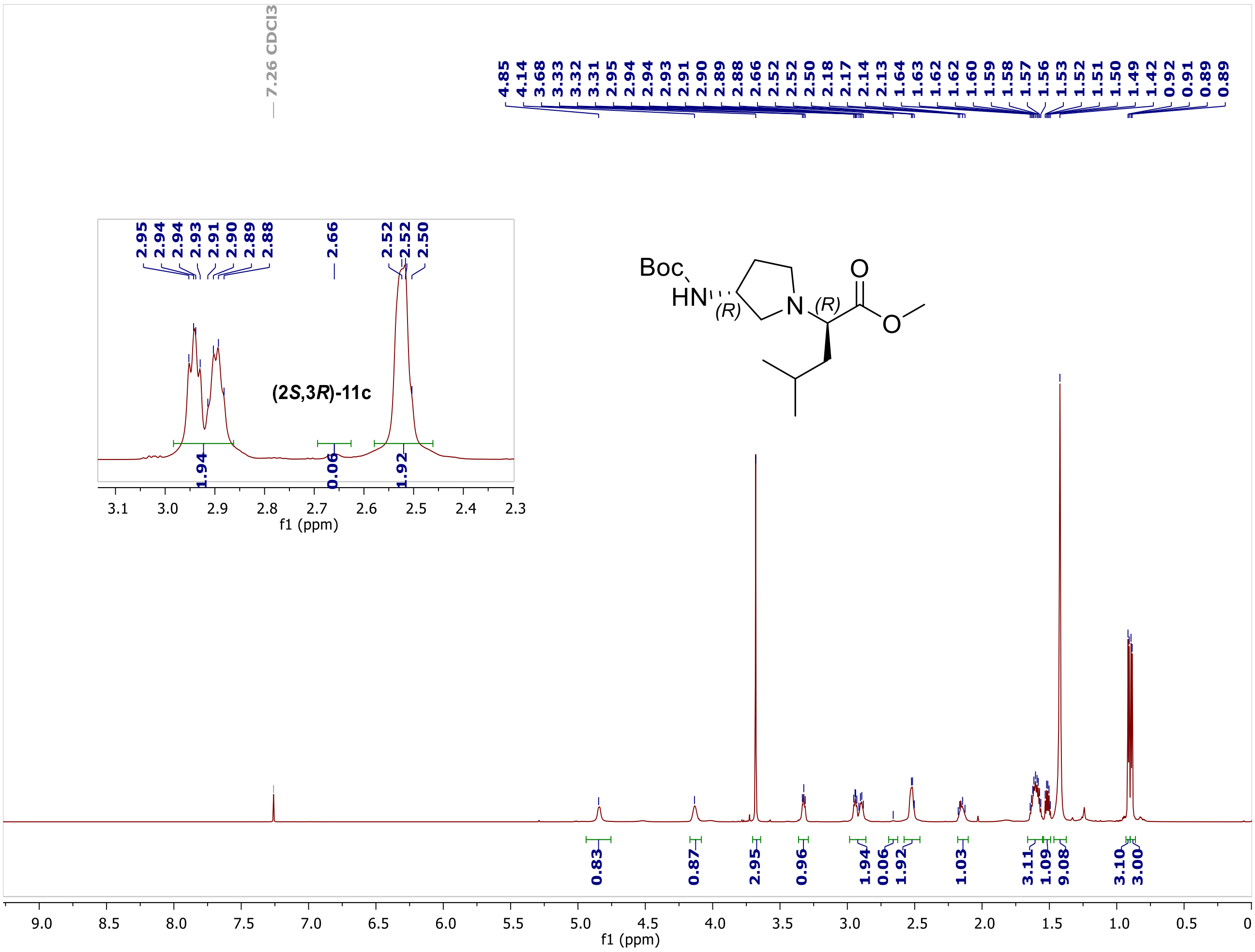

**Figure S132. Methyl (2*R*)-2-{(3*R*)-3-[(*tert*-butoxycarbonyl)amino]pyrrolidin-1-yl}-4-methylpentanoate ((2*R*,3*R*)-11c). $^{1}$H NMR spectrum (700 MHz, $CDCl_3$).**

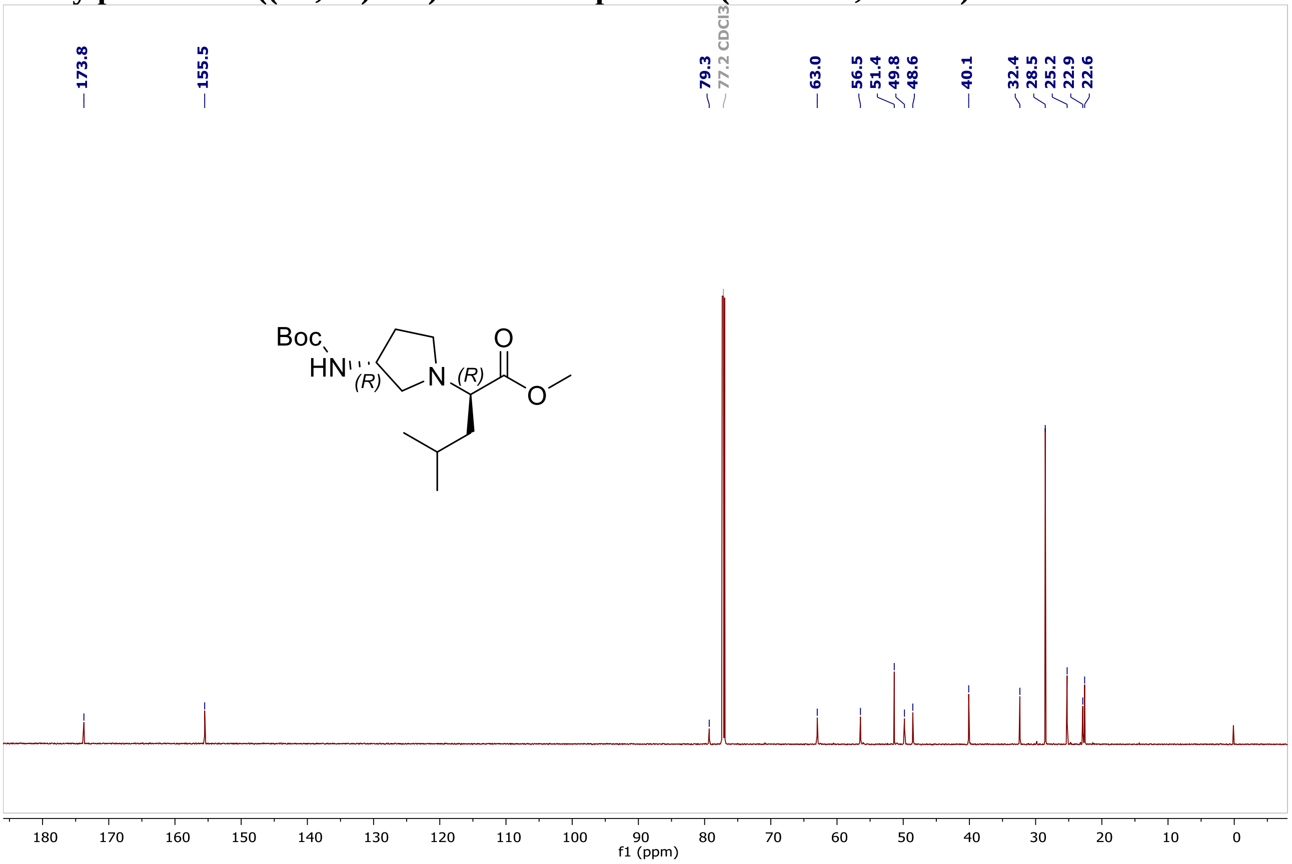

**Figure S133. Methyl (2*R*)-2-{(3*R*)-3-[(*tert*-butoxycarbonyl)amino]pyrrolidin-1-yl}-4-methylpentanoate ((2*R*,3*R*)-11c). $^{13}$C NMR spectrum (176 MHz, $CDCl_3$).**

# Mass Spectrum SmartFormula Report

**Analysis Info**

| | | | |
|---|---|---|---|
| Analysis Name | D:\Data\Organikai\2023_04_11\GMP_594_1-E,3_01_10260.d | Acquisition Date | 4/27/2023 3:35:22 PM |
| Method | organikai_esi_pos_2013_recover.m | Operator | Milda Pukalskiene |
| Sample Name | GMP_594 | Instrument / Ser# | maXis 4G 20218 |
| Comment | | | |

**Acquisition Parameter**

| | | | | | |
|---|---|---|---|---|---|
| Source Type | ESI | Ion Polarity | Positive | Set Nebulizer | 1.5 Bar |
| Focus | Not active | Set Capillary | 4500 V | Set Dry Heater | 180 °C |
| Scan Begin | 40 m/z | Set End Plate Offset | -500 V | Set Dry Gas | 8.0 l/min |
| Scan End | 1800 m/z | Set Collision Cell RF | 350.0 Vpp | Set Divert Valve | Waste |

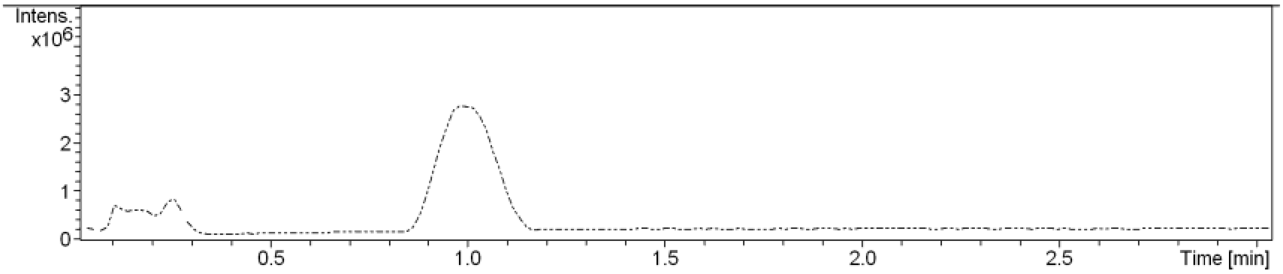


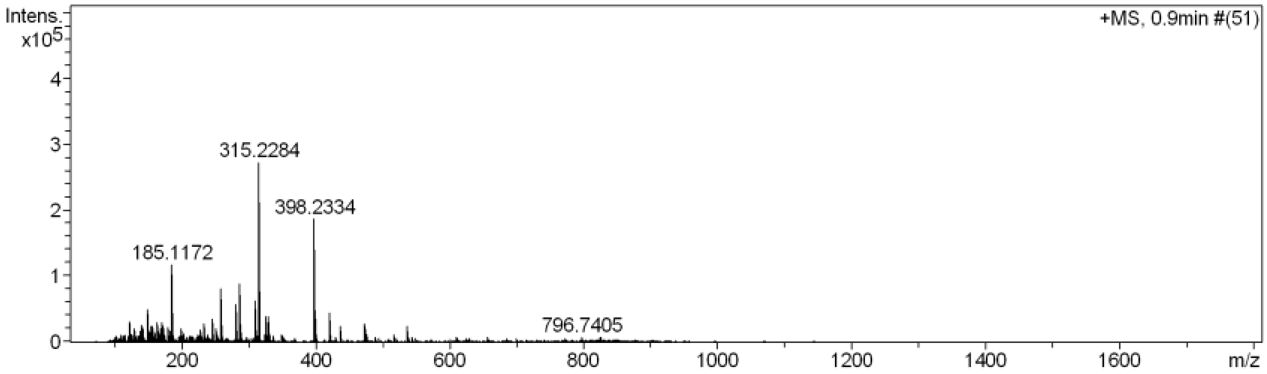


| Meas. m/z | # | Formula | Score | m/z | err [ppm] | Mean err [ppm] | mSigma | rdb | e⁻ Conf | N-Rule |
|---|---|---|---|---|---|---|---|---|---|---|
| 315.2284 | 1 | C 16 H 31 N 2 O 4 | 100.00 | 315.2278 | -1.8 | -1.7 | 19.8 | 2.5 | even | ok |

**Figure S134. Methyl (2*R*)-2-{(3*R*)-3-[(*tert*-butoxycarbonyl)amino]pyrrolidin-1-yl}-4-methylpentanoate ((2*R*,3*R*)-11c). HRMS (ESI-TOF).**

- **X-ray analysis of compound (*S*)-3b:**

Single crystals of $C_{32}H_{60}N_4O_8$ [(***S***)-**3b**] were investigated on a Rigaku, XtaLAB Synergy, Dualflex, HyPix diffractometer. The crystal was kept at 150.0(1) K during data collection. Using Olex2 [1], the structure was solved with the ShelXT [2] structure solution program using Intrinsic Phasing and refined with the olex2.refine [3] refinement package using Gauss-Newton minimisation.

**Table S1**: Experimental parameter and CCDC-2168602.

| Sample | Machine | Source | Temp. | Detector distance | Time/ Frame | #Frames | Frame width | CCDC |
|---|---|---|---|---|---|---|---|---|
| | | | [K] | [mm] | [s] | | [°] | |
| (***S***)-**3b** | Rigaku, XtaLAB Synergy, Dualflex, HyPix | μ(Cu Kα) = 0.669mm$^{-1}$ | 150 | 34 | 1.25 | 5748 | 0.50 | 2168602 |

**Table S2:** Sample and crystal data of compound (***S***)-**3b**.

| | | | | |
|---|---|---|---|---|
| **Chemical formula** | $2(C_{16}H_{30}N_2O_4)$ | **Crystal system** | Monoclinic | |
| **Formula weight [g/mol]** | 628.86 | **Space group** | *P*2$_1$ | |
| **Temperature [K]** | 150 | **Z** | 2 | |
| **Measurement method** | ϕ and ω scans | **Volume [Å$^3$]** | 1805.08(5) | |
| **Radiation vawelength [Å]** | 1.54184 | **Unit cell dimensions [Å$^3$] and [°]** | 10.0924(2)<br>12.8242(2)<br>14.0647(2) | 90<br>97.425(2)<br>90 |
| **Crystal size/ [mm$^3$]** | 0.17 × 0.11 × 0.08 | | | |
| **Crystal habit** | Block, colourless | | | |
| **Density (calculated)/ [g/cm$^3$]** | 1.1569 | **Absorption coefficient [mm$^{-1}$]** | 0.669 | |
| **Abs. Correction $T_{min}$** | 0.859 | **Abs. Correction $T_{max}$** | 0.961 | |
| **Abs. Correction type** | multi-scan *CrysAlis PRO* 1.171.40.35a (Rigaku Oxford Diffraction, 2018) Empirical absorption correction using spherical harmonics, implemented in SCALE3 ABSPACK scaling algorithm. | **F(000) [e$^-$]** | 688 | |

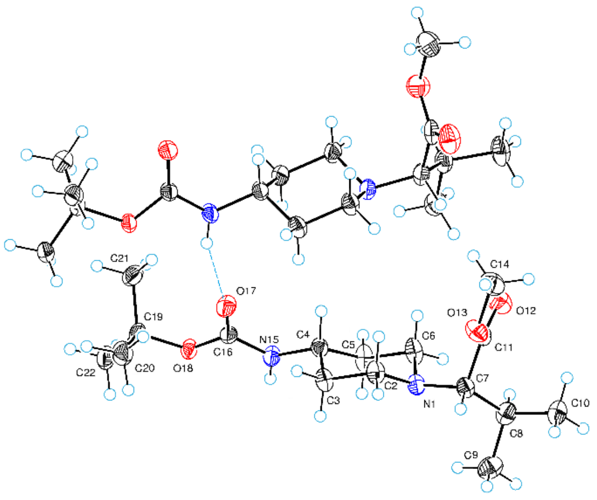


**Figure S135.** ORTEP view of assymetric unit of crystal (***S***)-**3b**.

**Table S3**: Data collection and structure refinement of compound (***S***)-**3b**.

<table>
<tr><td>Index ranges</td><td>-12 ≤ h ≤ 12<br>-16≤ k ≤ 13<br>-17 ≤ l ≤ 17</td><td>2θ range for data collection [°]</td><td colspan="2">155.0</td></tr>
<tr><td>Reflections numbers</td><td>37496</td><td>Data / restraints / parameters</td><td colspan="2">6342/1/417</td></tr>
<tr><td>Refinement method</td><td>Least squares matrix: full</td><td rowspan="2">Final R indices</td><td>All data</td><td>R1 = 0.0316;<br>wR2 = 0.0840</td></tr>
<tr><td>Function minimized</td><td>$\Sigma w[|Fo|^2 - (1/k)|Fc|^2]$</td><td>I > 2σ(I)</td><td>R1 = 0.0309;<br>wR2 = 0.0835</td></tr>
<tr><td>Goodness-of-fit on F2</td><td>1.052</td><td>Weighting scheme</td><td colspan="2">$w = 1/[\sigma^2(F_o^2) + (0.052P)^2 + 0.1876P]$<br>where $P = (F_o^2 + 2F_c^2)/3$</td></tr>
<tr><td>Largest diff. peak and hole [e Å-3]</td><td>+0.20 and -0.12</td><td>Flack's x parameter</td><td colspan="2">0.04(9)</td></tr>
</table>

- **X-ray analysis of compound (*R*)-3b:**

Single crystals of $C_{32}H_{60}N_4O_8$ [(***R***)-**3b**] were investigated on a Rigaku, XtaLAB Synergy, Dualflex, HyPix diffractometer. The crystal was kept at 150.0(1) K during data collection. Using Olex2 [1], the structure was solved with the ShelXT [2] structure solution program using Intrinsic Phasing and refined with the olex2.refine [3] refinement package using Gauss-Newton minimisation.

**Table S4**: Experimental parameter and CCDC-2168596

| Sample | Machine | Source | Temp. | Detector distance | Time/ Frame | #Frames | Frame width | CCDC |
|---|---|---|---|---|---|---|---|---|
| | | | [K] | [mm] | [s] | | [°] | |
| (***R***)-**3b** | Rigaku, XtaLAB Synergy, Dualflex, HyPix | μ(Cu Kα) = 0.669 $mm^{-1}$ | 150 | 34 | 1.25 | 5748 | 0.50 | 2168596 |

**Table S5:** Sample and crystal data of compound (***R***)-**3b**.

| | | | | |
|---|---|---|---|---|
| **Chemical formula** | $2(C_{16}H_{30}N_2O_4)$ | **Crystal system** | Monoclinic | |
| **Formula weight [g/mol]** | 628.86 | **Space group** | *P2₁* | |
| **Temperature [K]** | 150 | **Z** | 2 | |
| **Measurement method** | ϕ and ω scans | **Volume [Å³]** | 1802.25(4) | |
| **Radiation vawelength [Å]** | 1.54184 | **Unit cell dimensions [Å³] and [°]** | 10.0869(1)<br>12.8088(2)<br>14.0693(2) | 90<br>97.4889(12)<br>90 |
| **Crystal size/ [mm³]** | 0.18 × 0.11 × 0.08 | | | |
| **Crystal habit** | Colourless block | | | |
| **Density (calculated)/ [g/cm³]** | 1.1587 | **Absorption coefficient [mm⁻¹]** | 0.670 | |
| **Abs. Correction $T_{min}$** | 0.872 | **Abs. Correction $T_{max}$** | 0.960 | |
| **Abs. Correction type** | multi-scan *CrysAlis PRO* 1.171.40.35a (Rigaku Oxford Diffraction, 2018) Empirical absorption correction using spherical harmonics, implemented in SCALE3 ABSPACK scaling algorithm. | **F(000) [e⁻]** | 688 | |

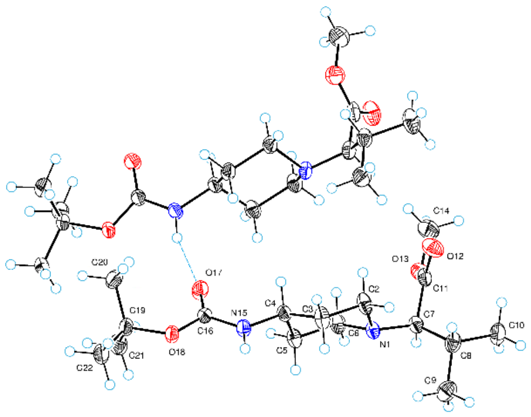


**Figure S136.** ORTEP view of assymetric unit of crystal (***R***)-**3b**.

**Table S6**: Data collection and structure refinement of compound (***R***)-**3b**.

<table>
<tr><td>Index ranges</td><td>-12 ≤ h ≤ 9<br>-16≤ k ≤ 16<br>-17 ≤ l ≤ 17</td><td>2θ range for data collection [°]</td><td colspan="2">155.0</td></tr>
<tr><td>Reflections numbers</td><td>22287</td><td>Data / restraints / parameters</td><td colspan="2">7121/1/417</td></tr>
<tr><td>Refinement method</td><td>Least squares matrix: full</td><td rowspan="2">Final R indices</td><td>All data</td><td>R1 = 0.0314;<br>wR2 = 0.0818</td></tr>
<tr><td>Function minimized</td><td>$\Sigma w[|Fo|^2 - (1/k)|Fc|^2]$</td><td>I > 2σ(I)</td><td>R1 = 0.0306;<br>wR2 = 0.0804</td></tr>
<tr><td>Goodness-of-fit on F2</td><td>1.051</td><td>Weighting scheme</td><td colspan="2">$w = 1/[\sigma^2(F_o^2) + (0.0P)^2 + 0.2072P]$<br>where $P = (F_o^2 + 2F_c^2)/3$</td></tr>
<tr><td>Largest diff. peak and hole [e Å<sup>-3</sup>]</td><td>+0.14 and -0.19</td><td>Flack's x parameter</td><td colspan="2">0.06(8)</td></tr>
</table>

- **Examples of unsuccessful HPLC analysis**

Analysis were performed using Shimadzu LC2030C chromatograph with chiral columns and water + 0.1% formic acid / acetonitrile (various gradient conditions).

**Table S7.** Experimental parameter.

| | |
|---|---|
| Mobile phase | A – Acetonitrile<br>B – Water + 0.1% formic acid |
| Temperature | 36 °C |
| Flow rate | 1 mL/min |
| Detection | UV (254, 210 nm); ELSD (Evaporative light scattering detector) |
| Injection | 10 µL |
| Sample | Dissolved in methanol |

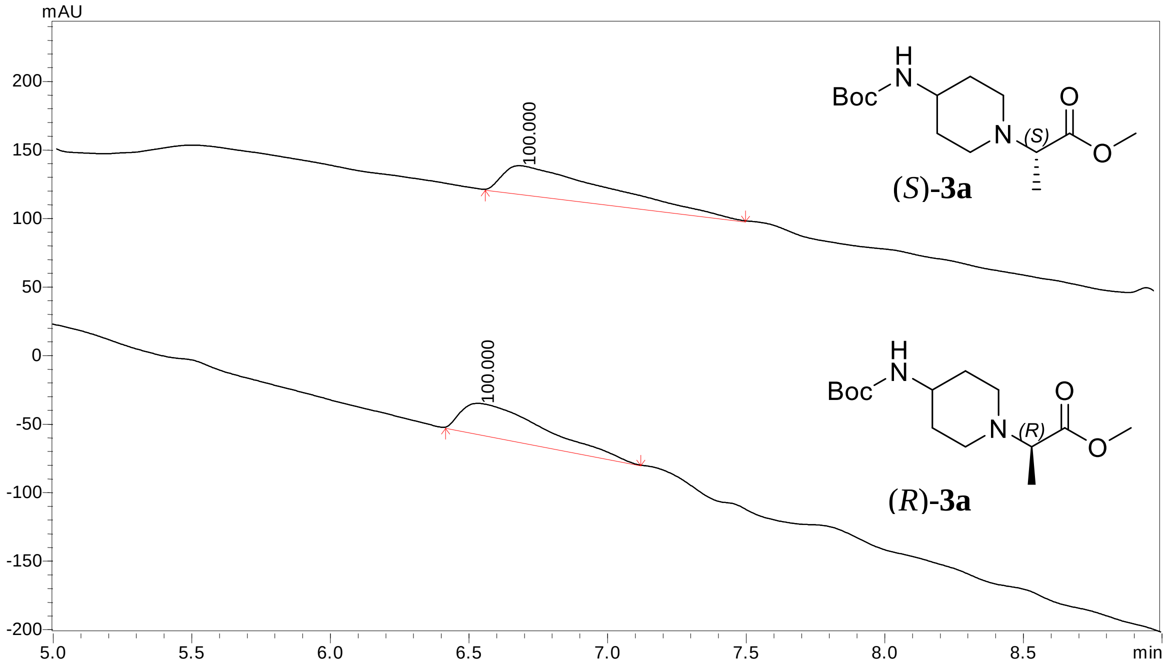


**Figure S137.** Unsuccessful chiral HPLC analysis of compounds (*S*)-**3a** and (*R*)-**3a**. Separation was carried out on YMC chiral column CHIRAL NEA (R), gradient conditions water + 0.1% formic acid / acetonitrile 90:10 to 20:80 in 15 minutes. UV detector, 210 nm.

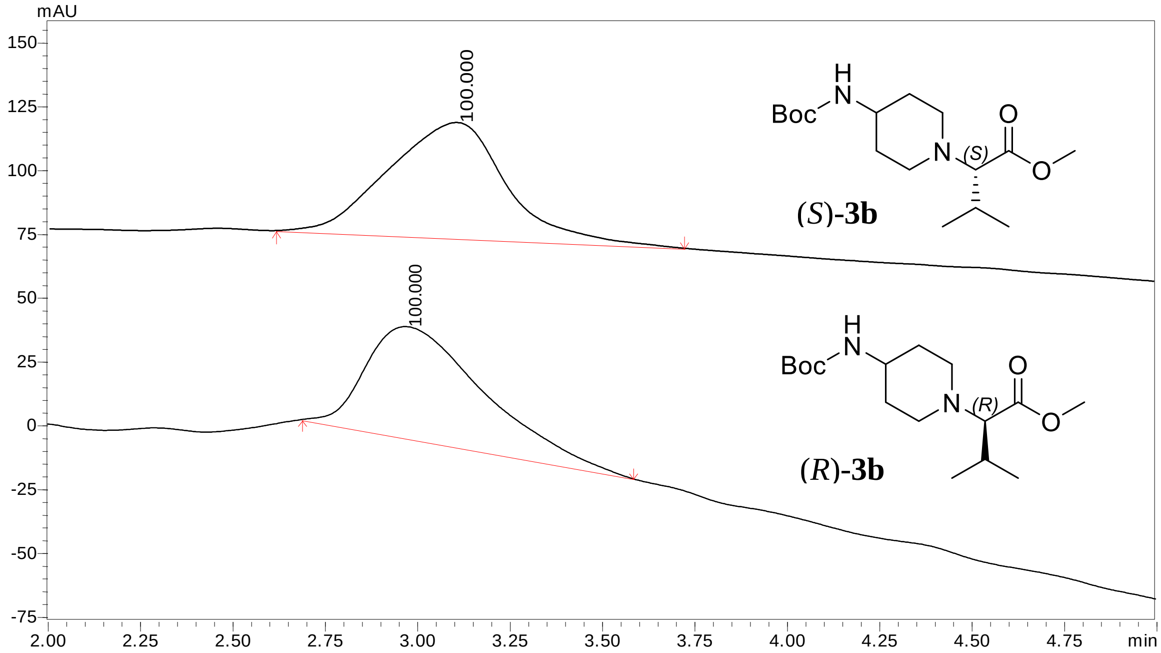


**Figure S138.** Unsuccessful chiral HPLC analysis of compounds (*S*)-**3b** and (*R*)-**3b**. Separation was carried out on chiral column CHIRAL ART Cellulose-SB, gradient conditions: water + 0.1% formic acid / acetonitrile 90:10 to 60:40 in 12 minutes. UV detector, 210 nm.

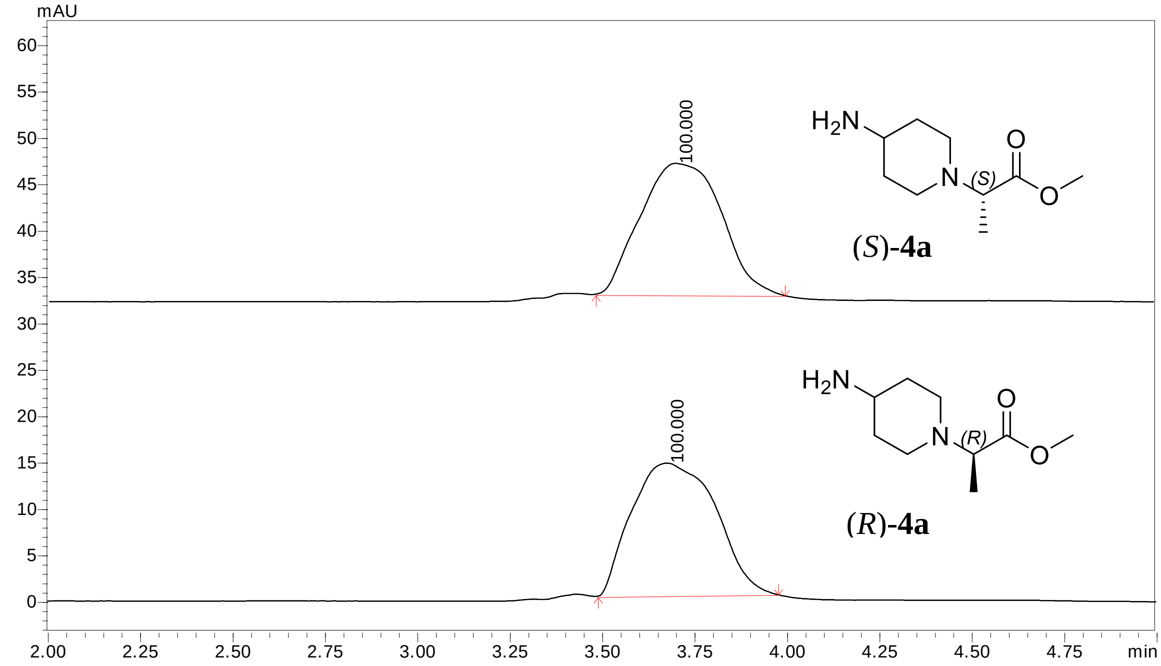


**Figure S139.** Unsuccessful chiral HPLC analysis of compounds (*S*)-**4a** and (*R*)-**4a**. Separation was carried out on YMC chiral column CHIRAL NEA (R), gradient conditions: water + 0.1% formic acid / acetonitrile 90:10 to 20:80 in 15 minutes. UV detector, 254 nm.

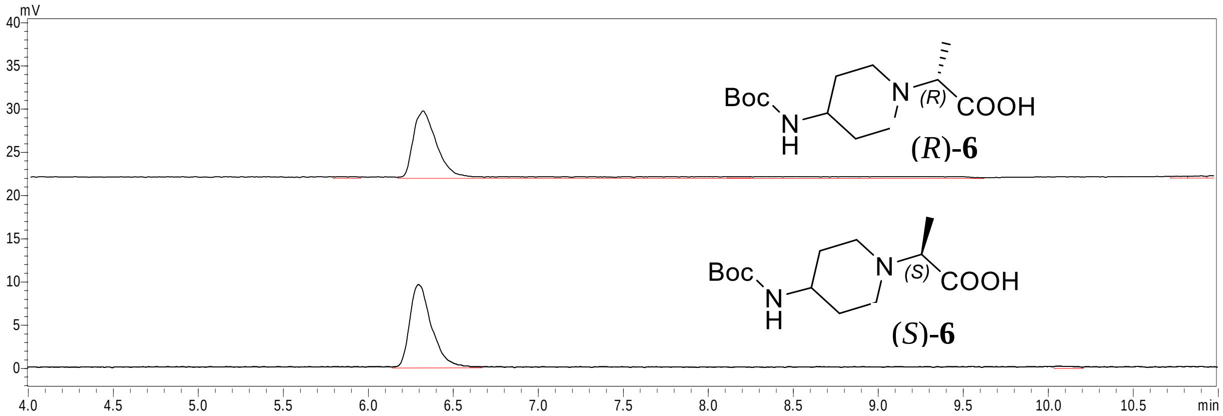


**Figure S140.** Unsuccessful chiral HPLC analysis of compounds (*S*)-**6** and (*R*)-**6**. Separation was carried out on YMC chiral column CHIRAL NEA (R), gradient conditions water + 0.1% formic acid / acetonitrile 90:10 to 20:80 in 15 minutes. Evaporative light scattering detector.

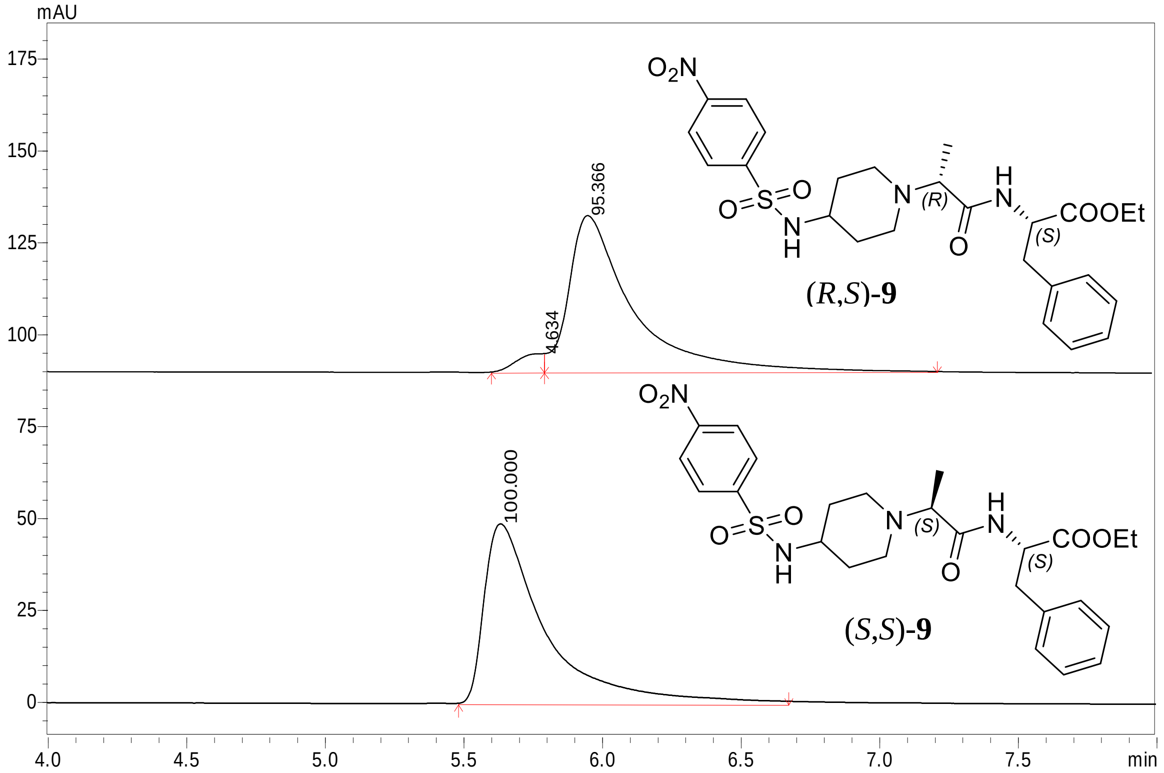


**Figure S141.** Unsuccessful chiral HPLC analysis of compounds (*S*,*S*)-**9** and (*R*,*S*)-**9**. Separation was carried out on chiral column CHIRAL ART Cellulose-SB, gradient conditions: water + 0.1% formic acid / acetonitrile 85:15 to 60:40 in 10 minutes. UV detector, 254 nm wavelength.

- **References:**


1. Dolomanov, O.V., Bourhis, L.J., Gildea, R.J, Howard, J.A.K. & Puschmann, H. (2009), J. Appl. Cryst. 42, 339-341.
2. Sheldrick, G.M. (2015). Acta Cryst. A71, 3-8.
3. Bourhis, L.J., Dolomanov, O.V., Gildea, R.J., Howard, J.A.K., Puschmann, H. (2015). Acta Cryst. A71, 59-75.
